# Identifying Virulence-Associated Genes Using Transcriptomic and Proteomic Association Analyses of the Plant Parasitic Nematode *Bursaphelenchus mucronatus*

**DOI:** 10.3390/ijms17091492

**Published:** 2016-09-07

**Authors:** Lifeng Zhou, Fengmao Chen, Hongyang Pan, Jianren Ye, Xuejiao Dong, Chunyan Li, Fengling Lin

**Affiliations:** 1 Collaborative Innovation Center of Sustainable Forestry in Southern China, College of Forestry, Nanjing Forestry University, Nanjing 210037, China; lf.zhou@njfu.edu.cn (L.Z.); jrye@njfu.edu.cn (J.Y.); 15077876285@163.com (X.D.); m15150581224@163.com (C.L.); 15751865019@163.com (F.L.); 2 Institute of Forest Protection, College of Forestry, Nanjing Forestry University, Nanjing 210037, China; 3 General Station of Forest Pest Management, The State Forestry Administration, Shenyang 110034, China; hongyangpan@163.com

**Keywords:** *Bursaphelenchus mucronatus*, virulence-associated gene, transcriptomic, proteomic

## Abstract

*Bursaphelenchus mucronatus* (*B. mucronatus*) isolates that originate from different regions may vary in their virulence, but their virulence-associated genes and proteins are poorly understood. Thus, we conducted an integrated study coupling RNA-Seq and isobaric tags for relative and absolute quantitation (iTRAQ) to analyse transcriptomic and proteomic data of highly and weakly virulent *B. mucronatus* isolates during the pathogenic processes. Approximately 40,000 annotated unigenes and 5000 proteins were gained from the isolates. When we matched all of the proteins with their detected transcripts, a low correlation coefficient of *r* = 0.138 was found, indicating probable post-transcriptional gene regulation involved in the pathogenic processes. A functional analysis showed that five differentially expressed proteins which were all highly expressed in the highly virulent isolate were involved in the pathogenic processes of nematodes. Peroxiredoxin, fatty acid- and retinol-binding protein, and glutathione peroxidase relate to resistance against plant defence responses, while β-1,4-endoglucanase and expansin are associated with the breakdown of plant cell walls. Thus, the pathogenesis of *B. mucronatus* depends on its successful survival in host plants. Our work adds to the understanding of *B. mucronatus*’ pathogenesis, and will aid in controlling *B. mucronatus* and other pinewood nematode species complexes in the future.

## 1. Introduction

The genus *Bursaphelenchus* is currently comprised of ~100 recognized species, most of which are mycophagous or plant parasitic [1,2]. A number of *Bursaphelenchus* species, including *Bursaphelenchus xylophilus* (*B. xylophilus*) and *Bursaphelenchus mucronatus* (*B. mucronatus*), are found in damaged, dying or dead coniferous trees. These species, along with a few others, which share several morphological features and biological characteristics, are known as pinewood nematode species complexes (PWNSCs) [3]. PWNSCs that originate from different regions of the world vary in their pathogenicity, but little is known about their virulence-associated gene components or their regulation in pathogenic processes [4,5,6]. However, most studies have focused on the identification and virulence testing of PWNSCs because of the limitations of traditional biotechniques. Although a number of studies have examined the factors contributing to virulence at the molecular level [7,8,9,10,11,12,13], the possible molecular mechanisms of PWNSC-associated virulence are still not very clear.

*B. mucronatus* is a PWNSC species that is a sister species to *B. xylophilus*, and it is widely distributed in natural pine forests throughout the northern hemisphere [14,15,16]. Both *B. mucronatus* and *B. xylophilus* parasitic on the living pine trees. When the hosts die, they begin to feed on fungi [16,17]. The nematodes could not leave their hosts independently and, thus, they are transferred to new hosts by insect vectors in natural conditions [18,19,20]. *B. xylophilus* complete one generation much faster than *B. mucronatus*, theirs life cycles having completed in 4–5 and 6–7 days, respectively [21]. Therefore, the virulence of *B. mucronatus* has attracted increasing attention. *B. mucronatus* was considered as nonpathogenic, or as having a very low virulence level only under stress conditions, although it had killed some pine seedlings in the past [16,22,23,24]. In recent years, research showed that the virulence of *B. mucronatus* varies to a certain degree, such as in some isolates from Eurasia that were highly virulent to pine trees under greenhouse or outdoor conditions [25,26,27,28,29]. To our knowledge, no studies have investigated the molecular events occurring during the pathogenic processes of *B. mucronatus*, and thus, determining the genes and proteins involved in the pathogenic processes of *B. mucronatus* will help us better understand the molecular events during the pathogenic processes of *B. mucronatus* and other PWNSCs.

To obtain a better understanding of the genes and proteins involved in the pathogenic processes of PWNSCs, various multilevel hierarchical approaches can be applied. Current advances in molecular biological technology, especially novel high-throughput sequencing technologies, such as RNA-Seq and isobaric tags for relative and absolute quantitation (iTRAQ), may provide valid methods for detecting the genes and proteins associated with PWNSCs’ virulence [30,31]. In iTRAQ, a multiplexed set of isobaric reagents are utilized to label the amine of peptides for the qualitative and quantitative analysis of different samples synchronously [32]. Furthermore, the isobaric reagent labelling increases tandem mass spectrometric (MS/MS) fragmentation, resulting in more reliable results than former techniques [32]. Additionally, RNA-Seq transcriptomic analyses were restricted to species having available reference genomes until the debut of de novo RNA-Seq technology [33,34]. The novel technology has been an extremely effective in rapidly and inexpensively obtaining abundant gene resources for many organisms, however, few researches on PWNSCs have been reported to this day [35]. Transcriptome analyses have been used to identify the differentially expressed genes (DEGs) involved in many physiologic processes [36,37,38]. Nevertheless, the analyses were unable to reveal the main proteins involved in the pathogenic process. Hence, in addition to a transcriptome analysis, we also surveyed proteins involved in the pathogenic processes of *B. mucronatus* by iTRAQ, which has been employed for proteomics analysis of many species [39,40,41].

The aim of the present study was to investigate the differences in both the transcriptome and proteome that occur during the pathogenic processes of two *B. mucronatus* (a low-virulence nematode species compared with *B. xylophilus*) isolates. These isolates originated from different regions and vary in their pathogenicity (Bm5, high-virulence isolate; Bm7, low-virulence compared with Bm5) [29]. The comparisons at two “omic” levels allow for a more comprehensive analysis of processes that are occurring during the pathogenesis of *B. mucronatus*.

## 2. Results

### 2.1. Identification of the Re-Isolated Nematodes

All of the re-isolated nematodes had distinct mucro of ~3–5 μm (Supplementary Figure S1) that were morphologically consistent with of the terminal mucro of *B. mucronatus* and previous research results [16]. Furthermore, a dendrogram was constructed based on internal transcribed spacer (ITS) sequences indicated that the re-isolated nematodes clustered with *B. mucronatus* (Supplementary Figure S2). Thus, the morphological characteristics combined with the phylogenetic analysis confirmed that the re-isolated nematodes in this study were *B. mucronatus*.

### 2.2. Quantitative Transcript and Protein Profiling

Bm5 (high virulence) and Bm7 (low virulence) isolates of *B. mucronatus* were used for RNA-Seq and iTRAQ analysis. The transcriptome sequencing resulted in 114,154,604 raw reads (55,168,960 and 58,985,644 reads for Bm5 and Bm7, respectively), and 107,097,176 clean reads were generated after filtering. The raw reads were deposited into the NCBI Sequence Read Archive (Study accession number SRP075786). After clustering the clean reads, we finally obtained 40,355 unigenes, including 17,851 clusters and 22,504 singletons. The unigenes had an average length of 1220 bp and N50 value of 2007 bp (Table 1). All of the 40,355 unigenes were annotated by BLASTX and BLASTN algorithms to search protein databases and nucleotide databases (*E*-value < 0.00001). In Nr, Swiss-Prot, KEGG, COG, GO, and Nt databases, 26,623, 22,507, 19,776, 12,180, 17,041, and 9615 unigenes were aligned, respectively. For gene expression determination, we used FPKM (fragments per kilobase of transcript per million mapped reads) values to calculate the gene expression levels in the different *B. mucronatus* isolates, and DEGs were defined as having a false discovery rate < 0.001 and log_2_(ratio) > 1. We determined that 16,527 genes were differentially expressed between the two isolates (Supplementary Table S1). Additionally, compared with Bm7, 4919 and 11,608 genes were up- and down-regulated in Bm5 (Supplementary Table S1).

Based on the iTRAQ proteomic analysis of the two isolates, 5092 proteins were identified from the 329,868 spectra, which included 16,205 peptides, of which 12,452 were unique. Of the 5092 proteins, 1463 were statistically significantly differentially expressed proteins (DEPs) with fold-changes > 1.5 and *p*-values < 0.05 (Supplementary Tables S2 and S3). When Bm7 served as the control, 835 of the DEPs were up-regulated and the surplus 628 were down-regulated in Bm5 (Supplementary Tables S2 and S3).

### 2.3. Validation of Gene Expression Data Using Real-Time Quantitative PCR (qRT-PCR)

**Figure 1:**
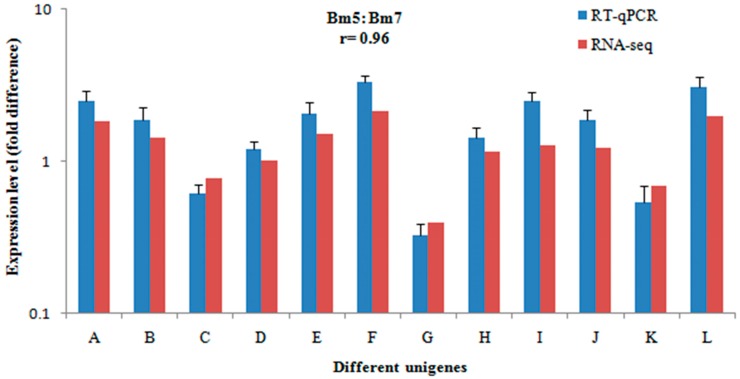
Validation of RNA-Seq data using real-time quantitative PCR (qRT-PCR). The correlations of the 12 unigenes were detected using RNA-Seq (red bars) and qRT-PCR (blue bars). The 12 column pairs (A–L) from left to right represent the following unigenes: CL8625.Contig1, CL8429.Contig1, CL5894.Contig1, CL5488.Contig2, CL5080.Contig2, CL4567.Contig1, CL3263.Contig1, CL2270.Contig1, CL2264.Contig1, Unigene2394, Unigene2628, and Unigene7511. Error bars show standard deviations.

To validate the RNA-Seq data, 12 genes were selected from the differentially abundant sequences in the transcriptome analyses and analysed by qRT-PCR. The results were consistent (*r* = 0.96) with the expression profiles estimated from RNA-Seq data (Figure 1), indicating the dependability of the RNA-Seq data.

### 2.4. Gene Ontology (GO) Analysis of Differentially Expressed Transcripts and Proteins

**Figure 2:**
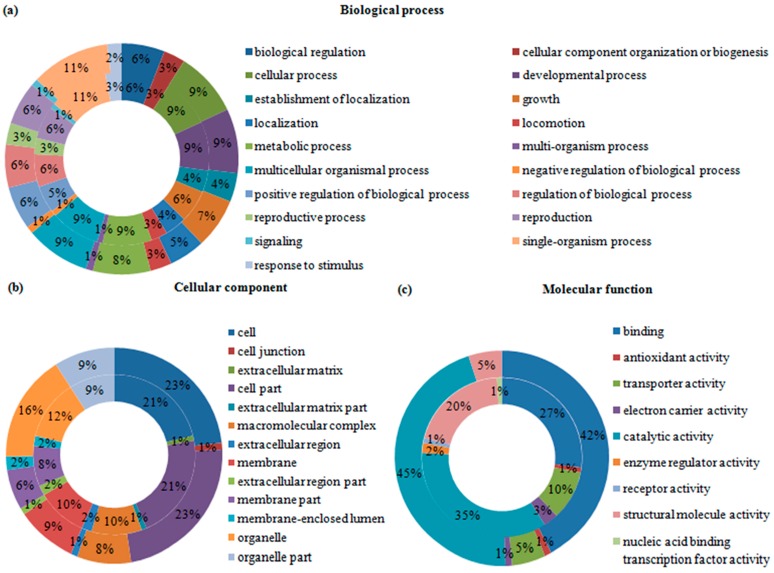
Gene ontology (GO) terms were assigned to the significantly up-regulated transcripts and proteins in highly and weakly virulent *Bursaphelenchus mucronatus* (*B. mucronatus*) isolates. The percentage of each category shown is based on the three groups: (**a**) biological process; (**b**) cellular component; and (**c**) molecular function. External ring = protein; internal ring = transcript.

**Figure 3:**
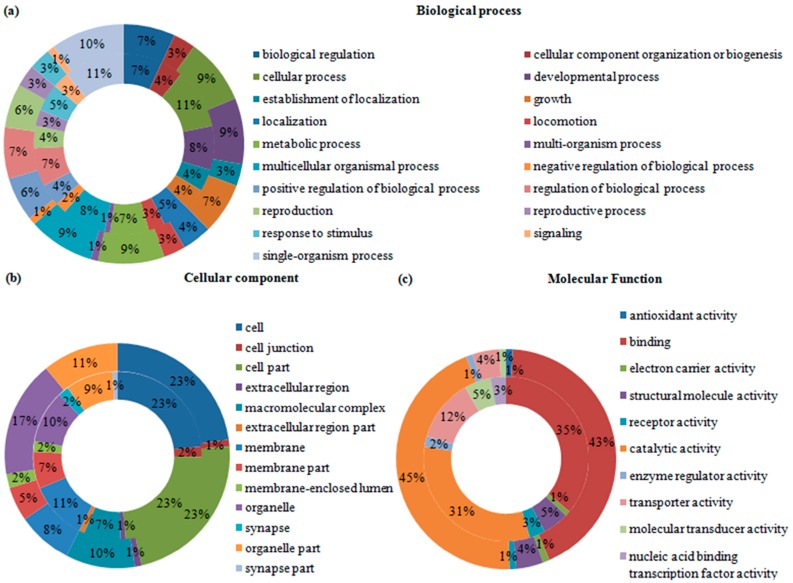
Gene ontology (GO) terms were assigned to the significantly down-regulated transcripts and proteins in highly and weakly virulent *Bursaphelenchus mucronatus* (*B. mucronatus*) isolates. The percentage of each category shown is based on the three groups: (**a**) biological process; (**b**) cellular component; and (**c**) molecular function. External ring = protein; internal ring = transcript.

Gene ontology (GO) was employed to categorize the functions of the DEGs and DEPs in the two *B. mucronatus* isolates. All of the DEGs and DEPs were categorized into three groups: biological process, cellular component, and molecular function. In the transcriptomic analysis, 17,515 GO terms were assigned to 7454 transcripts, and a single transcript might be annotated to several GO terms. Among the 17,515 GO terms, 69% fell into “biological process” group, 11% into “cellular component” group, and 20% into “molecular function” group. “Single-organism” was the largest category in the “biological processes” group, occupying 11%. “Cell” and “cell parts” occupied nearly half the “cellular components” group. In the group “molecular function”, 41% were involved in “binding” and 35% in “catalytic activity”. The percentages of the GO categories in each of the three groups are shown for up-regulated transcripts (Figure 2) and down-regulated transcripts (Figure 3). In the proteomic analysis, 586 up-regulated proteins were annotated to 3719 GO terms while 444 down-regulated proteins were annotated to 2831 GO terms. As with transcripts, a single protein might be annotated to several GO terms. The “biological process” group of up-regulated proteins contained 19 categories; “single-organism process” was the largest, accounting for 10%, followed by 9% each for “cellular process”, “developmental process”, “metabolic process”, and “multicellular organismal process“. In the group ”cellular component“, “cell”, “cell part” and “organelle” accounted for 23%, 23%, and 17%, respectively. Moreover, “molecular function” had two major categories, “catalytic activity” and “binding”, which accounted for 45% and 43%, respectively (Figure 2). Meanwhile, the component and percentages of the categories in the three groups for down-regulated proteins were like that of the up-regulated proteins (Figure 3).

### 2.5. KEGG Pathway Enrichment Analysis of Differentially Expressed Genes and Proteins

To investigate the activated biological pathways between the two *B. mucronatus* isolates, DEGs and DEPs were assigned to the reference pathways in KEGG. As a result, all of the 16,527 DEGs were mapped to 254 KEGG pathways. Sixty-three of these were significantly enriched (*p*-value < 0.05), including “calcium signalling pathway”, “salivary secretion” and “spliceosome” (Table 2). In the proteome pathway analysis, 1463 DEPs were assigned to 217 pathways. Among them, 38 pathways were significantly enriched (*p*-value < 0.05), such as “fatty acid metabolism”, “drug metabolism–cytochrome P450”, and “α-linolenic acid metabolism” (Table 3). The KEGG pathway enrichment analysis also found some enriched peptides—such as cytochrome P450s, glucuronosyltransferases, glutathione *S*-transferases, glucosyltransferases, and uridine 5′-diphosph-glucuronosyltransferases (UDP-glucuronosyltransferases, UGT)—that were involved in xenobiotic metabolism and helped nematodes adapt to life on pine hosts.

### 2.6. Correlation Analysis of Protein and RNA Expression

To examine the correlations between protein and mRNA expression levels, the proteins obtained from the *B. mucronatus* iTRAQ analysis were compared with the corresponding transcripts from the RNA-Seq analysis. When we matched all of the proteins with their detected transcripts, a low correlation coefficient of *r* = 0.138 was found, indicating that the transcriptomic and proteomic data shared a low identity (Supplementary Figure S3). In the proteomic analysis, 1463 DEPs were identified, 795 of which (54.3%) had corresponding genes in the transcriptomic analysis. Then, we detected whether the changes between proteins and transcripts were in the same direction, and found that only 135 DEPs (17%) were accordant (Supplementary Table S4). The present results indicate a low correlation between the transcript and protein abundances, probably because post-transcriptional regulation occurred in the pathological process.

### 2.7. Analysis of Pathogenesis-Related Genes

To date, many genes involved in the pathogenic processes of plant parasitic nematodes have been identified. Little is known as to whether these virulence-associated genes are related to the pathogenic processes of PWNSCs, such as *B. mucronatus*. We, therefore, used the BLAST algorithm to query the DEPs against the NCBI protein database and Uniprot, and five proteins related to pathogenic processes in other plant parasitic nematode species were obtained. Three of them, peroxiredoxin (*TPX*), fatty acid- and retinol-binding protein (*FAR*) and glutathione peroxidase (*GPX*), are related to resistance against plant defence responses, while the other two, β-1,4-endoglucanase (*ENG*) and expansin (*EXP*), are associated with the nematode breakdown of plant cell walls. In addition, all of them were detected to have corresponding genes in iTRAQ analysis based on their amino acid sequences and regulatory changes (Table 4). However, most important, all five virulence-associated proteins have higher expression levels in the high-virulence isolate Bm5 than in the low-virulence isolate Bm7.

### 2.8. Characterization and Analysis of RNAi in B. mucronatu

**Figure 4:**
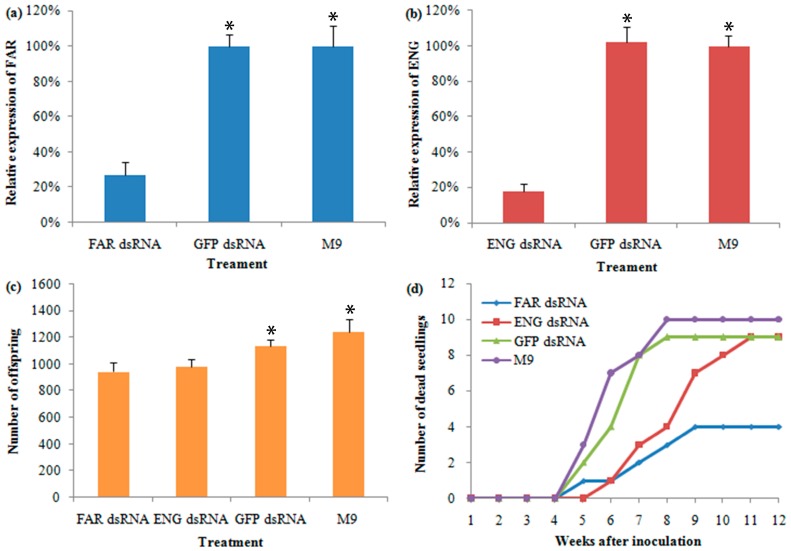
Influence of RNAi knockdowns of fatty acid- and retinol-binding protein (*FAR*) and β-1,4-endoglucanase (*ENG*) genes in *Bursaphelenchus mucronatus* (*B. mucronatus*). (**a**) mRNA expression levels of *FAR* gene in *B. mucronatus* after exposure to *FAR* dsRNA- and *GFP* dsRNA-soaking buffers (expression level of M9 was taken as 100%); (**b**) mRNA expression levels of the *ENG* gene in *B. mucronatus* after exposure to *ENG* dsRNA-soaking buffers; (**c**) influence of RNAi on the reproduction efficiency of *B. mucronatus*; (**d**) the cumulative mortality rate of *Pinus thunbergii* seedlings after inoculation with differently treated nematodes. Error bars show standard deviations. Asterisks (*) indicate a significant difference exists between target dsRNA treatments and controls (*p*-value < 0.001).

To confirm their involvement in *B. mucronatus*’ virulence, *FAR* (CL4567.Contig1) and *ENG* (CL5080.Contig2) were selected for RNAi construction because they were highly expressed at both mRNA and protein levels in the highly virulent *B. mucronatus* isolate (Bm5). Once the RNAi were constructed and introduced into the nematode, qPCR was used to assess the *FAR* and *ENG* mRNA expression levels in different treatments. By comparing the expression levels of the *FAR* gene in the nematodes exposed to the target dsRNA-soaking solution and controls, it was shown that the RNAi procedure caused a significant reduction in the target mRNA’s expression level (Figure 4a). Taking the mRNA expression level in M9 medium (non-dsRNA treated) as 100%, the average expression levels of samples with the green fluorescent protein (*GFP*) dsRNA treatment was 99.8%, while with the target dsRNA treatment it was only 26.9%. The expression levels of the *ENG* gene produced similar results after soaking in an *ENG* dsRNA solution (Figure 4b). The number of dsRNA-treated nematode offspring was used to assess the influence of RNAi on the development and propagation of *B. mucronatus*. The number of offspring from the dsRNA-treated nematodes was less than that of non-dsRNA-treated nematodes (Figure 4c). In the non-dsRNA treatment, an average of 1239 individuals in the F1 generation were produced by 20 pairs of females and males, but an average of only 945 and 985 offspring were produced in the dsRNA treatments of *FAR* and *ENG*, respectively. The difference between the target dsRNA treatment and M9 was highly significant (*p*-value < 0.001). Treatment with *GFP* dsRNA soaking resulted in an average of 1133 individuals in the F1 generation, which is a little less than that of non-dsRNA treatment, but not highly significant. However, it is highly significantly different (*p*-value < 0.001) from the target dsRNA treatment. Thus, the knockdowns of the two target genes had marked effects on the development and propagation of *B. mucronatus*. Additionally, a possible dsRNA toxicity to the nematodes was not obvious at a concentration of 800 ng/μL. To assess the influence of RNAi on the virulence of *B. mucronatus*, the dsRNA-treated nematodes were inoculated on two-year-old *Pinus thunbergii* (*P. thunbergii*) seedlings under greenhouse conditions. The first wilted seedling infected with *ENG* dsRNA-, *GFP* dsRNA-, and M9-treated nematodes were observed 5 weeks after inoculation, and the first wilted *FAR* dsRNA-treated seedling was observed 6 weeks after inoculation. All of the treatments of *B. mucronatus* caused some seedlings to wilt (ranging from 4 to 10 seedlings); while the *FAR* dsRNA-treated nematodes did not cause as many wilted seedlings as the others, and the *ENG* dsRNA-treated nematodes caused a slower wilting of seedlings than *GFP* dsRNA- and M9-treated nematodes (Figure 4d). Not a single seedling was wilted in the uninoculated control group. In addition, nematodes were re-isolated from each wilted seedling.

## 3. Discussion

PWNSC species are found in wilted coniferous trees, including *B. xylophilus*, *B. mucronatus* and a few other *Bursaphelenchus* species, causing enormous ecological and economic losses worldwide [3,6,27,42]. However, the molecular mechanisms behind the pathogenic processes of these PWNSC species are still poorly understood. *B. mucronatus* isolates are known to be vary in their level of pathogenicity [25,26,27,28,29]. With the recent advancements in RNA-Seq approach, we report the first study to investigate transcriptomic data from *B. mucronatus* isolates gained from wilted trees and we present proteomic data obtained by the iTRAQ technology. Furthermore, when the transcriptome and proteome data were coupled, we were capable of making, for the first time, integrated and precision measurements of gene and protein expression patterns in *B. mucronatus* isolates from wilted trees in the field.

Constructing an RNA-Seq transcriptome is a novel approach we have used to identify virulence-associated genes in *B. mucronatus* isolates having different virulence levels during the pathogenic processes. RNA-Seq approach has been successfully applied to detect common and different molecular idiosyncrasies between *B. xylophilus* and *B. mucronatus*, when they were cultured on fungal colonies [35]. Similar to that report, RNA-Seq data revealed a remarkable change in gene expression levels between the highly and lowly virulent isolates, indicating that the RNA-Seq is a high-efficiency technique for detecting different molecular idiosyncrasies among nematode isolates. Many proteins referred to xenobiotic biodegradation pathways were enriched in highly virulent *B. mucronatus* isolates’ transcriptomes based on the KEGG annotation (Supplementary Table S5), which was similar to the results of a previous study [35]. Recently, transcriptome and genome sequencing have been conducted on strongly virulent and weakly virulent *B. xylophilus* isolates to investigate the molecular differences between them. More than 3 Mb of genetic variations were investigated as single-nucleotide polymorphisms (SNPs) or small indels between different Japanese *B. xylophilus* isolates, and the genetic diversity was involved with their virulence and ecological characteristic differences [12]. Meanwhile, a number of transcripts and exons revealed remarkable differences between the strongly and weakly virulent Chinese *B. xylophilus* isolates, and functional study of those differences suggested that various virulence *B. xylophilus* isolates showed differences in their development, propagation, and oxidoreductase activities [43]. Similarly, there were considerable differences in strongly and weakly virulent isolates of both nematode species. It is known that *B. mucronatus* is a low-virulence nematode species compared with *B. xylophilus*, but the two *Bursaphelenchus* species have evolved similar parasitic mechanisms for living on host trees [35], which could perhaps be explained by the fact that the developmental and movement speed of *B. xylophilus* is much faster than *B. mucronatus* [21,44,45].

Meanwhile, in the proteomic profiling, we also found some enriched proteins that are involved in xenobiotic biodegradation, such as cytochrome P450s, glucuronosyltransferases, glutathione *S*-transferases and UDP-glucuronosyltransferases, based on KEGG pathway analysis (Supplementary Table S6). A global correlation analysis between iTRAQ and RNA-Seq data revealed a low correlation in *B. mucronatus* during the pathogenic processes (Supplementary Figure S3), indicating that post-transcriptional regulation may be involved in the pathogenic processes. Interestingly, in the vast majority of organisms that have been examined in recent years, transcript abundance only partially predicted protein abundance, suggesting that after experimental errors have been removed, other modes of regulation must be employed to determine the protein abundances within cells [46]. First, mRNAs are less stable, having an average half-life of less than 7 h, while that of proteins is approximate 46 h [47]. Second, experiments in living cells found that variations in protein abundance levels are primarily determined by the regulation of translation and protein degradation [47,48]. Therefore, these post-transcriptional regulations contribute to the variation in protein abundance, leading to a low correlation between the iTRAQ and RNA-Seq data.

Proteins direct the work of living cells, therefore, we used the BLAST algorithm to query the DEPs against the NCBI protein and Uniprot databases. Five DEPs were homologous to proteins with previously identified roles in general parasitic nematode pathogenic processes (Table 4). More importantly, all five of these proteins have higher expression levels in the highly virulent isolate than in the weakly virulent isolate. In addition, all five were detected to have corresponding transcripts in the RNA-Seq analysis, based on their amino acid sequences and read direction (Table 4). They were divided into two categories based on their functions: proteins related to resistance against plant defence responses and those that were associated with breaking down plant cell walls. To overcome host defence responses and survive in different tissues of the host, parasitic nematodes have evolved an ingenious strategy to manipulate the host’s metabolism to their own advantage. Three DEPs related to resistance against host defence responses were found to be more highly expressed in the highly virulent isolates than in the weakly virulent isolate of *B. mucronatus*. First, the *FAR* protein facilitates parasitic nematode infections by removing and transporting fatty acids, which are necessary for the development and reproduction of the nematodes [49]. Furthermore, the *FAR* protein also takes part in disturbing intra- and inter-cellular lipid signals which are related to host defence responses [50]. Second, *GPX* functions in parasitic nematodes by either removing hydrogen peroxide or transforming peroxidised fatty acids, which related to inhibition of lipid peroxidation [51]. It may take part in active oxygen from the in vivo metabolism, whereas the protein protects nematodes from host defence responses [52]. Finally, *TPX* particularly metabolises hydrogen peroxide and may also take part in protecting nematodes from host defences [53]. For a plant parasitic nematode that completes most of its life cycle within the plant, the plant cell wall is supposed to be its main obstacle, and the ability to get through the wall is, thus, quite important. *ENG* and *EXP* proteins, which can modify plant cell walls, were also more highly expressed in the highly virulent isolates of *B. mucronatus*. *ENG*, which degrades polysaccharides possessing β-1,4-glucan backbones (for instance, cellulose and xyloglucan) presumably facilitates nematode migration through plant tissue, and has been found among various plant parasitic nematodes [8,54,55]. *EXP* rapidly induces extensions of plant cell walls by melting the network of wall polysaccharides to allow turgor-driven cell enlargement [56]. However, plant parasitic nematodes can also produce functional expansin that is used to loosen cell walls during host plant invasion [57].

The ability to inactivate a target gene transiently by RNAi was first found in the nematode *Caenorhabditis elegans* [58]. Since then, RNAi has been a fairly effective strategy to provoke sequence-specific silencing in most eukaryotic cells [59]. In the present study, the RNAi strategy was employed to assess the influence of RNAi knockdowns of *FAR* and *ENG* genes in *B. mucronatus*. The inhibition of the seedling death rate may be explained by the *ENG* knockdown resulting in a reduced ability to locate and invade plant cells, and a decreased number of established nematodes [60,61]. The results of *ENG* gene RNAi in *B. mucronatus* are in general agreement with the previous report of its knockdown in *B. xylophilus* [59]. *FAR* is a member of the nematode-specific *FAR* family of proteins, and it exists in animal- and plant-parasitic nematodes [49,50]. After *B. mucronatus* was soaked in the *FAR* dsRNA solution, the cumulative mortality rate of its infected seedlings was sharply reduced compared with the other treatments (Figure 4d). Similar results were also found for the tomato root-knot nematode *Meloidogyne javanica* [50]. *FAR* facilitates invasion by manipulating host lipid-based defences, and this is a key component for the nematodes’ successful parasitism [62].

## 4. Materials and Methods

### 4.1. Nematode Sample Preparation

Bm5 and Bm7 isolates used in this study were originally isolated from wilted *Pinus massoniana* trees in different towns of China by the Baermann funnel technique [63] and have been maintained for over 6 years in the Institute of Forest Protection Lab in Nanjing Forestry University (Nanjing, China), have significantly different in virulence levels according to our previous studies [29]. Both isolates are from inbred lines that were established by sister-brother mating (full-sib mating repeated 10 times) and were used to generate materials for subsequent experiments. Nematodes isolates were cultured on the *Botrytis cinerea* colonies of potato-dextrose agar plates at 25 °C for 7 days and then separated for inoculation. Then, each 12-year-old *P. thunbergii* tree was inoculated with ~1 mL of nematode suspension (~10,000 of mixed-stage nematodes). The treated trees were grown under natural conditions, observed once a week, and cut when wilted. The mixed-stage nematodes were extracted from the small pieces of the wilted trees using Baermann funnel technique (place at 25 °C for 3 h) and cleaned by sucrose flotation, then rinsed three times in 1× poly(butylene succinate-*co*-butylene terephthalate) (PBST) [8,64], and promptly immersed in liquid nitrogen and deposited at −80 °C. In addition, these re-isolated nematodes were identified based on morphological and molecular analyses [65,66].

### 4.2. RNA Preparation and Transcriptome Sequencing

The RNA-Seq experiments were conducted with the assistance of the Beijing Genomics Institute (BGI, Shenzhen, China). For RNA-seq, total RNAs were extracted from the frozen samples using the RNAprep kit (Tiangen, Beijing, China) and then purified further with the RNA clean kit (Tiangen, Beijing, China) according to the manufacturer’s instructions. RNA was quantified with an Agilent 2100 Bioanalyzer RNA Nanochip (Agilent Technologies, Inc., Waldbronn, Germany). Finally, the SolexaHiSeq™ 2000 platform was employed for sequencing according to the manufacturer’s instructions (Illumina, San Diego, CA, USA).

### 4.3. Redundant Data Filtering and de Novo Assembly

Raw reads obtained from the RNA sequencing machines contained redundant reads that comprised of adapters, and unknown or inferior quality bases. These data have an adverse impact on the bioinformatics analysis and, thus, they were dumped using the following steps: (I) removed reads with adaptors; (II) removed reads with unknown nucleotides larger than 5%; (III) removed low-quality reads; and (IV) isolated the clean reads. Unigene de novo assembly was conducted with the short clean reads-assembling program Trinity [67].

### 4.4. Unigene Annotation and Classification

The unigenes annotation and classification methods and software were conducted as reported in the previous research [68]. For each isolate, the homologous gene expression ratios were counted based on the RPKM (reads per kilobase of transcript per million mapped read). The unigenes with two-fold changes between Bm5 and Bm7 were identified as DEGs.

### 4.5. qRT-PCR

To ensure the reliability of the RNA-Seq data, we selected 12 genes having different abundances in the transcriptome analysis, and validated the data by performing qRT-PCR. The *β-tubulin* gene (*β-tub*, AB500151) was used as the reference gene, and the Primer Premier 5 software (Premier Biosoft International, Palo Alto, CA, USA) was employed to design gene-specific primers for the target and reference genes (Supplementary Table S7). The total RNAs were extracted as described above and subjected to reverse transcription using the TransScript^®^ One-Step gDNA Removal and cDNA Synthesis SuperMix Kit (TransGen Biotech, Beijing, China). qRT-PCR was performed with the SYBR Green Master Mix (Vazyme, Nanjing, China) using the StepOne Plus Real-time PCR System (Applied Biosystems, Foster City, CA, USA). All of the qPCR experiments were performed with three replicates.

### 4.6. Protein Preparation and iTRAQ Labelling

The iTRAQ experiments were conducted with the help of the Beijing Genomics Institute (BGI, Shenzhen, China). The frozen nematodes were suspended in the Lysis buffer and sonicated in the ice. The proteins were reduced with 10 mM dl-dithiothreitol solution (DTT) at 56 °C for 60 min and then alkylated by 55 mM iodoacetamide under dark conditions for 60 min. After that, the reduced and alkylated protein mixtures were precipitated by adding 4× volume of chilled acetone at −20 °C overnight. After centrifugation at 30,000× *g* at 4 °C, the pellet was dissolved in 0.5 M tetraethylammonium bromide (Applied Biosystems, Milan, Italy) and sonicated in ice. After centrifuging at 30,000× *g* at 4 °C, an aliquot of the supernatant was taken to measure the protein concentration by Thermo NanoDrop 2000C (Thermo Fisher Scientific Inc., Wilmington, DE, USA). Total protein (100 µg) samples were removed, and then the proteins were digested with Trypsin Gold (Promega, Madison, WI, USA) at the protein: trypsin ratio of 30:1 at 37 °C for 16 h. After the digestion, peptides were dried and reconstituted in 0.5 M tetraethylammonium bromide. Finally, the reconstituted samples were labelled by the 8-plex iTRAQ reagent (Applied Biosystems, Foster City, CA, USA) according to the manufacturer’s instruction.

### 4.7. Strong Cation Exchange (SCX) Fractionation and LC-ESI-MS/MS Analysis

Strong Cation Exchange (SCX) fractionation and LC-ESI-MS/MS analysis were performed as reported in the previous articles [69,70].

### 4.8. Proteomic Data Analysis

The iTRAQ data analyses were conducted as reported in the previous research [71]. Proteins with a 1.5-fold change between Bm5 and Bm7 samples and *p*-value <0.05 were identified as DEPs.

### 4.9. Construction and Analysis of RNAi

In the present study, the RNAi strategy was used to evaluate the influence of RNAi knockdowns of two genes, *FAR* (CL4567.Contig1) and *ENG* (CL5080.Contig2), on *B. mucronatus*. *GFP* was used as non-endogenous control gene, and gene-specific primers for the target and control genes were designed using Primer Premier 5 software (Supplementary Table S8). In addition, M9 (42.3 mM Na_2_HPO_4_, 22 mM KH_2_PO_4_, 85.6 mM NaCl, and 1 mM MgSO_4_, pH 7.0) was used as a non-dsRNA-treated control. The dsRNAs for RNAi were synthesized using the MEGscript^®^ RNAi Kit (Ambion Inc., Austin, TX, USA) according to the manufacturer’s instructions (FAR dsRNA is 588 bp, *ENG* dsRNA is 441 bp, and *GFP* dsRNA is 670 bp). The RNAi-soaking approach utilized in the present study was conducted according to previous reports [10,59]. The RNAi efficacy was detected by qRT-PCR, and gene function was assessed by comparative fecundity and virulence assessments between the wild-type and the RNAi-treated nematodes. All of the experiments were conducted with three replicates.

## 5. Conclusions

In conclusion, we conducted transcriptomic and proteomic analyses as a complementary approach to investigate *B. mucronatus* isolates during the pathogenic processes. Lots of DEGs and DEPs were identified which are potentially involved in the pathogenic processes of *B. mucronatus*. Interestingly, the highly virulent isolate had higher expression levels of *TPX*, *FAR*, *GPX*, *ENG*, and *EXP* compared with the weakly virulent isolate. These genes are critical factors in *B. mucronatus* that are associated with breaking down plant cell walls and resisting plant defence responses in pine trees, therefore, we hypothesize that the pathogenesis of *B. mucronatus* is a result of their survival in the host tree. It is in accordance with the hypothesis which has been proposed in *B. xylophilus* researches [8,9,13]. This study increases our understanding of *B. mucronatus* pathogenesis, and may be useful in the future for controlling *B. mucronatus* and other PWNSC species.

## Figures and Tables

**Table 1:** Transcriptome and proteome data in *Bursaphelenchus mucronatus* (*B. mucronatus*).

Transcriptome Data		Proteome Data	
Raw reads (pair)	114,154,604	Total spectra	329,868
Clean reads (pair)	107,097,176	Peptide number	16,205
Total nucleotide length (bp)	9,638,745,840	Unique number	12,452
Unigene number	40,355	Protein number	5092
Average length (bp)	1220	-	-
N50 length (bp)	2007	-	-

**Table 2:** KEGG pathways’ enrichment analysis in the *Bursaphelenchus mucronatus* (*B. mucronatus*) transcriptome.

Pathway	DEGs ^1^	Genes ^2^	*p*-Value	Pathway ID
Ribosome	358	520	4.35 × 10^−^^30^	ko03010
Amoebiasis	362	576	1.50 × 10^−^^19^	ko05146
Pathogenic Escherichia coli infection	177	280	1.50 × 10^−^^10^	ko05130
Dilated cardiomyopathy	213	356	2.76 × 10^−^^9^	ko05414
Focal adhesion	452	837	8.36 × 10^−^^9^	ko04510
Alzheimer’s disease	295	521	9.96 × 10^−^^9^	ko05010
Huntington’s disease	288	515	7.34 × 10^−^^8^	ko05016
Cardiac muscle contraction	222	387	1.67 × 10^−^^7^	ko04260
Hypertrophic cardiomyopathy	203	352	3.27 × 10^−^^7^	ko05410
Viral myocarditis	153	255	3.55 × 10^−^^7^	ko05416
Extracellular matrix-receptor interaction	255	458	6.56 × 10^−^^7^	ko04512
Tight junction	262	475	1.29 × 10^−^^6^	ko04530
Oxidative phosphorylation	242	436	1.72 × 10^−^^6^	Ko00190
Salmonella infection	204	362	2.81 × 10^−^^6^	Ko05132
Melanogenesis	124	208	6.87 × 10^−^^6^	Ko04916
Parkinson’s disease	238	435	8.45 × 10^−^^6^	Ko05012
Salivary secretion	162	284	1.14 × 10^−^^5^	Ko04970
Gastric acid secretion	187	338	3.19 × 10^−^^5^	Ko04971
Endocytosis	223	411	3.30 × 10^−^^5^	Ko04144
Phagosome	181	328	5.14 × 10^−^^5^	Ko04145
Endocrine and other factor-regulated calcium reabsorption	99	166	5.43 × 10^−^^5^	Ko04961
Calcium signalling pathway	260	494	1.26 × 10^−^^4^	Ko04020
Vibrio cholerae infection	188	349	2.19 × 10^−^^4^	Ko05110
Ribosome biogenesis in eukaryotes	141	256	3.66 × 10^−^^4^	Ko03008
Vascular smooth muscle contraction	332	654	5.38 × 10^−^^4^	Ko04270
African trypanosomiasis	22	29	5.83 × 10^−^^4^	Ko05143
GnRH signalling pathway	133	242	5.94 × 10^−^^4^	Ko04912
Aminoacyl-tRNA biosynthesis	73	123	5.97 × 10^−^^4^	Ko00970
Cholinergic synapse	109	194	6.21 × 10^−^^4^	Ko04725
Chemokine signalling pathway	159	296	7.57 × 10^−^^4^	Ko04062
Antigen processing and presentation	98	175	1.31 × 10^−^^3^	Ko04612
RNA transport	238	463	1.34 × 10^−^^3^	Ko03013
Herpes simplex infection	160	303	1.92 × 10^−^^3^	Ko05168
Hedgehog signalling pathway	61	104	2.40 × 10^−^^3^	Ko04340
Staphylococcus aureus infection	26	38	2.42 × 10^−^^3^	Ko05150
Transcriptional misregulation in cancer	165	317	3.57 × 10^−^^3^	Ko05202
Phototransduction	71	126	4.61 × 10^−^^3^	Ko04744
Prion diseases	72	128	4.62 × 10^−^^3^	Ko05020
Pancreatic secretion	133	255	7.53 × 10^−^^3^	Ko04972
Morphine addiction	98	183	7.88 × 10^−^^3^	Ko05032
Sulphur relay system	32	53	1.40 × 10^−^^2^	Ko04122
Olfactory transduction	109	209	1.44 × 10^−^^2^	Ko04740
Bacterial invasion of epithelial cells	131	255	1.47 × 10^−^^2^	Ko05100
Spliceosome	274	559	1.50 × 10^−^^2^	Ko03040
Long-term depression	71	131	1.52 × 10^−^^2^	Ko04730
GABAergic synapse	110	212	1.69 × 10^−^^2^	Ko04727
Dopaminergic synapse	148	292	1.76 × 10^−^^2^	Ko04728
Legionellosis	73	136	1.85 × 10^−^^2^	Ko05134
Protein digestion and absorption	303	624	1.93 × 10^−^^2^	Ko04974
Glutamatergic synapse	112	218	2.25 × 10^−^^2^	Ko04724
Toxoplasmosis	100	193	2.28 × 10^−^^2^	Ko05145
Retrograde endocannabinoid signalling	111	216	2.29 × 10^−^^2^	Ko04723
Arrhythmogenic right ventricular cardiomyopathy (ARVC)	63	117	2.51 × 10^−^^2^	Ko05412
Type I diabetes mellitus	8	10	2.51 × 10^−^^2^	Ko04940
Neuroactive ligand-receptor interaction	223	455	2.61 × 10^−^^2^	Ko04080
Influenza A	155	310	2.68 × 10^−^^2^	Ko05164
Gap junction	109	214	3.19 × 10^−^^2^	Ko04540
Shigellosis	90	176	4.27 × 10^−^^2^	Ko05131
ErbB signalling pathway	99	195	4.30 × 10^−^^2^	Ko04012
Fc gamma receptor-mediated phagocytosis	115	229	4.41 × 10^−^^2^	Ko04666
Synaptic vesicle cycle	110	219	4.77 × 10^−^^2^	Ko04721
mRNA surveillance pathway	169	345	4.81 × 10^−^^2^	Ko03015
Regulation of actin cytoskeleton	363	766	4.98 × 10^−^^2^	Ko04810

^1^ DEGs: Differentially expressed genes which involve in each KEGG pathway; ^2^ Genes: Expressed genes which involve in each KEGG pathway.

**Table 3:** KEGG pathways enrichment analysis in the *Bursaphelenchus mucronatus* (*B. mucronatus*) proteome.

Pathway	DEPs ^1^	Proteins ^2^	*p*-Value	Pathway ID
Metabolic pathways	444	1119	2.96 × 10^−^^18^	Ko01100
Valine, leucine and isoleucine degradation	61	111	1.39 × 10^−^^8^	Ko00280
Glycolysis/Gluconeogenesis	67	134	3.60 × 10^−^^7^	Ko00010
Fatty acid metabolism	67	136	7.28 × 10^−^^7^	Ko00071
Pyruvate metabolism	51	98	2.05 × 10^−^^6^	Ko00620
Citrate cycle (TCA cycle)	42	76	2.17 × 10^−^^7^	Ko00020
β-Alanine metabolism	38	70	1.17 × 10^−^^5^	Ko00410
Aminoacyl-tRNA biosynthesis	26	42	1.29 × 10^−^^5^	Ko00970
Oxidative phosphorylation	71	160	3.72 × 10^−^^5^	Ko00190
Propanoate metabolism	42	84	5.74 × 10^−^^5^	Ko00640
Parkinson’s disease	63	150	6.58 × 10^−^^4^	Ko05012
Peroxisome	71	174	8.22 × 10^−^^4^	Ko04146
Alzheimer’s disease	67	167	1.93 × 10^−^^3^	Ko05010
Butanoate metabolism	28	58	1.95 × 10^−^^3^	Ko00650
Retinol metabolism	48	113	2.13 × 10^−^^3^	Ko00830
Tryptophan metabolism	41	94	2.44 × 10^−^^3^	Ko00380
Galactose metabolism	21	41	2.85 × 10^−^^3^	Ko00052
Huntington’s disease	72	184	2.89 × 10^−^^3^	Ko05016
Arginine and proline metabolism	40	92	2.94 × 10^−^^3^	Ko00330
Glyoxylate and dicarboxylate metabolism	23	47	3.89 × 10^−^^3^	Ko00630
Peroxisome proliferator-activated signalling pathway	29	63	3.99 × 10^−^^3^	Ko03320
Metabolism of xenobiotics by cytochrome P450	48	116	4.02 × 10^−^^3^	Ko00980
Pentose phosphate pathway	23	48	5.42 × 10^−^^3^	Ko00030
Drug metabolism–cytochrome P450	44	107	6.59 × 10^−^^3^	Ko00982
Lysine degradation	27	60	7.82 × 10^−^^3^	Ko00310
Biosynthesis of unsaturated fatty acids	17	34	9.56 × 10^−^^3^	Ko01040
Glutathione metabolism	40	98	1.08 × 10^−^^2^	Ko00480
Phenylalanine metabolism	26	59	1.23 × 10^−^^2^	Ko00360
Glycerolipid metabolism	26	59	1.23 × 10^−^^2^	Ko00561
Starch and sucrose metabolism	22	49	1.61 × 10^−^^2^	Ko00500
Synthesis and degradation of ketone bodies	7	11	1.99 × 10^−^^2^	Ko00072
Alanine, aspartate and glutamate metabolism	20	45	2.40 × 10^−^^2^	Ko00250
Tyrosine metabolism	27	65	2.58 × 10^−^^2^	Ko00350
Mismatch repair	4	5	2.92 × 10^−^^2^	Ko03430
Pentose and glucuronate inter conversions	33	83	2.93 × 10^−^^2^	Ko00040
Arachidonic acid metabolism	30	75	3.36 × 10^−^^2^	Ko00590
Lysosome	47	128	4.70 × 10^−^^2^	Ko04142
α-Linolenic acid metabolism	11	23	4.91 × 10^−^^2^	Ko00592

^1^ DEPs: Differentially expressed proteins which involve in each KEGG pathway; ^2^ Proteins: Expressed proteins which involve in each KEGG pathway.

**Table 4:** Putative virulence-associated genes in *Bursaphelenchus mucronatus* (*B. mucronatus*).

Gene ID	Annotation	Fold Change Bm5:Bm7	
		Protein	RNA
Uni5244	Glutathione peroxidase	3.65	1.60
CL3566.Contig2	Expansin-like protein	1.79	1.45
CL4567.Contig1	Fatty acid- and retinol-binding protein	11.66	1.94
CL5080.Contig2	β-1,4-Endoglucanase	5.34	1.51
CL8429.Contig1	Peroxiredoxin	4.38	1.41

## Supplementary Materials

Supplementary materials can be found at www.mdpi.com/1422-0067/17/9/1492/s1.
