# Supplementary material for: Identifying Virulence-Associated Genes Using Transcriptomic and Proteomic Association Analyses of the Plant Parasitic Nematode Bursaphelenchus mucronatus
Source: Int J Mol Sci. 2016 Sep 7;17(9):1492. doi: 10.3390/ijms17091492 (PMC5037770; doi:10.3390/ijms17091492)
Supplement: Supplementary file 1 [file ijms-17-01492-s001.zip › ijms-139092-Supplementary Materials/ijms-139092-supplymentary figures and Tables S4-S8 proofreaded.pdf]

Supplementary Materials: Identifying Virulence-Associated Genes Using Transcriptomic and Proteomic Association Analyses of the Plant Parasitic Nematode *Bursaphelenchus mucronatus*

Lifeng Zhou, Fengmao Chen, Hongyang Pan, Jianren Ye, Xuejiao Dong, Chunyan Li and Fengling Lin

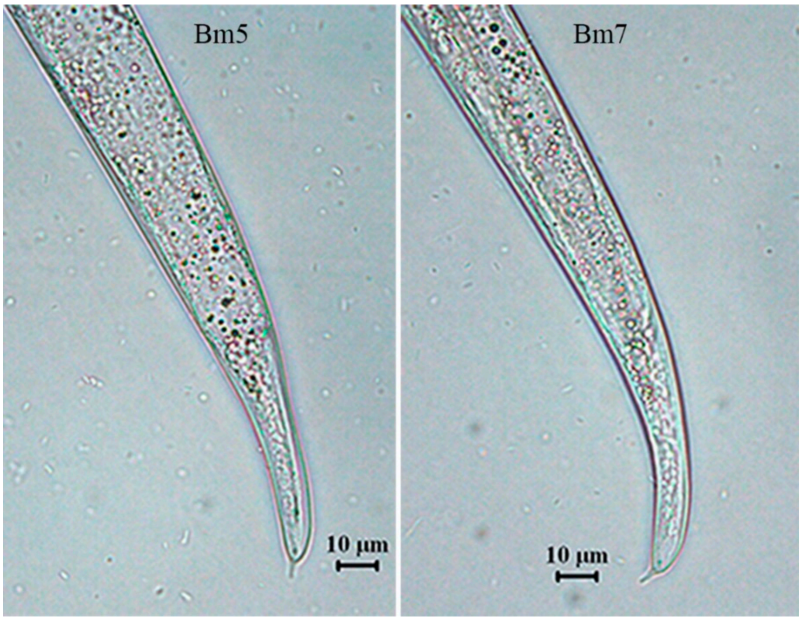

Figure S1. Photomicrographs showing the terminal mucro of the two *B. mucronatus* isolates.

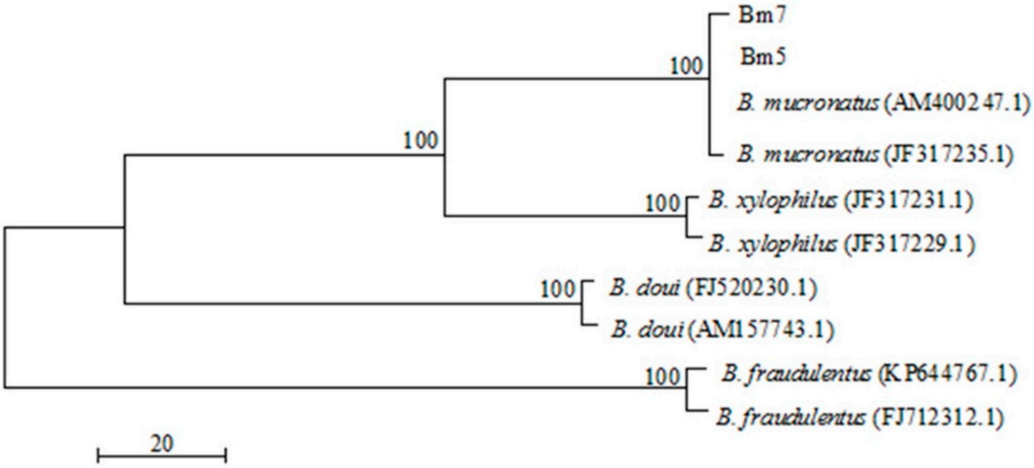

Figure S2. Phylogenetic tree of the two nematode isolates based on their internal transcribed spacer (ITS) sequences and that of four *Bursaphelenchus* species.

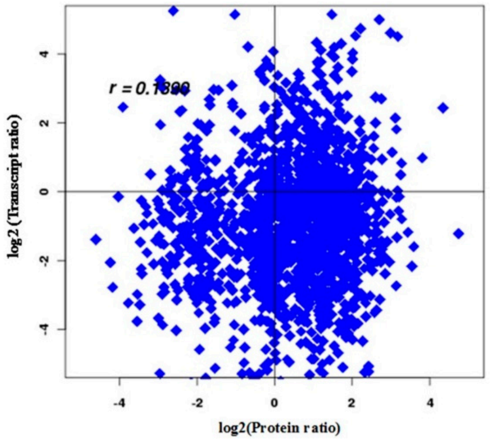

Figure S3. Correlation of protein and mRNA levels in *B. mucronatus*.

Table S4. Differential expression proteins and differential expression genes were in the same direction (either up or down).

Protein	Protein Quantition (Bm5/Bm7)	Gene Quantitionlog2 (Bm5/Bm7)	Fdr	diff
Unigene5099_All	4.596	1.723270188	2.52×10^{-68}	+
CL1521.Contig2_All	0.453	−4.524521727	5.06×10^{-6}	-
CL4602.Contig1_All	3.433	1.463417323	3.00×10^{-7}	+
Unigene11200_All	4.235	4.522907959	3.74×10^{-6}	+
CL3279.Contig1_All	0.055	−2.784541118	3.58×10^{-6}	-
Unigene1879_All	3.183	2.35834097	2.58×10^{-69}	+
Unigene518_All	2.889	3.384299994	8.99×10^{-21}	+
CL2220.Contig2_All	0.212	−2.64689025	5.54×10^{-10}	-
CL3787.Contig2_All	2.129	2.349444402	1.64×10^{-17}	+
CL2825.Contig2_All	0.299	−2.827380033	2.04×10^{-6}	-
Unigene3119_All	2.36	3.148094253	0	+
CL3388.Contig1_All	0.207	−1.236009293	2.09×10^{-10}	-
Unigene816_All	3.245	1.220040673	0	+
CL4434.Contig1_All	0.172	−4.031658084	2.89×10^{-11}	-
Unigene3383_All	2.538	1.023359726	0	+
CL4841.Contig2_All	0.272	−1.996926327	6.10×10^{-8}	-
Unigene6964_All	1.713	2.887065527	0	+
Unigene3207_All	3.286	4.289922182	0	+
Unigene12118_All	2.372	12.36888837	1.19×10^{-8}	+
Unigene917_All	3.5	2.102744668	3.68×10^{-31}	+
Unigene1752_All	0.247	−2.768538381	0	-
Unigene5837_All	1.956	1.013823468	7.80×10^{-12}	+
Unigene8364_All	1.606	2.309994064	1.09×10^{-11}	+
CL2312.Contig1_All	0.192	−9.259037041	5.33×10^{-5}	-
Unigene4571_All	3.721	1.082402147	8.31×10^{-15}	+
CL1393.Contig2_All	2.288	3.10791162	1.68×10^{-11}	+
Unigene7838_All	0.36	−1.256647206	2.75×10^{-12}	-
Unigene2609_All	0.134	−2.036025556	4.67×10^{-13}	-
CL2047.Contig2_All	0.181	−9.153298683	2.70×10^{-5}	-
Unigene8379_All	2.01	1.988572286	6.70×10^{-4}	+
CL3063.Contig1_All	0.254	−1.76894171	1.17×10^{-91}	-
Unigene7452_All	0.293	−3.041774758	2.67×10^{-13}	-

Unigene18768_All	0.258	−11.8473706	3.13×10^{-12}	-
CL3469.Contig5_All	0.328	−2.686477752	1.35×10^{-6}	-
Unigene5292_All	2.005	1.584937987	0	+
Unigene3722_All	0.2	−1.460350097	0	-
Unigene10607_All	7.04	10.69740201	1.50×10^{-6}	+
CL5015.Contig1_All	2.363	1.528488236	5.47×10^{-56}	+
Unigene7817_All	2.172	1.219507525	7.44×10^{-16}	+
CL3471.Contig2_All	0.139	−2.903368661	3.06×10^{-15}	-
Unigene1357_All	0.272	−1.260321281	6.09×10^{-25}	-
CL3666.Contig1_All	0.086	−2.98516452	1.67×10^{-16}	-
CL3542.Contig2_All	0.38	−2.850457012	8.76×10^{-26}	-
Unigene11218_All	4.02	11.55161214	1.20×10^{-14}	+
Unigene936_All	3.208	1.625188933	0	+
Unigene7663_All	1.88	1.403618816	0	+
Unigene5221_All	1.836	2.012118245	1.09×10^{-72}	+
CL5411.Contig2_All	0.138	−2.773890033	3.98×10^{-31}	-
CL2676.Contig1_All	0.125	−3.676776745	5.39×10^{-11}	-
Unigene7737_All	0.183	−1.692996527	6.59×10^{-69}	-
Unigene8544_All	4.609	1.99414376	1.54×10^{-27}	+
Unigene7882_All	1.507	1.980910585	5.40×10^{-25}	+
Unigene5671_All	2.508	2.89177294	0	+
CL1349.Contig1_All	2.069	1.593332494	1.09×10^{-5}	+
Unigene5006_All	2.743	1.548866142	4.75×10^{-21}	+
Unigene4972_All	3.046	1.743653549	0	+
Unigene5774_All	2.247	1.040686422	4.40×10^{-6}	+
Unigene7816_All	2.769	2.081529377	2.16×10^{-47}	+
CL5445.Contig2_All	2.62	1.466409867	6.89×10^{-11}	+
CL3920.Contig2_All	0.23	−2.957486241	6.15×10^{-44}	-
CL4520.Contig1_All	3.594	12.05985141	4.49×10^{-10}	+
CL4233.Contig1_All	0.63	−1.332132346	6.13×10^{-6}	-
CL3833.Contig2_All	0.195	−1.037796077	8.59×10^{-6}	-
Unigene4651_All	0.21	−2.762411646	1.32×10^{-25}	-
CL477.Contig1_All	2.519	15.43549447	2.59×10^{-56}	+
CL4807.Contig1_All	2.034	12.40577968	1.20×10^{-14}	+
Unigene8425_All	2.141	3.774518662	6.04×10^{-41}	+
Unigene8344_All	1.567	3.300875754	0	+
Unigene18266_All	0.576	−4.000440989	5.49×10^{-11}	-
Unigene9872_All	1.687	2.249200963	2.59×10^{-64}	+
CL152.Contig2_All	0.273	−1.468562505	3.34×10^{-44}	-
CL5250.Contig1_All	0.657	−11.17573666	1.36×10^{-5}	-
Unigene4416_All	5.171	1.033065258	0	+
Unigene6078_All	0.085	−3.765484578	0	-
CL4316.Contig2_All	0.235	−1.702461015	1.44×10^{-5}	-
Unigene8408_All	2.502	1.729414841	9.39×10^{-5}	+
CL2368.Contig1_All	1.755	1.828087957	9.40×10^{-32}	+
Unigene6753_All	0.435	−1.161753016	0	-
Unigene12944_All	3.073	10.77000309	2.84×10^{-5}	+
CL1555.Contig2_All	0.225	−5.554709379	7.89×10^{-25}	-
Unigene8377_All	0.168	−2.686543802	2.80×10^{-12}	-
Unigene15532_All	0.587	−4.521463064	5.07×10^{-6}	-
Unigene4866_All	3.098	3.25994237	1.66×10^{-80}	+
Unigene2198_All	9.041	4.508872749	0	+

CL2595.Contig1_All	4.69	1.307882885	0	+
CL4242.Contig1_All	0.135	−3.869728975	7.90×10^{-4}	-
CL2751.Contig2_All	0.251	−2.554052115	5.53×10^{-14}	-
Unigene4171_All	3.963	1.508763112	9.71×10^{-29}	+
Unigene636_All	6.058	1.996905085	1.14×10^{-13}	+
Unigene7958_All	0.241	−1.563677495	1.32×10^{-90}	-
Unigene6172_All	2.299	3.775971382	0	+
CL4169.Contig1_All	0.122	−1.379472793	2.99×10^{-11}	-
CL5426.Contig1_All	0.26	−4.585133873	2.65×10^{-6}	-
Unigene2628_All	3.672	2.790465783	1.40×10^{-20}	+
CL827.Contig2_All	0.189	−1.284312981	5.68×10^{-7}	-
CL3876.Contig1_All	1.536	2.049552103	1.11×10^{-13}	+
CL3787.Contig1_All	2.912	4.185763469	5.94×10^{-9}	+
CL1188.Contig2_All	0.222	−1.004005634	7.84×10^{-4}	-
Unigene7182_All	3.986	3.495924139	8.99×10^{-21}	+
CL4990.Contig2_All	0.642	−11.46209323	4.03×10^{-4}	-
CL731.Contig1_All	0.182	−1.836004402	2.46×10^{-17}	-
Unigene2932_All	0.153	−2.126150701	1.18×10^{-11}	-
9CL918.Contig3_All	0.474	−2.869697298	3.98×10^{-10}	-
CL846.Contig2_All	0.266	−3.015984624	2.95×10^{-6}	-
Unigene2081_All	0.277	−3.033619909	0	-
Unigene776_All	2.739	2.689053679	0	+
Unigene4940_All	2.036	2.528609427	1.68×10^{-70}	+
Unigene5927_All	0.623	−1.873163964	3.23×10^{-38}	-
Unigene2447_All	4.653	4.72863827	0	+
CL4768.Contig1_All	2.822	1.825873141	5.45×10^{-79}	+
Unigene10193_All	1.719	11.89129007	1.21×10^{-12}	+
Unigene110_All	2.515	2.28784217	3.59×10^{-62}	+
CL5135.Contig1_All	0.219	−1.189702902	5.28×10^{-4}	-
CL924.Contig2_All	0.307	−2.52142377	3.07×10^{-29}	-
CL5150.Contig1_All	0.244	−9.239121132	5.32×10^{-5}	-
Unigene9846_All	1.754	2.855470488	0	+
Unigene9538_All	2.144	3.081704627	1.03×10^{-55}	+
Unigene3371_All	4.018	1.322974996	2.64×10^{-31}	+
CL1117.Contig2_All	0.528	−1.811884428	1.80×10^{-4}	-
CL3034.Contig2_All	0.216	−2.510042029	0	-
CL454.Contig2_All	2.206	12.05263597	4.48×10^{-14}	+
CL5348.Contig1_All	0.483	−2.469665996	3.20×10^{-37}	-
Unigene1544_All	2.05	1.020429219	0	+
CL1554.Contig2_All	2.698	1.789916426	5.82×10^{-14}	+
CL1735.Contig2_All	0.276	−2.949575182	5.38×10^{-26}	-
Unigene703_All	8.136	1.143461235	1.77×10^{-70}	+
Unigene8638_All	8.646	2.126552005	0	+
CL3920.Contig3_All	0.174	−3.149478214	5.10×10^{-7}	-
Unigene2623_All	1.824	2.03487808	5.52×10^{-9}	+
Unigene6108_All	2.08	1.816755907	0	+
Unigene11930_All	3.211	11.95077458	2.84×10^{-5}	+
CL2320.Contig1_All	2.839	16.09505081	0	+
Unigene680_All	2.249	2.301898073	2.52×10^{-77}	+
CL1534.Contig2_All	0.344	−3.676561673	5.38×10^{-11}	-
Unigene21428_All	0.288	−10.4604559	2.05×10^{-4}	-

Table S5. KEGG pathway enrichment analysis of differentially expressed transcripts in high virulence (Bm5) and low virulence (Bm7) *Bursaphelenchus mucronarus* isolates.

#	Pathway	DEGs Genes with Pathway Annotation (8785)	All genes with Pathway Annotation (19776)	*p* Value	*Q* Value	Pathway ID	Level 1	Level 2
1	Ribosome	358 (4.08%)	520 (2.63%)	4.349155e-30	1.104685e-27	ko03010	Genetic Information Processing	Translation
2	Amoebiasis	362 (4.12%)	576 (2.91%)	1.499134e-19	1.903900e-17	ko05146	Human Diseases	Infectious diseases: Parasitic
3	Pathogenic Escherichia coli infection	177 (2.01%)	280 (1.42%)	1.493591e-10	1.264574e-08	ko05130	Human Diseases	Infectious diseases: Bacterial
4	Dilated cardiomyopathy	213 (2.42%)	356 (1.8%)	2.758634e-09	1.751733e-07	ko05414	Human Diseases	Cardiovascular diseases
5	Focal adhesion	452 (5.15%)	837 (4.23%)	8.358826e-09	4.214419e-07	ko04510	Cellular Processes	Cell communication
6	Alzheimer's disease	295 (3.36%)	521 (2.63%)	9.955321e-09	4.214419e-07	ko05010	Human Diseases	Neurodegenerative diseases
7	Huntington's disease	288 (3.28%)	515 (2.6%)	7.343513e-08	2.664646e-06	ko05016	Human Diseases	Neurodegenerative diseases
8	Cardiac muscle contraction	222 (2.53%)	387 (1.96%)	1.667616e-07	5.294681e-06	ko04260	Organismal Systems	Circulatory system
9	Hypertrophic cardiomyopathy (HCM)	203 (2.31%)	352 (1.78%)	3.274355e-07	9.013662e-06	ko05410	Human Diseases	Cardiovascular diseases
10	Viral myocarditis	153 (1.74%)	255 (1.29%)	3.548686e-07	9.013662e-06	ko05416	Human Diseases	Cardiovascular diseases
11	ECM-receptor interaction	255 (2.9%)	458 (2.32%)	6.558946e-07	1.514520e-05	ko04512	Environmental Information Processing	Signaling molecules and interaction
12	Tight junction	262 (2.98%)	475 (2.4%)	1.29183e-06	2.734374e-05	ko04530	Cellular Processes	Cell communication
13	Oxidative phosphorylation	242 (2.75%)	436 (2.2%)	1.721523e-06	3.363591e-05	ko00190	Metabolism	Energy metabolism
14	Salmonella infection	204 (2.32%)	362 (1.83%)	2.813534e-06	5.104555e-05	ko05132	Human Diseases	Infectious diseases: Bacterial
15	Melanogenesis	124 (1.41%)	208 (1.05%)	6.869217e-06	1.163187e-04	ko04916	Organismal Systems	Endocrine system
16	Parkinson's disease	238 (2.71%)	435 (2.2%)	8.45124e-06	1.341634e-04	ko05012	Human Diseases	Neurodegenerative diseases
17	Salivary secretion	162 (1.84%)	284 (1.44%)	1.143766e-05	1.708921e-04	ko04970	Organismal Systems	Digestive system
18	Gastric acid secretion	187 (2.13%)	338 (1.71%)	3.187806e-05	4.415084e-04	ko04971	Organismal Systems	Digestive system
19	Endocytosis	223 (2.54%)	411 (2.08%)	3.302622e-05	4.415084e-04	ko04144	Cellular Processes	Transport and catabolism
20	Phagosome	181 (2.06%)	328 (1.66%)	5.135719e-05	6.522363e-04	ko04145	Cellular Processes	Transport and catabolism
21	Endocrine and other factor-regulated calcium reabsorption	99 (1.13%)	166 (0.84%)	5.430883e-05	6.568782e-04	ko04961	Organismal Systems	Excretory system
22	Calcium signaling pathway	260 (2.96%)	494 (2.5%)	0.0001260372	1.455157e-03	ko04020	Environmental Information Processing	Signal transduction
23	Vibrio cholerae infection	188 (2.14%)	349 (1.76%)	0.0002191648	2.420342e-03	ko05110	Human Diseases	Infectious diseases: Bacterial
24	Ribosome biogenesis in eukaryotes	141 (1.61%)	256 (1.29%)	0.0003655331	3.868559e-03	ko03008	Genetic Information Processing	Translation
25	Vascular smooth muscle contraction	332 (3.78%)	654 (3.31%)	0.0005381806	5.419711e-03	ko04270	Organismal Systems	Circulatory system
26	African trypanosomiasis	22 (0.25%)	29 (0.15%)	0.0005827658	5.419711e-03	ko05143	Human Diseases	Infectious diseases: Parasitic
27	GnRH signaling pathway	133 (1.51%)	242 (1.22%)	0.0005936804	5.419711e-03	ko04912	Organismal Systems	Endocrine system
28	Aminoacyl-tRNA biosynthesis	73 (0.83%)	123 (0.62%)	0.0005974485	5.419711e-03	ko00970	Genetic Information Processing	Translation
29	Cholinergic synapse	109 (1.24%)	194 (0.98%)	0.0006211402	5.440331e-03	ko04725	Organismal Systems	Nervous system
30	Chemokine signaling pathway	159 (1.81%)	296 (1.5%)	0.0007569009	6.408428e-03	ko04062	Organismal Systems	Immune system
31	Antigen processing and presentation	98 (1.12%)	175 (0.88%)	0.001312383	1.062029e-02	ko04612	Organismal Systems	Immune system
32	RNA transport	238 (2.71%)	463 (2.34%)	0.001337989	1.062029e-02	ko03013	Genetic Information Processing	Translation
33	Herpes simplex infection	160 (1.82%)	303 (1.53%)	0.001917465	1.475867e-02	ko05168	Human Diseases	Infectious diseases: Viral

34	Hedgehog signaling pathway	61 (0.69%)	104 (0.53%)	0.00240174	1.754602e-02	ko04340	Environmental Information Processing	Signal transduction
35	Staphylococcus aureus infection	26 (0.3%)	38 (0.19%)	0.002417758	1.754602e-02	ko05150	Human Diseases	Infectious diseases: Bacterial
36	Transcriptional misregulation in cancer	165 (1.88%)	317 (1.6%)	0.003572673	2.520719e-02	ko05202	Human Diseases	Cancers: Overview
37	Phototransduction	71 (0.81%)	126 (0.64%)	0.004609588	3.085248e-02	ko04744	Organismal Systems	Sensory system
38	Prion diseases	72 (0.82%)	128 (0.65%)	0.004615726	3.085248e-02	ko05020	Human Diseases	Neurodegenerative diseases
39	Pancreatic secretion	133 (1.51%)	255 (1.29%)	0.007529461	4.903803e-02	ko04972	Organismal Systems	Digestive system
40	Morphine addiction	98 (1.12%)	183 (0.93%)	0.007880047	5.003830e-02	ko05032	Human Diseases	Substance dependence
41	Sulfur relay system	32 (0.36%)	53 (0.27%)	0.0140478	8.592148e-02	ko04122	Genetic Information Processing	Folding, sorting and degradation
42	Olfactory transduction	109 (1.24%)	209 (1.06%)	0.01444948	8.592148e-02	ko04740	Organismal Systems	Sensory system
43	Bacterial invasion of epithelial cells	131 (1.49%)	255 (1.29%)	0.01467617	8.592148e-02	ko05100	Human Diseases	Infectious diseases: Bacterial
44	Spliceosome	274 (3.12%)	559 (2.83%)	0.01501525	8.592148e-02	ko03040	Genetic Information Processing	Transcription
45	Long-term depression	71 (0.81%)	131 (0.66%)	0.01522231	8.592148e-02	ko04730	Organismal Systems	Nervous system
46	GABAergic synapse	110 (1.25%)	212 (1.07%)	0.01685084	9.304594e-02	ko04727	Organismal Systems	Nervous system
47	Dopaminergic synapse	148 (1.68%)	292 (1.48%)	0.01764124	9.533777e-02	ko04728	Organismal Systems	Nervous system
48	Legionellosis	73 (0.83%)	136 (0.69%)	0.01846899	9.773174e-02	ko05134	Human Diseases	Infectious diseases: Bacterial
49	Protein digestion and absorption	303 (3.45%)	624 (3.16%)	0.01933188	1.002102e-01	ko04974	Organismal Systems	Digestive system
50	Glutamatergic synapse	112 (1.27%)	218 (1.1%)	0.02253579	1.117004e-01	ko04724	Organismal Systems	Nervous system
51	Toxoplasmosis	100 (1.14%)	193 (0.98%)	0.02283469	1.117004e-01	ko05145	Human Diseases	Infectious diseases: Parasitic
52	Retrograde endocannabinoid signaling	111 (1.26%)	216 (1.09%)	0.02286780	1.117004e-01	ko04723	Organismal Systems	Nervous system
53	Arrhythmogenic right ventricular cardiomyopathy (ARVC)	63 (0.72%)	117 (0.59%)	0.02508922	1.180287e-01	ko05412	Human Diseases	Cardiovascular diseases
54	Type I diabetes mellitus	8 (0.09%)	10 (0.05%)	0.02509272	1.180287e-01	ko04940	Human Diseases	Endocrine and metabolic diseases
55	Neuroactive ligand-receptor interaction	223 (2.54%)	455 (2.3%)	0.02610547	1.205598e-01	ko04080	Environmental Information Processing	Signaling molecules and interaction
56	Influenza A	155 (1.76%)	310 (1.57%)	0.02679343	1.215273e-01	ko05164	Human Diseases	Infectious diseases: Viral
57	Gap junction	109 (1.24%)	214 (1.08%)	0.03186056	1.419751e-01	ko04540	Cellular Processes	Cell communication
58	Shigellosis	90 (1.02%)	176 (0.89%)	0.04266738	1.853152e-01	ko05131	Human Diseases	Infectious diseases: Bacterial
59	ErbB signaling pathway	99 (1.13%)	195 (0.99%)	0.04304565	1.853152e-01	ko04012	Environmental Information Processing	Signal transduction
60	Fc gamma R-mediated phagocytosis	115 (1.31%)	229 (1.16%)	0.04408724	1.866360e-01	ko04666	Organismal Systems	Immune system
61	Synaptic vesicle cycle	110 (1.25%)	219 (1.11%)	0.04774566	1.971348e-01	ko04721	Organismal Systems	Nervous system
62	mRNA surveillance pathway	169 (1.92%)	345 (1.74%)	0.04811953	1.971348e-01	ko03015	Genetic Information Processing	Translation
63	Regulation of actin cytoskeleton	363 (4.13%)	766 (3.87%)	0.04984448	2.002273e-01	ko04810	Cellular Processes	Cell motility
64	Phototransduction - fly	98 (1.12%)	194 (0.98%)	0.05045097	2.002273e-01	ko04745	Organismal Systems	Sensory system
65	Inositol phosphate metabolism	61 (0.69%)	117 (0.59%)	0.05619881	2.196077e-01	ko00562	Metabolism	Carbohydrate metabolism
66	Apoptosis	45 (0.51%)	84 (0.42%)	0.05735088	2.207140e-01	ko04210	Cellular Processes	Cell growth and death
67	Glioma	89 (1.01%)	177 (0.9%)	0.06712598	2.540511e-01	ko05214	Human Diseases	Cancers: Specific types
68	Basal transcription factors	60 (0.68%)	116 (0.59%)	0.06801367	2.540511e-01	ko03022	Genetic Information Processing	Transcription
69	Amyotrophic lateral sclerosis (ALS)	123 (1.4%)	250 (1.26%)	0.07162287	2.636552e-01	ko05014	Human Diseases	Neurodegenerative diseases

70	Renin-angiotensin system	42 (0.48%)	79 (0.4%)	0.07345429	2.665341e-01	ko04614	Organismal Systems	Endocrine system
71	Small cell lung cancer	126 (1.43%)	258 (1.3%)	0.08505847	3.042937e-01	ko05222	Human Diseases	Cancers: Specific types
72	Taste transduction	44 (0.5%)	84 (0.42%)	0.08710842	3.071653e-01	ko04742	Organismal Systems	Sensory system
73	Leukocyte transendothelial migration	117 (1.33%)	239 (1.21%)	0.0882798	3.071653e-01	ko04670	Organismal Systems	Immune system
74	Tuberculosis	175 (1.99%)	365 (1.85%)	0.09462265	3.213730e-01	ko05152	Human Diseases	Infectious diseases: Bacterial
75	Chagas disease (American trypanosomiasis)	50 (0.57%)	97 (0.49%)	0.09489359	3.213730e-01	ko05142	Human Diseases	Infectious diseases: Parasitic
76	Wnt signaling pathway	146 (1.66%)	303 (1.53%)	0.1022364	3.416848e-01	ko04310	Environmental Information Processing	Signal transduction
77	Systemic lupus erythematosus	46 (0.52%)	90 (0.46%)	0.1204795	3.974259e-01	ko05322	Human Diseases	Immune diseases
78	Maturity onset diabetes of the young	34 (0.39%)	65 (0.33%)	0.1239469	4.036220e-01	ko04950	Human Diseases	Endocrine and metabolic diseases
79	Rheumatoid arthritis	56 (0.64%)	112 (0.57%)	0.1366617	4.393933e-01	ko05323	Human Diseases	Immune diseases
80	MAPK signaling pathway	187 (2.13%)	396 (2%)	0.1397795	4.437999e-01	ko04010	Environmental Information Processing	Signal transduction
81	Oocyte meiosis	157 (1.79%)	333 (1.68%)	0.1701126	5.285681e-01	ko04114	Cellular Processes	Cell growth and death
82	Circadian rhythm - fly	10 (0.11%)	17 (0.09%)	0.1706401	5.285681e-01	ko04711	Organismal Systems	Environmental adaptation
83	Leishmaniasis	26 (0.3%)	50 (0.25%)	0.1742012	5.289260e-01	ko05140	Human Diseases	Infectious diseases: Parasitic
84	p53 signaling pathway	43 (0.49%)	86 (0.43%)	0.1749204	5.289260e-01	ko04115	Cellular Processes	Cell growth and death
85	Neurotrophin signaling pathway	136 (1.55%)	289 (1.46%)	0.1977902	5.910437e-01	ko04722	Organismal Systems	Nervous system
86	MAPK signaling pathway - fly	27 (0.31%)	53 (0.27%)	0.2062291	6.090952e-01	ko04013	Environmental Information Processing	Signal transduction
87	Glycosaminoglycan degradation	14 (0.16%)	26 (0.13%)	0.2199982	6.422936e-01	ko00531	Metabolism	Glycan biosynthesis and metabolism
88	Basal cell carcinoma	38 (0.43%)	78 (0.39%)	0.2569182	7.415593e-01	ko05217	Human Diseases	Cancers: Specific types
89	Phosphatidylinositol signaling system	106 (1.21%)	228 (1.15%)	0.2854237	8.068121e-01	ko04070	Environmental Information Processing	Signal transduction
90	Fatty acid biosynthesis	13 (0.15%)	25 (0.13%)	0.2858783	8.068121e-01	ko00061	Metabolism	Lipid metabolism
91	Lysine degradation	104 (1.18%)	224 (1.13%)	0.2940191	8.206687e-01	ko00310	Metabolism	Amino acid metabolism
92	Pathways in cancer	357 (4.06%)	787 (3.98%)	0.3065453	8.463316e-01	ko05200	Human Diseases	Cancers: Overview
93	ABC transporters	66 (0.75%)	141 (0.71%)	0.3122936	8.529309e-01	ko02010	Environmental Information Processing	Membrane transport
94	Collecting duct acid secretion	33 (0.38%)	69 (0.35%)	0.3257073	8.801027e-01	ko04966	Organismal Systems	Excretory system
95	Caffeine metabolism	13 (0.15%)	26 (0.13%)	0.3517585	9.391965e-01	ko00232	Metabolism	Biosynthesis of other secondary metabolites
96	Osteoclast differentiation	54 (0.61%)	116 (0.59%)	0.3549719	9.391965e-01	ko04380	Organismal Systems	Development
97	Pentose phosphate pathway	37 (0.42%)	79 (0.4%)	0.3735302	9.734596e-01	ko00030	Metabolism	Carbohydrate metabolism
98	Proteasome	55 (0.63%)	119 (0.6%)	0.3798541	9.734596e-01	ko03050	Genetic Information Processing	Folding, sorting and degradation
99	Complement and coagulation cascades	31 (0.35%)	66 (0.33%)	0.3832478	9.734596e-01	ko04610	Organismal Systems	Immune system
100	Axon guidance	117 (1.33%)	257 (1.3%)	0.3832518	9.734596e-01	ko04360	Organismal Systems	Development
101	Base excision repair	30 (0.34%)	64 (0.32%)	0.392225	9.793156e-01	ko03410	Genetic Information Processing	Replication and repair
102	Progesterone-mediated oocyte maturation	101 (1.15%)	222 (1.12%)	0.3982541	9.793156e-01	ko04914	Organismal Systems	Endocrine system

103	Measles	79 (0.9%)	173 (0.87%)	0.3989945	9.793156e-01	ko05162	Human Diseases	Infectious diseases: Viral
104	Nucleotide excision repair	47 (0.54%)	102 (0.52%)	0.4048352	9.793156e-01	ko03420	Genetic Information Processing	Replication and repair
105	Glyoxylate and dicarboxylate metabolism	47 (0.54%)	102 (0.52%)	0.4048352	9.793156e-01	ko00630	Metabolism	Carbohydrate metabolism
106	Pyrimidine metabolism	124 (1.41%)	274 (1.39%)	0.4128348	9.892457e-01	ko00240	Metabolism	Nucleotide metabolism
107	Protein processing in endoplasmic reticulum	211 (2.4%)	470 (2.38%)	0.4353992	1.000000e+00	ko04141	Genetic Information Processing	Folding, sorting and degradation
108	Cytokine-cytokine receptor interaction	21 (0.24%)	45 (0.23%)	0.4370963	1.000000e+00	ko04060	Environmental Information Processing	Signaling molecules and interaction
109	Allograft rejection	1 (0.01%)	1 (0.01%)	0.4442253	1.000000e+00	ko05330	Human Diseases	Immune diseases
110	Graft-versus-host disease	1 (0.01%)	1 (0.01%)	0.4442253	1.000000e+00	ko05332	Human Diseases	Immune diseases
111	Selenocompound metabolism	11 (0.13%)	23 (0.12%)	0.4498165	1.000000e+00	ko00450	Metabolism	Metabolism of other amino acids
112	Notch signaling pathway	48 (0.55%)	106 (0.54%)	0.4664055	1.000000e+00	ko04330	Environmental Information Processing	Signal transduction
113	Insulin signaling pathway	189 (2.15%)	423 (2.14%)	0.4759296	1.000000e+00	ko04910	Organismal Systems	Endocrine system
114	Epstein-Barr virus infection	192 (2.19%)	430 (2.17%)	0.4804038	1.000000e+00	ko05169	Human Diseases	Infectious diseases: Viral
115	Serotonergic synapse	120 (1.37%)	269 (1.36%)	0.4989401	1.000000e+00	ko04726	Organismal Systems	Nervous system
116	Adherens junction	147 (1.67%)	330 (1.67%)	0.5034025	1.000000e+00	ko04520	Cellular Processes	Cell communication
117	Glycosaminoglycan biosynthesis - heparan sulfate	19 (0.22%)	42 (0.21%)	0.517168	1.000000e+00	ko00534	Metabolism	Glycan biosynthesis and metabolism
118	Prostate cancer	93 (1.06%)	209 (1.06%)	0.5181261	1.000000e+00	ko05215	Human Diseases	Cancers: Specific types
119	Porphyrin and chlorophyll metabolism	57 (0.65%)	128 (0.65%)	0.5243531	1.000000e+00	ko00860	Metabolism	Metabolism of cofactors and vitamins
120	mTOR signaling pathway	49 (0.56%)	110 (0.56%)	0.5265521	1.000000e+00	ko04150	Environmental Information Processing	Signal transduction
121	Non-small cell lung cancer	59 (0.67%)	133 (0.67%)	0.5392918	1.000000e+00	ko05223	Human Diseases	Cancers: Specific types
122	Polyketide sugar unit biosynthesis	3 (0.03%)	6 (0.03%)	0.5467876	1.000000e+00	ko00523	Metabolism	Metabolism of terpenoids and polyketides
123	Amphetamine addiction	110 (1.25%)	249 (1.26%)	0.5558322	1.000000e+00	ko05031	Human Diseases	Substance dependence
124	Glycolysis / Gluconeogenesis	117 (1.33%)	265 (1.34%)	0.5594456	1.000000e+00	ko00010	Metabolism	Carbohydrate metabolism
125	Ascorbate and aldarate metabolism	70 (0.8%)	159 (0.8%)	0.5708117	1.000000e+00	ko00053	Metabolism	Carbohydrate metabolism
126	Vitamin B6 metabolism	5 (0.06%)	11 (0.06%)	0.5876634	1.000000e+00	ko00750	Metabolism	Metabolism of cofactors and vitamins
127	Pertussis	46 (0.52%)	105 (0.53%)	0.5876843	1.000000e+00	ko05133	Human Diseases	Infectious diseases: Bacterial
128	Glycosphingolipid biosynthesis - ganglio series	8 (0.09%)	18 (0.09%)	0.5892996	1.000000e+00	ko00604	Metabolism	Glycan biosynthesis and metabolism
129	Drug metabolism - other enzymes	104 (1.18%)	237 (1.2%)	0.5917125	1.000000e+00	ko00983	Metabolism	Xenobiotics biodegradation and metabolism
130	Nicotinate and nicotinamide metabolism	21 (0.24%)	48 (0.24%)	0.5924202	1.000000e+00	ko00760	Metabolism	Metabolism of cofactors and vitamins
131	D-Arginine and D-ornithine metabolism	14 (0.16%)	32 (0.16%)	0.59783	1.000000e+00	ko00472	Metabolism	Metabolism of other amino acids
132	Long-term potentiation	114 (1.3%)	261 (1.32%)	0.6195086	1.000000e+00	ko04720	Organismal Systems	Nervous system
133	Homologous recombination	19 (0.22%)	44 (0.22%)	0.6224825	1.000000e+00	ko03440	Genetic Information Processing	Replication and repair
134	Dorso-ventral axis formation	63 (0.72%)	145 (0.73%)	0.624726	1.000000e+00	ko04320	Organismal Systems	Development

135	Vasopressin-regulated water reabsorption	45 (0.51%)	104 (0.53%)	0.6303483	1.000000e+00	ko04962	Organismal Systems	Excretory system
136	NOD-like receptor signaling pathway	28 (0.32%)	65 (0.33%)	0.6327729	1.000000e+00	ko04621	Organismal Systems	Immune system
137	Fc epsilon RI signaling pathway	61 (0.69%)	141 (0.71%)	0.6407186	1.000000e+00	ko04664	Organismal Systems	Immune system
138	HTLV-I infection	201 (2.29%)	460 (2.33%)	0.6418567	1.000000e+00	ko05166	Human Diseases	Infectious diseases: Viral
139	Arginine and proline metabolism	101 (1.15%)	233 (1.18%)	0.6540727	1.000000e+00	ko00330	Metabolism	Amino acid metabolism
140	RNA polymerase	59 (0.67%)	137 (0.69%)	0.6569479	1.000000e+00	ko03020	Genetic Information Processing	Transcription
141	Glycosphingolipid biosynthesis - globo series	8 (0.09%)	19 (0.1%)	0.6647507	1.000000e+00	ko00603	Metabolism	Glycan biosynthesis and metabolism
142	Circadian rhythm - mammal	19 (0.22%)	45 (0.23%)	0.6708903	1.000000e+00	ko04710	Organismal Systems	Environmental adaptation
143	Melanoma	34 (0.39%)	80 (0.4%)	0.6757388	1.000000e+00	ko05218	Human Diseases	Cancers: Specific types
144	Asthma	1 (0.01%)	2 (0.01%)	0.691127	1.000000e+00	ko05310	Human Diseases	Immune diseases
145	Nicotine addiction	48 (0.55%)	113 (0.57%)	0.6947262	1.000000e+00	ko05033	Human Diseases	Substance dependence
146	Glycosaminoglycan biosynthesis—chondroitin sulfate	12 (0.14%)	29 (0.15%)	0.6952176	1.000000e+00	ko00532	Metabolism	Glycan biosynthesis and metabolism
147	Malaria	17 (0.19%)	41 (0.21%)	0.703337	1.000000e+00	ko05144	Human Diseases	Infectious diseases: Parasitic
148	Valine, leucine and isoleucine biosynthesis	14 (0.16%)	34 (0.17%)	0.708308	1.000000e+00	ko00290	Metabolism	Amino acid metabolism
149	Cell cycle	124 (1.41%)	289 (1.46%)	0.7192413	1.000000e+00	ko04110	Cellular Processes	Cell growth and death
150	Type II diabetes mellitus	36 (0.41%)	86 (0.43%)	0.720706	1.000000e+00	ko04930	Human Diseases	Endocrine and metabolic diseases
151	Hepatitis C	57 (0.65%)	135 (0.68%)	0.7260495	1.000000e+00	ko05160	Human Diseases	Infectious diseases: Viral
152	DNA replication	35 (0.4%)	84 (0.42%)	0.7312281	1.000000e+00	ko03030	Genetic Information Processing	Replication and repair
153	Folate biosynthesis	18 (0.2%)	44 (0.22%)	0.7314347	1.000000e+00	ko00790	Metabolism	Metabolism of cofactors and vitamins
154	Taurine and hypotaurine metabolism	7 (0.08%)	18 (0.09%)	0.759129	1.000000e+00	ko00430	Metabolism	Metabolism of other amino acids
155	Pantothenate and CoA biosynthesis	16 (0.18%)	40 (0.2%)	0.7639636	1.000000e+00	ko00770	Metabolism	Metabolism of cofactors and vitamins
156	Cocaine addiction	46 (0.52%)	112 (0.57%)	0.7909484	1.000000e+00	ko05030	Human Diseases	Substance dependence
157	RNA degradation	68 (0.77%)	164 (0.83%)	0.800609	1.000000e+00	ko03018	Genetic Information Processing	Folding, sorting and degradation
158	Other types of O-glycan biosynthesis	48 (0.55%)	118 (0.6%)	0.8194658	1.000000e+00	ko00514	Metabolism	Glycan biosynthesis and metabolism
159	Natural killer cell mediated cytotoxicity	52 (0.59%)	128 (0.65%)	0.8305636	1.000000e+00	ko04650	Organismal Systems	Immune system
160	Pyruvate metabolism	92 (1.05%)	222 (1.12%)	0.8331586	1.000000e+00	ko00620	Metabolism	Carbohydrate metabolism
161	Endometrial cancer	57 (0.65%)	140 (0.71%)	0.8343074	1.000000e+00	ko05213	Human Diseases	Cancers: Specific types
162	Hematopoietic cell lineage	38 (0.43%)	95 (0.48%)	0.8346877	1.000000e+00	ko04640	Organismal Systems	Immune system
163	Fat digestion and absorption	61 (0.69%)	150 (0.76%)	0.8442	1.000000e+00	ko04975	Organismal Systems	Digestive system
164	Pancreatic cancer	39 (0.44%)	98 (0.5%)	0.8476057	1.000000e+00	ko05212	Human Diseases	Cancers: Specific types
165	Propanoate metabolism	62 (0.71%)	153 (0.77%)	0.8546639	1.000000e+00	ko00640	Metabolism	Carbohydrate metabolism
166	Carbohydrate digestion and absorption	51 (0.58%)	127 (0.64%)	0.8555495	1.000000e+00	ko04973	Organismal Systems	Digestive system
167	Insect hormone biosynthesis	4 (0.05%)	12 (0.06%)	0.8566835	1.000000e+00	ko00981	Metabolism	Metabolism of terpenoids and polyketides
168	PPAR signaling pathway	89 (1.01%)	217 (1.1%)	0.861123	1.000000e+00	ko03320	Organismal Systems	Endocrine system
169	Riboflavin metabolism	25 (0.28%)	65 (0.33%)	0.8632479	1.000000e+00	ko00740	Metabolism	Metabolism of cofactors and vitamins

170	Cytosolic DNA-sensing pathway	42 (0.48%)	106 (0.54%)	0.8634934	1.000000e+00	ko04623	Organismal Systems	Immune system
171	Mineral absorption	71 (0.81%)	175 (0.88%)	0.8658602	1.000000e+00	ko04978	Organismal Systems	Digestive system
172	Chronic myeloid leukemia	52 (0.59%)	130 (0.66%)	0.8659895	1.000000e+00	ko05220	Human Diseases	Cancers: Specific types
173	Biosynthesis of unsaturated fatty acids	34 (0.39%)	87 (0.44%)	0.8674404	1.000000e+00	ko01040	Metabolism	Lipid metabolism
174	TGF-beta signaling pathway	51 (0.58%)	128 (0.65%)	0.8720987	1.000000e+00	ko04350	Environmental Information Processing	Signal transduction
175	Primary bile acid biosynthesis	17 (0.19%)	46 (0.23%)	0.8793335	1.000000e+00	ko00120	Metabolism	Lipid metabolism
176	T cell receptor signaling pathway	70 (0.8%)	174 (0.88%)	0.8841625	1.000000e+00	ko04660	Organismal Systems	Immune system
177	Ubiquitin mediated proteolysis	129 (1.47%)	313 (1.58%)	0.8868602	1.000000e+00	ko04120	Genetic Information Processing	Folding, sorting and degradation
178	Thyroid cancer	31 (0.35%)	81 (0.41%)	0.890885	1.000000e+00	ko05216	Human Diseases	Cancers: Specific types
179	Epithelial cell signaling in Helicobacter pylori infection	60 (0.68%)	151 (0.76%)	0.8939781	1.000000e+00	ko05120	Human Diseases	Infectious diseases: Bacterial
180	Protein export	26 (0.3%)	69 (0.35%)	0.8950366	1.000000e+00	ko03060	Genetic Information Processing	Folding, sorting and degradation
181	Aldosterone-regulated sodium reabsorption	65 (0.74%)	163 (0.82%)	0.8950612	1.000000e+00	ko04960	Organismal Systems	Excretory system
182	beta-Alanine metabolism	47 (0.54%)	120 (0.61%)	0.895603	1.000000e+00	ko00410	Metabolism	Metabolism of other amino acids
183	Glutathione metabolism	70 (0.8%)	175 (0.88%)	0.896364	1.000000e+00	ko00480	Metabolism	Metabolism of other amino acids
184	VEGF signaling pathway	72 (0.82%)	180 (0.91%)	0.8992129	1.000000e+00	ko04370	Environmental Information Processing	Signal transduction
185	Toll-like receptor signaling pathway	39 (0.44%)	101 (0.51%)	0.8999602	1.000000e+00	ko04620	Organismal Systems	Immune system
186	Mucin type O-Glycan biosynthesis	15 (0.17%)	42 (0.21%)	0.9028875	1.000000e+00	ko00512	Metabolism	Glycan biosynthesis and metabolism
187	Non-homologous end-joining	6 (0.07%)	19 (0.1%)	0.9148106	1.000000e+00	ko03450	Genetic Information Processing	Replication and repair
188	Bladder cancer	19 (0.22%)	53 (0.27%)	0.919752	1.000000e+00	ko05219	Human Diseases	Cancers: Specific types
189	Terpenoid backbone biosynthesis	15 (0.17%)	43 (0.22%)	0.9225685	1.000000e+00	ko00900	Metabolism	Metabolism of terpenoids and polyketides
190	Sulfur metabolism	11 (0.13%)	33 (0.17%)	0.9292739	1.000000e+00	ko00920	Metabolism	Energy metabolism
191	Fatty acid elongation	31 (0.35%)	84 (0.42%)	0.9340648	1.000000e+00	ko00062	Metabolism	Lipid metabolism
192	N-Glycan biosynthesis	39 (0.44%)	104 (0.53%)	0.9369967	1.000000e+00	ko00510	Metabolism	Glycan biosynthesis and metabolism
193	Butanoate metabolism	39 (0.44%)	104 (0.53%)	0.9369967	1.000000e+00	ko00650	Metabolism	Carbohydrate metabolism
194	Tryptophan metabolism	91 (1.04%)	230 (1.16%)	0.9408917	1.000000e+00	ko00380	Metabolism	Amino acid metabolism
195	Fanconi anemia pathway	29 (0.33%)	80 (0.4%)	0.9447464	1.000000e+00	ko03460	Genetic Information Processing	Replication and repair
196	Histidine metabolism	33 (0.38%)	90 (0.46%)	0.9450861	1.000000e+00	ko00340	Metabolism	Amino acid metabolism
197	Thiamine metabolism	1 (0.01%)	5 (0.03%)	0.9469948	1.000000e+00	ko00730	Metabolism	Metabolism of cofactors and vitamins
198	NF-kappa B signaling pathway	43 (0.49%)	115 (0.58%)	0.9477767	1.000000e+00	ko04064	Environmental Information Processing	Signal transduction
199	RIG-I-like receptor signaling pathway	17 (0.19%)	50 (0.25%)	0.9496323	1.000000e+00	ko04622	Organismal Systems	Immune system
200	Cyanoamino acid metabolism	15 (0.17%)	45 (0.23%)	0.9519911	1.000000e+00	ko00460	Metabolism	Metabolism of other amino acids
201	Acute myeloid leukemia	33 (0.38%)	91 (0.46%)	0.9540838	1.000000e+00	ko05221	Human Diseases	Cancers: Specific types
202	Alanine, aspartate and glutamate metabolism	37 (0.42%)	101 (0.51%)	0.9544182	1.000000e+00	ko00250	Metabolism	Amino acid metabolism
203	Regulation of autophagy	13 (0.15%)	40 (0.2%)	0.9550858	1.000000e+00	ko04140	Cellular Processes	Transport and catabolism

204	Ubiquinone and other terpenoid-quinone biosynthesis	21 (0.24%)	61 (0.31%)	0.9569864	1.000000e+00	ko00130	Metabolism	Metabolism of cofactors and vitamins
205	Drug metabolism - cytochrome P450	118 (1.34%)	298 (1.51%)	0.9602252	1.000000e+00	ko00982	Metabolism	Xenobiotics biodegradation and metabolism
206	Valine, leucine and isoleucine degradation	81 (0.92%)	210 (1.06%)	0.963483	1.000000e+00	ko00280	Metabolism	Amino acid metabolism
207	Steroid hormone biosynthesis	56 (0.64%)	150 (0.76%)	0.9675774	1.000000e+00	ko00140	Metabolism	Lipid metabolism
208	D-Glutamine and D-glutamate metabolism	5 (0.06%)	19 (0.1%)	0.968733	1.000000e+00	ko00471	Metabolism	Metabolism of other amino acids
209	Autoimmune thyroid disease	5 (0.06%)	19 (0.1%)	0.968733	1.000000e+00	ko05320	Human Diseases	Immune diseases
210	Synthesis and degradation of ketone bodies	4 (0.05%)	16 (0.08%)	0.9688595	1.000000e+00	ko00072	Metabolism	Lipid metabolism
211	Glycosylphosphatidylinositol(GPI)-anchor biosynthesis	15 (0.17%)	47 (0.24%)	0.9711501	1.000000e+00	ko00563	Metabolism	Glycan biosynthesis and metabolism
212	Glycine, serine and threonine metabolism	41 (0.47%)	114 (0.58%)	0.9732326	1.000000e+00	ko00260	Metabolism	Amino acid metabolism
213	Citrate cycle (TCA cycle)	67 (0.76%)	179 (0.91%)	0.9760162	1.000000e+00	ko00020	Metabolism	Carbohydrate metabolism
214	Alcoholism	133 (1.51%)	339 (1.71%)	0.9773698	1.000000e+00	ko05034	Human Diseases	Substance dependence
215	Phenylalanine, tyrosine and tryptophan biosynthesis	8 (0.09%)	29 (0.15%)	0.980081	1.000000e+00	ko00400	Metabolism	Amino acid metabolism
216	Steroid biosynthesis	13 (0.15%)	43 (0.22%)	0.980315	1.000000e+00	ko00100	Metabolism	Lipid metabolism
217	Tyrosine metabolism	65 (0.74%)	177 (0.9%)	0.9846428	1.000000e+00	ko00350	Metabolism	Amino acid metabolism
218	Metabolism of xenobiotics by cytochrome P450	119 (1.35%)	310 (1.57%)	0.986917	1.000000e+00	ko00980	Metabolism	Xenobiotics biodegradation and metabolism
219	B cell receptor signaling pathway	56 (0.64%)	156 (0.79%)	0.9877532	1.000000e+00	ko04662	Organismal Systems	Immune system
220	Renal cell carcinoma	66 (0.75%)	181 (0.92%)	0.9879464	1.000000e+00	ko05211	Human Diseases	Cancers: Specific types
221	One carbon pool by folate	9 (0.1%)	34 (0.17%)	0.9902732	1.000000e+00	ko00670	Metabolism	Metabolism of cofactors and vitamins
222	Purine metabolism	202 (2.3%)	514 (2.6%)	0.992304	1.000000e+00	ko00230	Metabolism	Nucleotide metabolism
223	Fatty acid metabolism	114 (1.3%)	302 (1.53%)	0.992328	1.000000e+00	ko00071	Metabolism	Lipid metabolism
224	Pentose and glucuronate interconversions	86 (0.98%)	235 (1.19%)	0.9940098	1.000000e+00	ko00040	Metabolism	Carbohydrate metabolism
225	Cell adhesion molecules (CAMs)	51 (0.58%)	148 (0.75%)	0.9947133	1.000000e+00	ko04514	Environmental Information Processing	Signaling molecules and interaction
226	Proximal tubule bicarbonate reclamation	41 (0.47%)	123 (0.62%)	0.995388	1.000000e+00	ko04964	Organismal Systems	Excretory system
227	Glycosaminoglycan biosynthesis - keratan sulfate	5 (0.06%)	24 (0.12%)	0.995819	1.000000e+00	ko00533	Metabolism	Glycan biosynthesis and metabolism
228	Galactose metabolism	27 (0.31%)	87 (0.44%)	0.9961774	1.000000e+00	ko00052	Metabolism	Carbohydrate metabolism
229	SNARE interactions in vesicular transport	12 (0.14%)	46 (0.23%)	0.9967637	1.000000e+00	ko04130	Genetic Information Processing	Folding, sorting and degradation
230	Starch and sucrose metabolism	78 (0.89%)	219 (1.11%)	0.996819	1.000000e+00	ko00500	Metabolism	Carbohydrate metabolism
231	Retinol metabolism	114 (1.3%)	311 (1.57%)	0.9978565	1.000000e+00	ko00830	Metabolism	Metabolism of cofactors and vitamins
232	Lysosome	301 (3.43%)	764 (3.86%)	0.9981312	1.000000e+00	ko04142	Cellular Processes	Transport and catabolism

233	Fructose and mannose metabolism	53 (0.6%)	159 (0.8%)	0.9983592	1.000000e+00	ko00051	Metabolism	Carbohydrate metabolism
234	Amino sugar and nucleotide sugar metabolism	52 (0.59%)	157 (0.79%)	0.9985484	1.000000e+00	ko00520	Metabolism	Carbohydrate metabolism
235	Mismatch repair	18 (0.2%)	66 (0.33%)	0.9986553	1.000000e+00	ko03430	Genetic Information Processing	Replication and repair
236	Sphingolipid metabolism	31 (0.35%)	103 (0.52%)	0.9989863	1.000000e+00	ko00600	Metabolism	Lipid metabolism
237	Glycosphingolipid biosynthesis - lacto and neolacto series	15 (0.17%)	59 (0.3%)	0.9992063	1.000000e+00	ko00601	Metabolism	Glycan biosynthesis and metabolism
238	Cysteine and methionine metabolism	44 (0.5%)	140 (0.71%)	0.9994003	1.000000e+00	ko00270	Metabolism	Amino acid metabolism
239	Linoleic acid metabolism	38 (0.43%)	124 (0.63%)	0.9994042	1.000000e+00	ko00591	Metabolism	Lipid metabolism
240	Colorectal cancer	50 (0.57%)	156 (0.79%)	0.9994192	1.000000e+00	ko05210	Human Diseases	Cancers: Specific types
241	Primary immunodeficiency	1 (0.01%)	13 (0.07%)	0.9995188	1.000000e+00	ko05340	Human Diseases	Immune diseases
242	Jak-STAT signaling pathway	31 (0.35%)	107 (0.54%)	0.9996493	1.000000e+00	ko04630	Environmental Information Processing	Signal transduction
243	Phenylalanine metabolism	41 (0.47%)	136 (0.69%)	0.9997756	1.000000e+00	ko00360	Metabolism	Amino acid metabolism
244	Ether lipid metabolism	29 (0.33%)	109 (0.55%)	0.9999606	1.000000e+00	ko00565	Metabolism	Lipid metabolism
245	Arachidonic acid metabolism	67 (0.76%)	213 (1.08%)	0.9999623	1.000000e+00	ko00590	Metabolism	Lipid metabolism
246	alpha-Linolenic acid metabolism	32 (0.36%)	121 (0.61%)	0.999986	1.000000e+00	ko00592	Metabolism	Lipid metabolism
247	Vitamin digestion and absorption	21 (0.24%)	94 (0.48%)	0.9999977	1.000000e+00	ko04977	Organismal Systems	Digestive system
248	Other glycan degradation	17 (0.19%)	82 (0.41%)	0.9999981	1.000000e+00	ko00511	Metabolism	Glycan biosynthesis and metabolism
249	Glycerolipid metabolism	69 (0.79%)	235 (1.19%)	0.9999993	1.000000e+00	ko00561	Metabolism	Lipid metabolism
250	Bile secretion	148 (1.68%)	447 (2.26%)	0.9999997	1.000000e+00	ko04976	Organismal Systems	Digestive system
251	Adipocytokine signaling pathway	52 (0.59%)	191 (0.97%)	0.9999997	1.000000e+00	ko04920	Organismal Systems	Endocrine system
252	Peroxisome	119 (1.35%)	373 (1.89%)	0.9999998	1.000000e+00	ko04146	Cellular Processes	Transport and catabolism
253	Metabolic pathways	1361 (15.49%)	3423 (17.31%)	1	1.000000e+00	ko01100	Metabolism	Global map
254	Glycerophospholipid metabolism	79 (0.9%)	305 (1.54%)	1	1.000000e+00	ko00564	Metabolism	Lipid metabolism

#	Pathway	Differentially expressed genes
1	Ribosome	CL1053.Contig1_All, CL1053.Contig2_All, CL1074.Contig1_All, CL110.Contig11_All, CL110.Contig1_All, CL110.Contig3_All, CL110.Contig6_All, CL110.Contig7_All, CL110.Contig8_All, CL1244.Contig1_All, CL1321.Contig1_All, CL1321.Contig2_All, CL1351.Contig2_All, CL1523.Contig3_All, CL1523.Contig4_All, CL1642.Contig2_All, CL1703.Contig2_All, CL1703.Contig3_All, CL1703.Contig4_All, CL1828.Contig5_All, CL1928.Contig1_All, CL2081.Contig1_All, CL2221.Contig1_All, CL2283.Contig1_All, CL2283.Contig2_All, CL2320.Contig1_All, CL2320.Contig2_All, CL2434.Contig1_All, CL2676.Contig1_All, CL2806.Contig1_All, CL2961.Contig1_All, CL2961.Contig3_All, CL3021.Contig1_All, CL3021.Contig2_All, CL3036.Contig1_All, CL3093.Contig1_All, CL3189.Contig2_All, CL323.Contig1_All, CL3257.Contig1_All, CL3395.Contig1_All, CL3395.Contig2_All, CL343.Contig2_All, CL3447.Contig1_All, CL3733.Contig2_All, CL3733.Contig3_All, CL3762.Contig2_All, CL3762.Contig3_All, CL3894.Contig2_All, CL4125.Contig1_All, CL4130.Contig2_All, CL4135.Contig1_All, CL4170.Contig1_All, CL4170.Contig2_All, CL4286.Contig1_All, CL4425.Contig3_All, CL4529.Contig2_All, CL4581.Contig1_All, CL4581.Contig2_All, CL4681.Contig2_All, CL477.Contig1_All, CL4792.Contig2_All, CL4990.Contig2_All, CL5145.Contig2_All, CL5223.Contig1_All, CL5223.Contig2_All, CL5347.Contig1_All, CL5448.Contig1_All, CL616.Contig1_All, CL651.Contig1_All, CL661.Contig2_All, CL85.Contig1_All, CL993.Contig1_All, Unigene10115_All, Unigene10132_All, Unigene10215_All, Unigene10387_All, Unigene10416_All, Unigene10676_All, Unigene10677_All, Unigene10681_All, Unigene10682_All, Unigene10683_All, Unigene10690_All, Unigene10697_All, Unigene10772_All, Unigene10783_All, Unigene10789_All, Unigene10794_All, Unigene10795_All, Unigene10796_All, Unigene10797_All, Unigene10798_All, Unigene10799_All, Unigene10800_All, Unigene10806_All, Unigene10814_All, Unigene10815_All, Unigene10827_All, Unigene10828_All, Unigene10895_All, Unigene10896_All, Unigene10990_All, Unigene11015_All, Unigene11070_All, Unigene11089_All, Unigene11142_All, Unigene11143_All, Unigene11144_All, Unigene11154_All, Unigene11155_All, Unigene11291_All, Unigene11292_All, Unigene11383_All, Unigene11398_All, Unigene11399_All, Unigene11400_All, Unigene11487_All, Unigene11501_All, Unigene11618_All, Unigene11619_All, Unigene11621_All, Unigene11645_All, Unigene11646_All, Unigene11669_All, Unigene11691_All, Unigene11702_All, Unigene11703_All, Unigene11704_All, Unigene11705_All, Unigene11706_All, Unigene11707_All, Unigene11708_All, Unigene11709_All, Unigene11710_All, Unigene11712_All, Unigene11713_All, Unigene11714_All, Unigene11715_All, Unigene11716_All, Unigene11717_All, Unigene11718_All, Unigene11719_All, Unigene11722_All, Unigene11723_All, Unigene11725_All, Unigene11726_All, Unigene11727_All, Unigene11728_All, Unigene11731_All, Unigene11733_All, Unigene11734_All, Unigene11735_All, Unigene11737_All, Unigene11738_All, Unigene11739_All, Unigene11740_All, Unigene11742_All, Unigene11743_All, Unigene11745_All, Unigene11746_All, Unigene11747_All, Unigene11748_All, Unigene11749_All, Unigene11750_All, Unigene11751_All, Unigene11752_All, Unigene11753_All, Unigene11754_All, Unigene11755_All, Unigene11757_All, Unigene11758_All, Unigene11760_All, Unigene11761_All, Unigene11762_All, Unigene11764_All, Unigene11766_All, Unigene11771_All, Unigene11776_All, Unigene11825_All, Unigene11835_All, Unigene12000_All, Unigene12119_All, Unigene12222_All, Unigene12260_All, Unigene12282_All, Unigene12306_All, Unigene12324_All, Unigene12333_All, Unigene12431_All, Unigene1244_All, Unigene12489_All, Unigene12504_All, Unigene12506_All, Unigene12514_All, Unigene12521_All, Unigene12531_All, Unigene12566_All, Unigene12626_All, Unigene12649_All, Unigene12653_All, Unigene12690_All, Unigene12726_All, Unigene12883_All, Unigene12908_All, Unigene12970_All, Unigene13090_All, Unigene13123_All, Unigene13133_All, Unigene13149_All, Unigene13164_All, Unigene13173_All, Unigene13213_All, Unigene13214_All, Unigene13489_All, Unigene13585_All, Unigene13620_All, Unigene1422_All, Unigene15131_All, Unigene15422_All, Unigene15508_All, Unigene15522_All, Unigene15523_All, Unigene15534_All, Unigene15569_All, Unigene15572_All, Unigene16094_All, Unigene16754_All, Unigene16756_All, Unigene16757_All, Unigene16759_All, Unigene16760_All, Unigene16762_All, Unigene16765_All, Unigene16792_All, Unigene16793_All, Unigene16858_All, Unigene16898_All, Unigene16969_All, Unigene17012_All, Unigene17039_All, Unigene1703_All, Unigene17225_All, Unigene17429_All, Unigene17455_All, Unigene17562_All, Unigene17563_All, Unigene17573_All, Unigene17599_All, Unigene17646_All, Unigene17665_All, Unigene17680_All, Unigene17711_All, Unigene17716_All, Unigene17719_All, Unigene17757_All, Unigene17758_All, Unigene17759_All, Unigene17761_All, Unigene17762_All, Unigene17767_All, Unigene17774_All, Unigene17779_All, Unigene17780_All, Unigene17782_All, Unigene17784_All, Unigene17789_All, Unigene17791_All, Unigene17996_All, Unigene18011_All, Unigene18020_All, Unigene18048_All, Unigene18049_All, Unigene18138_All, Unigene18150_All, Unigene18164_All, Unigene18198_All, Unigene18206_All, Unigene18207_All, Unigene18223_All, Unigene18242_All, Unigene18247_All, Unigene18269_All, Unigene18289_All, Unigene18302_All, Unigene18313_All, Unigene18327_All, Unigene18353_All, Unigene18383_All, Unigene18403_All, Unigene18476_All, Unigene18491_All, Unigene18498_All, Unigene18542_All, Unigene19006_All, Unigene19016_All, Unigene19017_All, Unigene19020_All, Unigene19023_All, Unigene19025_All, Unigene19029_All, Unigene19050_All, Unigene19271_All, Unigene19905_All, Unigene201_All, Unigene20636_All, Unigene20677_All, Unigene21409_All, Unigene2223_All, Unigene2227_All, Unigene2303_All, Unigene2561_All, Unigene2585_All, Unigene3339_All, Unigene3490_All, Unigene3612_All, Unigene3639_All, Unigene3649_All, Unigene444_All, Unigene4483_All, Unigene4605_All, Unigene5299_All, Unigene538_All, Unigene5524_All, Unigene6158_All, Unigene6179_All, Unigene6189_All, Unigene6257_All, Unigene629_All, Unigene6335_All, Unigene6421_All, Unigene6491_All, Unigene6520_All, Unigene6555_All, Unigene6593_All, Unigene6600_All, Unigene6607_All, Unigene6996_All, Unigene7366_All, Unigene7486_All, Unigene7571_All, Unigene7621_All, Unigene7624_All, Unigene8165_All, Unigene8219_All, Unigene8260_All, Unigene8363_All, Unigene8416_All, Unigene8543_All, Unigene8603_All, Unigene9243_All, Unigene9392_All, Unigene9397_All, Unigene9432_All, Unigene9484_All, Unigene9515_All, Unigene9574_All, Unigene9590_All, Unigene9689_All

2	Amoebiasis	CL1011.Contig1_All, CL1011.Contig4_All, CL1055.Contig1_All, CL1055.Contig3_All, CL1055.Contig4_All, CL1055.Contig5_All, CL1055.Contig6_All, CL1055.Contig7_All, CL1062.Contig1_All, CL1066.Contig1_All, CL1066.Contig2_All, CL1066.Contig3_All, CL1079.Contig4_All, CL1116.Contig2_All, CL1147.Contig1_All, CL1147.Contig2_All, CL1202.Contig1_All, CL1202.Contig2_All, CL1370.Contig3_All, CL1436.Contig2_All, CL1452.Contig1_All, CL1495.Contig1_All, CL1495.Contig2_All, CL1503.Contig3_All, CL1503.Contig4_All, CL1503.Contig5_All, CL1566.Contig2_All, CL1613.Contig1_All, CL1718.Contig1_All, CL1718.Contig2_All, CL1868.Contig1_All, CL1868.Contig2_All, CL1868.Contig4_All, CL1890.Contig4_All, CL2009.Contig2_All, CL2066.Contig1_All, CL209.Contig1_All, CL209.Contig2_All, CL209.Contig3_All, CL209.Contig4_All, CL2099.Contig1_All, CL2105.Contig1_All, CL2182.Contig1_All, CL2182.Contig3_All, CL2182.Contig4_All, CL2252.Contig1_All, CL2252.Contig2_All, CL2252.Contig3_All, CL2293.Contig1_All, CL2297.Contig2_All, CL2328.Contig1_All, CL2424.Contig3_All, CL2478.Contig2_All, CL2481.Contig1_All, CL2496.Contig1_All, CL2611.Contig1_All, CL2611.Contig2_All, CL2698.Contig2_All, CL2757.Contig2_All, CL2927.Contig1_All, CL2927.Contig3_All, CL2930.Contig5_All, CL2952.Contig1_All, CL2979.Contig1_All, CL3022.Contig2_All, CL3145.Contig2_All, CL3243.Contig1_All, CL3243.Contig2_All, CL3244.Contig1_All, CL3248.Contig2_All, CL326.Contig1_All, CL3290.Contig2_All, CL3290.Contig3_All, CL3327.Contig1_All, CL3327.Contig2_All, CL3371.Contig1_All, CL3387.Contig1_All, CL3387.Contig2_All, CL3442.Contig1_All, CL3487.Contig6_All, CL367.Contig1_All, CL367.Contig2_All, CL367.Contig3_All, CL3690.Contig1_All, CL3797.Contig1_All, CL3797.Contig2_All, CL387.Contig2_All, CL39.Contig1_All, CL39.Contig2_All, CL39.Contig3_All, CL39.Contig4_All, CL4005.Contig2_All, CL4005.Contig3_All, CL4026.Contig1_All, CL4042.Contig1_All, CL4042.Contig2_All, CL4042.Contig3_All, CL4193.Contig1_All, CL4399.Contig3_All, CL4401.Contig2_All, CL4401.Contig3_All, CL4401.Contig4_All, CL4491.Contig1_All, CL4511.Contig1_All, CL470.Contig1_All, CL4844.Contig1_All, CL4844.Contig2_All, CL4879.Contig1_All, CL4891.Contig1_All, CL4891.Contig2_All, CL4992.Contig1_All, CL5048.Contig1_All, CL5221.Contig1_All, CL5268.Contig2_All, CL5268.Contig3_All, CL5280.Contig1_All, CL5373.Contig1_All, CL5373.Contig2_All, CL5391.Contig1_All, CL5391.Contig2_All, CL5399.Contig1_All, CL5399.Contig2_All, CL5464.Contig1_All, CL5464.Contig2_All, CL5464.Contig3_All, CL598.Contig1_All, CL598.Contig2_All, CL626.Contig1_All, CL626.Contig2_All, CL626.Contig3_All, CL626.Contig4_All, CL626.Contig6_All, CL695.Contig16_All, CL748.Contig1_All, CL748.Contig3_All, CL80.Contig1_All, CL847.Contig1_All, CL940.Contig3_All, CL940.Contig5_All, Unigene10012_All, Unigene10058_All, Unigene10093_All, Unigene10095_All, Unigene10096_All, Unigene10123_All, Unigene10200_All, Unigene10238_All, Unigene1024_All, Unigene10254_All, Unigene1026_All, Unigene10316_All, Unigene10469_All, Unigene10623_All, Unigene10723_All, Unigene10724_All, Unigene10779_All, Unigene10787_All, Unigene1096_All, Unigene10974_All, Unigene10983_All, Unigene10984_All, Unigene11093_All, Unigene11094_All, Unigene11097_All, Unigene11141_All, Unigene11160_All, Unigene11190_All, Unigene11203_All, Unigene11222_All, Unigene11337_All, Unigene11338_All, Unigene11358_All, Unigene11377_All, Unigene11436_All, Unigene11447_All, Unigene11448_All, Unigene11469_All, Unigene11510_All, Unigene11511_All, Unigene11515_All, Unigene11538_All, Unigene11543_All, Unigene11544_All, Unigene11557_All, Unigene11565_All, Unigene11576_All, Unigene11578_All, Unigene11587_All, Unigene11588_All, Unigene11589_All, Unigene11590_All, Unigene11629_All, Unigene11648_All, Unigene11660_All, Unigene11662_All, Unigene11672_All, Unigene11687_All, Unigene11797_All, Unigene11810_All, Unigene11880_All, Unigene11985_All, Unigene12016_All, Unigene12017_All, Unigene12118_All, Unigene12125_All, Unigene12154_All, Unigene12155_All, Unigene12194_All, Unigene12210_All, Unigene12212_All, Unigene12285_All, Unigene12315_All, Unigene12357_All, Unigene12435_All, Unigene12533_All, Unigene12537_All, Unigene12692_All, Unigene12710_All, Unigene12793_All, Unigene12853_All, Unigene12974_All, Unigene1322_All, Unigene13324_All, Unigene13732_All, Unigene14133_All, Unigene1488_All, Unigene15320_All, Unigene15658_All, Unigene15814_All, Unigene15815_All, Unigene15824_All, Unigene15840_All, Unigene1614_All, Unigene16558_All, Unigene16580_All, Unigene16659_All, Unigene16965_All, Unigene16966_All, Unigene1704_All, Unigene17111_All, Unigene17321_All, Unigene17327_All, Unigene1757_All, Unigene17596_All, Unigene17679_All, Unigene17727_All, Unigene17728_All, Unigene17731_All, Unigene17798_All, Unigene1792_All, Unigene18063_All, Unigene18488_All, Unigene18496_All, Unigene18873_All, Unigene18898_All, Unigene19237_All, Unigene19303_All, Unigene19360_All, Unigene19426_All, Unigene19840_All, Unigene20095_All, Unigene20128_All, Unigene20912_All, Unigene20_All, Unigene2105_All, Unigene2180_All, Unigene2190_All, Unigene2209_All, Unigene2251_All, Unigene2297_All, Unigene2503_All, Unigene2560_All, Unigene28_All, Unigene3000_All, Unigene3063_All, Unigene3069_All, Unigene3123_All, Unigene3157_All, Unigene3193_All, Unigene3207_All, Unigene3261_All, Unigene3316_All, Unigene3323_All, Unigene3346_All, Unigene3391_All, Unigene3403_All, Unigene3476_All, Unigene3669_All, Unigene3779_All, Unigene3889_All, Unigene3947_All, Unigene3964_All, Unigene4004_All, Unigene4053_All, Unigene4070_All, Unigene4112_All, Unigene414_All, Unigene4218_All, Unigene4305_All, Unigene4359_All, Unigene4418_All, Unigene4444_All, Unigene4532_All, Unigene4825_All, Unigene4922_All, Unigene5059_All, Unigene5086_All, Unigene5121_All, Unigene5169_All, Unigene5233_All, Unigene5265_All, Unigene5589_All, Unigene5799_All, Unigene579_All, Unigene5889_All, Unigene5913_All, Unigene6009_All, Unigene6050_All, Unigene6076_All, Unigene6098_All, Unigene6145_All, Unigene6305_All, Unigene6326_All, Unigene646_All, Unigene6478_All, Unigene65_All, Unigene6741_All, Unigene6780_All, Unigene6886_All, Unigene7054_All, Unigene7106_All, Unigene7280_All, Unigene7431_All, Unigene7440_All, Unigene7534_All, Unigene7560_All, Unigene8102_All, Unigene8151_All, Unigene8179_All, Unigene8204_All, Unigene8231_All, Unigene8262_All, Unigene8324_All, Unigene8399_All, Unigene8422_All, Unigene8514_All, Unigene8537_All, Unigene8562_All, Unigene8936_All, Unigene9040_All, Unigene9042_All, Unigene9161_All, Unigene9271_All, Unigene9284_All, Unigene936_All, Unigene9437_All, Unigene9513_All, Unigene955_All, Unigene9611_All, Unigene9733_All, Unigene981_All, Unigene9988_All

3	Pathogenic Escherichia coli infection	CL107.Contig3_All, CL1252.Contig6_All, CL1355.Contig1_All, CL1498.Contig1_All, CL1539.Contig1_All, CL1539.Contig2_All, CL1539.Contig3_All, CL1539.Contig4_All, CL156.Contig1_All, CL156.Contig3_All, CL156.Contig4_All, CL1723.Contig1_All, CL1723.Contig2_All, CL1723.Contig7_All, CL1803.Contig1_All, CL2049.Contig3_All, CL2085.Contig1_All, CL2182.Contig1_All, CL2182.Contig3_All, CL2182.Contig4_All, CL2224.Contig2_All, CL2224.Contig3_All, CL2224.Contig4_All, CL2375.Contig2_All, CL249.Contig1_All, CL249.Contig3_All, CL249.Contig5_All, CL249.Contig6_All, CL257.Contig1_All, CL257.Contig2_All, CL263.Contig3_All, CL271.Contig2_All, CL2923.Contig2_All, CL2975.Contig2_All, CL2977.Contig1_All, CL2977.Contig2_All, CL2986.Contig1_All, CL3027.Contig3_All, CL3315.Contig3_All, CL3315.Contig4_All, CL3489.Contig1_All, CL3510.Contig3_All, CL3696.Contig1_All, CL3823.Contig1_All, CL3823.Contig2_All, CL3848.Contig2_All, CL39.Contig1_All, CL39.Contig2_All, CL39.Contig3_All, CL39.Contig4_All, CL402.Contig1_All, CL4088.Contig2_All, CL4130.Contig1_All, CL4320.Contig3_All, CL4479.Contig2_All, CL4479.Contig3_All, CL4479.Contig5_All, CL4776.Contig1_All, CL4776.Contig2_All, CL5005.Contig1_All, CL5005.Contig2_All, CL5109.Contig1_All, CL5109.Contig2_All, CL5149.Contig1_All, CL5149.Contig2_All, CL5186.Contig2_All, CL5207.Contig2_All, CL5391.Contig1_All, CL5391.Contig2_All, CL5468.Contig3_All, CL621.Contig2_All, CL621.Contig4_All, CL621.Contig5_All, CL855.Contig1_All, CL855.Contig2_All, CL855.Contig3_All, CL940.Contig3_All, CL940.Contig5_All, Unigene10097_All, Unigene10125_All, Unigene10126_All, Unigene10128_All, Unigene1016_All, Unigene1017_All, Unigene10448_All, Unigene1050_All, Unigene10998_All, Unigene11082_All, Unigene11121_All, Unigene11130_All, Unigene11197_All, Unigene11202_All, Unigene11307_All, Unigene11369_All, Unigene11395_All, Unigene11459_All, Unigene11521_All, Unigene11597_All, Unigene11598_All, Unigene11599_All, Unigene1169_All, Unigene11874_All, Unigene11922_All, Unigene11992_All, Unigene12201_All, Unigene12203_All, Unigene12314_All, Unigene12522_All, Unigene12603_All, Unigene12684_All, Unigene13039_All, Unigene13060_All, Unigene13092_All, Unigene1447_All, Unigene15308_All, Unigene1538_All, Unigene16235_All, Unigene16840_All, Unigene16963_All, Unigene17404_All, Unigene18142_All, Unigene18598_All, Unigene18644_All, Unigene18674_All, Unigene18898_All, Unigene198_All, Unigene19920_All, Unigene20095_All, Unigene2741_All, Unigene2771_All, Unigene278_All, Unigene3176_All, Unigene3337_All, Unigene3457_All, Unigene3482_All, Unigene3945_All, Unigene4081_All, Unigene4207_All, Unigene420_All, Unigene5303_All, Unigene5448_All, Unigene5654_All, Unigene5727_All, Unigene5808_All, Unigene5873_All, Unigene6014_All, Unigene6029_All, Unigene615_All, Unigene649_All, Unigene6526_All, Unigene7001_All, Unigene7063_All, Unigene715_All, Unigene7213_All, Unigene7541_All, Unigene7767_All, Unigene783_All, Unigene7983_All, Unigene8103_All, Unigene8184_All, Unigene8775_All, Unigene9050_All, Unigene9137_All, Unigene9217_All, Unigene9374_All, Unigene9578_All, Unigene9604_All, Unigene9740_All, Unigene9785_All, Unigene9787_All, Unigene9855_All, Unigene9877_All, Unigene9902_All, Unigene9947_All, Unigene9972_All, Unigene9973_All, Unigene9984_All
4	Dilated cardiomyopathy	CL1075.Contig3_All, CL1079.Contig4_All, CL1087.Contig2_All, CL1087.Contig3_All, CL1132.Contig1_All, CL1132.Contig2_All, CL1141.Contig2_All, CL1355.Contig1_All, CL1452.Contig1_All, CL156.Contig1_All, CL156.Contig3_All, CL156.Contig4_All, CL1687.Contig4_All, CL1698.Contig2_All, CL1704.Contig1_All, CL1704.Contig2_All, CL1803.Contig1_All, CL2265.Contig1_All, CL2265.Contig2_All, CL2331.Contig2_All, CL2331.Contig3_All, CL2517.Contig4_All, CL2904.Contig1_All, CL2904.Contig2_All, CL3011.Contig1_All, CL3046.Contig1_All, CL3092.Contig1_All, CL3092.Contig2_All, CL3260.Contig1_All, CL3260.Contig2_All, CL3260.Contig3_All, CL3260.Contig4_All, CL3260.Contig5_All, CL3260.Contig7_All, CL3295.Contig1_All, CL3297.Contig1_All, CL3297.Contig2_All, CL3347.Contig1_All, CL3347.Contig2_All, CL3428.Contig1_All, CL3428.Contig2_All, CL3442.Contig1_All, CL349.Contig12_All, CL349.Contig3_All, CL349.Contig5_All, CL3543.Contig2_All, CL3543.Contig3_All, CL3543.Contig4_All, CL3554.Contig1_All, CL3590.Contig2_All, CL3672.Contig4_All, CL3761.Contig1_All, CL4005.Contig2_All, CL4005.Contig3_All, CL4187.Contig1_All, CL4250.Contig2_All, CL4262.Contig2_All, CL4332.Contig1_All, CL4332.Contig2_All, CL440.Contig3_All, CL440.Contig5_All, CL440.Contig6_All, CL4454.Contig3_All, CL4596.Contig1_All, CL470.Contig1_All, CL4992.Contig1_All, CL503.Contig2_All, CL503.Contig3_All, CL503.Contig4_All, CL503.Contig5_All, CL503.Contig6_All, CL503.Contig8_All, CL531.Contig1_All, CL5352.Contig1_All, CL5352.Contig2_All, CL5468.Contig3_All, CL573.Contig1_All, CL636.Contig1_All, CL636.Contig2_All, CL642.Contig1_All, CL642.Contig2_All, CL644.Contig1_All, CL703.Contig2_All, CL703.Contig3_All, CL703.Contig5_All, CL703.Contig6_All, CL923.Contig1_All, CL923.Contig2_All, CL923.Contig3_All, CL940.Contig3_All, CL940.Contig5_All, Unigene10040_All, Unigene10058_All, Unigene10201_All, Unigene10202_All, Unigene10471_All, Unigene11289_All, Unigene11298_All, Unigene11300_All, Unigene1131_All, Unigene11345_All, Unigene11597_All, Unigene11598_All, Unigene11599_All, Unigene11623_All, Unigene11923_All, Unigene11935_All, Unigene12418_All, Unigene12476_All, Unigene1257_All, Unigene12627_All, Unigene12658_All, Unigene12684_All, Unigene12697_All, Unigene12719_All, Unigene12828_All, Unigene13092_All, Unigene136_All, Unigene15271_All, Unigene15824_All, Unigene1633_All, Unigene16435_All, Unigene16510_All, Unigene16716_All, Unigene16874_All, Unigene16976_All, Unigene1705_All, Unigene17116_All, Unigene17117_All, Unigene17279_All, Unigene17362_All, Unigene17398_All, Unigene17697_All, Unigene17718_All, Unigene17773_All, Unigene1777_All, Unigene17824_All, Unigene18076_All, Unigene18106_All, Unigene18173_All, Unigene18215_All, Unigene18378_All, Unigene18598_All, Unigene18645_All, Unigene19289_All, Unigene19364_All, Unigene19453_All, Unigene19647_All, Unigene19738_All, Unigene19745_All, Unigene19866_All, Unigene20065_All, Unigene2020_All, Unigene20299_All, Unigene2029_All, Unigene2032_All, Unigene2052_All, Unigene20530_All, Unigene2053_All, Unigene20625_All, Unigene210_All, Unigene237_All, Unigene2503_All, Unigene2514_All, Unigene2741_All, Unigene278_All, Unigene2980_All, Unigene3061_All, Unigene3187_All, Unigene3300_All, Unigene3328_All, Unigene3407_All, Unigene3659_All, Unigene3696_All, Unigene3779_All, Unigene3945_All, Unigene4052_All, Unigene4112_All, Unigene4161_All, Unigene4207_All, Unigene4269_All, Unigene4406_All, Unigene4532_All, Unigene4692_All, Unigene4920_All, Unigene500_All, Unigene58_All, Unigene5994_All, Unigene6013_All, Unigene6037_All, Unigene6273_All, Unigene6284_All, Unigene6896_All, Unigene6907_All, Unigene7015_All, Unigene7063_All, Unigene7096_All, Unigene7129_All, Unigene7829_All, Unigene7854_All, Unigene785_All, Unigene8009_All, Unigene8482_All, Unigene8621_All, Unigene8830_All, Unigene8959_All, Unigene9039_All, Unigene9062_All, Unigene9076_All, Unigene9200_All, Unigene93_All, Unigene9722_All, Unigene9733_All

5	Focal adhesion	CL1011.Contig1_All, CL1011.Contig4_All, CL1055.Contig1_All, CL1055.Contig3_All, CL1055.Contig4_All, CL1055.Contig5_All, CL1055.Contig6_All, CL1055.Contig7_All, CL1066.Contig1_All, CL1066.Contig2_All, CL1066.Contig3_All, CL1068.Contig5_All, CL1092.Contig1_All, CL1099.Contig3_All, CL1120.Contig2_All, CL1147.Contig1_All, CL1147.Contig2_All, CL119.Contig10_All, CL119.Contig11_All, CL119.Contig3_All, CL119.Contig4_All, CL119.Contig8_All, CL119.Contig9_All, CL1202.Contig1_All, CL1202.Contig2_All, CL1355.Contig1_All, CL1436.Contig2_All, CL147.Contig6_All, CL1473.Contig1_All, CL1495.Contig1_All, CL1495.Contig2_All, CL1503.Contig3_All, CL1503.Contig4_All, CL1503.Contig5_All, CL1535.Contig3_All, CL1548.Contig2_All, CL1548.Contig3_All, CL156.Contig1_All, CL156.Contig3_All, CL156.Contig4_All, CL1563.Contig2_All, CL1566.Contig1_All, CL1566.Contig2_All, CL1718.Contig1_All, CL1718.Contig2_All, CL1795.Contig5_All, CL1803.Contig1_All, CL1807.Contig1_All, CL1807.Contig2_All, CL1843.Contig3_All, CL1874.Contig2_All, CL1890.Contig4_All, CL1954.Contig1_All, CL1954.Contig2_All, CL2009.Contig2_All, CL2039.Contig1_All, CL2061.Contig1_All, CL2066.Contig1_All, CL209.Contig1_All, CL209.Contig2_All, CL209.Contig3_All, CL209.Contig4_All, CL2099.Contig1_All, CL2182.Contig1_All, CL2182.Contig3_All, CL2182.Contig4_All, CL2224.Contig2_All, CL2224.Contig3_All, CL2224.Contig4_All, CL2252.Contig1_All, CL2252.Contig2_All, CL2252.Contig3_All, CL2297.Contig2_All, CL2331.Contig2_All, CL2331.Contig3_All, CL241.Contig1_All, CL241.Contig7_All, CL244.Contig2_All, CL2552.Contig3_All, CL257.Contig1_All, CL257.Contig2_All, CL2611.Contig1_All, CL2611.Contig2_All, CL2658.Contig1_All, CL2658.Contig2_All, CL271.Contig2_All, CL2722.Contig1_All, CL2833.Contig1_All, CL2927.Contig1_All, CL2927.Contig3_All, CL2952.Contig1_All, CL296.Contig16_All, CL3042.Contig1_All, CL3042.Contig3_All, CL305.Contig2_All, CL309.Contig2_All, CL309.Contig3_All, CL309.Contig4_All, CL3116.Contig2_All, CL3327.Contig1_All, CL3327.Contig2_All, CL3371.Contig1_All, CL3387.Contig1_All, CL3387.Contig2_All, CL3500.Contig1_All, CL3525.Contig1_All, CL3525.Contig2_All, CL3622.Contig2_All, CL367.Contig1_All, CL367.Contig2_All, CL367.Contig3_All, CL3689.Contig2_All, CL3797.Contig1_All, CL3797.Contig2_All, CL3823.Contig1_All, CL3823.Contig2_All, CL3879.Contig2_All, CL39.Contig1_All, CL39.Contig2_All, CL39.Contig3_All, CL39.Contig4_All, CL392.Contig1_All, CL392.Contig2_All, CL392.Contig3_All, CL400.Contig1_All, CL400.Contig2_All, CL4002.Contig1_All, CL4026.Contig1_All, CL4042.Contig1_All, CL4042.Contig2_All, CL4042.Contig3_All, CL4203.Contig3_All, CL4216.Contig1_All, CL4216.Contig2_All, CL4381.Contig1_All, CL4401.Contig2_All, CL4401.Contig3_All, CL4401.Contig4_All, CL4402.Contig1_All, CL4439.Contig2_All, CL4511.Contig1_All, CL4559.Contig2_All, CL4559.Contig3_All, CL4637.Contig1_All, CL4654.Contig2_All, CL4690.Contig1_All, CL4690.Contig2_All, CL470.Contig1_All, CL4736.Contig1_All, CL4736.Contig2_All, CL4787.Contig1_All, CL4844.Contig1_All, CL4844.Contig2_All, CL4862.Contig1_All, CL498.Contig2_All, CL498.Contig4_All, CL4992.Contig1_All, CL5048.Contig1_All, CL5061.Contig2_All, CL5115.Contig1_All, CL5162.Contig1_All, CL5162.Contig2_All, CL5212.Contig1_All, CL5212.Contig2_All, CL5221.Contig1_All, CL5222.Contig2_All, CL5239.Contig1_All, CL5246.Contig3_All, CL5268.Contig2_All, CL5268.Contig3_All, CL5330.Contig1_All, CL5330.Contig2_All, CL5361.Contig2_All, CL5361.Contig4_All, CL5373.Contig1_All, CL5373.Contig2_All, CL5391.Contig1_All, CL5391.Contig2_All, CL5464.Contig1_All, CL5464.Contig2_All, CL5464.Contig3_All, CL5468.Contig3_All, CL5469.Contig2_All, CL571.Contig2_All, CL577.Contig1_All, CL577.Contig2_All, CL695.Contig16_All, CL741.Contig2_All, CL748.Contig1_All, CL748.Contig3_All, CL80.Contig1_All, CL903.Contig1_All, CL916.Contig2_All, CL940.Contig3_All, CL940.Contig5_All, CL95.Contig1_All, CL970.Contig2_All, Unigene10012_All, Unigene10093_All, Unigene10095_All, Unigene10096_All, Unigene10123_All, Unigene10174_All, Unigene10200_All, Unigene10238_All, Unigene10254_All, Unigene10316_All, Unigene1040_All, Unigene10623_All, Unigene10723_All, Unigene10724_All, Unigene10779_All, Unigene10787_All, Unigene1096_All, Unigene10974_All, Unigene10983_All, Unigene10984_All, Unigene10991_All, Unigene11034_All, Unigene11097_All, Unigene11141_All, Unigene11160_All, Unigene11190_All, Unigene11203_All, Unigene11307_All, Unigene11337_All, Unigene11338_All, Unigene11358_All, Unigene11377_All, Unigene1137_All, Unigene11436_All, Unigene11447_All, Unigene11448_All, Unigene11469_All, Unigene11510_All, Unigene11511_All, Unigene11515_All, Unigene11538_All, Unigene11543_All, Unigene11544_All, Unigene11565_All, Unigene11576_All, Unigene11587_All, Unigene11588_All, Unigene11589_All, Unigene11590_All, Unigene11597_All, Unigene11598_All, Unigene11599_All, Unigene11628_All, Unigene11629_All, Unigene11648_All, Unigene11662_All, Unigene11672_All, Unigene11677_All, Unigene11687_All, Unigene1168_All, Unigene11797_All, Unigene11922_All, Unigene11985_All, Unigene12118_All, Unigene12125_All, Unigene12154_All, Unigene12155_All, Unigene12194_All, Unigene12210_All, Unigene12212_All, Unigene12435_All, Unigene12533_All, Unigene12537_All, Unigene1257_All, Unigene12684_All, Unigene12692_All, Unigene12710_All, Unigene12810_All, Unigene12974_All, Unigene13036_All, Unigene13092_All, Unigene13209_All, Unigene14133_All, Unigene1447_All, Unigene1479_All, Unigene1512_All, Unigene15513_All, Unigene15658_All, Unigene15840_All, Unigene16098_All, Unigene1614_All, Unigene1679_All, Unigene16965_All, Unigene16966_All, Unigene16994_All, Unigene17679_All, Unigene17728_All, Unigene17751_All, Unigene17815_All, Unigene17851_All, Unigene1792_All, Unigene1799_All, Unigene18178_All, Unigene18276_All, Unigene18317_All, Unigene18488_All, Unigene18496_All, Unigene1855_All, Unigene18598_All, Unigene18644_All, Unigene18873_All, Unigene18898_All, Unigene19237_All, Unigene1941_All, Unigene19426_All, Unigene19601_All, Unigene1974_All, Unigene19840_All, Unigene20111_All, Unigene20578_All, Unigene2067_All, Unigene20_All, Unigene21008_All, Unigene2145_All, Unigene214_All, Unigene2180_All, Unigene2190_All, Unigene220_All, Unigene2233_All, Unigene2251_All, Unigene2297_All, Unigene236_All, Unigene247_All, Unigene24_All, Unigene2503_All, Unigene2560_All, Unigene2741_All, Unigene2742_All, Unigene2749_All, Unigene278_All, Unigene28_All, Unigene3000_All, Unigene3063_All, Unigene3066_All, Unigene3070_All, Unigene3088_All, Unigene3123_All, Unigene3193_All, Unigene3261_All, Unigene327_All, Unigene3300_All, Unigene3316_All, Unigene3323_All, Unigene3346_All, Unigene3403_All, Unigene3471_All, Unigene352_All, Unigene3669_All, Unigene3889_All, Unigene3902_All, Unigene3945_All, Unigene3947_All, Unigene3964_All, Unigene4004_All, Unigene4022_All, Unigene4074_All, Unigene414_All, Unigene4207_All, Unigene4218_All, Unigene4418_All, Unigene4825_All, Unigene4922_All, Unigene4935_All, Unigene4970_All, Unigene5059_All, Unigene5088_All, Unigene5096_All, Unigene5108_All, Unigene5121_All, Unigene5169_All, Unigene5727_All, Unigene5799_All, Unigene579_All, Unigene5808_All, Unigene5813_All, Unigene5821_All, Unigene5889_All, Unigene5993_All, Unigene6059_All, Unigene6073_All, Unigene6076_All, Unigene6101_All, Unigene6283_All, Unigene6525_All, Unigene6655_All, Unigene6780_All, Unigene6886_All, Unigene6994_All, Unigene699_All, Unigene7016_All, Unigene7029_All, Unigene7054_All, Unigene7063_All, Unigene7094_All, Unigene7100_All, Unigene7106_All, Unigene7136_All, Unigene7220_All, Unigene7280_All, Unigene7297_All, Unigene7431_All, Unigene7440_All, Unigene7534_All, Unigene7547_All, Unigene7560_All, Unigene7667_All, Unigene7704_All, Unigene7759_All, Unigene783_All, Unigene7993_All, Unigene8017_All, Unigene8046_All, Unigene8102_All, Unigene8179_All, Unigene8204_All, Unigene8231_All, Unigene8262_All, Unigene8324_All, Unigene8399_All, Unigene8514_All, Unigene8537_All, Unigene8562_All, Unigene8585_All, Unigene8615_All, Unigene8725_All, Unigene8747_All, Unigene8784_All, Unigene8933_All, Unigene8936_All, Unigene9014_All, Unigene9042_All, Unigene9050_All, Unigene9059_All, Unigene9125_All, Unigene9258_All, Unigene9284_All, Unigene936_All, Unigene9379_All, Unigene9611_All, Unigene9693_All, Unigene9719_All, Unigene9836_All, Unigene9877_All, Unigene9904_All, Unigene9988_All

6	Alzheimer's disease	CL1087.Contig2_All, CL1087.Contig3_All, CL1146.Contig2_All, CL1146.Contig3_All, CL1286.Contig2_All, CL142.Contig1_All, CL142.Contig2_All, CL142.Contig5_All, CL1581.Contig2_All, CL1594.Contig1_All, CL1798.Contig1_All, CL1798.Contig2_All, CL1823.Contig8_All, CL1843.Contig3_All, CL1995.Contig1_All, CL1995.Contig2_All, CL1995.Contig3_All, CL2017.Contig4_All, CL2017.Contig5_All, CL2131.Contig4_All, CL2131.Contig5_All, CL2131.Contig6_All, CL2131.Contig8_All, CL2164.Contig2_All, CL2253.Contig2_All, CL2468.Contig1_All, CL2468.Contig2_All, CL2678.Contig1_All, CL2678.Contig2_All, CL2882.Contig1_All, CL2882.Contig2_All, CL2930.Contig5_All, CL3019.Contig2_All, CL3019.Contig3_All, CL3019.Contig4_All, CL3106.Contig1_All, CL3135.Contig1_All, CL3135.Contig2_All, CL3135.Contig3_All, CL3350.Contig1_All, CL3350.Contig2_All, CL345.Contig1_All, CL3667.Contig1_All, CL3667.Contig2_All, CL3667.Contig3_All, CL3730.Contig1_All, CL3941.Contig1_All, CL4304.Contig1_All, CL4304.Contig2_All, CL4365.Contig1_All, CL4395.Contig1_All, CL4509.Contig1_All, CL4509.Contig2_All, CL4521.Contig1_All, CL4521.Contig2_All, CL4521.Contig3_All, CL4521.Contig5_All, CL4628.Contig1_All, CL4663.Contig1_All, CL4872.Contig1_All, CL4872.Contig2_All, CL4881.Contig2_All, CL4918.Contig1_All, CL5012.Contig1_All, CL5064.Contig2_All, CL5078.Contig12_All, CL5078.Contig8_All, CL5260.Contig1_All, CL527.Contig2_All, CL527.Contig3_All, CL5299.Contig1_All, CL5337.Contig2_All, CL5399.Contig1_All, CL5399.Contig2_All, CL691.Contig1_All, CL772.Contig1_All, CL772.Contig2_All, CL823.Contig1_All, CL851.Contig3_All, CL870.Contig4_All, CL870.Contig8_All, CL870.Contig9_All, CL898.Contig1_All, CL898.Contig2_All, CL962.Contig3_All, Unigene10006_All, Unigene10037_All, Unigene10040_All, Unigene10073_All, Unigene10338_All, Unigene10373_All, Unigene10471_All, Unigene10570_All, Unigene10574_All, Unigene10674_All, Unigene10675_All, Unigene10716_All, Unigene10839_All, Unigene10846_All, Unigene10849_All, Unigene10856_All, Unigene10913_All, Unigene10919_All, Unigene10962_All, Unigene10968_All, Unigene10975_All, Unigene10988_All, Unigene10989_All, Unigene11009_All, Unigene11010_All, Unigene11028_All, Unigene11046_All, Unigene11047_All, Unigene11054_All, Unigene11074_All, Unigene11103_All, Unigene11104_All, Unigene11107_All, Unigene11108_All, Unigene11109_All, Unigene11136_All, Unigene11148_All, Unigene11149_All, Unigene11158_All, Unigene11404_All, Unigene11415_All, Unigene11440_All, Unigene11445_All, Unigene11450_All, Unigene11465_All, Unigene11494_All, Unigene11534_All, Unigene11560_All, Unigene11561_All, Unigene11568_All, Unigene11613_All, Unigene11614_All, Unigene11724_All, Unigene11756_All, Unigene11786_All, Unigene11792_All, Unigene11796_All, Unigene11802_All, Unigene11806_All, Unigene11809_All, Unigene11819_All, Unigene11832_All, Unigene11833_All, Unigene11836_All, Unigene11851_All, Unigene11853_All, Unigene11864_All, Unigene11868_All, Unigene11870_All, Unigene11871_All, Unigene11898_All, Unigene11904_All, Unigene11909_All, Unigene11912_All, Unigene11918_All, Unigene11921_All, Unigene11922_All, Unigene11926_All, Unigene11968_All, Unigene12004_All, Unigene12013_All, Unigene12018_All, Unigene12051_All, Unigene12065_All, Unigene12067_All, Unigene12075_All, Unigene12135_All, Unigene12162_All, Unigene12188_All, Unigene12207_All, Unigene12232_All, Unigene12256_All, Unigene12259_All, Unigene12279_All, Unigene12286_All, Unigene12315_All, Unigene12384_All, Unigene12407_All, Unigene12437_All, Unigene12502_All, Unigene12512_All, Unigene12602_All, Unigene12719_All, Unigene12800_All, Unigene12812_All, Unigene12873_All, Unigene12912_All, Unigene12917_All, Unigene12966_All, Unigene13177_All, Unigene13362_All, Unigene13422_All, Unigene13498_All, Unigene13638_All, Unigene13853_All, Unigene1417_All, Unigene14208_All, Unigene1515_All, Unigene15327_All, Unigene15384_All, Unigene1544_All, Unigene15629_All, Unigene15769_All, Unigene15950_All, Unigene16204_All, Unigene16218_All, Unigene16290_All, Unigene1634_All, Unigene16549_All, Unigene16599_All, Unigene16616_All, Unigene16660_All, Unigene16672_All, Unigene16761_All, Unigene16769_All, Unigene16807_All, Unigene17278_All, Unigene17406_All, Unigene17468_All, Unigene17618_All, Unigene17702_All, Unigene17865_All, Unigene18145_All, Unigene18358_All, Unigene1843_All, Unigene18495_All, Unigene18538_All, Unigene18539_All, Unigene18666_All, Unigene18993_All, Unigene19148_All, Unigene19218_All, Unigene19351_All, Unigene19388_All, Unigene19418_All, Unigene19523_All, Unigene19555_All, Unigene19844_All, Unigene20025_All, Unigene20152_All, Unigene2032_All, Unigene21092_All, Unigene21282_All, Unigene2209_All, Unigene236_All, Unigene2380_All, Unigene247_All, Unigene2626_All, Unigene2835_All, Unigene314_All, Unigene3691_All, Unigene3945_All, Unigene3996_All, Unigene4013_All, Unigene4053_All, Unigene4070_All, Unigene4510_All, Unigene4581_All, Unigene4940_All, Unigene5065_All, Unigene5203_All, Unigene5257_All, Unigene5605_All, Unigene5675_All, Unigene6309_All, Unigene6369_All, Unigene6605_All, Unigene661_All, Unigene6791_All, Unigene7049_All, Unigene7205_All, Unigene7297_All, Unigene7387_All, Unigene7667_All, Unigene7799_All, Unigene8107_All, Unigene812_All, Unigene8221_All, Unigene8347_All, Unigene8422_All, Unigene8621_All, Unigene8784_All, Unigene883_All, Unigene9282_All, Unigene9312_All, Unigene9437_All, Unigene9663_All, Unigene9664_All, Unigene9995_All, Unigene9996_All

7	Huntington’s disease	CL1146.Contig2_All, CL1146.Contig3_All, CL1594.Contig1_All, CL1798.Contig1_All, CL1798.Contig2_All, CL1810.Contig1_All, CL1810.Contig2_All, CL1991.Contig2_All, CL1991.Contig3_All, CL1991.Contig4_All, CL1995.Contig1_All, CL1995.Contig2_All, CL1995.Contig3_All, CL2019.Contig1_All, CL2019.Contig2_All, CL2164.Contig2_All, CL2170.Contig2_All, CL2170.Contig5_All, CL2678.Contig1_All, CL2678.Contig2_All, CL2848.Contig4_All, CL2882.Contig1_All, CL2882.Contig2_All, CL2930.Contig5_All, CL3019.Contig2_All, CL3019.Contig3_All, CL3019.Contig4_All, CL3135.Contig1_All, CL3135.Contig2_All, CL3135.Contig3_All, CL3200.Contig2_All, CL3420.Contig1_All, CL3664.Contig1_All, CL3664.Contig2_All, CL3730.Contig1_All, CL3920.Contig2_All, CL4105.Contig2_All, CL4304.Contig1_All, CL4304.Contig2_All, CL4365.Contig1_All, CL4555.Contig1_All, CL4663.Contig1_All, CL475.Contig4_All, CL4872.Contig1_All, CL4872.Contig2_All, CL4881.Contig2_All, CL5012.Contig1_All, CL5078.Contig12_All, CL5078.Contig8_All, CL5098.Contig2_All, CL527.Contig2_All, CL527.Contig3_All, CL5299.Contig1_All, CL5399.Contig1_All, CL5399.Contig2_All, CL5433.Contig2_All, CL598.Contig1_All, CL598.Contig2_All, CL691.Contig1_All, CL851.Contig3_All, CL870.Contig4_All, CL870.Contig8_All, CL870.Contig9_All, CL962.Contig3_All, Unigene10037_All, Unigene10073_All, Unigene10338_All, Unigene10570_All, Unigene10674_All, Unigene10675_All, Unigene10716_All, Unigene10817_All, Unigene10839_All, Unigene10846_All, Unigene10849_All, Unigene10856_All, Unigene10887_All, Unigene10913_All, Unigene10919_All, Unigene10948_All, Unigene10962_All, Unigene10968_All, Unigene10975_All, Unigene10988_All, Unigene10989_All, Unigene11009_All, Unigene11010_All, Unigene11028_All, Unigene11046_All, Unigene11047_All, Unigene11074_All, Unigene11103_All, Unigene11104_All, Unigene11107_All, Unigene11108_All, Unigene11109_All, Unigene11136_All, Unigene11148_All, Unigene11149_All, Unigene11158_All, Unigene11404_All, Unigene11412_All, Unigene11415_All, Unigene11422_All, Unigene11440_All, Unigene11445_All, Unigene11450_All, Unigene11465_All, Unigene11494_All, Unigene11534_All, Unigene11568_All, Unigene11613_All, Unigene11614_All, Unigene11724_All, Unigene11756_All, Unigene11786_All, Unigene11792_All, Unigene11796_All, Unigene11802_All, Unigene11806_All, Unigene11809_All, Unigene11819_All, Unigene11832_All, Unigene11833_All, Unigene11836_All, Unigene11851_All, Unigene11853_All, Unigene11860_All, Unigene11864_All, Unigene11868_All, Unigene11870_All, Unigene11871_All, Unigene11898_All, Unigene11904_All, Unigene11909_All, Unigene11912_All, Unigene11918_All, Unigene11921_All, Unigene11926_All, Unigene11935_All, Unigene11968_All, Unigene12004_All, Unigene12013_All, Unigene12017_All, Unigene12018_All, Unigene12051_All, Unigene12060_All, Unigene12065_All, Unigene12067_All, Unigene12075_All, Unigene12080_All, Unigene12096_All, Unigene12117_All, Unigene12135_All, Unigene12162_All, Unigene12175_All, Unigene12186_All, Unigene12188_All, Unigene12207_All, Unigene12232_All, Unigene12256_All, Unigene12279_All, Unigene12286_All, Unigene12331_All, Unigene12384_All, Unigene12437_All, Unigene12479_All, Unigene12488_All, Unigene12502_All, Unigene12512_All, Unigene12602_All, Unigene1278_All, Unigene12800_All, Unigene12812_All, Unigene12873_All, Unigene12912_All, Unigene12917_All, Unigene12966_All, Unigene13177_All, Unigene13225_All, Unigene13284_All, Unigene13422_All, Unigene13498_All, Unigene13638_All, Unigene13978_All, Unigene1417_All, Unigene1459_All, Unigene1515_All, Unigene15327_All, Unigene15384_All, Unigene1544_All, Unigene15629_All, Unigene15827_All, Unigene15855_All, Unigene15950_All, Unigene16204_All, Unigene16218_All, Unigene1634_All, Unigene16538_All, Unigene16599_All, Unigene16616_All, Unigene1663_All, Unigene16660_All, Unigene16672_All, Unigene16761_All, Unigene16807_All, Unigene17278_All, Unigene17406_All, Unigene17468_All, Unigene17478_All, Unigene17618_All, Unigene17674_All, Unigene17675_All, Unigene17702_All, Unigene17865_All, Unigene18145_All, Unigene18284_All, Unigene18358_All, Unigene1843_All, Unigene18538_All, Unigene18539_All, Unigene18950_All, Unigene18993_All, Unigene19116_All, Unigene19148_All, Unigene19218_All, Unigene19288_All, Unigene19351_All, Unigene19388_All, Unigene19418_All, Unigene19523_All, Unigene19555_All, Unigene19880_All, Unigene20025_All, Unigene21092_All, Unigene21282_All, Unigene2209_All, Unigene236_All, Unigene2380_All, Unigene2513_All, Unigene2570_All, Unigene2626_All, Unigene2760_All, Unigene2835_All, Unigene294_All, Unigene314_All, Unigene3241_All, Unigene3419_All, Unigene3691_All, Unigene4053_All, Unigene4070_All, Unigene4162_All, Unigene4251_All, Unigene4581_All, Unigene5179_All, Unigene5203_All, Unigene5245_All, Unigene54_All, Unigene5589_All, Unigene5605_All, Unigene5716_All, Unigene5745_All, Unigene5781_All, Unigene6242_All, Unigene6309_All, Unigene6369_All, Unigene6605_All, Unigene661_All, Unigene7049_All, Unigene7205_All, Unigene7387_All, Unigene7737_All, Unigene7751_All, Unigene7799_All, Unigene8002_All, Unigene807_All, Unigene8422_All, Unigene8605_All, Unigene883_All, Unigene9039_All, Unigene9282_All, Unigene9312_All, Unigene9437_All, Unigene9663_All, Unigene9664_All, Unigene9989_All, Unigene9995_All, Unigene9996_All

8	Cardiac muscle contraction	CL1075.Contig3_All, CL1087.Contig2_All, CL1087.Contig3_All, CL1141.Contig2_All, CL156.Contig4_All, CL1594.Contig1_All, CL1687.Contig4_All, CL1798.Contig1_All, CL1798.Contig2_All, CL2137.Contig1_All, CL2137.Contig2_All, CL2265.Contig1_All, CL2265.Contig2_All, CL2474.Contig2_All, CL2904.Contig1_All, CL2904.Contig2_All, CL3011.Contig1_All, CL3019.Contig2_All, CL3019.Contig3_All, CL3019.Contig4_All, CL3046.Contig1_All, CL3092.Contig1_All, CL3092.Contig2_All, CL3135.Contig1_All, CL3135.Contig2_All, CL3135.Contig3_All, CL3260.Contig1_All, CL3260.Contig2_All, CL3260.Contig3_All, CL3260.Contig4_All, CL3260.Contig5_All, CL3260.Contig7_All, CL3295.Contig1_All, CL3297.Contig1_All, CL3297.Contig2_All, CL3347.Contig1_All, CL3347.Contig2_All, CL349.Contig12_All, CL349.Contig3_All, CL349.Contig5_All, CL3543.Contig2_All, CL3543.Contig3_All, CL3543.Contig4_All, CL3672.Contig4_All, CL3761.Contig1_All, CL3776.Contig2_All, CL41.Contig3_All, CL41.Contig4_All, CL41.Contig8_All, CL4187.Contig1_All, CL4250.Contig2_All, CL4304.Contig1_All, CL4304.Contig2_All, CL4332.Contig1_All, CL4332.Contig2_All, CL4454.Contig3_All, CL4596.Contig1_All, CL4872.Contig1_All, CL4872.Contig2_All, CL5012.Contig1_All, CL503.Contig2_All, CL503.Contig3_All, CL503.Contig4_All, CL503.Contig5_All, CL503.Contig6_All, CL503.Contig8_All, CL531.Contig1_All, CL573.Contig1_All, CL642.Contig1_All, CL642.Contig2_All, CL644.Contig1_All, CL703.Contig2_All, CL703.Contig3_All, CL703.Contig5_All, CL703.Contig6_All, CL923.Contig1_All, CL923.Contig2_All, CL923.Contig3_All, CL962.Contig3_All, Unigene10037_All, Unigene10040_All, Unigene10201_All, Unigene10202_All, Unigene10338_All, Unigene10471_All, Unigene10594_All, Unigene10674_All, Unigene10675_All, Unigene10856_All, Unigene10913_All, Unigene10988_All, Unigene10989_All, Unigene11107_All, Unigene11136_All, Unigene11148_All, Unigene11149_All, Unigene11158_All, Unigene11289_All, Unigene11298_All, Unigene11300_All, Unigene1131_All, Unigene11345_All, Unigene11404_All, Unigene11415_All, Unigene11445_All, Unigene11480_All, Unigene11482_All, Unigene11483_All, Unigene11494_All, Unigene11534_All, Unigene11568_All, Unigene11756_All, Unigene11792_All, Unigene11796_All, Unigene11802_All, Unigene11806_All, Unigene11819_All, Unigene11833_All, Unigene11853_All, Unigene11923_All, Unigene11935_All, Unigene12067_All, Unigene12279_All, Unigene12418_All, Unigene12476_All, Unigene12658_All, Unigene12697_All, Unigene12719_All, Unigene12812_All, Unigene12828_All, Unigene12873_All, Unigene13638_All, Unigene1515_All, Unigene15271_All, Unigene15384_All, Unigene15950_All, Unigene16218_All, Unigene1633_All, Unigene1634_All, Unigene16435_All, Unigene16510_All, Unigene16616_All, Unigene16716_All, Unigene16807_All, Unigene16874_All, Unigene16976_All, Unigene1705_All, Unigene17117_All, Unigene17278_All, Unigene17362_All, Unigene17398_All, Unigene17406_All, Unigene17468_All, Unigene17618_All, Unigene17697_All, Unigene17718_All, Unigene17773_All, Unigene1777_All, Unigene17824_All, Unigene18106_All, Unigene18145_All, Unigene18215_All, Unigene18358_All, Unigene18378_All, Unigene18538_All, Unigene18539_All, Unigene18645_All, Unigene19289_All, Unigene19351_All, Unigene19364_All, Unigene19388_All, Unigene19418_All, Unigene19453_All, Unigene19647_All, Unigene19745_All, Unigene20065_All, Unigene20299_All, Unigene2032_All, Unigene2052_All, Unigene20530_All, Unigene2053_All, Unigene20625_All, Unigene210_All, Unigene237_All, Unigene2514_All, Unigene3010_All, Unigene3300_All, Unigene3328_All, Unigene3659_All, Unigene3691_All, Unigene3696_All, Unigene4161_All, Unigene4269_All, Unigene4323_All, Unigene4692_All, Unigene4920_All, Unigene500_All, Unigene5724_All, Unigene5994_All, Unigene6013_All, Unigene6037_All, Unigene6215_All, Unigene6273_All, Unigene6369_All, Unigene6605_All, Unigene6896_All, Unigene6907_All, Unigene7063_All, Unigene7129_All, Unigene7206_All, Unigene7799_All, Unigene7829_All, Unigene7854_All, Unigene785_All, Unigene8009_All, Unigene8621_All, Unigene9039_All, Unigene9076_All, Unigene9163_All, Unigene9282_All, Unigene9663_All, Unigene9722_All

9	Hypertrophic cardiomyopathy (HCM)	CL1075.Contig3_All, CL1079.Contig4_All, CL1087.Contig2_All, CL1087.Contig3_All, CL1132.Contig1_All, CL1132.Contig2_All, CL1141.Contig2_All, CL1355.Contig1_All, CL156.Contig1_All, CL156.Contig3_All, CL156.Contig4_All, CL1687.Contig4_All, CL1698.Contig2_All, CL1704.Contig1_All, CL1704.Contig2_All, CL1803.Contig1_All, CL2265.Contig1_All, CL2265.Contig2_All, CL2331.Contig2_All, CL2331.Contig3_All, CL2517.Contig4_All, CL2843.Contig2_All, CL2904.Contig1_All, CL2904.Contig2_All, CL3011.Contig1_All, CL3046.Contig1_All, CL3092.Contig1_All, CL3092.Contig2_All, CL3112.Contig3_All, CL3260.Contig1_All, CL3260.Contig2_All, CL3260.Contig3_All, CL3260.Contig4_All, CL3260.Contig5_All, CL3260.Contig7_All, CL3295.Contig1_All, CL3297.Contig1_All, CL3297.Contig2_All, CL3347.Contig1_All, CL3347.Contig2_All, CL3428.Contig1_All, CL3428.Contig2_All, CL3442.Contig1_All, CL349.Contig12_All, CL349.Contig3_All, CL349.Contig5_All, CL3543.Contig2_All, CL3543.Contig3_All, CL3543.Contig4_All, CL3554.Contig1_All, CL3590.Contig2_All, CL3672.Contig4_All, CL3761.Contig1_All, CL408.Contig1_All, CL408.Contig3_All, CL408.Contig4_All, CL4187.Contig1_All, CL4250.Contig2_All, CL4332.Contig1_All, CL4332.Contig2_All, CL4454.Contig3_All, CL4596.Contig1_All, CL470.Contig1_All, CL4992.Contig1_All, CL503.Contig2_All, CL503.Contig3_All, CL503.Contig4_All, CL503.Contig5_All, CL503.Contig6_All, CL503.Contig8_All, CL531.Contig1_All, CL5352.Contig1_All, CL5352.Contig2_All, CL5468.Contig3_All, CL573.Contig1_All, CL636.Contig1_All, CL636.Contig2_All, CL642.Contig1_All, CL642.Contig2_All, CL644.Contig1_All, CL703.Contig2_All, CL703.Contig3_All, CL703.Contig5_All, CL703.Contig6_All, CL923.Contig1_All, CL923.Contig2_All, CL923.Contig3_All, Unigene10040_All, Unigene10201_All, Unigene10202_All, Unigene10471_All, Unigene11289_All, Unigene11298_All, Unigene11300_All, Unigene1131_All, Unigene11345_All, Unigene11597_All, Unigene11598_All, Unigene11599_All, Unigene11623_All, Unigene11923_All, Unigene11935_All, Unigene12418_All, Unigene12476_All, Unigene1257_All, Unigene12627_All, Unigene12658_All, Unigene12684_All, Unigene12697_All, Unigene12719_All, Unigene12828_All, Unigene13092_All, Unigene136_All, Unigene15271_All, Unigene15824_All, Unigene1633_All, Unigene16435_All, Unigene16510_All, Unigene16716_All, Unigene16874_All, Unigene16976_All, Unigene1705_All, Unigene17116_All, Unigene17117_All, Unigene17279_All, Unigene17362_All, Unigene17398_All, Unigene17697_All, Unigene17718_All, Unigene17773_All, Unigene1777_All, Unigene17824_All, Unigene18076_All, Unigene18106_All, Unigene18173_All, Unigene18215_All, Unigene18378_All, Unigene18598_All, Unigene18645_All, Unigene19289_All, Unigene19364_All, Unigene19453_All, Unigene19647_All, Unigene19738_All, Unigene19745_All, Unigene19866_All, Unigene20065_All, Unigene2020_All, Unigene20299_All, Unigene2032_All, Unigene2052_All, Unigene20530_All, Unigene2053_All, Unigene20625_All, Unigene210_All, Unigene237_All, Unigene2503_All, Unigene2514_All, Unigene2741_All, Unigene278_All, Unigene2980_All, Unigene3061_All, Unigene3187_All, Unigene3300_All, Unigene3328_All, Unigene3407_All, Unigene3659_All, Unigene3696_All, Unigene3945_All, Unigene4052_All, Unigene4161_All, Unigene4207_All, Unigene4269_All, Unigene4406_All, Unigene4692_All, Unigene4920_All, Unigene500_All, Unigene5722_All, Unigene58_All, Unigene5994_All, Unigene6013_All, Unigene6037_All, Unigene6273_All, Unigene6284_All, Unigene6896_All, Unigene6907_All, Unigene7015_All, Unigene7063_All, Unigene7096_All, Unigene7129_All, Unigene7829_All, Unigene7854_All, Unigene785_All, Unigene8009_All, Unigene8482_All, Unigene8621_All, Unigene8830_All, Unigene8959_All, Unigene9039_All, Unigene9062_All, Unigene9076_All, Unigene9200_All, Unigene9722_All
10	Viral myocarditis	CL1046.Contig1_All, CL1046.Contig2_All, CL1046.Contig3_All, CL1075.Contig3_All, CL1079.Contig4_All, CL1141.Contig2_All, CL1355.Contig1_All, CL1535.Contig3_All, CL156.Contig1_All, CL156.Contig3_All, CL156.Contig4_All, CL1687.Contig4_All, CL1803.Contig1_All, CL2265.Contig1_All, CL2265.Contig2_All, CL2517.Contig4_All, CL257.Contig1_All, CL257.Contig2_All, CL2882.Contig1_All, CL2882.Contig2_All, CL2904.Contig1_All, CL2904.Contig2_All, CL3011.Contig1_All, CL3046.Contig1_All, CL3092.Contig1_All, CL3092.Contig2_All, CL3260.Contig1_All, CL3260.Contig2_All, CL3260.Contig3_All, CL3260.Contig4_All, CL3260.Contig5_All, CL3260.Contig7_All, CL3295.Contig1_All, CL3297.Contig1_All, CL3297.Contig2_All, CL3347.Contig1_All, CL3347.Contig2_All, CL3442.Contig1_All, CL3543.Contig2_All, CL3543.Contig3_All, CL3543.Contig4_All, CL3554.Contig1_All, CL3672.Contig4_All, CL3761.Contig1_All, CL4187.Contig1_All, CL4250.Contig2_All, CL4454.Contig3_All, CL4596.Contig1_All, CL470.Contig1_All, CL4992.Contig1_All, CL503.Contig2_All, CL503.Contig3_All, CL503.Contig4_All, CL503.Contig5_All, CL503.Contig6_All, CL503.Contig8_All, CL5352.Contig1_All, CL5352.Contig2_All, CL5468.Contig3_All, CL573.Contig1_All, CL644.Contig1_All, CL923.Contig1_All, CL923.Contig2_All, CL923.Contig3_All, Unigene10201_All, Unigene10202_All, Unigene11289_All, Unigene11298_All, Unigene11300_All, Unigene1131_All, Unigene11597_All, Unigene11598_All, Unigene11599_All, Unigene11724_All, Unigene11869_All, Unigene11935_All, Unigene12188_All, Unigene12418_All, Unigene12658_All, Unigene12684_All, Unigene12697_All, Unigene12828_All, Unigene13092_All, Unigene136_All, Unigene15271_All, Unigene15824_All, Unigene1633_All, Unigene16435_All, Unigene16510_All, Unigene16716_All, Unigene16874_All, Unigene16976_All, Unigene1705_All, Unigene17117_All, Unigene17362_All, Unigene17702_All, Unigene1777_All, Unigene17824_All, Unigene18106_All, Unigene18215_All, Unigene18378_All, Unigene18598_All, Unigene18645_All, Unigene19289_All, Unigene19364_All, Unigene19453_All, Unigene19647_All, Unigene19745_All, Unigene198_All, Unigene20065_All, Unigene20260_All, Unigene20299_All, Unigene2052_All, Unigene20530_All, Unigene2053_All, Unigene20625_All, Unigene210_All, Unigene237_All, Unigene2503_All, Unigene2514_All, Unigene2741_All, Unigene278_All, Unigene3187_All, Unigene3328_All, Unigene3659_All, Unigene3696_All, Unigene4161_All, Unigene4269_All, Unigene4581_All, Unigene4692_All, Unigene4920_All, Unigene500_All, Unigene5092_All, Unigene5994_All, Unigene6013_All, Unigene6014_All, Unigene6037_All, Unigene6273_All, Unigene6896_All, Unigene6907_All, Unigene7063_All, Unigene7096_All, Unigene7129_All, Unigene7829_All, Unigene7854_All, Unigene785_All, Unigene7983_All, Unigene8009_All, Unigene8933_All, Unigene9039_All, Unigene9062_All, Unigene9076_All, Unigene9722_All

11	ECM-receptor interaction	CL1011.Contig1_All, CL1011.Contig4_All, CL1055.Contig1_All, CL1055.Contig3_All, CL1055.Contig4_All, CL1055.Contig5_All, CL1055.Contig6_All, CL1055.Contig7_All, CL1062.Contig2_All, CL1079.Contig4_All, CL1092.Contig1_All, CL1147.Contig1_All, CL1147.Contig2_All, CL1202.Contig1_All, CL1202.Contig2_All, CL1370.Contig4_All, CL1436.Contig2_All, CL1473.Contig1_All, CL1495.Contig1_All, CL1495.Contig2_All, CL1566.Contig1_All, CL1566.Contig2_All, CL1718.Contig1_All, CL1718.Contig2_All, CL1890.Contig4_All, CL2009.Contig2_All, CL209.Contig1_All, CL209.Contig2_All, CL209.Contig3_All, CL209.Contig4_All, CL2099.Contig1_All, CL2252.Contig1_All, CL2252.Contig2_All, CL2252.Contig3_All, CL2265.Contig1_All, CL2265.Contig2_All, CL2297.Contig2_All, CL2331.Contig2_All, CL2331.Contig3_All, CL2517.Contig4_All, CL2611.Contig1_All, CL2611.Contig2_All, CL2927.Contig1_All, CL2927.Contig3_All, CL2952.Contig1_All, CL3327.Contig1_All, CL3327.Contig2_All, CL3371.Contig1_All, CL3387.Contig1_All, CL3387.Contig2_All, CL3442.Contig1_All, CL3797.Contig1_All, CL3797.Contig2_All, CL3879.Contig2_All, CL4026.Contig1_All, CL4042.Contig1_All, CL4042.Contig2_All, CL4042.Contig3_All, CL4087.Contig1_All, CL4401.Contig2_All, CL4401.Contig3_All, CL4401.Contig4_All, CL4511.Contig1_All, CL4654.Contig2_All, CL4690.Contig1_All, CL4690.Contig2_All, CL470.Contig1_All, CL4844.Contig1_All, CL4844.Contig2_All, CL498.Contig2_All, CL498.Contig4_All, CL4992.Contig1_All, CL5048.Contig1_All, CL5221.Contig1_All, CL5222.Contig2_All, CL5268.Contig2_All, CL5268.Contig3_All, CL5373.Contig1_All, CL5373.Contig2_All, CL5394.Contig2_All, CL5464.Contig1_All, CL5464.Contig2_All, CL5464.Contig3_All, CL577.Contig1_All, CL577.Contig2_All, CL80.Contig1_All, CL986.Contig2_All, Unigene10012_All, Unigene10093_All, Unigene10095_All, Unigene10096_All, Unigene10123_All, Unigene10200_All, Unigene10238_All, Unigene10254_All, Unigene10316_All, Unigene1040_All, Unigene10623_All, Unigene10723_All, Unigene10724_All, Unigene10779_All, Unigene10787_All, Unigene1096_All, Unigene10974_All, Unigene10983_All, Unigene10984_All, Unigene10991_All, Unigene11034_All, Unigene11097_All, Unigene11141_All, Unigene11160_All, Unigene11190_All, Unigene11203_All, Unigene11337_All, Unigene11338_All, Unigene11358_All, Unigene11377_All, Unigene11436_All, Unigene11447_All, Unigene11448_All, Unigene11469_All, Unigene11510_All, Unigene11511_All, Unigene11515_All, Unigene11538_All, Unigene11543_All, Unigene11544_All, Unigene11565_All, Unigene11576_All, Unigene11587_All, Unigene11588_All, Unigene11589_All, Unigene11590_All, Unigene11629_All, Unigene11648_All, Unigene11662_All, Unigene11672_All, Unigene11687_All, Unigene11797_All, Unigene11985_All, Unigene12118_All, Unigene12125_All, Unigene12154_All, Unigene12155_All, Unigene12185_All, Unigene12194_All, Unigene12210_All, Unigene12212_All, Unigene12435_All, Unigene12533_All, Unigene12537_All, Unigene12692_All, Unigene12710_All, Unigene12974_All, Unigene14133_All, Unigene15320_All, Unigene15321_All, Unigene15367_All, Unigene15513_All, Unigene15658_All, Unigene15824_All, Unigene15840_All, Unigene1614_All, Unigene16906_All, Unigene16965_All, Unigene16966_All, Unigene17324_All, Unigene17679_All, Unigene17728_All, Unigene17851_All, Unigene1792_All, Unigene1799_All, Unigene18488_All, Unigene18496_All, Unigene19237_All, Unigene19426_All, Unigene19840_All, Unigene20073_All, Unigene20_All, Unigene2105_All, Unigene2180_All, Unigene2190_All, Unigene2251_All, Unigene2297_All, Unigene2503_All, Unigene2560_All, Unigene28_All, Unigene3000_All, Unigene3063_All, Unigene3066_All, Unigene3123_All, Unigene3193_All, Unigene3261_All, Unigene3316_All, Unigene3323_All, Unigene3324_All, Unigene3346_All, Unigene3403_All, Unigene352_All, Unigene3669_All, Unigene3889_All, Unigene3902_All, Unigene3945_All, Unigene3947_All, Unigene3964_All, Unigene4004_All, Unigene4074_All, Unigene414_All, Unigene4207_All, Unigene4218_All, Unigene4418_All, Unigene4757_All, Unigene4825_All, Unigene4922_All, Unigene5059_All, Unigene5096_All, Unigene5108_All, Unigene5121_All, Unigene5169_All, Unigene5799_All, Unigene579_All, Unigene5889_All, Unigene6076_All, Unigene6780_All, Unigene6886_All, Unigene690_All, Unigene7029_All, Unigene7054_All, Unigene7094_All, Unigene7100_All, Unigene7106_All, Unigene7280_All, Unigene7353_All, Unigene7431_All, Unigene7440_All, Unigene7534_All, Unigene7560_All, Unigene7836_All, Unigene8102_All, Unigene8179_All, Unigene8204_All, Unigene8231_All, Unigene8262_All, Unigene8324_All, Unigene8399_All, Unigene8514_All, Unigene8537_All, Unigene8562_All, Unigene8915_All, Unigene8936_All, Unigene9258_All, Unigene9284_All, Unigene936_All, Unigene9611_All, Unigene9988_All

12	Tight junction	CL1075.Contig3_All, CL1141.Contig2_All, CL128.Contig10_All, CL1355.Contig1_All, CL1503.Contig3_All, CL1503.Contig4_All, CL1503.Contig5_All, CL156.Contig1_All, CL156.Contig3_All, CL156.Contig4_All, CL1687.Contig4_All, CL1803.Contig1_All, CL1929.Contig5_All, CL1929.Contig6_All, CL2034.Contig1_All, CL2034.Contig2_All, CL2085.Contig1_All, CL2182.Contig1_All, CL2182.Contig3_All, CL2182.Contig4_All, CL2265.Contig1_All, CL2265.Contig2_All, CL2552.Contig3_All, CL2614.Contig10_All, CL2614.Contig11_All, CL2614.Contig12_All, CL2614.Contig4_All, CL271.Contig2_All, CL2791.Contig2_All, CL2873.Contig1_All, CL2873.Contig2_All, CL2904.Contig1_All, CL2904.Contig2_All, CL2909.Contig1_All, CL2909.Contig3_All, CL2909.Contig4_All, CL3011.Contig1_All, CL3046.Contig1_All, CL3047.Contig1_All, CL3047.Contig2_All, CL3092.Contig1_All, CL3092.Contig2_All, CL3231.Contig1_All, CL3231.Contig2_All, CL3260.Contig1_All, CL3260.Contig2_All, CL3260.Contig3_All, CL3260.Contig4_All, CL3260.Contig5_All, CL3260.Contig7_All, CL3295.Contig1_All, CL3297.Contig1_All, CL3297.Contig2_All, CL3347.Contig1_All, CL3347.Contig2_All, CL3348.Contig1_All, CL3436.Contig1_All, CL3436.Contig4_All, CL3436.Contig9_All, CL3460.Contig2_All, CL3460.Contig3_All, CL3498.Contig1_All, CL3498.Contig2_All, CL3543.Contig2_All, CL3543.Contig3_All, CL3543.Contig4_All, CL358.Contig1_All, CL362.Contig2_All, CL362.Contig3_All, CL362.Contig4_All, CL3672.Contig4_All, CL3761.Contig1_All, CL3819.Contig1_All, CL3819.Contig2_All, CL39.Contig1_All, CL39.Contig2_All, CL39.Contig3_All, CL39.Contig4_All, CL3939.Contig1_All, CL3939.Contig2_All, CL402.Contig1_All, CL4040.Contig1_All, CL4187.Contig1_All, CL4250.Contig2_All, CL4420.Contig1_All, CL4420.Contig2_All, CL4439.Contig2_All, CL4454.Contig3_All, CL4479.Contig2_All, CL4479.Contig3_All, CL4479.Contig5_All, CL4591.Contig2_All, CL4596.Contig1_All, CL4855.Contig1_All, CL4855.Contig2_All, CL4896.Contig1_All, CL503.Contig2_All, CL503.Contig3_All, CL503.Contig4_All, CL503.Contig5_All, CL503.Contig6_All, CL503.Contig8_All, CL5159.Contig1_All, CL5159.Contig2_All, CL523.Contig1_All, CL523.Contig2_All, CL5299.Contig1_All, CL5330.Contig1_All, CL5330.Contig2_All, CL538.Contig1_All, CL5391.Contig1_All, CL5391.Contig2_All, CL540.Contig2_All, CL5468.Contig3_All, CL573.Contig1_All, CL593.Contig1_All, CL622.Contig1_All, CL622.Contig2_All, CL622.Contig4_All, CL626.Contig3_All, CL644.Contig1_All, CL697.Contig1_All, CL697.Contig2_All, CL709.Contig2_All, CL709.Contig3_All, CL724.Contig1_All, CL761.Contig1_All, CL761.Contig2_All, CL866.Contig3_All, CL923.Contig1_All, CL923.Contig2_All, CL923.Contig3_All, CL940.Contig3_All, CL940.Contig5_All, Unigene10201_All, Unigene10202_All, Unigene10242_All, Unigene10619_All, Unigene10863_All, Unigene11289_All, Unigene11298_All, Unigene11300_All, Unigene11307_All, Unigene1131_All, Unigene11597_All, Unigene11598_All, Unigene11599_All, Unigene11607_All, Unigene11823_All, Unigene11874_All, Unigene11935_All, Unigene12057_All, Unigene12099_All, Unigene12314_All, Unigene12418_All, Unigene12591_All, Unigene12658_All, Unigene12671_All, Unigene12684_All, Unigene12697_All, Unigene12828_All, Unigene13092_All, Unigene13134_All, Unigene13432_All, Unigene15271_All, Unigene15806_All, Unigene1633_All, Unigene16435_All, Unigene16510_All, Unigene16716_All, Unigene16874_All, Unigene16976_All, Unigene1705_All, Unigene17117_All, Unigene17206_All, Unigene17362_All, Unigene17628_All, Unigene1777_All, Unigene17824_All, Unigene18106_All, Unigene18215_All, Unigene18317_All, Unigene18378_All, Unigene18423_All, Unigene18598_All, Unigene18645_All, Unigene18873_All, Unigene18898_All, Unigene18903_All, Unigene18923_All, Unigene19289_All, Unigene19347_All, Unigene19364_All, Unigene19453_All, Unigene19647_All, Unigene19708_All, Unigene19745_All, Unigene20065_All, Unigene20299_All, Unigene2052_All, Unigene20530_All, Unigene20535_All, Unigene2053_All, Unigene20625_All, Unigene210_All, Unigene21582_All, Unigene2166_All, Unigene2374_All, Unigene237_All, Unigene2514_All, Unigene256_All, Unigene2671_All, Unigene2741_All, Unigene2749_All, Unigene278_All, Unigene3300_All, Unigene3328_All, Unigene3659_All, Unigene3696_All, Unigene3751_All, Unigene4056_All, Unigene4133_All, Unigene4161_All, Unigene4269_All, Unigene4500_All, Unigene4692_All, Unigene4920_All, Unigene500_All, Unigene5075_All, Unigene5378_All, Unigene5521_All, Unigene5727_All, Unigene5808_All, Unigene5994_All, Unigene6010_All, Unigene6013_All, Unigene6037_All, Unigene6217_All, Unigene6273_All, Unigene63_All, Unigene6896_All, Unigene6907_All, Unigene7063_All, Unigene7129_All, Unigene7158_All, Unigene7829_All, Unigene783_All, Unigene7854_All, Unigene785_All, Unigene8009_All, Unigene8056_All, Unigene8061_All, Unigene8146_All, Unigene8247_All, Unigene9039_All, Unigene9042_All, Unigene9050_All, Unigene9076_All, Unigene9249_All, Unigene9722_All, Unigene9730_All, Unigene9877_All

13	Oxidative phosphorylation	CL1146.Contig2_All, CL1146.Contig3_All, CL1447.Contig2_All, CL1594.Contig1_All, CL1798.Contig1_All, CL1798.Contig2_All, CL1995.Contig1_All, CL1995.Contig2_All, CL1995.Contig3_All, CL2128.Contig1_All, CL2150.Contig3_All, CL2164.Contig2_All, CL2634.Contig1_All, CL2634.Contig2_All, CL2678.Contig1_All, CL2678.Contig2_All, CL3019.Contig2_All, CL3019.Contig3_All, CL3019.Contig4_All, CL3091.Contig1_All, CL3091.Contig2_All, CL3091.Contig3_All, CL3091.Contig4_All, CL3135.Contig1_All, CL3135.Contig2_All, CL3135.Contig3_All, CL3730.Contig1_All, CL3884.Contig2_All, CL4090.Contig2_All, CL41.Contig4_All, CL41.Contig8_All, CL4304.Contig1_All, CL4304.Contig2_All, CL4365.Contig1_All, CL4459.Contig1_All, CL4460.Contig1_All, CL4482.Contig2_All, CL4663.Contig1_All, CL4872.Contig1_All, CL4872.Contig2_All, CL4881.Contig2_All, CL5012.Contig1_All, CL5078.Contig12_All, CL5078.Contig8_All, CL527.Contig2_All, CL527.Contig3_All, CL962.Contig3_All, Unigene10026_All, Unigene10037_All, Unigene10073_All, Unigene10338_All, Unigene10483_All, Unigene10570_All, Unigene10674_All, Unigene10675_All, Unigene10716_All, Unigene10829_All, Unigene10839_All, Unigene10846_All, Unigene10849_All, Unigene10856_All, Unigene10898_All, Unigene10899_All, Unigene10913_All, Unigene10919_All, Unigene10962_All, Unigene10968_All, Unigene10975_All, Unigene10988_All, Unigene10989_All, Unigene11009_All, Unigene11010_All, Unigene11028_All, Unigene11046_All, Unigene11047_All, Unigene11074_All, Unigene11103_All, Unigene11104_All, Unigene11105_All, Unigene11106_All, Unigene11107_All, Unigene11108_All, Unigene11109_All, Unigene11114_All, Unigene11136_All, Unigene11148_All, Unigene11149_All, Unigene11158_All, Unigene11240_All, Unigene11386_All, Unigene11404_All, Unigene11415_All, Unigene11419_All, Unigene11420_All, Unigene11440_All, Unigene11445_All, Unigene11450_All, Unigene11465_All, Unigene11494_All, Unigene11534_All, Unigene11568_All, Unigene11613_All, Unigene11614_All, Unigene11756_All, Unigene11767_All, Unigene11768_All, Unigene11778_All, Unigene11786_All, Unigene11792_All, Unigene11796_All, Unigene11802_All, Unigene11806_All, Unigene11809_All, Unigene11817_All, Unigene11819_All, Unigene11832_All, Unigene11833_All, Unigene11836_All, Unigene11851_All, Unigene11853_All, Unigene11864_All, Unigene11868_All, Unigene11870_All, Unigene11871_All, Unigene11898_All, Unigene11899_All, Unigene1189_All, Unigene11904_All, Unigene11909_All, Unigene11912_All, Unigene11918_All, Unigene11921_All, Unigene11926_All, Unigene11948_All, Unigene11966_All, Unigene11968_All, Unigene12004_All, Unigene12013_All, Unigene12018_All, Unigene12028_All, Unigene12051_All, Unigene12062_All, Unigene12065_All, Unigene12067_All, Unigene12075_All, Unigene12135_All, Unigene12162_All, Unigene12168_All, Unigene12207_All, Unigene12209_All, Unigene12232_All, Unigene12256_All, Unigene12279_All, Unigene12286_All, Unigene12302_All, Unigene12329_All, Unigene12384_All, Unigene12423_All, Unigene12437_All, Unigene12502_All, Unigene12512_All, Unigene12602_All, Unigene12744_All, Unigene12757_All, Unigene12800_All, Unigene12812_All, Unigene12873_All, Unigene12912_All, Unigene12917_All, Unigene12923_All, Unigene12966_All, Unigene13177_All, Unigene13422_All, Unigene13498_All, Unigene13508_All, Unigene13589_All, Unigene13638_All, Unigene1515_All, Unigene15327_All, Unigene15384_All, Unigene1544_All, Unigene15629_All, Unigene15950_All, Unigene15969_All, Unigene16021_All, Unigene16204_All, Unigene16218_All, Unigene1634_All, Unigene16599_All, Unigene16616_All, Unigene16660_All, Unigene16672_All, Unigene16761_All, Unigene16807_All, Unigene17278_All, Unigene17406_All, Unigene1741_All, Unigene17468_All, Unigene17602_All, Unigene17618_All, Unigene17865_All, Unigene18145_All, Unigene18358_All, Unigene1843_All, Unigene18538_All, Unigene18539_All, Unigene18707_All, Unigene19218_All, Unigene19351_All, Unigene19388_All, Unigene19418_All, Unigene19517_All, Unigene19523_All, Unigene19555_All, Unigene19602_All, Unigene20025_All, Unigene20295_All, Unigene20614_All, Unigene21092_All, Unigene21282_All, Unigene2380_All, Unigene2626_All, Unigene2835_All, Unigene314_All, Unigene3351_All, Unigene3691_All, Unigene3730_All, Unigene5203_All, Unigene5605_All, Unigene6309_All, Unigene6369_All, Unigene6605_All, Unigene671_All, Unigene7387_All, Unigene7799_All, Unigene883_All, Unigene925_All, Unigene9282_All, Unigene9312_All, Unigene9663_All, Unigene9995_All, Unigene9996_All

14	Salmonella infection	CL1075.Contig3_All, CL1120.Contig2_All, CL1141.Contig2_All, CL1355.Contig1_All, CL1535.Contig3_All, CL156.Contig1_All, CL156.Contig3_All, CL156.Contig4_All, CL1687.Contig4_All, CL1723.Contig1_All, CL1723.Contig2_All, CL1723.Contig7_All, CL1803.Contig1_All, CL2049.Contig3_All, CL2224.Contig2_All, CL2224.Contig3_All, CL2224.Contig4_All, CL2265.Contig1_All, CL2265.Contig2_All, CL2375.Contig2_All, CL2437.Contig1_All, CL2437.Contig2_All, CL271.Contig2_All, CL2764.Contig2_All, CL284.Contig2_All, CL284.Contig3_All, CL284.Contig4_All, CL284.Contig5_All, CL2904.Contig1_All, CL2904.Contig2_All, CL2975.Contig2_All, CL3011.Contig1_All, CL3027.Contig3_All, CL3046.Contig1_All, CL3092.Contig1_All, CL3092.Contig2_All, CL3114.Contig2_All, CL3260.Contig1_All, CL3260.Contig2_All, CL3260.Contig3_All, CL3260.Contig4_All, CL3260.Contig5_All, CL3260.Contig7_All, CL3282.Contig1_All, CL3282.Contig2_All, CL3282.Contig3_All, CL3295.Contig1_All, CL3297.Contig1_All, CL3297.Contig2_All, CL3315.Contig3_All, CL3315.Contig4_All, CL3347.Contig1_All, CL3347.Contig2_All, CL3510.Contig3_All, CL3543.Contig2_All, CL3543.Contig3_All, CL3543.Contig4_All, CL3622.Contig2_All, CL3672.Contig4_All, CL3761.Contig1_All, CL3823.Contig1_All, CL3823.Contig2_All, CL4088.Contig2_All, CL4130.Contig1_All, CL4187.Contig1_All, CL4250.Contig2_All, CL4320.Contig3_All, CL4454.Contig3_All, CL4596.Contig1_All, CL4661.Contig1_All, CL4661.Contig2_All, CL4736.Contig1_All, CL4736.Contig2_All, CL4966.Contig1_All, CL503.Contig2_All, CL503.Contig3_All, CL503.Contig4_All, CL503.Contig5_All, CL503.Contig6_All, CL503.Contig8_All, CL5115.Contig1_All, CL5468.Contig3_All, CL573.Contig1_All, CL644.Contig1_All, CL923.Contig1_All, CL923.Contig2_All, CL923.Contig3_All, Unigene10097_All, Unigene10201_All, Unigene10202_All, Unigene1023_All, Unigene1028_All, Unigene10448_All, Unigene10998_All, Unigene11082_All, Unigene11289_All, Unigene11298_All, Unigene11300_All, Unigene1131_All, Unigene11372_All, Unigene11459_All, Unigene11521_All, Unigene11597_All, Unigene11598_All, Unigene11599_All, Unigene11935_All, Unigene12201_All, Unigene12330_All, Unigene12343_All, Unigene12418_All, Unigene12603_All, Unigene12658_All, Unigene12684_All, Unigene12697_All, Unigene12711_All, Unigene12828_All, Unigene12853_All, Unigene13092_All, Unigene13209_All, Unigene1447_All, Unigene1479_All, Unigene1496_All, Unigene15271_All, Unigene1633_All, Unigene16435_All, Unigene16510_All, Unigene16716_All, Unigene16840_All, Unigene16874_All, Unigene16963_All, Unigene16976_All, Unigene1705_All, Unigene17117_All, Unigene17362_All, Unigene1757_All, Unigene17751_All, Unigene1777_All, Unigene17824_All, Unigene18106_All, Unigene18215_All, Unigene18378_All, Unigene18598_All, Unigene18644_All, Unigene18645_All, Unigene19289_All, Unigene19364_All, Unigene19453_All, Unigene19647_All, Unigene19745_All, Unigene20065_All, Unigene20095_All, Unigene20299_All, Unigene2052_All, Unigene20530_All, Unigene2053_All, Unigene20578_All, Unigene20625_All, Unigene2067_All, Unigene210_All, Unigene220_All, Unigene237_All, Unigene2514_All, Unigene2741_All, Unigene278_All, Unigene3070_All, Unigene3094_All, Unigene327_All, Unigene3328_All, Unigene3482_All, Unigene3659_All, Unigene3696_All, Unigene4133_All, Unigene4161_All, Unigene4269_All, Unigene4692_All, Unigene4920_All, Unigene500_All, Unigene5303_All, Unigene5688_All, Unigene5873_All, Unigene5994_All, Unigene6013_All, Unigene6014_All, Unigene6029_All, Unigene6037_All, Unigene6062_All, Unigene6273_All, Unigene6896_All, Unigene6907_All, Unigene7063_All, Unigene7129_All, Unigene7829_All, Unigene7854_All, Unigene785_All, Unigene8009_All, Unigene8151_All, Unigene8184_All, Unigene9039_All, Unigene9076_All, Unigene9722_All, Unigene9785_All, Unigene9787_All, Unigene9902_All, Unigene9947_All
15	Melanogenesis	CL1286.Contig2_All, CL1452.Contig1_All, CL1685.Contig2_All, CL1823.Contig8_All, CL1843.Contig3_All, CL2131.Contig4_All, CL2131.Contig5_All, CL2131.Contig6_All, CL2131.Contig8_All, CL2170.Contig2_All, CL2170.Contig5_All, CL2182.Contig1_All, CL2182.Contig3_All, CL2182.Contig4_All, CL2208.Contig1_All, CL2468.Contig1_All, CL2468.Contig2_All, CL252.Contig2_All, CL252.Contig3_All, CL2930.Contig5_All, CL3156.Contig1_All, CL3567.Contig1_All, CL3567.Contig2_All, CL3567.Contig3_All, CL3667.Contig1_All, CL3667.Contig2_All, CL3667.Contig3_All, CL39.Contig1_All, CL39.Contig2_All, CL39.Contig3_All, CL39.Contig4_All, CL3920.Contig2_All, CL4005.Contig2_All, CL4005.Contig3_All, CL4262.Contig2_All, CL4395.Contig1_All, CL44.Contig10_All, CL44.Contig12_All, CL44.Contig13_All, CL44.Contig16_All, CL44.Contig17_All, CL44.Contig18_All, CL44.Contig19_All, CL44.Contig1_All, CL44.Contig22_All, CL44.Contig23_All, CL44.Contig2_All, CL44.Contig3_All, CL44.Contig4_All, CL44.Contig5_All, CL44.Contig6_All, CL44.Contig8_All, CL44.Contig9_All, CL440.Contig3_All, CL440.Contig5_All, CL440.Contig6_All, CL4439.Contig2_All, CL4509.Contig1_All, CL4509.Contig2_All, CL4657.Contig2_All, CL4787.Contig1_All, CL4923.Contig2_All, CL5111.Contig1_All, CL5391.Contig1_All, CL5391.Contig2_All, CL5399.Contig1_All, CL5399.Contig2_All, CL772.Contig1_All, CL772.Contig2_All, CL940.Contig3_All, CL940.Contig5_All, Unigene10006_All, Unigene10058_All, Unigene10226_All, Unigene10574_All, Unigene10747_All, Unigene1099_All, Unigene11054_All, Unigene11922_All, Unigene12315_All, Unigene12671_All, Unigene12719_All, Unigene16769_All, Unigene1743_All, Unigene176_All, Unigene18898_All, Unigene2029_All, Unigene2196_All, Unigene2209_All, Unigene236_All, Unigene247_All, Unigene2760_All, Unigene2915_All, Unigene3779_All, Unigene3975_All, Unigene3996_All, Unigene4013_All, Unigene4053_All, Unigene4070_All, Unigene4112_All, Unigene4126_All, Unigene4532_All, Unigene4731_All, Unigene54_All, Unigene5521_All, Unigene5675_All, Unigene5727_All, Unigene5781_All, Unigene6162_All, Unigene6217_All, Unigene69_All, Unigene7163_All, Unigene7297_All, Unigene766_All, Unigene777_All, Unigene783_All, Unigene8014_All, Unigene8213_All, Unigene8422_All, Unigene8784_All, Unigene8861_All, Unigene93_All, Unigene9437_All, Unigene9733_All

16	Parkinson's disease	CL1146.Contig2_All, CL1146.Contig3_All, CL1594.Contig1_All, CL1798.Contig1_All, CL1798.Contig2_All, CL1991.Contig2_All, CL1991.Contig3_All, CL1991.Contig4_All, CL1995.Contig1_All, CL1995.Contig2_All, CL1995.Contig3_All, CL2091.Contig5_All, CL2091.Contig6_All, CL2128.Contig1_All, CL2164.Contig2_All, CL2678.Contig1_All, CL2678.Contig2_All, CL2882.Contig1_All, CL2882.Contig2_All, CL3019.Contig2_All, CL3019.Contig3_All, CL3019.Contig4_All, CL3135.Contig1_All, CL3135.Contig2_All, CL3135.Contig3_All, CL3286.Contig1_All, CL3730.Contig1_All, CL4105.Contig2_All, CL4304.Contig1_All, CL4304.Contig2_All, CL4365.Contig1_All, CL4663.Contig1_All, CL475.Contig4_All, CL4757.Contig2_All, CL4872.Contig1_All, CL4872.Contig2_All, CL4881.Contig2_All, CL5010.Contig2_All, CL5012.Contig1_All, CL5078.Contig12_All, CL5078.Contig8_All, CL527.Contig2_All, CL527.Contig3_All, CL5299.Contig1_All, CL584.Contig17_All, CL851.Contig3_All, CL962.Contig3_All, Unigene10037_All, Unigene10073_All, Unigene10338_All, Unigene10570_All, Unigene10674_All, Unigene10675_All, Unigene10716_All, Unigene10817_All, Unigene10839_All, Unigene10846_All, Unigene10849_All, Unigene10856_All, Unigene10898_All, Unigene10899_All, Unigene10913_All, Unigene10919_All, Unigene10962_All, Unigene10968_All, Unigene10975_All, Unigene10988_All, Unigene10989_All, Unigene11009_All, Unigene11010_All, Unigene11028_All, Unigene11046_All, Unigene11047_All, Unigene11074_All, Unigene11103_All, Unigene11104_All, Unigene11107_All, Unigene11108_All, Unigene11109_All, Unigene11136_All, Unigene11148_All, Unigene11149_All, Unigene11158_All, Unigene11404_All, Unigene11415_All, Unigene11419_All, Unigene11420_All, Unigene11422_All, Unigene11440_All, Unigene11445_All, Unigene11450_All, Unigene11465_All, Unigene11494_All, Unigene11534_All, Unigene11568_All, Unigene11613_All, Unigene11614_All, Unigene11724_All, Unigene11756_All, Unigene11778_All, Unigene11786_All, Unigene11792_All, Unigene11796_All, Unigene11802_All, Unigene11806_All, Unigene11809_All, Unigene11817_All, Unigene11819_All, Unigene11828_All, Unigene11832_All, Unigene11833_All, Unigene11836_All, Unigene11851_All, Unigene11853_All, Unigene11860_All, Unigene11864_All, Unigene11868_All, Unigene11870_All, Unigene11871_All, Unigene11898_All, Unigene11904_All, Unigene11909_All, Unigene11912_All, Unigene11918_All, Unigene11921_All, Unigene11926_All, Unigene11948_All, Unigene11968_All, Unigene12004_All, Unigene12013_All, Unigene12018_All, Unigene12051_All, Unigene12062_All, Unigene12065_All, Unigene12067_All, Unigene12075_All, Unigene12081_All, Unigene12135_All, Unigene12157_All, Unigene12162_All, Unigene12188_All, Unigene12207_All, Unigene12232_All, Unigene12256_All, Unigene12279_All, Unigene12286_All, Unigene12329_All, Unigene12331_All, Unigene12384_All, Unigene12437_All, Unigene12502_All, Unigene12512_All, Unigene12602_All, Unigene12744_All, Unigene12800_All, Unigene12812_All, Unigene12873_All, Unigene12912_All, Unigene12917_All, Unigene12966_All, Unigene13177_All, Unigene13422_All, Unigene13498_All, Unigene13638_All, Unigene1459_All, Unigene1515_All, Unigene15327_All, Unigene15384_All, Unigene1544_All, Unigene15629_All, Unigene15855_All, Unigene15950_All, Unigene16204_All, Unigene16218_All, Unigene1634_All, Unigene16538_All, Unigene16599_All, Unigene16616_All, Unigene16660_All, Unigene16672_All, Unigene16761_All, Unigene16807_All, Unigene17278_All, Unigene17406_All, Unigene17468_All, Unigene17478_All, Unigene17618_All, Unigene17674_All, Unigene17675_All, Unigene17702_All, Unigene17865_All, Unigene18145_All, Unigene18284_All, Unigene18358_All, Unigene1843_All, Unigene18538_All, Unigene18539_All, Unigene19116_All, Unigene19218_All, Unigene19351_All, Unigene19388_All, Unigene19418_All, Unigene19523_All, Unigene19555_All, Unigene19602_All, Unigene20025_All, Unigene20614_All, Unigene21092_All, Unigene21282_All, Unigene2193_All, Unigene2380_All, Unigene2626_All, Unigene2835_All, Unigene314_All, Unigene3691_All, Unigene3791_All, Unigene4116_All, Unigene4581_All, Unigene5203_All, Unigene5605_All, Unigene6309_All, Unigene6369_All, Unigene6605_All, Unigene6931_All, Unigene7049_All, Unigene7205_All, Unigene7298_All, Unigene7387_All, Unigene7799_All, Unigene8605_All, Unigene883_All, Unigene9282_All, Unigene9312_All, Unigene9663_All, Unigene9664_All, Unigene9989_All, Unigene9995_All, Unigene9996_All
17	Salivary secretion	CL126.Contig2_All, CL1286.Contig2_All, CL1452.Contig1_All, CL1453.Contig2_All, CL1507.Contig1_All, CL1507.Contig2_All, CL1559.Contig1_All, CL1613.Contig1_All, CL1823.Contig8_All, CL1880.Contig1_All, CL1880.Contig2_All, CL1880.Contig3_All, CL191.Contig1_All, CL191.Contig2_All, CL1987.Contig1_All, CL2131.Contig4_All, CL2131.Contig5_All, CL2131.Contig6_All, CL2131.Contig8_All, CL2137.Contig1_All, CL2137.Contig2_All, CL2182.Contig1_All, CL2182.Contig3_All, CL2182.Contig4_All, CL2468.Contig1_All, CL2468.Contig2_All, CL2474.Contig2_All, CL2478.Contig2_All, CL2734.Contig1_All, CL2930.Contig5_All, CL2975.Contig2_All, CL3027.Contig2_All, CL3311.Contig1_All, CL3311.Contig2_All, CL3489.Contig1_All, CL3667.Contig1_All, CL3667.Contig2_All, CL3667.Contig3_All, CL3776.Contig2_All, CL39.Contig1_All, CL39.Contig2_All, CL39.Contig3_All, CL39.Contig4_All, CL4005.Contig2_All, CL4005.Contig3_All, CL4084.Contig1_All, CL4084.Contig2_All, CL41.Contig3_All, CL41.Contig4_All, CL41.Contig8_All, CL4130.Contig2_All, CL4181.Contig1_All, CL4181.Contig4_All, CL4262.Contig2_All, CL4395.Contig1_All, CL440.Contig3_All, CL440.Contig5_All, CL440.Contig6_All, CL4509.Contig1_All, CL4509.Contig2_All, CL467.Contig2_All, CL4917.Contig1_All, CL5308.Contig1_All, CL5372.Contig2_All, CL5391.Contig1_All, CL5391.Contig2_All, CL5399.Contig1_All, CL5399.Contig2_All, CL573.Contig6_All, CL600.Contig1_All, CL600.Contig2_All, CL600.Contig3_All, CL600.Contig4_All, CL600.Contig6_All, CL648.Contig2_All, CL691.Contig1_All, CL718.Contig1_All, CL772.Contig1_All, CL772.Contig2_All, CL847.Contig1_All, CL878.Contig1_All, CL878.Contig2_All, CL878.Contig3_All, CL878.Contig4_All, CL878.Contig5_All, CL940.Contig3_All, CL940.Contig5_All, Unigene10006_All, Unigene10058_All, Unigene10574_All, Unigene10594_All, Unigene11054_All, Unigene11480_All, Unigene11482_All, Unigene11483_All, Unigene11694_All, Unigene11730_All, Unigene11863_All, Unigene11973_All, Unigene12016_All, Unigene12170_All, Unigene12315_All, Unigene125_All, Unigene12719_All, Unigene12926_All, Unigene15176_All, Unigene15194_All, Unigene15195_All, Unigene15815_All, Unigene1671_All, Unigene16769_All, Unigene18179_All, Unigene18652_All, Unigene18898_All, Unigene18993_All, Unigene19223_All, Unigene20111_All, Unigene2029_All, Unigene2209_All, Unigene2236_All, Unigene2250_All, Unigene2274_All, Unigene2476_All, Unigene2763_All, Unigene2795_All, Unigene3010_All, Unigene3177_All, Unigene3779_All, Unigene3861_All, Unigene3996_All, Unigene4013_All, Unigene4053_All, Unigene4070_All, Unigene4112_All, Unigene4323_All, Unigene4532_All, Unigene4783_All, Unigene490_All, Unigene5086_All, Unigene5105_All, Unigene5303_All, Unigene554_All, Unigene5675_All, Unigene5724_All, Unigene6215_All, Unigene62_All, Unigene6597_All, Unigene7054_All, Unigene7102_All, Unigene7206_All, Unigene7284_All, Unigene8109_All, Unigene8208_All, Unigene8422_All, Unigene8464_All, Unigene8700_All, Unigene8770_All, Unigene9122_All, Unigene9163_All, Unigene93_All, Unigene9437_All, Unigene9733_All

18	Gastric acid secretion	CL1068.Contig5_All, CL1286.Contig2_All, CL1355.Contig1_All, CL1452.Contig1_All, CL156.Contig1_All, CL156.Contig3_All, CL156.Contig4_All, CL1803.Contig1_All, CL1823.Contig8_All, CL2039.Contig1_All, CL2061.Contig1_All, CL2131.Contig4_All, CL2131.Contig5_All, CL2131.Contig6_All, CL2131.Contig8_All, CL2137.Contig1_All, CL2137.Contig2_All, CL214.Contig3_All, CL2182.Contig1_All, CL2182.Contig3_All, CL2182.Contig4_All, CL2468.Contig1_All, CL2468.Contig2_All, CL2474.Contig2_All, CL263.Contig3_All, CL2720.Contig1_All, CL2930.Contig5_All, CL305.Contig2_All, CL336.Contig1_All, CL336.Contig2_All, CL3500.Contig1_All, CL3525.Contig1_All, CL3525.Contig2_All, CL3545.Contig2_All, CL3667.Contig1_All, CL3667.Contig2_All, CL3667.Contig3_All, CL3776.Contig2_All, CL39.Contig1_All, CL39.Contig2_All, CL39.Contig3_All, CL39.Contig4_All, CL392.Contig1_All, CL392.Contig2_All, CL392.Contig3_All, CL4005.Contig2_All, CL4005.Contig3_All, CL41.Contig3_All, CL41.Contig4_All, CL41.Contig8_All, CL4216.Contig1_All, CL4216.Contig2_All, CL4262.Contig2_All, CL4395.Contig1_All, CL44.Contig10_All, CL44.Contig12_All, CL44.Contig13_All, CL44.Contig16_All, CL44.Contig17_All, CL44.Contig18_All, CL44.Contig19_All, CL44.Contig1_All, CL44.Contig22_All, CL44.Contig23_All, CL44.Contig2_All, CL44.Contig3_All, CL44.Contig4_All, CL44.Contig5_All, CL44.Contig6_All, CL44.Contig8_All, CL44.Contig9_All, CL440.Contig3_All, CL440.Contig5_All, CL440.Contig6_All, CL4402.Contig1_All, CL4509.Contig1_All, CL4509.Contig2_All, CL4637.Contig1_All, CL4862.Contig1_All, CL4928.Contig1_All, CL4928.Contig2_All, CL5061.Contig2_All, CL5246.Contig3_All, CL5391.Contig1_All, CL5391.Contig2_All, CL5399.Contig1_All, CL5399.Contig2_All, CL5441.Contig3_All, CL5468.Contig3_All, CL571.Contig2_All, CL600.Contig1_All, CL600.Contig2_All, CL600.Contig3_All, CL600.Contig4_All, CL600.Contig6_All, CL691.Contig1_All, CL772.Contig1_All, CL772.Contig2_All, CL916.Contig2_All, CL940.Contig3_All, CL940.Contig5_All, CL970.Contig2_All, CL974.Contig2_All, Unigene10006_All, Unigene10058_All, Unigene10574_All, Unigene10594_All, Unigene11054_All, Unigene1111_All, Unigene1137_All, Unigene11480_All, Unigene11482_All, Unigene11483_All, Unigene11597_All, Unigene11598_All, Unigene11599_All, Unigene12315_All, Unigene1257_All, Unigene12684_All, Unigene12719_All, Unigene13092_All, Unigene1427_All, Unigene15194_All, Unigene15195_All, Unigene16098_All, Unigene16769_All, Unigene16994_All, Unigene17815_All, Unigene18178_All, Unigene18276_All, Unigene18598_All, Unigene18898_All, Unigene18993_All, Unigene1974_All, Unigene2002_All, Unigene2003_All, Unigene20111_All, Unigene2029_All, Unigene21008_All, Unigene2145_All, Unigene214_All, Unigene2209_All, Unigene24_All, Unigene2741_All, Unigene278_All, Unigene3010_All, Unigene3471_All, Unigene3748_All, Unigene3779_All, Unigene3996_All, Unigene4013_All, Unigene4053_All, Unigene4070_All, Unigene4112_All, Unigene4211_All, Unigene4323_All, Unigene4532_All, Unigene5088_All, Unigene5521_All, Unigene5675_All, Unigene5724_All, Unigene5821_All, Unigene5993_All, Unigene6059_All, Unigene6073_All, Unigene6215_All, Unigene6217_All, Unigene6525_All, Unigene6972_All, Unigene7061_All, Unigene7110_All, Unigene7206_All, Unigene7993_All, Unigene8017_All, Unigene8422_All, Unigene8464_All, Unigene8615_All, Unigene8770_All, Unigene9014_All, Unigene9059_All, Unigene9071_All, Unigene9163_All, Unigene93_All, Unigene9437_All, Unigene9668_All, Unigene9733_All, Unigene9836_All

19	Endocytosis	CL102.Contig1_All, CL102.Contig2_All, CL1054.Contig1_All, CL1054.Contig2_All, CL1066.Contig2_All, CL1066.Contig3_All, CL1155.Contig1_All, CL1155.Contig2_All, CL1155.Contig3_All, CL1225.Contig6_All, CL1435.Contig1_All, CL1435.Contig2_All, CL1435.Contig3_All, CL1435.Contig4_All, CL1522.Contig10_All, CL1522.Contig1_All, CL1534.Contig1_All, CL1534.Contig2_All, CL1546.Contig1_All, CL1546.Contig3_All, CL1546.Contig4_All, CL1546.Contig5_All, CL1546.Contig6_All, CL1546.Contig7_All, CL1546.Contig8_All, CL1548.Contig2_All, CL1548.Contig3_All, CL1622.Contig1_All, CL1622.Contig2_All, CL1622.Contig3_All, CL1845.Contig1_All, CL1901.Contig3_All, CL1925.Contig1_All, CL1953.Contig1_All, CL1974.Contig1_All, CL1974.Contig2_All, CL2019.Contig1_All, CL2019.Contig2_All, CL2068.Contig5_All, CL2165.Contig2_All, CL2190.Contig2_All, CL2224.Contig2_All, CL2224.Contig3_All, CL2224.Contig4_All, CL2355.Contig1_All, CL241.Contig1_All, CL241.Contig7_All, CL2441.Contig1_All, CL2441.Contig2_All, CL2642.Contig1_All, CL2642.Contig2_All, CL2658.Contig1_All, CL2658.Contig2_All, CL2663.Contig1_All, CL2663.Contig2_All, CL2674.Contig1_All, CL2674.Contig2_All, CL2674.Contig3_All, CL2675.Contig1_All, CL2675.Contig2_All, CL2686.Contig1_All, CL2686.Contig2_All, CL271.Contig2_All, CL2734.Contig1_All, CL2853.Contig1_All, CL2853.Contig4_All, CL309.Contig2_All, CL309.Contig3_All, CL309.Contig4_All, CL3183.Contig2_All, CL3188.Contig1_All, CL3188.Contig2_All, CL3200.Contig2_All, CL3221.Contig1_All, CL3270.Contig2_All, CL3274.Contig1_All, CL3332.Contig1_All, CL3332.Contig2_All, CL3460.Contig2_All, CL3460.Contig3_All, CL3526.Contig5_All, CL3694.Contig1_All, CL3694.Contig2_All, CL3738.Contig1_All, CL3845.Contig1_All, CL3959.Contig1_All, CL3959.Contig2_All, CL3967.Contig2_All, CL399.Contig10_All, CL399.Contig1_All, CL399.Contig2_All, CL399.Contig3_All, CL399.Contig4_All, CL399.Contig5_All, CL399.Contig6_All, CL399.Contig7_All, CL399.Contig8_All, CL399.Contig9_All, CL4040.Contig1_All, CL4187.Contig1_All, CL4228.Contig1_All, CL4228.Contig2_All, CL4250.Contig2_All, CL4271.Contig1_All, CL4273.Contig2_All, CL4381.Contig1_All, CL4439.Contig2_All, CL4541.Contig1_All, CL4541.Contig2_All, CL4591.Contig2_All, CL4596.Contig2_All, CL4722.Contig2_All, CL4748.Contig2_All, CL4765.Contig1_All, CL4765.Contig2_All, CL4766.Contig1_All, CL4766.Contig2_All, CL4789.Contig2_All, CL495.Contig11_All, CL495.Contig1_All, CL495.Contig3_All, CL495.Contig4_All, CL495.Contig5_All, CL495.Contig6_All, CL5256.Contig1_All, CL5256.Contig2_All, CL5256.Contig4_All, CL5272.Contig1_All, CL5466.Contig1_All, CL558.Contig1_All, CL558.Contig2_All, CL558.Contig4_All, CL58.Contig1_All, CL58.Contig2_All, CL622.Contig1_All, CL622.Contig2_All, CL622.Contig4_All, CL724.Contig1_All, CL95.Contig1_All, Unigene10664_All, Unigene1074_All, Unigene10837_All, Unigene10855_All, Unigene11086_All, Unigene11264_All, Unigene11265_All, Unigene11307_All, Unigene1131_All, Unigene1142_All, Unigene11446_All, Unigene11549_All, Unigene12044_All, Unigene12328_All, Unigene12517_All, Unigene12810_All, Unigene12813_All, Unigene12853_All, Unigene13225_All, Unigene13732_All, Unigene13978_All, Unigene14005_All, Unigene14401_All, Unigene15587_All, Unigene15863_All, Unigene16093_All, Unigene16747_All, Unigene1679_All, Unigene1769_All, Unigene18106_All, Unigene18535_All, Unigene18600_All, Unigene18950_All, Unigene1935_All, Unigene20226_All, Unigene20260_All, Unigene2053_All, Unigene2247_All, Unigene2279_All, Unigene2667_All, Unigene322_All, Unigene3684_All, Unigene3819_All, Unigene4137_All, Unigene4149_All, Unigene4671_All, Unigene4753_All, Unigene4789_All, Unigene4801_All, Unigene555_All, Unigene5651_All, Unigene5664_All, Unigene5716_All, Unigene5808_All, Unigene5835_All, Unigene5875_All, Unigene5912_All, Unigene6013_All, Unigene6550_All, Unigene6706_All, Unigene6907_All, Unigene699_All, Unigene7018_All, Unigene7056_All, Unigene7108_All, Unigene7129_All, Unigene7348_All, Unigene746_All, Unigene7751_All, Unigene781_All, Unigene7838_All, Unigene8046_All, Unigene8151_All, Unigene8662_All, Unigene8737_All, Unigene8933_All, Unigene8937_All, Unigene9050_All, Unigene9661_All, Unigene9730_All, Unigene9759_All, Unigene9877_All, Unigene9921_All, Unigene9971_All

20	Phagosome	CL1085.Contig3_All, CL1355.Contig1_All, CL1358.Contig1_All, CL1358.Contig2_All, CL1358.Contig3_All, CL1534.Contig1_All, CL1534.Contig2_All, CL1535.Contig3_All, CL156.Contig1_All, CL156.Contig3_All, CL156.Contig4_All, CL1776.Contig1_All, CL1803.Contig1_All, CL2150.Contig3_All, CL2190.Contig2_All, CL2224.Contig2_All, CL2224.Contig3_All, CL2224.Contig4_All, CL2355.Contig1_All, CL2437.Contig1_All, CL2437.Contig2_All, CL2634.Contig1_All, CL2634.Contig2_All, CL2750.Contig3_All, CL3091.Contig1_All, CL3091.Contig2_All, CL3091.Contig3_All, CL3091.Contig4_All, CL3221.Contig1_All, CL326.Contig1_All, CL3274.Contig1_All, CL3282.Contig1_All, CL3282.Contig2_All, CL3282.Contig3_All, CL3506.Contig1_All, CL3506.Contig2_All, CL3706.Contig1_All, CL3884.Contig2_All, CL4057.Contig1_All, CL4090.Contig2_All, CL4187.Contig1_All, CL4247.Contig2_All, CL4247.Contig3_All, CL4250.Contig2_All, CL4317.Contig1_All, CL4459.Contig1_All, CL4576.Contig1_All, CL4596.Contig2_All, CL4633.Contig1_All, CL4633.Contig2_All, CL4661.Contig1_All, CL4661.Contig2_All, CL4666.Contig1_All, CL4666.Contig2_All, CL4776.Contig1_All, CL4776.Contig2_All, CL4857.Contig1_All, CL5034.Contig1_All, CL5034.Contig2_All, CL5149.Contig1_All, CL5149.Contig2_All, CL5186.Contig2_All, CL5207.Contig2_All, CL5466.Contig1_All, CL5468.Contig3_All, CL621.Contig2_All, CL621.Contig4_All, CL621.Contig5_All, CL724.Contig1_All, Unigene10026_All, Unigene10125_All, Unigene10126_All, Unigene10128_All, Unigene1016_All, Unigene1017_All, Unigene1028_All, Unigene10296_All, Unigene10423_All, Unigene10424_All, Unigene10433_All, Unigene10483_All, Unigene10503_All, Unigene1050_All, Unigene11121_All, Unigene11130_All, Unigene11240_All, Unigene11270_All, Unigene11282_All, Unigene1131_All, Unigene11369_All, Unigene11375_All, Unigene11395_All, Unigene11564_All, Unigene11589_All, Unigene11597_All, Unigene11598_All, Unigene11599_All, Unigene11845_All, Unigene11879_All, Unigene11884_All, Unigene11899_All, Unigene1189_All, Unigene11901_All, Unigene11908_All, Unigene11973_All, Unigene11992_All, Unigene12028_All, Unigene12168_All, Unigene12203_All, Unigene12209_All, Unigene12302_All, Unigene12330_All, Unigene12343_All, Unigene12423_All, Unigene12684_All, Unigene12710_All, Unigene12713_All, Unigene12853_All, Unigene12923_All, Unigene13092_All, Unigene13324_All, Unigene13396_All, Unigene13508_All, Unigene13589_All, Unigene13732_All, Unigene15308_All, Unigene15969_All, Unigene16098_All, Unigene16747_All, Unigene1741_All, Unigene17602_All, Unigene18095_All, Unigene18106_All, Unigene18114_All, Unigene18195_All, Unigene18598_All, Unigene18707_All, Unigene18950_All, Unigene19920_All, Unigene20095_All, Unigene20260_All, Unigene2053_All, Unigene2111_All, Unigene2275_All, Unigene2741_All, Unigene278_All, Unigene2961_All, Unigene2982_All, Unigene3094_All, Unigene3157_All, Unigene3351_All, Unigene3730_All, Unigene3945_All, Unigene4159_All, Unigene4207_All, Unigene420_All, Unigene44_All, Unigene4921_All, Unigene5448_All, Unigene5688_All, Unigene6013_All, Unigene615_All, Unigene6907_All, Unigene6915_All, Unigene6916_All, Unigene7056_All, Unigene7129_All, Unigene7723_All, Unigene7767_All, Unigene8151_All, Unigene8662_All, Unigene8737_All, Unigene9217_All, Unigene9229_All, Unigene9251_All, Unigene925_All, Unigene9604_All, Unigene9928_All, Unigene9972_All, Unigene9973_All, Unigene9984_All
21	Endocrine and other factor-regulated calcium reabsorption	CL1452.Contig1_All, CL1880.Contig1_All, CL1880.Contig2_All, CL1880.Contig3_All, CL2019.Contig1_All, CL2019.Contig2_All, CL2137.Contig1_All, CL2137.Contig2_All, CL2182.Contig1_All, CL2182.Contig3_All, CL2182.Contig4_All, CL2474.Contig2_All, CL2711.Contig1_All, CL2711.Contig2_All, CL2930.Contig5_All, CL3200.Contig2_All, CL3776.Contig2_All, CL39.Contig1_All, CL39.Contig2_All, CL39.Contig3_All, CL39.Contig4_All, CL399.Contig10_All, CL399.Contig1_All, CL399.Contig2_All, CL399.Contig3_All, CL399.Contig4_All, CL399.Contig5_All, CL399.Contig6_All, CL399.Contig7_All, CL399.Contig8_All, CL399.Contig9_All, CL4084.Contig1_All, CL4084.Contig2_All, CL41.Contig3_All, CL41.Contig4_All, CL41.Contig8_All, CL4362.Contig1_All, CL4362.Contig2_All, CL440.Contig3_All, CL440.Contig5_All, CL440.Contig6_All, CL4541.Contig1_All, CL4541.Contig2_All, CL495.Contig11_All, CL495.Contig1_All, CL495.Contig3_All, CL495.Contig4_All, CL495.Contig5_All, CL495.Contig6_All, CL531.Contig1_All, CL5391.Contig1_All, CL5391.Contig2_All, CL5399.Contig1_All, CL5399.Contig2_All, CL573.Contig6_All, CL703.Contig2_All, CL703.Contig3_All, CL703.Contig5_All, CL703.Contig6_All, CL878.Contig1_All, CL878.Contig2_All, CL878.Contig3_All, CL878.Contig4_All, CL878.Contig5_All, CL940.Contig3_All, CL940.Contig5_All, Unigene10058_All, Unigene10594_All, Unigene11086_All, Unigene11480_All, Unigene11482_All, Unigene11483_All, Unigene13225_All, Unigene13978_All, Unigene14005_All, Unigene18898_All, Unigene2029_All, Unigene2209_All, Unigene2274_All, Unigene2763_All, Unigene3010_All, Unigene3177_All, Unigene3779_All, Unigene4053_All, Unigene4070_All, Unigene4112_All, Unigene4323_All, Unigene4532_All, Unigene5716_All, Unigene5724_All, Unigene6215_All, Unigene62_All, Unigene6597_All, Unigene7206_All, Unigene7751_All, Unigene8422_All, Unigene8700_All, Unigene9437_All, Unigene9733_All

22	Calcium signaling pathway	CL1015.Contig11_All, CL1015.Contig27_All, CL1015.Contig9_All, CL1068.Contig5_All, CL1087.Contig2_All, CL1087.Contig3_All, CL1286.Contig2_All, CL1385.Contig1_All, CL1385.Contig2_All, CL1452.Contig1_All, CL1525.Contig14_All, CL1525.Contig15_All, CL1525.Contig9_All, CL1548.Contig2_All, CL1548.Contig3_All, CL1764.Contig2_All, CL1823.Contig8_All, CL187.Contig3_All, CL1880.Contig1_All, CL1880.Contig2_All, CL1880.Contig3_All, CL1887.Contig2_All, CL191.Contig1_All, CL191.Contig2_All, CL1920.Contig1_All, CL1920.Contig2_All, CL1991.Contig2_All, CL1991.Contig3_All, CL1991.Contig4_All, CL2039.Contig1_All, CL2061.Contig1_All, CL2131.Contig4_All, CL2131.Contig5_All, CL2131.Contig6_All, CL2131.Contig8_All, CL2182.Contig1_All, CL2182.Contig3_All, CL2182.Contig4_All, CL2468.Contig1_All, CL2468.Contig2_All, CL260.Contig15_All, CL270.Contig1_All, CL270.Contig2_All, CL2734.Contig1_All, CL2762.Contig4_All, CL2835.Contig1_All, CL2835.Contig2_All, CL2835.Contig3_All, CL2835.Contig4_All, CL2930.Contig5_All, CL2944.Contig1_All, CL2944.Contig2_All, CL305.Contig2_All, CL3136.Contig1_All, CL336.Contig1_All, CL336.Contig2_All, CL3500.Contig1_All, CL3525.Contig1_All, CL3525.Contig2_All, CL3545.Contig2_All, CL3667.Contig1_All, CL3667.Contig2_All, CL3667.Contig3_All, CL39.Contig1_All, CL39.Contig2_All, CL39.Contig3_All, CL39.Contig4_All, CL392.Contig1_All, CL392.Contig2_All, CL392.Contig3_All, CL3948.Contig1_All, CL3979.Contig1_All, CL3979.Contig2_All, CL4004.Contig1_All, CL4004.Contig2_All, CL4005.Contig2_All, CL4005.Contig3_All, CL4084.Contig1_All, CL4084.Contig2_All, CL4105.Contig2_All, CL4216.Contig1_All, CL4216.Contig2_All, CL4262.Contig2_All, CL4395.Contig1_All, CL44.Contig10_All, CL44.Contig12_All, CL44.Contig13_All, CL44.Contig16_All, CL44.Contig17_All, CL44.Contig18_All, CL44.Contig19_All, CL44.Contig1_All, CL44.Contig22_All, CL44.Contig23_All, CL44.Contig2_All, CL44.Contig3_All, CL44.Contig4_All, CL44.Contig5_All, CL44.Contig6_All, CL44.Contig8_All, CL44.Contig9_All, CL440.Contig3_All, CL440.Contig5_All, CL440.Contig6_All, CL4402.Contig1_All, CL4509.Contig1_All, CL4509.Contig2_All, CL4521.Contig1_All, CL4521.Contig2_All, CL4521.Contig3_All, CL4521.Contig5_All, CL4533.Contig1_All, CL4637.Contig1_All, CL467.Contig2_All, CL475.Contig4_All, CL4862.Contig1_All, CL5061.Contig2_All, CL5064.Contig2_All, CL5098.Contig2_All, CL5246.Contig3_All, CL531.Contig1_All, CL5310.Contig2_All, CL5391.Contig1_All, CL5391.Contig2_All, CL5399.Contig1_All, CL5399.Contig2_All, CL5458.Contig7_All, CL571.Contig2_All, CL573.Contig6_All, CL578.Contig2_All, CL578.Contig3_All, CL600.Contig1_All, CL600.Contig2_All, CL600.Contig3_All, CL600.Contig4_All, CL600.Contig6_All, CL623.Contig1_All, CL623.Contig3_All, CL623.Contig4_All, CL623.Contig5_All, CL623.Contig6_All, CL662.Contig1_All, CL688.Contig2_All, CL691.Contig1_All, CL691.Contig5_All, CL703.Contig2_All, CL703.Contig3_All, CL703.Contig5_All, CL703.Contig6_All, CL728.Contig1_All, CL772.Contig1_All, CL772.Contig2_All, CL878.Contig1_All, CL878.Contig2_All, CL878.Contig3_All, CL878.Contig4_All, CL878.Contig5_All, CL916.Contig2_All, CL940.Contig3_All, CL940.Contig5_All, CL970.Contig2_All, Unigene10006_All, Unigene10040_All, Unigene10058_All, Unigene10471_All, Unigene10574_All, Unigene10817_All, Unigene1099_All, Unigene11054_All, Unigene1137_All, Unigene11422_All, Unigene11446_All, Unigene11860_All, Unigene1186_All, Unigene12315_All, Unigene12331_All, Unigene1257_All, Unigene12719_All, Unigene13853_All, Unigene1417_All, Unigene1459_All, Unigene15194_All, Unigene15195_All, Unigene15855_All, Unigene16098_All, Unigene16538_All, Unigene1671_All, Unigene16769_All, Unigene16994_All, Unigene17478_All, Unigene17674_All, Unigene17675_All, Unigene17812_All, Unigene17815_All, Unigene17987_All, Unigene18178_All, Unigene18199_All, Unigene18276_All, Unigene18284_All, Unigene18898_All, Unigene18993_All, Unigene19116_All, Unigene19288_All, Unigene1974_All, Unigene20111_All, Unigene2032_All, Unigene2058_All, Unigene21008_All, Unigene2145_All, Unigene214_All, Unigene2172_All, Unigene217_All, Unigene2209_All, Unigene24_All, Unigene2763_All, Unigene3126_All, Unigene3471_All, Unigene3779_All, Unigene3786_All, Unigene3967_All, Unigene3996_All, Unigene4013_All, Unigene4053_All, Unigene4070_All, Unigene4092_All, Unigene4112_All, Unigene4532_All, Unigene5088_All, Unigene5675_All, Unigene5681_All, Unigene5821_All, Unigene5993_All, Unigene6059_All, Unigene6073_All, Unigene6242_All, Unigene6525_All, Unigene661_All, Unigene6640_All, Unigene699_All, Unigene7042_All, Unigene7993_All, Unigene8017_All, Unigene8019_All, Unigene807_All, Unigene8210_All, Unigene8422_All, Unigene8605_All, Unigene8615_All, Unigene8621_All, Unigene8700_All, Unigene8770_All, Unigene9014_All, Unigene9059_All, Unigene93_All, Unigene9437_All, Unigene9733_All, Unigene9805_All, Unigene9836_All, Unigene9921_All, Unigene9989_All

23	Vibrio cholerae infection	CL1062.Contig1_All, CL1079.Contig4_All, CL1116.Contig2_All, CL1355.Contig1_All, CL1370.Contig3_All, CL1452.Contig1_All, CL156.Contig1_All, CL156.Contig3_All, CL156.Contig4_All, CL1613.Contig1_All, CL1803.Contig1_All, CL2105.Contig1_All, CL2150.Contig3_All, CL2182.Contig1_All, CL2182.Contig3_All, CL2182.Contig4_All, CL2293.Contig1_All, CL2328.Contig1_All, CL2424.Contig3_All, CL2478.Contig2_All, CL2481.Contig1_All, CL2496.Contig1_All, CL2634.Contig1_All, CL2634.Contig2_All, CL2698.Contig2_All, CL2757.Contig2_All, CL2979.Contig1_All, CL3022.Contig2_All, CL3091.Contig1_All, CL3091.Contig2_All, CL3091.Contig3_All, CL3091.Contig4_All, CL3145.Contig2_All, CL3243.Contig1_All, CL3243.Contig2_All, CL3244.Contig1_All, CL3248.Contig2_All, CL326.Contig1_All, CL3442.Contig1_All, CL3487.Contig6_All, CL3690.Contig1_All, CL387.Contig2_All, CL3884.Contig2_All, CL39.Contig1_All, CL39.Contig2_All, CL39.Contig3_All, CL39.Contig4_All, CL3979.Contig1_All, CL3979.Contig2_All, CL4090.Contig2_All, CL4193.Contig1_All, CL4399.Contig3_All, CL440.Contig3_All, CL440.Contig5_All, CL440.Contig6_All, CL4459.Contig1_All, CL4491.Contig1_All, CL4666.Contig1_All, CL4666.Contig2_All, CL4879.Contig1_All, CL4891.Contig1_All, CL4891.Contig2_All, CL5034.Contig1_All, CL5034.Contig2_All, CL5280.Contig1_All, CL5391.Contig1_All, CL5391.Contig2_All, CL5468.Contig3_All, CL598.Contig1_All, CL598.Contig2_All, CL626.Contig1_All, CL626.Contig2_All, CL626.Contig3_All, CL626.Contig4_All, CL626.Contig6_All, CL847.Contig1_All, CL940.Contig3_All, CL940.Contig5_All, Unigene10026_All, Unigene10058_All, Unigene1024_All, Unigene1026_All, Unigene10433_All, Unigene10435_All, Unigene10483_All, Unigene11093_All, Unigene11094_All, Unigene11240_All, Unigene11539_All, Unigene11557_All, Unigene11578_All, Unigene11597_All, Unigene11598_All, Unigene11599_All, Unigene11880_All, Unigene11884_All, Unigene11899_All, Unigene1189_All, Unigene11908_All, Unigene12016_All, Unigene12017_All, Unigene12028_All, Unigene12168_All, Unigene12209_All, Unigene12275_All, Unigene12302_All, Unigene12315_All, Unigene12357_All, Unigene12423_All, Unigene125_All, Unigene12684_All, Unigene12793_All, Unigene12923_All, Unigene13092_All, Unigene1322_All, Unigene13324_All, Unigene13508_All, Unigene13589_All, Unigene1488_All, Unigene15320_All, Unigene15814_All, Unigene15815_All, Unigene15824_All, Unigene15969_All, Unigene16558_All, Unigene16580_All, Unigene16659_All, Unigene1704_All, Unigene17111_All, Unigene17327_All, Unigene1741_All, Unigene17596_All, Unigene17602_All, Unigene17727_All, Unigene17731_All, Unigene17798_All, Unigene18063_All, Unigene18598_All, Unigene18707_All, Unigene18898_All, Unigene19303_All, Unigene19360_All, Unigene20128_All, Unigene20912_All, Unigene2105_All, Unigene2250_All, Unigene2741_All, Unigene278_All, Unigene3069_All, Unigene3157_All, Unigene3207_All, Unigene3351_All, Unigene3391_All, Unigene3476_All, Unigene3730_All, Unigene3779_All, Unigene4112_All, Unigene4133_All, Unigene4305_All, Unigene4444_All, Unigene4532_All, Unigene5086_All, Unigene5233_All, Unigene5265_All, Unigene5589_All, Unigene5913_All, Unigene6009_All, Unigene6050_All, Unigene6098_All, Unigene6145_All, Unigene6305_All, Unigene6326_All, Unigene646_All, Unigene6478_All, Unigene65_All, Unigene6741_All, Unigene7284_All, Unigene7723_All, Unigene8109_All, Unigene8208_All, Unigene8627_All, Unigene9040_All, Unigene9161_All, Unigene925_All, Unigene9271_All, Unigene9513_All, Unigene955_All, Unigene9733_All
24	Ribosome biogenesis in eukaryotes	CL1554.Contig2_All, CL1699.Contig4_All, CL1714.Contig4_All, CL1725.Contig1_All, CL1725.Contig2_All, CL1904.Contig1_All, CL1904.Contig2_All, CL2077.Contig2_All, CL2077.Contig3_All, CL2077.Contig4_All, CL2112.Contig7_All, CL2144.Contig1_All, CL2144.Contig2_All, CL2248.Contig2_All, CL2285.Contig1_All, CL2285.Contig2_All, CL2671.Contig2_All, CL2765.Contig1_All, CL2765.Contig2_All, CL2827.Contig4_All, CL2838.Contig1_All, CL2838.Contig2_All, CL2838.Contig4_All, CL2933.Contig1_All, CL2933.Contig2_All, CL2933.Contig3_All, CL2984.Contig1_All, CL2986.Contig1_All, CL2986.Contig2_All, CL3332.Contig2_All, CL3668.Contig1_All, CL3668.Contig2_All, CL3705.Contig1_All, CL3705.Contig2_All, CL3738.Contig1_All, CL3776.Contig2_All, CL3776.Contig3_All, CL3776.Contig4_All, CL378.Contig1_All, CL3838.Contig1_All, CL3838.Contig2_All, CL4160.Contig1_All, CL4394.Contig2_All, CL446.Contig2_All, CL4633.Contig1_All, CL4633.Contig2_All, CL4775.Contig1_All, CL4775.Contig2_All, CL4874.Contig1_All, CL5437.Contig1_All, CL5437.Contig2_All, CL560.Contig1_All, CL560.Contig2_All, CL560.Contig4_All, CL563.Contig1_All, CL563.Contig2_All, CL563.Contig4_All, CL775.Contig1_All, CL775.Contig2_All, CL830.Contig3_All, CL916.Contig2_All, Unigene10143_All, Unigene10144_All, Unigene101_All, Unigene10307_All, Unigene10598_All, Unigene10619_All, Unigene1065_All, Unigene10851_All, Unigene10859_All, Unigene11044_All, Unigene11145_All, Unigene11169_All, Unigene11170_All, Unigene11523_All, Unigene11791_All, Unigene11827_All, Unigene12010_All, Unigene12249_All, Unigene12265_All, Unigene12410_All, Unigene12440_All, Unigene12562_All, Unigene12586_All, Unigene12591_All, Unigene12608_All, Unigene12695_All, Unigene12706_All, Unigene12780_All, Unigene12806_All, Unigene13060_All, Unigene13125_All, Unigene13134_All, Unigene13201_All, Unigene13387_All, Unigene13541_All, Unigene13562_All, Unigene13904_All, Unigene14012_All, Unigene16235_All, Unigene165_All, Unigene16_All, Unigene1752_All, Unigene18186_All, Unigene18319_All, Unigene18714_All, Unigene1957_All, Unigene20319_All, Unigene2116_All, Unigene2552_All, Unigene2867_All, Unigene2931_All, Unigene3016_All, Unigene3299_All, Unigene3415_All, Unigene3634_All, Unigene4157_All, Unigene4180_All, Unigene4432_All, Unigene4683_All, Unigene4786_All, Unigene4846_All, Unigene5224_All, Unigene5362_All, Unigene5650_All, Unigene6183_All, Unigene6525_All, Unigene6776_All, Unigene6999_All, Unigene7148_All, Unigene7200_All, Unigene7245_All, Unigene7679_All, Unigene780_All, Unigene783_All, Unigene829_All, Unigene8499_All, Unigene9030_All, Unigene9259_All, Unigene9362_All, Unigene9829_All

25	Vascular smooth muscle contraction	CL1068.Contig5_All, CL1075.Contig3_All, CL1141.Contig2_All, CL126.Contig2_All, CL1286.Contig2_All, CL1452.Contig1_All, CL147.Contig6_All, CL1525.Contig15_All, CL1525.Contig9_All, CL1559.Contig1_All, CL1563.Contig2_All, CL1687.Contig4_All, CL1823.Contig8_All, CL187.Contig3_All, CL191.Contig1_All, CL191.Contig2_All, CL1920.Contig1_All, CL1920.Contig2_All, CL1987.Contig1_All, CL199.Contig1_All, CL2039.Contig1_All, CL2061.Contig1_All, CL2131.Contig4_All, CL2131.Contig5_All, CL2131.Contig6_All, CL2131.Contig8_All, CL2182.Contig1_All, CL2182.Contig3_All, CL2182.Contig4_All, CL2224.Contig2_All, CL2224.Contig3_All, CL2224.Contig4_All, CL2265.Contig1_All, CL2265.Contig2_All, CL2432.Contig2_All, CL244.Contig2_All, CL2468.Contig1_All, CL2468.Contig2_All, CL2762.Contig4_All, CL2904.Contig1_All, CL2904.Contig2_All, CL2930.Contig5_All, CL296.Contig16_All, CL3011.Contig1_All, CL3046.Contig1_All, CL3047.Contig1_All, CL3047.Contig2_All, CL305.Contig2_All, CL3092.Contig1_All, CL3092.Contig2_All, CL3224.Contig1_All, CL3260.Contig1_All, CL3260.Contig2_All, CL3260.Contig3_All, CL3260.Contig4_All, CL3260.Contig5_All, CL3260.Contig7_All, CL3294.Contig1_All, CL3295.Contig1_All, CL3297.Contig1_All, CL3297.Contig2_All, CL3311.Contig1_All, CL3311.Contig2_All, CL3347.Contig1_All, CL3347.Contig2_All, CL3500.Contig1_All, CL3525.Contig1_All, CL3525.Contig2_All, CL3543.Contig2_All, CL3543.Contig3_All, CL3543.Contig4_All, CL3667.Contig1_All, CL3667.Contig2_All, CL3667.Contig3_All, CL3672.Contig4_All, CL3689.Contig2_All, CL3761.Contig1_All, CL3823.Contig1_All, CL3823.Contig2_All, CL39.Contig1_All, CL39.Contig2_All, CL39.Contig3_All, CL39.Contig4_All, CL392.Contig1_All, CL392.Contig2_All, CL392.Contig3_All, CL4002.Contig1_All, CL4004.Contig1_All, CL4004.Contig2_All, CL4005.Contig2_All, CL4005.Contig3_All, CL4014.Contig1_All, CL4180.Contig3_All, CL4181.Contig1_All, CL4181.Contig4_All, CL4187.Contig1_All, CL4216.Contig1_All, CL4216.Contig2_All, CL4250.Contig2_All, CL4262.Contig2_All, CL4340.Contig1_All, CL4362.Contig1_All, CL4362.Contig2_All, CL4395.Contig1_All, CL440.Contig3_All, CL440.Contig5_All, CL440.Contig6_All, CL4402.Contig1_All, CL4442.Contig1_All, CL4454.Contig3_All, CL4509.Contig1_All, CL4509.Contig2_All, CL4596.Contig1_All, CL4637.Contig1_All, CL467.Contig2_All, CL4787.Contig1_All, CL4855.Contig1_All, CL4855.Contig2_All, CL4862.Contig1_All, CL4904.Contig1_All, CL503.Contig2_All, CL503.Contig3_All, CL503.Contig4_All, CL503.Contig5_All, CL503.Contig6_All, CL503.Contig8_All, CL5061.Contig2_All, CL52.Contig17_All, CL52.Contig28_All, CL52.Contig35_All, CL52.Contig3_All, CL52.Contig41_All, CL523.Contig1_All, CL523.Contig2_All, CL5246.Contig3_All, CL5310.Contig2_All, CL5391.Contig1_All, CL5391.Contig2_All, CL5399.Contig1_All, CL5399.Contig2_All, CL5458.Contig7_All, CL571.Contig2_All, CL573.Contig1_All, CL644.Contig1_All, CL648.Contig2_All, CL691.Contig1_All, CL741.Contig2_All, CL772.Contig1_All, CL772.Contig2_All, CL787.Contig5_All, CL860.Contig10_All, CL860.Contig8_All, CL860.Contig9_All, CL916.Contig2_All, CL923.Contig1_All, CL923.Contig2_All, CL923.Contig3_All, CL940.Contig3_All, CL940.Contig5_All, CL970.Contig2_All, Unigene10006_All, Unigene10058_All, Unigene10201_All, Unigene10202_All, Unigene10574_All, Unigene10682_All, Unigene10777_All, Unigene11054_All, Unigene11289_All, Unigene11298_All, Unigene11300_All, Unigene11307_All, Unigene1131_All, Unigene1137_All, Unigene1168_All, Unigene11730_All, Unigene11935_All, Unigene12315_All, Unigene12418_All, Unigene1257_All, Unigene12658_All, Unigene12697_All, Unigene12719_All, Unigene12828_All, Unigene1512_All, Unigene15176_All, Unigene15271_All, Unigene16033_All, Unigene16098_All, Unigene1633_All, Unigene16435_All, Unigene16510_All, Unigene16716_All, Unigene1671_All, Unigene16769_All, Unigene16860_All, Unigene16874_All, Unigene16976_All, Unigene16994_All, Unigene1705_All, Unigene17117_All, Unigene17301_All, Unigene17362_All, Unigene17488_All, Unigene17773_All, Unigene1777_All, Unigene17815_All, Unigene17824_All, Unigene18006_All, Unigene18037_All, Unigene18106_All, Unigene18178_All, Unigene18200_All, Unigene18215_All, Unigene18276_All, Unigene18317_All, Unigene18378_All, Unigene18644_All, Unigene18645_All, Unigene18898_All, Unigene18993_All, Unigene19289_All, Unigene19364_All, Unigene1941_All, Unigene19453_All, Unigene19647_All, Unigene19745_All, Unigene1974_All, Unigene20065_All, Unigene20111_All, Unigene2015_All, Unigene2019_All, Unigene20299_All, Unigene2029_All, Unigene2032_All, Unigene2052_All, Unigene20530_All, Unigene2053_All, Unigene2058_All, Unigene20625_All, Unigene21008_All, Unigene210_All, Unigene2145_All, Unigene214_All, Unigene217_All, Unigene2209_All, Unigene2233_All, Unigene237_All, Unigene24_All, Unigene2514_All, Unigene2519_All, Unigene2795_All, Unigene2817_All, Unigene3029_All, Unigene3033_All, Unigene3034_All, Unigene3036_All, Unigene3037_All, Unigene3042_All, Unigene3328_All, Unigene3471_All, Unigene3659_All, Unigene3696_All, Unigene3779_All, Unigene3861_All, Unigene3996_All, Unigene4013_All, Unigene4022_All, Unigene4053_All, Unigene4070_All, Unigene4112_All, Unigene4161_All, Unigene4221_All, Unigene4269_All, Unigene4532_All, Unigene4692_All, Unigene4783_All, Unigene4920_All, Unigene4935_All, Unigene4970_All, Unigene500_All, Unigene5035_All, Unigene5088_All, Unigene5675_All, Unigene5808_All, Unigene5821_All, Unigene5993_All, Unigene5994_All, Unigene6013_All, Unigene6015_All, Unigene6037_All, Unigene6059_All, Unigene6073_All, Unigene6273_All, Unigene6459_All, Unigene6525_All, Unigene6655_All, Unigene6896_All, Unigene6907_All, Unigene7063_All, Unigene7129_All, Unigene7183_All, Unigene7217_All, Unigene7704_All, Unigene7759_All, Unigene7829_All, Unigene7854_All, Unigene785_All, Unigene7993_All, Unigene8009_All, Unigene8017_All, Unigene8056_All, Unigene8160_All, Unigene8210_All, Unigene8422_All, Unigene8558_All, Unigene8615_All, Unigene8621_All, Unigene8747_All, Unigene9014_All, Unigene9039_All, Unigene9050_All, Unigene9059_All, Unigene9076_All, Unigene9222_All, Unigene93_All, Unigene9437_All, Unigene9722_All, Unigene9733_All, Unigene9836_All, Unigene9877_All, Unigene9904_All
26	African trypanosomiasis	CL2182.Contig1_All, CL2182.Contig3_All, CL2182.Contig4_All, CL2930.Contig5_All, CL39.Contig1_All, CL39.Contig2_All, CL39.Contig3_All, CL39.Contig4_All, CL5391.Contig1_All, CL5391.Contig2_All, CL5399.Contig1_All, CL5399.Contig2_All, CL940.Contig3_All, CL940.Contig5_All, Unigene10999_All, Unigene11371_All, Unigene18898_All, Unigene2209_All, Unigene4053_All, Unigene4070_All, Unigene8422_All, Unigene9437_All

27	GnRH signaling pathway	CL1085.Contig2_All, CL1286.Contig2_All, CL1452.Contig1_All, CL1548.Contig2_All, CL1548.Contig3_All, CL1807.Contig1_All, CL1807.Contig2_All, CL1823.Contig8_All, CL1966.Contig2_All, CL199.Contig1_All, CL2129.Contig4_All, CL2131.Contig4_All, CL2131.Contig5_All, CL2131.Contig6_All, CL2131.Contig8_All, CL2182.Contig1_All, CL2182.Contig3_All, CL2182.Contig4_All, CL2432.Contig2_All, CL2468.Contig1_All, CL2468.Contig2_All, CL271.Contig2_All, CL2930.Contig5_All, CL3007.Contig2_All, CL3047.Contig1_All, CL3047.Contig2_All, CL3667.Contig1_All, CL3667.Contig2_All, CL3667.Contig3_All, CL39.Contig1_All, CL39.Contig2_All, CL39.Contig3_All, CL39.Contig4_All, CL398.Contig4_All, CL400.Contig1_All, CL400.Contig2_All, CL4005.Contig2_All, CL4005.Contig3_All, CL4262.Contig2_All, CL4395.Contig1_All, CL44.Contig10_All, CL44.Contig12_All, CL44.Contig13_All, CL44.Contig16_All, CL44.Contig17_All, CL44.Contig18_All, CL44.Contig19_All, CL44.Contig1_All, CL44.Contig22_All, CL44.Contig23_All, CL44.Contig2_All, CL44.Contig3_All, CL44.Contig4_All, CL44.Contig5_All, CL44.Contig6_All, CL44.Contig8_All, CL44.Contig9_All, CL440.Contig3_All, CL440.Contig5_All, CL440.Contig6_All, CL4439.Contig2_All, CL4509.Contig1_All, CL4509.Contig2_All, CL4736.Contig1_All, CL4736.Contig2_All, CL4787.Contig1_All, CL4815.Contig1_All, CL4815.Contig2_All, CL4855.Contig1_All, CL4855.Contig2_All, CL4966.Contig1_All, CL5115.Contig1_All, CL5310.Contig2_All, CL5391.Contig1_All, CL5391.Contig2_All, CL5399.Contig1_All, CL5399.Contig2_All, CL5458.Contig5_All, CL5458.Contig7_All, CL691.Contig1_All, CL772.Contig1_All, CL772.Contig2_All, CL860.Contig10_All, CL860.Contig8_All, CL860.Contig9_All, CL940.Contig3_All, CL940.Contig5_All, Unigene10006_All, Unigene10058_All, Unigene10174_All, Unigene10574_All, Unigene11054_All, Unigene12139_All, Unigene12315_All, Unigene12671_All, Unigene12719_All, Unigene1326_All, Unigene1479_All, Unigene16769_All, Unigene18898_All, Unigene18993_All, Unigene2029_All, Unigene2032_All, Unigene2209_All, Unigene220_All, Unigene3029_All, Unigene3033_All, Unigene3034_All, Unigene3036_All, Unigene3037_All, Unigene3042_All, Unigene3779_All, Unigene3996_All, Unigene4013_All, Unigene4053_All, Unigene4070_All, Unigene4112_All, Unigene4532_All, Unigene5675_All, Unigene6101_All, Unigene6977_All, Unigene699_All, Unigene7018_All, Unigene7265_All, Unigene8046_All, Unigene8056_All, Unigene8205_All, Unigene8422_All, Unigene8621_All, Unigene9028_All, Unigene93_All, Unigene9437_All, Unigene9733_All
28	Aminoacyl-tRNA biosynthesis	CL1571.Contig2_All, CL1585.Contig5_All, CL1585.Contig6_All, CL1834.Contig1_All, CL2038.Contig1_All, CL2038.Contig7_All, CL2539.Contig1_All, CL2539.Contig2_All, CL289.Contig1_All, CL289.Contig3_All, CL2898.Contig1_All, CL2898.Contig2_All, CL2898.Contig3_All, CL3381.Contig1_All, CL3381.Contig2_All, CL4378.Contig2_All, CL4424.Contig1_All, CL4424.Contig2_All, CL4424.Contig3_All, CL4424.Contig4_All, CL5013.Contig3_All, CL5013.Contig4_All, CL505.Contig1_All, CL505.Contig2_All, CL628.Contig1_All, Unigene10131_All, Unigene10150_All, Unigene10151_All, Unigene10351_All, Unigene10425_All, Unigene10554_All, Unigene11234_All, Unigene11235_All, Unigene11236_All, Unigene11954_All, Unigene12290_All, Unigene12355_All, Unigene12439_All, Unigene12756_All, Unigene12924_All, Unigene1293_All, Unigene13310_All, Unigene13437_All, Unigene13506_All, Unigene14161_All, Unigene1513_All, Unigene15573_All, Unigene1779_All, Unigene1811_All, Unigene2410_All, Unigene3180_All, Unigene3188_All, Unigene3254_All, Unigene3387_All, Unigene4045_All, Unigene4904_All, Unigene5382_All, Unigene5984_All, Unigene6127_All, Unigene6205_All, Unigene6228_All, Unigene6787_All, Unigene6944_All, Unigene739_All, Unigene8039_All, Unigene8198_All, Unigene8652_All, Unigene9092_All, Unigene9219_All, Unigene9653_All, Unigene9662_All, Unigene9741_All, Unigene9791_All
29	Cholinergic synapse	CL1066.Contig1_All, CL1066.Contig2_All, CL1066.Contig3_All, CL1452.Contig1_All, CL1675.Contig2_All, CL2182.Contig1_All, CL2182.Contig3_All, CL2182.Contig4_All, CL2450.Contig1_All, CL2450.Contig2_All, CL257.Contig1_All, CL257.Contig2_All, CL2930.Contig5_All, CL367.Contig1_All, CL367.Contig2_All, CL367.Contig3_All, CL39.Contig1_All, CL39.Contig2_All, CL39.Contig3_All, CL39.Contig4_All, CL3920.Contig2_All, CL4005.Contig2_All, CL4005.Contig3_All, CL4037.Contig1_All, CL4197.Contig1_All, CL4197.Contig2_All, CL4262.Contig2_All, CL44.Contig10_All, CL44.Contig12_All, CL44.Contig13_All, CL44.Contig16_All, CL44.Contig17_All, CL44.Contig18_All, CL44.Contig19_All, CL44.Contig1_All, CL44.Contig22_All, CL44.Contig23_All, CL44.Contig2_All, CL44.Contig3_All, CL44.Contig4_All, CL44.Contig5_All, CL44.Contig6_All, CL44.Contig8_All, CL44.Contig9_All, CL440.Contig3_All, CL440.Contig5_All, CL440.Contig6_All, CL4439.Contig2_All, CL4787.Contig1_All, CL4923.Contig2_All, CL5122.Contig1_All, CL5122.Contig2_All, CL5125.Contig1_All, CL5391.Contig1_All, CL5391.Contig2_All, CL5399.Contig1_All, CL5399.Contig2_All, CL5441.Contig3_All, CL600.Contig1_All, CL600.Contig2_All, CL600.Contig3_All, CL600.Contig4_All, CL600.Contig6_All, CL691.Contig1_All, CL695.Contig16_All, CL940.Contig3_All, CL940.Contig5_All, Unigene12110_All, Unigene12671_All, Unigene15194_All, Unigene15195_All, Unigene16561_All, Unigene17025_All, Unigene1743_All, Unigene176_All, Unigene17968_All, Unigene18199_All, Unigene18898_All, Unigene18993_All, Unigene1925_All, Unigene2029_All, Unigene2032_All, Unigene2141_All, Unigene2196_All, Unigene2209_All, Unigene2749_All, Unigene2760_All, Unigene3357_All, Unigene3779_All, Unigene4053_All, Unigene4070_All, Unigene4096_All, Unigene4112_All, Unigene4126_All, Unigene4780_All, Unigene5521_All, Unigene6217_All, Unigene69_All, Unigene7023_All, Unigene7042_All, Unigene8422_All, Unigene8621_All, Unigene8643_All, Unigene8727_All, Unigene8770_All, Unigene93_All, Unigene9437_All, Unigene9644_All, Unigene9733_All

30	Chemokine signaling pathway	CL1066.Contig1_All, CL1066.Contig2_All, CL1066.Contig3_All, CL1104.Contig1_All, CL1104.Contig2_All, CL1104.Contig3_All, CL1104.Contig4_All, CL1104.Contig5_All, CL1104.Contig6_All, CL1104.Contig7_All, CL1104.Contig8_All, CL1104.Contig9_All, CL1452.Contig1_All, CL1535.Contig3_All, CL1546.Contig1_All, CL1546.Contig3_All, CL1546.Contig4_All, CL1546.Contig5_All, CL1546.Contig6_All, CL1546.Contig7_All, CL1546.Contig8_All, CL1675.Contig2_All, CL1723.Contig1_All, CL1723.Contig2_All, CL1723.Contig7_All, CL1807.Contig1_All, CL1807.Contig2_All, CL1843.Contig3_All, CL1874.Contig2_All, CL1954.Contig1_All, CL1954.Contig2_All, CL2038.Contig1_All, CL2038.Contig7_All, CL2066.Contig1_All, CL2182.Contig1_All, CL2182.Contig3_All, CL2182.Contig4_All, CL2224.Contig2_All, CL2224.Contig3_All, CL2224.Contig4_All, CL2375.Contig2_All, CL2642.Contig1_All, CL2642.Contig2_All, CL271.Contig2_All, CL2930.Contig5_All, CL2975.Contig2_All, CL2992.Contig1_All, CL3027.Contig3_All, CL3047.Contig1_All, CL3047.Contig2_All, CL3116.Contig2_All, CL3632.Contig2_All, CL3632.Contig3_All, CL367.Contig1_All, CL367.Contig2_All, CL367.Contig3_All, CL3823.Contig1_All, CL3823.Contig2_All, CL39.Contig1_All, CL39.Contig2_All, CL39.Contig3_All, CL39.Contig4_All, CL4005.Contig2_All, CL4005.Contig3_All, CL4040.Contig1_All, CL4088.Contig2_All, CL4130.Contig1_All, CL4158.Contig4_All, CL4158.Contig5_All, CL4158.Contig7_All, CL4262.Contig2_All, CL440.Contig3_All, CL440.Contig5_All, CL440.Contig6_All, CL4439.Contig2_All, CL4559.Contig2_All, CL4559.Contig3_All, CL4787.Contig1_All, CL4855.Contig1_All, CL4855.Contig2_All, CL5361.Contig2_All, CL5361.Contig4_All, CL5391.Contig1_All, CL5391.Contig2_All, CL5469.Contig2_All, CL622.Contig1_All, CL622.Contig2_All, CL622.Contig4_All, CL695.Contig16_All, CL748.Contig1_All, CL748.Contig3_All, CL940.Contig3_All, CL940.Contig5_All, Unigene10097_All, Unigene10174_All, Unigene10998_All, Unigene11082_All, Unigene11307_All, Unigene11459_All, Unigene11521_All, Unigene11922_All, Unigene12110_All, Unigene12201_All, Unigene12603_All, Unigene12671_All, Unigene13036_All, Unigene1447_All, Unigene16840_All, Unigene16963_All, Unigene1757_All, Unigene1855_All, Unigene18644_All, Unigene18898_All, Unigene1925_All, Unigene19601_All, Unigene20226_All, Unigene2029_All, Unigene2110_All, Unigene236_All, Unigene247_All, Unigene2749_All, Unigene3088_All, Unigene3168_All, Unigene3779_All, Unigene3967_All, Unigene4053_All, Unigene4070_All, Unigene4112_All, Unigene4780_All, Unigene5303_All, Unigene5521_All, Unigene555_All, Unigene5808_All, Unigene5813_All, Unigene5873_All, Unigene6014_All, Unigene6029_All, Unigene6217_All, Unigene7016_All, Unigene7058_All, Unigene7063_All, Unigene7297_All, Unigene7547_All, Unigene8046_All, Unigene8056_All, Unigene8184_All, Unigene8422_All, Unigene8747_All, Unigene8784_All, Unigene9050_All, Unigene9379_All, Unigene93_All, Unigene9719_All, Unigene9730_All, Unigene9733_All, Unigene9785_All, Unigene9787_All, Unigene9877_All, Unigene9902_All
31	Antigen processing and presentation	CL105.Contig1_All, CL105.Contig2_All, CL105.Contig3_All, CL1054.Contig1_All, CL1054.Contig2_All, CL1280.Contig1_All, CL1280.Contig2_All, CL1358.Contig1_All, CL1358.Contig2_All, CL1358.Contig3_All, CL1774.Contig2_All, CL1776.Contig1_All, CL1925.Contig1_All, CL2165.Contig2_All, CL2362.Contig1_All, CL2362.Contig2_All, CL2686.Contig1_All, CL2686.Contig2_All, CL3302.Contig2_All, CL3706.Contig1_All, CL3742.Contig1_All, CL3920.Contig2_All, CL4057.Contig1_All, CL4247.Contig2_All, CL4247.Contig3_All, CL4576.Contig1_All, CL4633.Contig1_All, CL4633.Contig2_All, CL5043.Contig1_All, CL5043.Contig2_All, CL5135.Contig1_All, CL5135.Contig2_All, CL5256.Contig1_All, CL5256.Contig2_All, CL5256.Contig4_All, CL558.Contig1_All, CL558.Contig2_All, CL558.Contig4_All, CL702.Contig2_All, CL702.Contig3_All, CL785.Contig1_All, Unigene10162_All, Unigene10296_All, Unigene10374_All, Unigene10837_All, Unigene10977_All, Unigene11000_All, Unigene11053_All, Unigene11166_All, Unigene11241_All, Unigene11282_All, Unigene11360_All, Unigene11361_All, Unigene11845_All, Unigene11876_All, Unigene11879_All, Unigene12091_All, Unigene12393_All, Unigene12508_All, Unigene12713_All, Unigene12890_All, Unigene13331_All, Unigene15263_All, Unigene15265_All, Unigene15587_All, Unigene15863_All, Unigene16093_All, Unigene16098_All, Unigene16499_All, Unigene17470_All, Unigene17781_All, Unigene18095_All, Unigene18114_All, Unigene18195_All, Unigene18535_All, Unigene1922_All, Unigene20260_All, Unigene2074_All, Unigene2121_All, Unigene22023_All, Unigene2270_All, Unigene2961_All, Unigene2982_All, Unigene3830_All, Unigene4155_All, Unigene44_All, Unigene4921_All, Unigene5187_All, Unigene5923_All, Unigene5935_All, Unigene6915_All, Unigene860_All, Unigene8850_All, Unigene9141_All, Unigene9438_All, Unigene962_All, Unigene9928_All, Unigene9971_All

32	RNA transport	CL1046.Contig1_All, CL1046.Contig2_All, CL1046.Contig3_All, CL1401.Contig1_All, CL1477.Contig8_All, CL1637.Contig1_All, CL1818.Contig1_All, CL1818.Contig2_All, CL1818.Contig4_All, CL1818.Contig5_All, CL1818.Contig6_All, CL1850.Contig1_All, CL1850.Contig2_All, CL1867.Contig1_All, CL1867.Contig2_All, CL1927.Contig2_All, CL2144.Contig1_All, CL2144.Contig2_All, CL2154.Contig1_All, CL2154.Contig2_All, CL2187.Contig2_All, CL2229.Contig1_All, CL2244.Contig2_All, CL2278.Contig1_All, CL2278.Contig2_All, CL2496.Contig1_All, CL2502.Contig2_All, CL2502.Contig3_All, CL2719.Contig1_All, CL2719.Contig2_All, CL2765.Contig1_All, CL2765.Contig2_All, CL2827.Contig4_All, CL2959.Contig2_All, CL296.Contig4_All, CL297.Contig1_All, CL3057.Contig1_All, CL3057.Contig2_All, CL3139.Contig1_All, CL3186.Contig5_All, CL3221.Contig1_All, CL3221.Contig2_All, CL3224.Contig1_All, CL3270.Contig1_All, CL3270.Contig2_All, CL3278.Contig1_All, CL3401.Contig2_All, CL3444.Contig2_All, CL3444.Contig3_All, CL3616.Contig1_All, CL3635.Contig2_All, CL3725.Contig2_All, CL3811.Contig1_All, CL3811.Contig2_All, CL3811.Contig3_All, CL3811.Contig4_All, CL3837.Contig1_All, CL3837.Contig2_All, CL4101.Contig1_All, CL4134.Contig1_All, CL4155.Contig1_All, CL4234.Contig2_All, CL4298.Contig3_All, CL4298.Contig4_All, CL4321.Contig1_All, CL4331.Contig1_All, CL4331.Contig2_All, CL4375.Contig2_All, CL452.Contig2_All, CL4551.Contig1_All, CL4551.Contig2_All, CL4679.Contig1_All, CL4783.Contig2_All, CL4803.Contig1_All, CL4874.Contig1_All, CL4884.Contig2_All, CL4884.Contig4_All, CL497.Contig1_All, CL5067.Contig2_All, CL5078.Contig12_All, CL5107.Contig1_All, CL5107.Contig2_All, CL5116.Contig1_All, CL5150.Contig1_All, CL5150.Contig4_All, CL5214.Contig1_All, CL5214.Contig2_All, CL5319.Contig1_All, CL5355.Contig1_All, CL5405.Contig1_All, CL560.Contig1_All, CL560.Contig2_All, CL560.Contig4_All, CL700.Contig1_All, CL700.Contig2_All, CL846.Contig2_All, CL846.Contig5_All, CL877.Contig1_All, CL92.Contig3_All, CL926.Contig1_All, CL926.Contig2_All, Unigene10053_All, Unigene10307_All, Unigene10361_All, Unigene10528_All, Unigene1055_All, Unigene10586_All, Unigene10662_All, Unigene10875_All, Unigene11025_All, Unigene11092_All, Unigene11099_All, Unigene11460_All, Unigene11623_All, Unigene1172_All, Unigene11785_All, Unigene11807_All, Unigene11811_All, Unigene11869_All, Unigene11873_All, Unigene11929_All, Unigene11930_All, Unigene12006_All, Unigene12047_All, Unigene12124_All, Unigene12158_All, Unigene12191_All, Unigene12234_All, Unigene12249_All, Unigene12299_All, Unigene12322_All, Unigene12410_All, Unigene12414_All, Unigene12464_All, Unigene12498_All, Unigene12534_All, Unigene12582_All, Unigene12608_All, Unigene1260_All, Unigene12618_All, Unigene12669_All, Unigene12782_All, Unigene12838_All, Unigene12866_All, Unigene12868_All, Unigene12894_All, Unigene12949_All, Unigene13010_All, Unigene13073_All, Unigene1313_All, Unigene13153_All, Unigene13265_All, Unigene13507_All, Unigene13527_All, Unigene13541_All, Unigene13944_All, Unigene1400_All, Unigene14100_All, Unigene15302_All, Unigene15303_All, Unigene16279_All, Unigene1651_All, Unigene16820_All, Unigene17244_All, Unigene1730_All, Unigene17486_All, Unigene17487_All, Unigene1768_All, Unigene1775_All, Unigene17856_All, Unigene18149_All, Unigene18867_All, Unigene19870_All, Unigene19925_All, Unigene19947_All, Unigene20230_All, Unigene2075_All, Unigene2203_All, Unigene2255_All, Unigene246_All, Unigene2691_All, Unigene2780_All, Unigene2783_All, Unigene2891_All, Unigene3415_All, Unigene3712_All, Unigene3991_All, Unigene407_All, Unigene4161_All, Unigene4251_All, Unigene4593_All, Unigene4662_All, Unigene4749_All, Unigene4764_All, Unigene4771_All, Unigene4781_All, Unigene4786_All, Unigene4800_All, Unigene4944_All, Unigene4945_All, Unigene4954_All, Unigene5055_All, Unigene5067_All, Unigene5079_All, Unigene5092_All, Unigene5264_All, Unigene5361_All, Unigene5362_All, Unigene5789_All, Unigene6009_All, Unigene6014_All, Unigene6236_All, Unigene6303_All, Unigene6681_All, Unigene6704_All, Unigene6976_All, Unigene7270_All, Unigene7646_All, Unigene764_All, Unigene7740_All, Unigene7764_All, Unigene7776_All, Unigene780_All, Unigene7981_All, Unigene8003_All, Unigene8119_All, Unigene8152_All, Unigene8228_All, Unigene8235_All, Unigene8692_All, Unigene8720_All, Unigene9019_All, Unigene9039_All, Unigene9252_All, Unigene9710_All, Unigene9742_All, Unigene9893_All, Unigene9895_All
33	Herpes simplex infection	CL1085.Contig3_All, CL1188.Contig1_All, CL1188.Contig2_All, CL1470.Contig1_All, CL1470.Contig2_All, CL1563.Contig2_All, CL1687.Contig4_All, CL1812.Contig1_All, CL1812.Contig2_All, CL2049.Contig1_All, CL2049.Contig3_All, CL2170.Contig2_All, CL2170.Contig5_All, CL2222.Contig9_All, CL2306.Contig2_All, CL244.Contig2_All, CL2480.Contig2_All, CL2480.Contig3_All, CL2789.Contig2_All, CL2848.Contig4_All, CL2882.Contig1_All, CL2882.Contig2_All, CL3057.Contig1_All, CL3057.Contig2_All, CL3420.Contig1_All, CL345.Contig1_All, CL3547.Contig1_All, CL3547.Contig2_All, CL3547.Contig4_All, CL3561.Contig1_All, CL3561.Contig2_All, CL3570.Contig1_All, CL3653.Contig1_All, CL3664.Contig1_All, CL3664.Contig2_All, CL3689.Contig2_All, CL3787.Contig1_All, CL3787.Contig2_All, CL3955.Contig1_All, CL3963.Contig1_All, CL4002.Contig1_All, CL4150.Contig4_All, CL4375.Contig2_All, CL4494.Contig1_All, CL4494.Contig2_All, CL4494.Contig3_All, CL4514.Contig2_All, CL4555.Contig1_All, CL4679.Contig1_All, CL4679.Contig2_All, CL4736.Contig1_All, CL4736.Contig2_All, CL5115.Contig1_All, CL560.Contig1_All, CL560.Contig2_All, CL560.Contig4_All, CL598.Contig1_All, CL598.Contig2_All, CL741.Contig2_All, Unigene1048_All, Unigene10619_All, Unigene10757_All, Unigene11040_All, Unigene11067_All, Unigene11068_All, Unigene11156_All, Unigene11157_All, Unigene1137_All, Unigene11411_All, Unigene1168_All, Unigene1169_All, Unigene11724_All, Unigene11789_All, Unigene11814_All, Unigene11877_All, Unigene11920_All, Unigene12006_All, Unigene12017_All, Unigene12052_All, Unigene12161_All, Unigene12188_All, Unigene12259_All, Unigene12299_All, Unigene12319_All, Unigene12405_All, Unigene12414_All, Unigene12464_All, Unigene1247_All, Unigene12515_All, Unigene12516_All, Unigene12591_All, Unigene12682_All, Unigene12740_All, Unigene1278_All, Unigene12877_All, Unigene12891_All, Unigene12903_All, Unigene13134_All, Unigene13403_All, Unigene13421_All, Unigene13541_All, Unigene1396_All, Unigene14208_All, Unigene1479_All, Unigene1512_All, Unigene15827_All, Unigene16120_All, Unigene17046_All, Unigene1757_All, Unigene17689_All, Unigene17702_All, Unigene17856_All, Unigene17875_All, Unigene17994_All, Unigene18195_All, Unigene18306_All, Unigene184_All, Unigene1941_All, Unigene1971_All, Unigene20095_All, Unigene20260_All, Unigene20941_All, Unigene220_All, Unigene2212_All, Unigene2233_All, Unigene236_All, Unigene2513_All, Unigene3131_All, Unigene3751_All, Unigene3871_All, Unigene4022_All, Unigene4251_All, Unigene4581_All, Unigene480_All, Unigene4817_All, Unigene4935_All, Unigene4954_All, Unigene4970_All, Unigene4975_All, Unigene5198_All, Unigene54_All, Unigene5733_All, Unigene5781_All, Unigene5896_All, Unigene6046_All, Unigene6503_All, Unigene6655_All, Unigene6663_All, Unigene6969_All, Unigene7049_All, Unigene7234_All, Unigene7286_All, Unigene7671_All, Unigene7704_All, Unigene7759_All, Unigene8056_All, Unigene8077_All, Unigene8833_All, Unigene9006_All, Unigene9904_All

34	Hedgehog signaling pathway	CL1452.Contig1_All, CL1471.Contig1_All, CL1843.Contig3_All, CL2208.Contig1_All, CL2268.Contig1_All, CL2268.Contig2_All, CL2782.Contig1_All, CL2782.Contig2_All, CL2838.Contig1_All, CL2838.Contig2_All, CL2838.Contig4_All, CL3293.Contig1_All, CL3315.Contig3_All, CL3315.Contig4_All, CL3923.Contig1_All, CL3923.Contig3_All, CL396.Contig21_All, CL396.Contig22_All, CL4496.Contig3_All, CL4522.Contig2_All, CL5106.Contig1_All, CL63.Contig10_All, CL63.Contig9_All, CL925.Contig2_All, CL940.Contig3_All, CL940.Contig5_All, Unigene10083_All, Unigene10373_All, Unigene10761_All, Unigene11263_All, Unigene1188_All, Unigene11922_All, Unigene12415_All, Unigene12874_All, Unigene1680_All, Unigene18477_All, Unigene18765_All, Unigene236_All, Unigene247_All, Unigene2905_All, Unigene3054_All, Unigene3715_All, Unigene3779_All, Unigene3975_All, Unigene4112_All, Unigene4314_All, Unigene4731_All, Unigene4765_All, Unigene4893_All, Unigene7025_All, Unigene7026_All, Unigene7297_All, Unigene768_All, Unigene798_All, Unigene8014_All, Unigene8784_All, Unigene8861_All, Unigene9026_All, Unigene9111_All, Unigene9279_All, Unigene9733_All
35	Staphylococcus aureus infection	CL107.Contig3_All, CL1085.Contig3_All, CL1498.Contig1_All, CL2923.Contig2_All, CL3236.Contig1_All, CL326.Contig1_All, CL3489.Contig1_All, CL3848.Contig2_All, CL4535.Contig1_All, CL4597.Contig2_All, Unigene11197_All, Unigene11202_All, Unigene11922_All, Unigene17259_All, Unigene17404_All, Unigene18142_All, Unigene20583_All, Unigene2172_All, Unigene2771_All, Unigene3176_All, Unigene3337_All, Unigene649_All, Unigene7541_All, Unigene8217_All, Unigene8848_All, Unigene9855_All
36	Transcriptional misregulation in cancer	CL1019.Contig3_All, CL1019.Contig4_All, CL1091.Contig14_All, CL1104.Contig3_All, CL1104.Contig4_All, CL1104.Contig5_All, CL1104.Contig6_All, CL1104.Contig7_All, CL1104.Contig8_All, CL1104.Contig9_All, CL1109.Contig1_All, CL1109.Contig2_All, CL1109.Contig3_All, CL1109.Contig4_All, CL131.Contig1_All, CL131.Contig2_All, CL1387.Contig1_All, CL1387.Contig3_All, CL1433.Contig1_All, CL1433.Contig2_All, CL1444.Contig3_All, CL1637.Contig1_All, CL1648.Contig1_All, CL1985.Contig2_All, CL1985.Contig3_All, CL1985.Contig9_All, CL2052.Contig1_All, CL2052.Contig2_All, CL2066.Contig1_All, CL2156.Contig2_All, CL2353.Contig3_All, CL241.Contig1_All, CL241.Contig7_All, CL2658.Contig1_All, CL2658.Contig2_All, CL2684.Contig1_All, CL2684.Contig2_All, CL2733.Contig2_All, CL2733.Contig3_All, CL2864.Contig2_All, CL2901.Contig2_All, CL2901.Contig3_All, CL2901.Contig4_All, CL2901.Contig5_All, CL2901.Contig8_All, CL3043.Contig2_All, CL3069.Contig1_All, CL3137.Contig2_All, CL3567.Contig1_All, CL3567.Contig2_All, CL3567.Contig3_All, CL3798.Contig3_All, CL3815.Contig2_All, CL3920.Contig1_All, CL3920.Contig3_All, CL4158.Contig4_All, CL4158.Contig7_All, CL4381.Contig1_All, CL4477.Contig1_All, CL4477.Contig3_All, CL48.Contig1_All, CL4890.Contig1_All, CL5023.Contig1_All, CL504.Contig1_All, CL504.Contig2_All, CL5155.Contig1_All, CL5155.Contig3_All, CL5204.Contig1_All, CL5204.Contig2_All, CL5236.Contig4_All, CL5236.Contig5_All, CL5452.Contig2_All, CL5452.Contig5_All, CL5452.Contig6_All, CL653.Contig1_All, CL72.Contig1_All, CL72.Contig2_All, CL748.Contig1_All, CL748.Contig3_All, CL83.Contig2_All, CL869.Contig1_All, CL869.Contig2_All, CL880.Contig3_All, CL95.Contig1_All, Unigene10001_All, Unigene10098_All, Unigene10344_All, Unigene1045_All, Unigene1046_All, Unigene10687_All, Unigene10762_All, Unigene10833_All, Unigene11145_All, Unigene11206_All, Unigene11384_All, Unigene1150_All, Unigene11579_All, Unigene1163_All, Unigene11652_All, Unigene12016_All, Unigene12044_All, Unigene12228_All, Unigene12322_All, Unigene1238_All, Unigene12810_All, Unigene1301_All, Unigene13060_All, Unigene13062_All, Unigene145_All, Unigene1663_All, Unigene1679_All, Unigene1757_All, Unigene1797_All, Unigene18278_All, Unigene18388_All, Unigene18390_All, Unigene1857_All, Unigene19340_All, Unigene19758_All, Unigene20090_All, Unigene200_All, Unigene2089_All, Unigene2160_All, Unigene2525_All, Unigene2654_All, Unigene3121_All, Unigene3982_All, Unigene4082_All, Unigene4101_All, Unigene4144_All, Unigene4157_All, Unigene4676_All, Unigene4677_All, Unigene4696_All, Unigene4807_All, Unigene5153_All, Unigene5179_All, Unigene5468_All, Unigene5623_All, Unigene5643_All, Unigene5770_All, Unigene5877_All, Unigene6009_All, Unigene6058_All, Unigene6069_All, Unigene6096_All, Unigene6222_All, Unigene6223_All, Unigene6236_All, Unigene6265_All, Unigene6916_All, Unigene6961_All, Unigene7190_All, Unigene7220_All, Unigene7287_All, Unigene8102_All, Unigene8232_All, Unigene8848_All, Unigene9006_All, Unigene9065_All, Unigene9067_All, Unigene9140_All, Unigene9552_All, Unigene9745_All, Unigene9867_All
37	Phototransduction	CL1048.Contig2_All, CL1286.Contig2_All, CL1805.Contig2_All, CL1805.Contig3_All, CL1823.Contig8_All, CL2014.Contig4_All, CL2014.Contig5_All, CL2014.Contig6_All, CL2131.Contig4_All, CL2131.Contig5_All, CL2131.Contig6_All, CL2131.Contig8_All, CL2402.Contig1_All, CL2466.Contig1_All, CL2466.Contig2_All, CL2468.Contig1_All, CL2468.Contig2_All, CL2642.Contig1_All, CL2642.Contig2_All, CL2801.Contig1_All, CL2801.Contig2_All, CL3023.Contig1_All, CL3023.Contig2_All, CL3023.Contig3_All, CL3023.Contig4_All, CL3023.Contig5_All, CL3667.Contig1_All, CL3667.Contig2_All, CL3667.Contig3_All, CL3803.Contig1_All, CL3803.Contig2_All, CL4395.Contig1_All, CL4509.Contig1_All, CL4509.Contig2_All, CL4633.Contig1_All, CL4633.Contig2_All, CL484.Contig1_All, CL497.Contig2_All, CL497.Contig3_All, CL772.Contig1_All, CL772.Contig2_All, Unigene10006_All, Unigene10574_All, Unigene11054_All, Unigene12315_All, Unigene12719_All, Unigene16098_All, Unigene16498_All, Unigene16769_All, Unigene17959_All, Unigene18121_All, Unigene2209_All, Unigene3327_All, Unigene3996_All, Unigene4009_All, Unigene4013_All, Unigene4221_All, Unigene4269_All, Unigene4541_All, Unigene4780_All, Unigene5051_All, Unigene5675_All, Unigene5803_All, Unigene6266_All, Unigene6525_All, Unigene7104_All, Unigene7869_All, Unigene8026_All, Unigene8786_All, Unigene9137_All, Unigene9851_All

38	Prion diseases	CL1012.Contig4_All, CL1012.Contig5_All, CL1012.Contig6_All, CL1012.Contig7_All, CL1012.Contig9_All, CL1054.Contig1_All, CL1054.Contig2_All, CL1193.Contig1_All, CL1193.Contig2_All, CL1193.Contig3_All, CL1452.Contig1_All, CL1925.Contig1_All, CL2165.Contig2_All, CL2563.Contig2_All, CL257.Contig1_All, CL257.Contig2_All, CL2686.Contig1_All, CL2686.Contig2_All, CL2824.Contig3_All, CL320.Contig1_All, CL320.Contig2_All, CL3742.Contig1_All, CL400.Contig1_All, CL400.Contig2_All, CL4787.Contig1_All, CL5048.Contig1_All, CL5256.Contig1_All, CL5256.Contig2_All, CL5256.Contig4_All, CL5432.Contig1_All, CL558.Contig1_All, CL558.Contig2_All, CL558.Contig4_All, CL940.Contig3_All, CL940.Contig5_All, Unigene10374_All, Unigene1040_All, Unigene10837_All, Unigene10948_All, Unigene12109_All, Unigene12175_All, Unigene12186_All, Unigene12810_All, Unigene12871_All, Unigene12890_All, Unigene13167_All, Unigene13331_All, Unigene15587_All, Unigene15863_All, Unigene16093_All, Unigene16965_All, Unigene16966_All, Unigene17728_All, Unigene18054_All, Unigene18488_All, Unigene18535_All, Unigene19237_All, Unigene19880_All, Unigene230_All, Unigene3080_All, Unigene32_All, Unigene3314_All, Unigene3779_All, Unigene4112_All, Unigene5064_All, Unigene6101_All, Unigene7093_All, Unigene8986_All, Unigene9065_All, Unigene9097_All, Unigene9733_All, Unigene9971_All
39	Pancreatic secretion	CL1087.Contig2_All, CL1087.Contig3_All, CL1535.Contig3_All, CL1880.Contig1_All, CL1880.Contig2_All, CL1880.Contig3_All, CL1987.Contig1_All, CL199.Contig1_All, CL2137.Contig1_All, CL2137.Contig2_All, CL2182.Contig1_All, CL2182.Contig3_All, CL2182.Contig4_All, CL2213.Contig1_All, CL2213.Contig2_All, CL2213.Contig4_All, CL2432.Contig2_All, CL2474.Contig2_All, CL2854.Contig1_All, CL2930.Contig5_All, CL349.Contig12_All, CL3545.Contig2_All, CL3776.Contig2_All, CL39.Contig1_All, CL39.Contig2_All, CL39.Contig3_All, CL39.Contig4_All, CL4005.Contig2_All, CL4005.Contig3_All, CL4084.Contig1_All, CL4084.Contig2_All, CL41.Contig3_All, CL41.Contig4_All, CL41.Contig8_All, CL4262.Contig2_All, CL440.Contig3_All, CL440.Contig5_All, CL440.Contig6_All, CL5310.Contig2_All, CL5391.Contig1_All, CL5391.Contig2_All, CL5399.Contig1_All, CL5399.Contig2_All, CL5428.Contig1_All, CL5428.Contig2_All, CL573.Contig6_All, CL600.Contig1_All, CL600.Contig2_All, CL600.Contig3_All, CL600.Contig4_All, CL600.Contig6_All, CL662.Contig1_All, CL691.Contig1_All, CL860.Contig10_All, CL860.Contig8_All, CL860.Contig9_All, CL878.Contig1_All, CL878.Contig2_All, CL878.Contig3_All, CL878.Contig4_All, CL878.Contig5_All, CL940.Contig3_All, CL940.Contig5_All, CL974.Contig2_All, Unigene10040_All, Unigene10058_All, Unigene10131_All, Unigene10471_All, Unigene10594_All, Unigene11086_All, Unigene11307_All, Unigene11480_All, Unigene11482_All, Unigene11483_All, Unigene11660_All, Unigene12454_All, Unigene125_All, Unigene14005_All, Unigene15194_All, Unigene15195_All, Unigene17380_All, Unigene18002_All, Unigene18652_All, Unigene18672_All, Unigene18898_All, Unigene18993_All, Unigene2002_All, Unigene2003_All, Unigene2029_All, Unigene2170_All, Unigene2172_All, Unigene2209_All, Unigene2250_All, Unigene2763_All, Unigene3010_All, Unigene3029_All, Unigene3033_All, Unigene3034_All, Unigene3036_All, Unigene3037_All, Unigene3042_All, Unigene4053_All, Unigene4070_All, Unigene4211_All, Unigene4310_All, Unigene4323_All, Unigene4532_All, Unigene5724_All, Unigene5808_All, Unigene5877_All, Unigene590_All, Unigene6215_All, Unigene6639_All, Unigene7061_All, Unigene7206_All, Unigene7284_All, Unigene7658_All, Unigene7957_All, Unigene8039_All, Unigene8109_All, Unigene8208_All, Unigene8422_All, Unigene8464_All, Unigene8700_All, Unigene8770_All, Unigene8848_All, Unigene9050_All, Unigene9163_All, Unigene93_All, Unigene9437_All, Unigene9668_All, Unigene9710_All, Unigene9877_All
40	Morphine addiction	CL1184.Contig1_All, CL1184.Contig2_All, CL1184.Contig3_All, CL1184.Contig4_All, CL1184.Contig5_All, CL1452.Contig1_All, CL1546.Contig1_All, CL1546.Contig3_All, CL1546.Contig4_All, CL1546.Contig5_All, CL1546.Contig6_All, CL1546.Contig7_All, CL1546.Contig8_All, CL1675.Contig2_All, CL2161.Contig1_All, CL2161.Contig2_All, CL2182.Contig1_All, CL2182.Contig3_All, CL2182.Contig4_All, CL225.Contig1_All, CL225.Contig3_All, CL2301.Contig2_All, CL2301.Contig4_All, CL260.Contig15_All, CL2642.Contig1_All, CL2642.Contig2_All, CL2703.Contig2_All, CL2735.Contig1_All, CL2770.Contig1_All, CL2770.Contig2_All, CL3166.Contig1_All, CL3507.Contig1_All, CL3597.Contig1_All, CL3597.Contig2_All, CL3676.Contig1_All, CL3836.Contig1_All, CL3836.Contig2_All, CL39.Contig1_All, CL39.Contig2_All, CL39.Contig3_All, CL39.Contig4_All, CL4005.Contig2_All, CL4005.Contig3_All, CL4262.Contig2_All, CL440.Contig3_All, CL440.Contig5_All, CL440.Contig6_All, CL4923.Contig2_All, CL5105.Contig1_All, CL5105.Contig2_All, CL5203.Contig1_All, CL5203.Contig2_All, CL5391.Contig1_All, CL5391.Contig2_All, CL677.Contig2_All, CL679.Contig1_All, CL679.Contig2_All, CL847.Contig3_All, CL894.Contig1_All, CL940.Contig3_All, CL940.Contig5_All, CL973.Contig1_All, Unigene10058_All, Unigene1044_All, Unigene10931_All, Unigene11615_All, Unigene12110_All, Unigene12433_All, Unigene12535_All, Unigene13612_All, Unigene16890_All, Unigene1743_All, Unigene1766_All, Unigene176_All, Unigene17896_All, Unigene18898_All, Unigene1925_All, Unigene2029_All, Unigene2032_All, Unigene2196_All, Unigene3779_All, Unigene3967_All, Unigene4112_All, Unigene4126_All, Unigene4532_All, Unigene4780_All, Unigene5063_All, Unigene5521_All, Unigene555_All, Unigene6217_All, Unigene68_All, Unigene69_All, Unigene8071_All, Unigene8170_All, Unigene8993_All, Unigene9167_All, Unigene93_All, Unigene9733_All
41	Sulfur relay system	CL15.Contig1_All, CL15.Contig2_All, CL15.Contig3_All, CL1843.Contig1_All, CL2479.Contig1_All, CL2479.Contig2_All, CL396.Contig12_All, CL396.Contig13_All, CL396.Contig14_All, CL396.Contig15_All, CL396.Contig16_All, CL396.Contig17_All, CL396.Contig18_All, CL396.Contig19_All, CL396.Contig1_All, CL396.Contig2_All, CL396.Contig32_All, CL396.Contig3_All, CL396.Contig4_All, CL396.Contig5_All, CL396.Contig6_All, CL396.Contig7_All, CL396.Contig8_All, CL396.Contig9_All, CL4152.Contig2_All, CL4242.Contig1_All, CL491.Contig2_All, CL491.Contig6_All, CL491.Contig8_All, Unigene13496_All, Unigene3252_All, Unigene9326_All

42	Olfactory transduction	CL1048.Contig2_All, CL1286.Contig2_All, CL1452.Contig1_All, CL1800.Contig2_All, CL1800.Contig3_All, CL1823.Contig8_All, CL2014.Contig4_All, CL2014.Contig5_All, CL2014.Contig6_All, CL2131.Contig4_All, CL2131.Contig5_All, CL2131.Contig6_All, CL2131.Contig8_All, CL2146.Contig4_All, CL2466.Contig1_All, CL2466.Contig2_All, CL2466.Contig3_All, CL2468.Contig1_All, CL2468.Contig2_All, CL260.Contig15_All, CL2642.Contig1_All, CL2642.Contig2_All, CL2762.Contig4_All, CL3311.Contig1_All, CL3311.Contig2_All, CL3667.Contig1_All, CL3667.Contig2_All, CL3667.Contig3_All, CL3679.Contig2_All, CL3803.Contig1_All, CL3803.Contig2_All, CL4181.Contig1_All, CL4181.Contig4_All, CL4395.Contig1_All, CL44.Contig10_All, CL44.Contig12_All, CL44.Contig13_All, CL44.Contig16_All, CL44.Contig17_All, CL44.Contig18_All, CL44.Contig19_All, CL44.Contig1_All, CL44.Contig22_All, CL44.Contig23_All, CL44.Contig2_All, CL44.Contig3_All, CL44.Contig4_All, CL44.Contig5_All, CL44.Contig6_All, CL44.Contig8_All, CL44.Contig9_All, CL4509.Contig1_All, CL4509.Contig2_All, CL4633.Contig1_All, CL4633.Contig2_All, CL4684.Contig1_All, CL484.Contig1_All, CL497.Contig2_All, CL497.Contig3_All, CL5004.Contig1_All, CL772.Contig1_All, CL772.Contig2_All, CL940.Contig3_All, CL940.Contig5_All, Unigene10006_All, Unigene10131_All, Unigene10574_All, Unigene11054_All, Unigene11730_All, Unigene12315_All, Unigene12719_All, Unigene16098_All, Unigene16498_All, Unigene16769_All, Unigene17001_All, Unigene17469_All, Unigene17959_All, Unigene265_All, Unigene2795_All, Unigene3327_All, Unigene3779_All, Unigene3861_All, Unigene3996_All, Unigene4009_All, Unigene4013_All, Unigene4112_All, Unigene4221_All, Unigene4269_All, Unigene4541_All, Unigene4643_All, Unigene4783_All, Unigene4817_All, Unigene5051_All, Unigene555_All, Unigene5675_All, Unigene5803_All, Unigene6266_All, Unigene6525_All, Unigene7039_All, Unigene7104_All, Unigene7658_All, Unigene7869_All, Unigene8026_All, Unigene8039_All, Unigene8210_All, Unigene9117_All, Unigene9137_All, Unigene9733_All, Unigene9851_All
43	Bacterial invasion of epithelial cells	CL1066.Contig1_All, CL1066.Contig2_All, CL1066.Contig3_All, CL1355.Contig1_All, CL1535.Contig3_All, CL156.Contig1_All, CL156.Contig3_All, CL156.Contig4_All, CL1723.Contig1_All, CL1723.Contig2_All, CL1723.Contig7_All, CL1795.Contig5_All, CL1803.Contig1_All, CL1874.Contig2_All, CL1953.Contig1_All, CL2019.Contig1_All, CL2019.Contig2_All, CL2034.Contig1_All, CL2034.Contig2_All, CL2066.Contig1_All, CL2085.Contig1_All, CL2091.Contig5_All, CL2091.Contig6_All, CL2375.Contig2_All, CL2658.Contig1_All, CL2658.Contig2_All, CL271.Contig2_All, CL2975.Contig2_All, CL3027.Contig3_All, CL3114.Contig2_All, CL3116.Contig2_All, CL3315.Contig3_All, CL3315.Contig4_All, CL3436.Contig1_All, CL3436.Contig4_All, CL3436.Contig9_All, CL3510.Contig3_All, CL367.Contig1_All, CL367.Contig2_All, CL367.Contig3_All, CL3888.Contig2_All, CL399.Contig10_All, CL399.Contig1_All, CL399.Contig2_All, CL399.Contig3_All, CL399.Contig4_All, CL399.Contig5_All, CL399.Contig6_All, CL399.Contig7_All, CL399.Contig8_All, CL399.Contig9_All, CL402.Contig1_All, CL4088.Contig2_All, CL4130.Contig1_All, CL4203.Contig3_All, CL4381.Contig1_All, CL4479.Contig2_All, CL4479.Contig3_All, CL4479.Contig5_All, CL4541.Contig1_All, CL4541.Contig2_All, CL4559.Contig2_All, CL4559.Contig3_All, CL495.Contig11_All, CL495.Contig1_All, CL495.Contig3_All, CL495.Contig4_All, CL495.Contig5_All, CL495.Contig6_All, CL5212.Contig1_All, CL5212.Contig2_All, CL5361.Contig2_All, CL5361.Contig4_All, CL5468.Contig3_All, CL695.Contig16_All, CL748.Contig1_All, CL748.Contig3_All, Unigene10097_All, Unigene10448_All, Unigene10998_All, Unigene11082_All, Unigene11307_All, Unigene11459_All, Unigene11521_All, Unigene11597_All, Unigene11598_All, Unigene11599_All, Unigene12201_All, Unigene12603_All, Unigene12684_All, Unigene13036_All, Unigene13092_All, Unigene13225_All, Unigene13978_All, Unigene1447_All, Unigene1679_All, Unigene16840_All, Unigene16963_All, Unigene1855_All, Unigene18598_All, Unigene19601_All, Unigene2741_All, Unigene278_All, Unigene3088_All, Unigene3482_All, Unigene3945_All, Unigene4207_All, Unigene5303_All, Unigene5727_All, Unigene5808_All, Unigene5813_All, Unigene5873_All, Unigene6014_All, Unigene6029_All, Unigene7016_All, Unigene7058_All, Unigene7547_All, Unigene7702_All, Unigene7751_All, Unigene783_All, Unigene8184_All, Unigene8357_All, Unigene8933_All, Unigene9050_All, Unigene9379_All, Unigene9740_All, Unigene9785_All, Unigene9787_All, Unigene9877_All, Unigene9902_All, Unigene9947_All

44	Spliceosome	CL1014.Contig1_All, CL1014.Contig2_All, CL104.Contig3_All, CL104.Contig4_All, CL104.Contig5_All, CL1045.Contig2_All, CL1054.Contig1_All, CL1054.Contig2_All, CL1279.Contig1_All, CL1279.Contig2_All, CL1291.Contig1_All, CL1291.Contig2_All, CL1291.Contig3_All, CL1291.Contig4_All, CL1339.Contig2_All, CL1406.Contig1_All, CL1406.Contig2_All, CL1457.Contig1_All, CL1457.Contig2_All, CL1457.Contig4_All, CL1485.Contig1_All, CL1485.Contig2_All, CL1485.Contig3_All, CL1485.Contig4_All, CL1485.Contig5_All, CL1485.Contig6_All, CL1485.Contig7_All, CL1485.Contig8_All, CL1487.Contig8_All, CL1498.Contig1_All, CL1507.Contig1_All, CL1507.Contig2_All, CL160.Contig1_All, CL1603.Contig2_All, CL1884.Contig1_All, CL1925.Contig1_All, CL196.Contig2_All, CL196.Contig4_All, CL196.Contig5_All, CL196.Contig7_All, CL196.Contig8_All, CL2044.Contig1_All, CL2044.Contig5_All, CL2165.Contig2_All, CL2202.Contig1_All, CL2202.Contig2_All, CL2251.Contig1_All, CL2251.Contig2_All, CL2480.Contig2_All, CL2480.Contig3_All, CL264.Contig4_All, CL264.Contig9_All, CL2664.Contig1_All, CL2664.Contig2_All, CL2664.Contig3_All, CL2686.Contig1_All, CL2686.Contig2_All, CL2700.Contig1_All, CL2700.Contig2_All, CL2718.Contig1_All, CL2830.Contig6_All, CL2864.Contig2_All, CL2904.Contig1_All, CL2904.Contig2_All, CL2909.Contig1_All, CL2909.Contig3_All, CL2909.Contig4_All, CL2945.Contig1_All, CL3024.Contig1_All, CL3057.Contig1_All, CL3057.Contig2_All, CL3069.Contig1_All, CL319.Contig10_All, CL319.Contig11_All, CL319.Contig1_All, CL319.Contig9_All, CL3224.Contig1_All, CL3488.Contig1_All, CL3488.Contig2_All, CL3488.Contig3_All, CL3547.Contig1_All, CL3547.Contig2_All, CL3547.Contig4_All, CL3570.Contig1_All, CL3579.Contig1_All, CL36.Contig3_All, CL3616.Contig1_All, CL3653.Contig1_All, CL3955.Contig1_All, CL4098.Contig3_All, CL4150.Contig4_All, CL4155.Contig1_All, CL4375.Contig2_All, CL4394.Contig2_All, CL449.Contig1_All, CL4679.Contig1_All, CL4679.Contig2_All, CL48.Contig1_All, CL4803.Contig1_All, CL4808.Contig1_All, CL4955.Contig1_All, CL4955.Contig2_All, CL5046.Contig1_All, CL5046.Contig2_All, CL5077.Contig1_All, CL5077.Contig2_All, CL5107.Contig1_All, CL5107.Contig2_All, CL5214.Contig1_All, CL5214.Contig2_All, CL5229.Contig1_All, CL5229.Contig2_All, CL5256.Contig1_All, CL5256.Contig2_All, CL5256.Contig4_All, CL5262.Contig1_All, CL5262.Contig2_All, CL5384.Contig1_All, CL5384.Contig2_All, CL5467.Contig2_All, CL558.Contig1_All, CL558.Contig2_All, CL558.Contig4_All, CL56.Contig1_All, CL56.Contig4_All, CL603.Contig1_All, CL603.Contig2_All, CL603.Contig3_All, CL746.Contig1_All, CL79.Contig2_All, CL83.Contig2_All, Unigene10053_All, Unigene10210_All, Unigene1021_All, Unigene10220_All, Unigene10221_All, Unigene10312_All, Unigene10344_All, Unigene1048_All, Unigene10744_All, Unigene10837_All, Unigene10905_All, Unigene11044_All, Unigene11067_All, Unigene11068_All, Unigene11098_All, Unigene11523_All, Unigene11579_All, Unigene11652_All, Unigene11789_All, Unigene11807_All, Unigene11885_All, Unigene11905_All, Unigene11920_All, Unigene11937_All, Unigene11946_All, Unigene11950_All, Unigene11961_All, Unigene12005_All, Unigene12044_All, Unigene12052_All, Unigene12078_All, Unigene12122_All, Unigene12124_All, Unigene12129_All, Unigene12192_All, Unigene12289_All, Unigene12299_All, Unigene12309_All, Unigene12319_All, Unigene12335_All, Unigene12383_All, Unigene12405_All, Unigene12414_All, Unigene12464_All, Unigene12474_All, Unigene12495_All, Unigene12515_All, Unigene12618_All, Unigene12661_All, Unigene12663_All, Unigene12669_All, Unigene12682_All, Unigene12696_All, Unigene12764_All, Unigene12949_All, Unigene12991_All, Unigene13010_All, Unigene13147_All, Unigene13167_All, Unigene13175_All, Unigene1331_All, Unigene13403_All, Unigene13731_All, Unigene13938_All, Unigene13_All, Unigene1400_All, Unigene15587_All, Unigene15863_All, Unigene16093_All, Unigene1697_All, Unigene16985_All, Unigene1737_All, Unigene17689_All, Unigene17964_All, Unigene18306_All, Unigene18381_All, Unigene18388_All, Unigene18390_All, Unigene184_All, Unigene18535_All, Unigene1971_All, Unigene2063_All, Unigene20941_All, Unigene2212_All, Unigene2812_All, Unigene2872_All, Unigene3554_All, Unigene367_All, Unigene3724_All, Unigene3751_All, Unigene3835_All, Unigene3920_All, Unigene3950_All, Unigene4226_All, Unigene453_All, Unigene4859_All, Unigene4954_All, Unigene5079_All, Unigene5092_All, Unigene5125_All, Unigene5215_All, Unigene5348_All, Unigene5498_All, Unigene5682_All, Unigene5717_All, Unigene5789_All, Unigene5945_All, Unigene5956_All, Unigene5993_All, Unigene6014_All, Unigene6043_All, Unigene6208_All, Unigene6225_All, Unigene6503_All, Unigene67_All, Unigene6969_All, Unigene6971_All, Unigene7283_All, Unigene7646_All, Unigene7744_All, Unigene780_All, Unigene7929_All, Unigene7988_All, Unigene8056_All, Unigene8077_All, Unigene8078_All, Unigene8247_All, Unigene8301_All, Unigene832_All, Unigene8580_All, Unigene8768_All, Unigene9085_All, Unigene9091_All, Unigene9153_All, Unigene921_All, Unigene9448_All, Unigene9611_All, Unigene9820_All, Unigene9839_All, Unigene9855_All, Unigene9866_All, Unigene9896_All, Unigene9971_All
45	Long-term depression	CL126.Contig2_All, CL1559.Contig1_All, CL199.Contig1_All, CL2182.Contig1_All, CL2182.Contig3_All, CL2182.Contig4_All, CL241.Contig1_All, CL241.Contig7_All, CL2432.Contig2_All, CL2930.Contig5_All, CL3294.Contig1_All, CL3311.Contig1_All, CL3311.Contig2_All, CL39.Contig1_All, CL39.Contig2_All, CL39.Contig3_All, CL39.Contig4_All, CL4181.Contig1_All, CL4181.Contig4_All, CL4439.Contig2_All, CL4727.Contig2_All, CL4787.Contig1_All, CL4923.Contig2_All, CL5098.Contig2_All, CL5310.Contig2_All, CL5391.Contig1_All, CL5391.Contig2_All, CL5399.Contig1_All, CL5399.Contig2_All, CL648.Contig2_All, CL691.Contig1_All, CL860.Contig10_All, CL860.Contig8_All, CL860.Contig9_All, CL940.Contig3_All, CL940.Contig5_All, CL95.Contig1_All, Unigene10058_All, Unigene10242_All, Unigene11730_All, Unigene12671_All, Unigene13432_All, Unigene15176_All, Unigene1743_All, Unigene176_All, Unigene18898_All, Unigene18993_All, Unigene19288_All, Unigene2196_All, Unigene2209_All, Unigene2795_All, Unigene3029_All, Unigene3033_All, Unigene3034_All, Unigene3036_All, Unigene3037_All, Unigene3042_All, Unigene3861_All, Unigene4053_All, Unigene4070_All, Unigene4126_All, Unigene4532_All, Unigene4783_All, Unigene5521_All, Unigene6217_All, Unigene6242_All, Unigene69_All, Unigene807_All, Unigene8422_All, Unigene8747_All, Unigene9437_All

46	GABAergic synapse	CL1273.Contig1_All, CL1273.Contig2_All, CL1452.Contig1_All, CL1586.Contig4_All, CL1599.Contig1_All, CL1599.Contig2_All, CL1675.Contig2_All, CL1802.Contig1_All, CL1802.Contig2_All, CL2161.Contig1_All, CL2161.Contig2_All, CL2182.Contig1_All, CL2182.Contig3_All, CL2182.Contig4_All, CL225.Contig1_All, CL225.Contig3_All, CL2382.Contig4_All, CL2550.Contig3_All, CL2703.Contig2_All, CL2735.Contig1_All, CL2862.Contig1_All, CL2862.Contig2_All, CL2862.Contig3_All, CL2862.Contig4_All, CL3118.Contig1_All, CL3118.Contig2_All, CL3166.Contig1_All, CL3283.Contig1_All, CL3507.Contig1_All, CL3546.Contig1_All, CL3546.Contig2_All, CL3597.Contig1_All, CL3597.Contig2_All, CL3676.Contig1_All, CL39.Contig1_All, CL39.Contig2_All, CL39.Contig3_All, CL39.Contig4_All, CL3990.Contig6_All, CL4005.Contig2_All, CL4005.Contig3_All, CL4262.Contig2_All, CL440.Contig3_All, CL440.Contig5_All, CL440.Contig6_All, CL4490.Contig1_All, CL4490.Contig2_All, CL4923.Contig2_All, CL5105.Contig1_All, CL5105.Contig2_All, CL5325.Contig2_All, CL5391.Contig1_All, CL5391.Contig2_All, CL567.Contig4_All, CL677.Contig2_All, CL679.Contig1_All, CL679.Contig2_All, CL847.Contig3_All, CL894.Contig1_All, CL940.Contig3_All, CL940.Contig5_All, CL973.Contig1_All, Unigene10412_All, Unigene1044_All, Unigene10909_All, Unigene10910_All, Unigene10931_All, Unigene11615_All, Unigene11856_All, Unigene11887_All, Unigene12110_All, Unigene12433_All, Unigene12470_All, Unigene12535_All, Unigene131_All, Unigene13612_All, Unigene16890_All, Unigene1743_All, Unigene1766_All, Unigene176_All, Unigene17763_All, Unigene17896_All, Unigene18234_All, Unigene18898_All, Unigene1925_All, Unigene19942_All, Unigene2029_All, Unigene2032_All, Unigene2196_All, Unigene2807_All, Unigene3017_All, Unigene3655_All, Unigene3779_All, Unigene4112_All, Unigene4126_All, Unigene4780_All, Unigene5521_All, Unigene6217_All, Unigene6315_All, Unigene68_All, Unigene69_All, Unigene7234_All, Unigene8071_All, Unigene8170_All, Unigene8509_All, Unigene8621_All, Unigene8993_All, Unigene9167_All, Unigene93_All, Unigene9733_All
47	Dopaminergic synapse	CL1066.Contig1_All, CL1066.Contig2_All, CL1286.Contig2_All, CL1452.Contig1_All, CL1563.Contig2_All, CL1675.Contig2_All, CL1823.Contig8_All, CL1843.Contig3_All, CL2131.Contig4_All, CL2131.Contig5_All, CL2131.Contig6_All, CL2131.Contig8_All, CL2182.Contig1_All, CL2182.Contig3_All, CL2182.Contig4_All, CL2400.Contig5_All, CL244.Contig2_All, CL2468.Contig1_All, CL2468.Contig2_All, CL2475.Contig1_All, CL2475.Contig2_All, CL2642.Contig1_All, CL2642.Contig2_All, CL2873.Contig1_All, CL2873.Contig2_All, CL2930.Contig5_All, CL3286.Contig1_All, CL3667.Contig1_All, CL3667.Contig2_All, CL3667.Contig3_All, CL3689.Contig2_All, CL3787.Contig1_All, CL3787.Contig2_All, CL39.Contig1_All, CL39.Contig2_All, CL39.Contig3_All, CL39.Contig4_All, CL3920.Contig2_All, CL3948.Contig3_All, CL4002.Contig1_All, CL4302.Contig1_All, CL4302.Contig2_All, CL4302.Contig3_All, CL4302.Contig4_All, CL4395.Contig1_All, CL44.Contig10_All, CL44.Contig12_All, CL44.Contig13_All, CL44.Contig16_All, CL44.Contig17_All, CL44.Contig18_All, CL44.Contig19_All, CL44.Contig1_All, CL44.Contig22_All, CL44.Contig23_All, CL44.Contig2_All, CL44.Contig3_All, CL44.Contig4_All, CL44.Contig5_All, CL44.Contig6_All, CL44.Contig8_All, CL44.Contig9_All, CL4509.Contig1_All, CL4509.Contig2_All, CL4521.Contig1_All, CL4521.Contig2_All, CL4521.Contig3_All, CL4521.Contig5_All, CL4594.Contig1_All, CL4923.Contig2_All, CL4966.Contig1_All, CL5115.Contig1_All, CL5391.Contig1_All, CL5391.Contig2_All, CL5399.Contig1_All, CL5399.Contig2_All, CL691.Contig1_All, CL741.Contig2_All, CL761.Contig1_All, CL761.Contig2_All, CL772.Contig1_All, CL772.Contig2_All, CL870.Contig4_All, CL870.Contig8_All, CL870.Contig9_All, CL940.Contig3_All, CL940.Contig5_All, Unigene10006_All, Unigene10058_All, Unigene10242_All, Unigene10574_All, Unigene11054_All, Unigene1168_All, Unigene11922_All, Unigene12110_All, Unigene12315_All, Unigene12719_All, Unigene13432_All, Unigene1512_All, Unigene16769_All, Unigene1743_All, Unigene176_All, Unigene18898_All, Unigene18993_All, Unigene19148_All, Unigene1925_All, Unigene1941_All, Unigene2029_All, Unigene2032_All, Unigene2193_All, Unigene2196_All, Unigene2209_All, Unigene220_All, Unigene2233_All, Unigene236_All, Unigene247_All, Unigene2749_All, Unigene2760_All, Unigene3779_All, Unigene3996_All, Unigene4013_All, Unigene4022_All, Unigene4053_All, Unigene4070_All, Unigene4112_All, Unigene4116_All, Unigene4126_All, Unigene4532_All, Unigene4780_All, Unigene4935_All, Unigene4970_All, Unigene5521_All, Unigene5675_All, Unigene6217_All, Unigene6655_All, Unigene6746_All, Unigene69_All, Unigene7297_All, Unigene7298_All, Unigene7702_All, Unigene7704_All, Unigene7759_All, Unigene8422_All, Unigene8621_All, Unigene8784_All, Unigene9437_All, Unigene9733_All, Unigene9904_All
48	Legionellosis	CL1054.Contig1_All, CL1054.Contig2_All, CL1085.Contig3_All, CL1401.Contig1_All, CL1534.Contig1_All, CL1534.Contig2_All, CL1818.Contig1_All, CL1818.Contig2_All, CL1818.Contig4_All, CL1818.Contig5_All, CL1818.Contig6_All, CL1925.Contig1_All, CL1927.Contig2_All, CL2049.Contig3_All, CL2165.Contig2_All, CL2279.Contig2_All, CL2686.Contig1_All, CL2686.Contig2_All, CL2872.Contig1_All, CL2872.Contig3_All, CL2872.Contig5_All, CL2882.Contig1_All, CL2882.Contig2_All, CL3332.Contig2_All, CL3620.Contig2_All, CL3725.Contig2_All, CL4184.Contig1_All, CL4184.Contig2_All, CL4320.Contig3_All, CL5256.Contig1_All, CL5256.Contig2_All, CL5256.Contig4_All, CL5299.Contig1_All, CL5311.Contig1_All, CL5428.Contig1_All, CL5428.Contig2_All, CL558.Contig1_All, CL558.Contig2_All, CL558.Contig4_All, CL851.Contig3_All, Unigene10179_All, Unigene10711_All, Unigene10714_All, Unigene10837_All, Unigene11539_All, Unigene11724_All, Unigene11911_All, Unigene11919_All, Unigene12188_All, Unigene12284_All, Unigene12677_All, Unigene15302_All, Unigene15303_All, Unigene15587_All, Unigene15863_All, Unigene16093_All, Unigene16820_All, Unigene1757_All, Unigene17702_All, Unigene18149_All, Unigene18214_All, Unigene18535_All, Unigene20095_All, Unigene3976_All, Unigene4581_All, Unigene6058_All, Unigene7049_All, Unigene7205_All, Unigene7245_All, Unigene7776_All, Unigene9012_All, Unigene9664_All, Unigene9971_All

49	Protein digestion and absorption	CL1011.Contig1_All, CL1011.Contig4_All, CL1055.Contig1_All, CL1055.Contig3_All, CL1055.Contig4_All, CL1055.Contig5_All, CL1055.Contig6_All, CL1055.Contig7_All, CL1092.Contig1_All, CL1147.Contig1_All, CL1147.Contig2_All, CL1183.Contig1_All, CL1183.Contig2_All, CL1202.Contig1_All, CL1202.Contig2_All, CL1436.Contig2_All, CL1437.Contig1_All, CL150.Contig2_All, CL1566.Contig1_All, CL1566.Contig2_All, CL1668.Contig1_All, CL1718.Contig1_All, CL1718.Contig2_All, CL1890.Contig4_All, CL190.Contig1_All, CL1989.Contig1_All, CL1989.Contig2_All, CL1989.Contig3_All, CL1989.Contig4_All, CL1989.Contig5_All, CL2009.Contig2_All, CL209.Contig1_All, CL209.Contig2_All, CL209.Contig3_All, CL209.Contig4_All, CL2099.Contig1_All, CL2137.Contig1_All, CL2137.Contig2_All, CL2213.Contig1_All, CL2213.Contig2_All, CL2213.Contig4_All, CL2252.Contig1_All, CL2252.Contig2_All, CL2252.Contig3_All, CL2297.Contig2_All, CL2474.Contig2_All, CL259.Contig2_All, CL259.Contig3_All, CL2611.Contig1_All, CL2611.Contig2_All, CL2927.Contig1_All, CL2927.Contig3_All, CL2952.Contig1_All, CL298.Contig1_All, CL298.Contig2_All, CL3106.Contig1_All, CL3160.Contig2_All, CL3327.Contig1_All, CL3327.Contig2_All, CL3371.Contig1_All, CL3387.Contig1_All, CL3387.Contig2_All, CL3622.Contig1_All, CL3622.Contig2_All, CL3776.Contig2_All, CL3797.Contig1_All, CL3797.Contig2_All, CL3848.Contig1_All, CL3880.Contig1_All, CL3880.Contig2_All, CL4026.Contig1_All, CL4042.Contig1_All, CL4042.Contig2_All, CL4042.Contig3_All, CL41.Contig3_All, CL41.Contig4_All, CL41.Contig8_All, CL4186.Contig2_All, CL4268.Contig1_All, CL4401.Contig2_All, CL4401.Contig3_All, CL4401.Contig4_All, CL4410.Contig1_All, CL4511.Contig1_All, CL4844.Contig1_All, CL4844.Contig2_All, CL4882.Contig1_All, CL4882.Contig2_All, CL5152.Contig1_All, CL5221.Contig1_All, CL5268.Contig2_All, CL5268.Contig3_All, CL531.Contig1_All, CL5373.Contig1_All, CL5373.Contig2_All, CL5440.Contig1_All, CL5464.Contig1_All, CL5464.Contig2_All, CL5464.Contig3_All, CL677.Contig2_All, CL679.Contig1_All, CL679.Contig2_All, CL703.Contig2_All, CL703.Contig3_All, CL703.Contig5_All, CL703.Contig6_All, CL80.Contig1_All, Unigene10007_All, Unigene10012_All, Unigene10093_All, Unigene10095_All, Unigene10096_All, Unigene10123_All, Unigene10171_All, Unigene10200_All, Unigene10238_All, Unigene10254_All, Unigene10316_All, Unigene1051_All, Unigene10594_All, Unigene10623_All, Unigene10705_All, Unigene10723_All, Unigene10724_All, Unigene10779_All, Unigene10787_All, Unigene108_All, Unigene10931_All, Unigene1096_All, Unigene10974_All, Unigene10983_All, Unigene10984_All, Unigene11097_All, Unigene11141_All, Unigene11160_All, Unigene11190_All, Unigene11203_All, Unigene11337_All, Unigene11338_All, Unigene11351_All, Unigene11358_All, Unigene11362_All, Unigene11377_All, Unigene11436_All, Unigene11447_All, Unigene11448_All, Unigene11469_All, Unigene11480_All, Unigene11482_All, Unigene11483_All, Unigene11510_All, Unigene11511_All, Unigene11515_All, Unigene11538_All, Unigene11543_All, Unigene11544_All, Unigene11565_All, Unigene11576_All, Unigene11587_All, Unigene11588_All, Unigene11589_All, Unigene11590_All, Unigene11629_All, Unigene11634_All, Unigene11648_All, Unigene11649_All, Unigene11660_All, Unigene11662_All, Unigene11672_All, Unigene11687_All, Unigene11797_All, Unigene1185_All, Unigene11985_All, Unigene12118_All, Unigene12125_All, Unigene12154_All, Unigene12155_All, Unigene12189_All, Unigene12194_All, Unigene12210_All, Unigene12212_All, Unigene12435_All, Unigene12533_All, Unigene12537_All, Unigene12634_All, Unigene12692_All, Unigene12710_All, Unigene12962_All, Unigene12974_All, Unigene14084_All, Unigene140_All, Unigene14133_All, Unigene1445_All, Unigene15580_All, Unigene15741_All, Unigene15769_All, Unigene16727_All, Unigene16840_All, Unigene1714_All, Unigene17380_All, Unigene17679_All, Unigene1792_All, Unigene17992_All, Unigene18002_All, Unigene18371_All, Unigene18672_All, Unigene1926_All, Unigene19_All, Unigene20176_All, Unigene2067_All, Unigene20_All, Unigene2170_All, Unigene2180_All, Unigene2190_All, Unigene2251_All, Unigene2297_All, Unigene2560_All, Unigene266_All, Unigene284_All, Unigene28_All, Unigene3000_All, Unigene3010_All, Unigene3063_All, Unigene3066_All, Unigene3193_All, Unigene3199_All, Unigene3261_All, Unigene3316_All, Unigene3323_All, Unigene3346_All, Unigene3403_All, Unigene3669_All, Unigene3889_All, Unigene3947_All, Unigene3964_All, Unigene4004_All, Unigene414_All, Unigene4310_All, Unigene4323_All, Unigene4418_All, Unigene4510_All, Unigene462_All, Unigene4825_All, Unigene4908_All, Unigene4922_All, Unigene5054_All, Unigene5059_All, Unigene5065_All, Unigene5096_All, Unigene5108_All, Unigene5121_All, Unigene5257_All, Unigene5453_All, Unigene5490_All, Unigene563_All, Unigene5724_All, Unigene5799_All, Unigene579_All, Unigene5877_All, Unigene5889_All, Unigene5890_All, Unigene590_All, Unigene6076_All, Unigene6215_All, Unigene6780_All, Unigene6886_All, Unigene6914_All, Unigene7054_All, Unigene7106_All, Unigene712_All, Unigene7206_All, Unigene7280_All, Unigene7431_All, Unigene7440_All, Unigene7534_All, Unigene7560_All, Unigene7665_All, Unigene7873_All, Unigene7926_All, Unigene7957_All, Unigene8071_All, Unigene8102_All, Unigene8170_All, Unigene8179_All, Unigene8204_All, Unigene8221_All, Unigene8231_All, Unigene8262_All, Unigene8324_All, Unigene8399_All, Unigene8514_All, Unigene8537_All, Unigene8562_All, Unigene8745_All, Unigene8848_All, Unigene8936_All, Unigene8962_All, Unigene9163_All, Unigene9284_All, Unigene936_All, Unigene9611_All, Unigene96_All, Unigene9988_All

50	Glutamatergic synapse	CL1069.Contig1_All, CL1069.Contig2_All, CL1069.Contig3_All, CL1069.Contig4_All, CL1069.Contig5_All, CL1069.Contig6_All, CL1069.Contig7_All, CL1069.Contig8_All, CL1452.Contig1_All, CL155.Contig1_All, CL155.Contig2_All, CL1675.Contig2_All, CL18.Contig1_All, CL199.Contig1_All, CL2182.Contig1_All, CL2182.Contig3_All, CL2182.Contig4_All, CL2432.Contig2_All, CL2930.Contig5_All, CL3118.Contig1_All, CL3118.Contig2_All, CL3283.Contig1_All, CL3562.Contig6_All, CL3886.Contig1_All, CL3886.Contig2_All, CL3886.Contig3_All, CL3886.Contig4_All, CL39.Contig1_All, CL39.Contig2_All, CL39.Contig3_All, CL39.Contig4_All, CL4005.Contig2_All, CL4005.Contig3_All, CL4262.Contig2_All, CL440.Contig3_All, CL440.Contig5_All, CL440.Contig6_All, CL4521.Contig1_All, CL4521.Contig2_All, CL4521.Contig3_All, CL4521.Contig5_All, CL4923.Contig2_All, CL5064.Contig2_All, CL5098.Contig2_All, CL5310.Contig2_All, CL5391.Contig1_All, CL5391.Contig2_All, CL5399.Contig1_All, CL5399.Contig2_All, CL567.Contig4_All, CL691.Contig1_All, CL860.Contig10_All, CL860.Contig8_All, CL860.Contig9_All, CL870.Contig4_All, CL870.Contig8_All, CL870.Contig9_All, CL940.Contig3_All, CL940.Contig5_All, Unigene10058_All, Unigene10412_All, Unigene10909_All, Unigene10910_All, Unigene11915_All, Unigene12110_All, Unigene12470_All, Unigene13853_All, Unigene1417_All, Unigene1743_All, Unigene176_All, Unigene18234_All, Unigene18690_All, Unigene18898_All, Unigene18993_All, Unigene19148_All, Unigene1925_All, Unigene19288_All, Unigene2029_All, Unigene2196_All, Unigene2209_All, Unigene3029_All, Unigene3033_All, Unigene3034_All, Unigene3036_All, Unigene3037_All, Unigene3042_All, Unigene3655_All, Unigene3779_All, Unigene4053_All, Unigene4070_All, Unigene4112_All, Unigene4126_All, Unigene4532_All, Unigene4780_All, Unigene5074_All, Unigene5261_All, Unigene5521_All, Unigene555_All, Unigene6217_All, Unigene6242_All, Unigene661_All, Unigene69_All, Unigene7018_All, Unigene807_All, Unigene8091_All, Unigene8422_All, Unigene855_All, Unigene8621_All, Unigene9015_All, Unigene93_All, Unigene9437_All, Unigene9733_All
51	Toxoplasmosis	CL1054.Contig1_All, CL1054.Contig2_All, CL1066.Contig1_All, CL1066.Contig2_All, CL1066.Contig3_All, CL1495.Contig1_All, CL1495.Contig2_All, CL1925.Contig1_All, CL199.Contig1_All, CL2129.Contig4_All, CL2165.Contig2_All, CL2331.Contig2_All, CL2331.Contig3_All, CL2432.Contig2_All, CL2686.Contig1_All, CL2686.Contig2_All, CL2882.Contig1_All, CL2882.Contig2_All, CL3554.Contig1_All, CL367.Contig1_All, CL367.Contig2_All, CL367.Contig3_All, CL39.Contig1_All, CL39.Contig2_All, CL39.Contig3_All, CL39.Contig4_All, CL470.Contig1_All, CL4923.Contig2_All, CL4966.Contig1_All, CL4992.Contig1_All, CL5048.Contig1_All, CL5115.Contig1_All, CL5256.Contig1_All, CL5256.Contig2_All, CL5256.Contig4_All, CL5310.Contig2_All, CL5352.Contig1_All, CL5352.Contig2_All, CL558.Contig1_All, CL558.Contig2_All, CL558.Contig4_All, CL695.Contig16_All, CL860.Contig10_All, CL860.Contig8_All, CL860.Contig9_All, Unigene10837_All, Unigene11724_All, Unigene12139_All, Unigene12188_All, Unigene12331_All, Unigene1326_All, Unigene15587_All, Unigene15658_All, Unigene15840_All, Unigene15855_All, Unigene15863_All, Unigene16093_All, Unigene1614_All, Unigene16538_All, Unigene16965_All, Unigene16966_All, Unigene1743_All, Unigene1757_All, Unigene17640_All, Unigene176_All, Unigene17702_All, Unigene17728_All, Unigene17929_All, Unigene18488_All, Unigene18496_All, Unigene18535_All, Unigene19237_All, Unigene19426_All, Unigene19840_All, Unigene20095_All, Unigene20226_All, Unigene2196_All, Unigene220_All, Unigene2503_All, Unigene2749_All, Unigene3029_All, Unigene3033_All, Unigene3034_All, Unigene3036_All, Unigene3037_All, Unigene3042_All, Unigene3123_All, Unigene3131_All, Unigene3945_All, Unigene4126_All, Unigene4207_All, Unigene4218_All, Unigene4581_All, Unigene5169_All, Unigene5521_All, Unigene6217_All, Unigene69_All, Unigene7220_All, Unigene8246_All, Unigene9971_All
52	Retrograde endocannabinoid signaling	CL1069.Contig1_All, CL1069.Contig2_All, CL1069.Contig3_All, CL1069.Contig4_All, CL1069.Contig5_All, CL1069.Contig6_All, CL1069.Contig7_All, CL1069.Contig8_All, CL1425.Contig2_All, CL1425.Contig5_All, CL1452.Contig1_All, CL1675.Contig2_All, CL1707.Contig1_All, CL2161.Contig1_All, CL2161.Contig2_All, CL2182.Contig1_All, CL2182.Contig3_All, CL2182.Contig4_All, CL225.Contig1_All, CL225.Contig3_All, CL2703.Contig2_All, CL2726.Contig1_All, CL2735.Contig1_All, CL2878.Contig3_All, CL2930.Contig5_All, CL3166.Contig1_All, CL3507.Contig1_All, CL3643.Contig2_All, CL3643.Contig6_All, CL39.Contig1_All, CL39.Contig2_All, CL39.Contig3_All, CL39.Contig4_All, CL4005.Contig2_All, CL4005.Contig3_All, CL4205.Contig1_All, CL4205.Contig2_All, CL4262.Contig2_All, CL440.Contig3_All, CL440.Contig5_All, CL440.Contig6_All, CL4600.Contig2_All, CL4923.Contig2_All, CL4966.Contig1_All, CL5098.Contig2_All, CL5105.Contig1_All, CL5105.Contig2_All, CL5115.Contig1_All, CL5288.Contig2_All, CL5391.Contig1_All, CL5391.Contig2_All, CL5399.Contig1_All, CL5399.Contig2_All, CL662.Contig1_All, CL677.Contig2_All, CL679.Contig1_All, CL679.Contig2_All, CL691.Contig1_All, CL847.Contig3_All, CL894.Contig1_All, CL90.Contig1_All, CL90.Contig2_All, CL940.Contig3_All, CL940.Contig5_All, CL973.Contig1_All, Unigene1044_All, Unigene10931_All, Unigene11615_All, Unigene12110_All, Unigene12433_All, Unigene12535_All, Unigene13612_All, Unigene16890_All, Unigene1743_All, Unigene1766_All, Unigene176_All, Unigene17896_All, Unigene18898_All, Unigene18993_All, Unigene1925_All, Unigene19288_All, Unigene2029_All, Unigene2032_All, Unigene2196_All, Unigene2209_All, Unigene220_All, Unigene3134_All, Unigene3779_All, Unigene4053_All, Unigene4070_All, Unigene4112_All, Unigene4126_All, Unigene4780_All, Unigene5433_All, Unigene5521_All, Unigene6134_All, Unigene6217_All, Unigene6242_All, Unigene68_All, Unigene69_All, Unigene8071_All, Unigene807_All, Unigene8170_All, Unigene8422_All, Unigene8621_All, Unigene8993_All, Unigene9060_All, Unigene9167_All, Unigene93_All, Unigene9437_All, Unigene9733_All

53	Arrhythmogenic right ventricular cardiomyopathy (ARVC)	CL1075.Contig2_All, CL1075.Contig3_All, CL1079.Contig4_All, CL1087.Contig2_All, CL1087.Contig3_All, CL1355.Contig1_All, CL1503.Contig3_All, CL1503.Contig4_All, CL1503.Contig5_All, CL156.Contig1_All, CL156.Contig3_All, CL156.Contig4_All, CL1803.Contig1_All, CL2034.Contig1_All, CL2034.Contig2_All, CL2331.Contig2_All, CL2331.Contig3_All, CL2517.Contig4_All, CL3436.Contig1_All, CL3436.Contig4_All, CL3436.Contig9_All, CL3442.Contig1_All, CL3554.Contig1_All, CL470.Contig1_All, CL4992.Contig1_All, CL531.Contig1_All, CL5352.Contig1_All, CL5352.Contig2_All, CL5468.Contig3_All, CL642.Contig1_All, CL642.Contig2_All, CL703.Contig2_All, CL703.Contig3_All, CL703.Contig5_All, CL703.Contig6_All, CL923.Contig3_All, Unigene10040_All, Unigene10471_All, Unigene11597_All, Unigene11598_All, Unigene11599_All, Unigene12627_All, Unigene12684_All, Unigene13092_All, Unigene136_All, Unigene15824_All, Unigene18598_All, Unigene18873_All, Unigene2032_All, Unigene2503_All, Unigene2741_All, Unigene278_All, Unigene3187_All, Unigene3945_All, Unigene4207_All, Unigene5727_All, Unigene7096_All, Unigene777_All, Unigene783_All, Unigene8621_All, Unigene9042_All, Unigene9062_All, Unigene9740_All
54	Type I diabetes mellitus	CL1599.Contig1_All, CL1599.Contig2_All, Unigene10337_All, Unigene10749_All, Unigene11919_All, Unigene20260_All, Unigene6768_All, Unigene9726_All
55	Neuroactive ligand-receptor interaction	CL1085.Contig2_All, CL1200.Contig2_All, CL1200.Contig4_All, CL1203.Contig5_All, CL1419.Contig7_All, CL1469.Contig1_All, CL1469.Contig2_All, CL1525.Contig14_All, CL1525.Contig15_All, CL1525.Contig9_All, CL18.Contig1_All, CL1849.Contig1_All, CL1849.Contig2_All, CL1849.Contig3_All, CL1849.Contig4_All, CL187.Contig3_All, CL1887.Contig1_All, CL1887.Contig2_All, CL1887.Contig3_All, CL191.Contig1_All, CL191.Contig2_All, CL1920.Contig1_All, CL1920.Contig2_All, CL2016.Contig1_All, CL2018.Contig3_All, CL2055.Contig1_All, CL2055.Contig3_All, CL214.Contig3_All, CL2146.Contig4_All, CL2161.Contig1_All, CL2161.Contig2_All, CL2213.Contig1_All, CL2213.Contig2_All, CL2213.Contig4_All, CL225.Contig1_All, CL225.Contig2_All, CL225.Contig3_All, CL2276.Contig1_All, CL2276.Contig2_All, CL2703.Contig2_All, CL2734.Contig1_All, CL2735.Contig1_All, CL2762.Contig4_All, CL2992.Contig1_All, CL2993.Contig1_All, CL3007.Contig2_All, CL3166.Contig1_All, CL3271.Contig1_All, CL3271.Contig2_All, CL336.Contig1_All, CL336.Contig2_All, CL3362.Contig1_All, CL3362.Contig2_All, CL3481.Contig1_All, CL3507.Contig1_All, CL3545.Contig1_All, CL3545.Contig2_All, CL3571.Contig1_All, CL3571.Contig2_All, CL3575.Contig1_All, CL3575.Contig2_All, CL3597.Contig1_All, CL3597.Contig2_All, CL3676.Contig1_All, CL3679.Contig2_All, CL393.Contig2_All, CL3948.Contig1_All, CL3948.Contig3_All, CL398.Contig4_All, CL4004.Contig1_All, CL4004.Contig2_All, CL4065.Contig1_All, CL4169.Contig1_All, CL4362.Contig1_All, CL4362.Contig2_All, CL4442.Contig1_All, CL4473.Contig1_All, CL4473.Contig2_All, CL4473.Contig3_All, CL4533.Contig1_All, CL4645.Contig1_All, CL467.Contig1_All, CL467.Contig2_All, CL4684.Contig1_All, CL4699.Contig1_All, CL4727.Contig2_All, CL4754.Contig1_All, CL4786.Contig1_All, CL4919.Contig1_All, CL4928.Contig1_All, CL4928.Contig2_All, CL4997.Contig1_All, CL4997.Contig2_All, CL5098.Contig2_All, CL5105.Contig1_All, CL5105.Contig2_All, CL526.Contig2_All, CL5287.Contig2_All, CL5406.Contig1_All, CL5458.Contig5_All, CL5458.Contig7_All, CL584.Contig17_All, CL595.Contig6_All, CL600.Contig1_All, CL600.Contig2_All, CL600.Contig3_All, CL600.Contig4_All, CL600.Contig6_All, CL622.Contig1_All, CL622.Contig2_All, CL622.Contig4_All, CL662.Contig1_All, CL763.Contig1_All, CL763.Contig2_All, CL801.Contig1_All, CL801.Contig2_All, CL801.Contig3_All, CL801.Contig4_All, CL801.Contig7_All, CL801.Contig8_All, CL801.Contig9_All, CL847.Contig3_All, CL870.Contig4_All, CL870.Contig8_All, CL870.Contig9_All, CL894.Contig1_All, CL894.Contig2_All, CL973.Contig1_All, CL982.Contig4_All, Unigene1007_All, Unigene10763_All, Unigene1099_All, Unigene1111_All, Unigene11357_All, Unigene11615_All, Unigene11660_All, Unigene1186_All, Unigene11915_All, Unigene12398_All, Unigene12535_All, Unigene12722_All, Unigene13612_All, Unigene1417_All, Unigene1427_All, Unigene142_All, Unigene15194_All, Unigene15195_All, Unigene16349_All, Unigene1671_All, Unigene17380_All, Unigene1766_All, Unigene17812_All, Unigene17864_All, Unigene17896_All, Unigene17987_All, Unigene18002_All, Unigene18066_All, Unigene18199_All, Unigene18431_All, Unigene18653_All, Unigene18654_All, Unigene18672_All, Unigene18690_All, Unigene19148_All, Unigene19288_All, Unigene19782_All, Unigene197_All, Unigene20226_All, Unigene2027_All, Unigene2058_All, Unigene2061_All, Unigene2139_All, Unigene213_All, Unigene2172_All, Unigene217_All, Unigene2616_All, Unigene2701_All, Unigene29_All, Unigene3126_All, Unigene3283_All, Unigene3357_All, Unigene3748_All, Unigene3967_All, Unigene4073_All, Unigene4092_All, Unigene4129_All, Unigene5045_All, Unigene5066_All, Unigene5074_All, Unigene5128_All, Unigene5147_All, Unigene5158_All, Unigene5213_All, Unigene5261_All, Unigene5273_All, Unigene5877_All, Unigene590_All, Unigene6242_All, Unigene661_All, Unigene68_All, Unigene6972_All, Unigene7020_All, Unigene7042_All, Unigene7110_All, Unigene7217_All, Unigene7265_All, Unigene75_All, Unigene8019_All, Unigene8038_All, Unigene8052_All, Unigene807_All, Unigene8091_All, Unigene8210_All, Unigene8331_All, Unigene84_All, Unigene8770_All, Unigene8848_All, Unigene8993_All, Unigene9036_All, Unigene9037_All, Unigene9117_All, Unigene9167_All, Unigene9205_All

56	Influenza A	CL1054.Contig1_All, CL1054.Contig2_All, CL1066.Contig1_All, CL1066.Contig2_All, CL1066.Contig3_All, CL107.Contig3_All, CL1355.Contig1_All, CL156.Contig1_All, CL156.Contig3_All, CL156.Contig4_All, CL1803.Contig1_All, CL1843.Contig3_All, CL1925.Contig1_All, CL1966.Contig2_All, CL1991.Contig2_All, CL1991.Contig3_All, CL1991.Contig4_All, CL2049.Contig1_All, CL2049.Contig3_All, CL2129.Contig4_All, CL2165.Contig2_All, CL2170.Contig2_All, CL2170.Contig5_All, CL2182.Contig1_All, CL2182.Contig3_All, CL2182.Contig4_All, CL2213.Contig1_All, CL2213.Contig2_All, CL2213.Contig4_All, CL2229.Contig1_All, CL2306.Contig2_All, CL2420.Contig1_All, CL2420.Contig2_All, CL2490.Contig1_All, CL2686.Contig1_All, CL2686.Contig2_All, CL2761.Contig1_All, CL2761.Contig2_All, CL2789.Contig2_All, CL2882.Contig1_All, CL2882.Contig2_All, CL345.Contig1_All, CL367.Contig1_All, CL367.Contig2_All, CL367.Contig3_All, CL3798.Contig3_All, CL39.Contig1_All, CL39.Contig2_All, CL39.Contig3_All, CL39.Contig4_All, CL4010.Contig1_All, CL4010.Contig2_All, CL4105.Contig2_All, CL4155.Contig1_All, CL4604.Contig1_All, CL4604.Contig2_All, CL4736.Contig1_All, CL4736.Contig2_All, CL475.Contig4_All, CL4787.Contig1_All, CL4815.Contig1_All, CL4815.Contig2_All, CL4874.Contig1_All, CL4966.Contig1_All, CL5051.Contig2_All, CL5115.Contig1_All, CL5256.Contig1_All, CL5256.Contig2_All, CL5256.Contig4_All, CL5391.Contig1_All, CL5391.Contig2_All, CL5468.Contig3_All, CL558.Contig1_All, CL558.Contig2_All, CL558.Contig4_All, CL560.Contig1_All, CL560.Contig2_All, CL560.Contig4_All, CL695.Contig16_All, CL910.Contig1_All, CL910.Contig6_All, CL910.Contig7_All, CL910.Contig9_All, CL940.Contig3_All, CL940.Contig5_All, Unigene10234_All, Unigene10817_All, Unigene10833_All, Unigene10837_All, Unigene1084_All, Unigene11422_All, Unigene11597_All, Unigene11598_All, Unigene11599_All, Unigene11660_All, Unigene11724_All, Unigene11922_All, Unigene12006_All, Unigene12088_All, Unigene12139_All, Unigene12188_All, Unigene12259_All, Unigene1247_All, Unigene12608_All, Unigene12618_All, Unigene12669_All, Unigene12684_All, Unigene13010_All, Unigene13092_All, Unigene1326_All, Unigene13541_All, Unigene14208_All, Unigene1459_All, Unigene1479_All, Unigene15587_All, Unigene15863_All, Unigene16093_All, Unigene17046_All, Unigene17380_All, Unigene1757_All, Unigene17702_All, Unigene18002_All, Unigene18278_All, Unigene18284_All, Unigene18535_All, Unigene18598_All, Unigene18672_All, Unigene18898_All, Unigene19116_All, Unigene20095_All, Unigene220_All, Unigene236_All, Unigene247_All, Unigene2741_All, Unigene2749_All, Unigene278_All, Unigene3415_All, Unigene453_All, Unigene4581_All, Unigene54_All, Unigene5781_All, Unigene5877_All, Unigene590_All, Unigene6648_All, Unigene6977_All, Unigene7286_All, Unigene7297_All, Unigene7702_All, Unigene8174_All, Unigene8246_All, Unigene8605_All, Unigene8784_All, Unigene8848_All, Unigene9971_All, Unigene9989_All
57	Gap junction	CL126.Contig2_All, CL1452.Contig1_All, CL1548.Contig2_All, CL1548.Contig3_All, CL1559.Contig1_All, CL1807.Contig1_All, CL1807.Contig2_All, CL2182.Contig1_All, CL2182.Contig3_All, CL2182.Contig4_All, CL2930.Contig5_All, CL3311.Contig1_All, CL3311.Contig2_All, CL39.Contig1_All, CL39.Contig2_All, CL39.Contig3_All, CL39.Contig4_All, CL3948.Contig3_All, CL4005.Contig2_All, CL4005.Contig3_All, CL4181.Contig1_All, CL4181.Contig4_All, CL4262.Contig2_All, CL440.Contig3_All, CL440.Contig5_All, CL440.Contig6_All, CL4439.Contig2_All, CL4684.Contig1_All, CL4776.Contig1_All, CL4776.Contig2_All, CL4787.Contig1_All, CL5098.Contig2_All, CL5149.Contig1_All, CL5149.Contig2_All, CL5186.Contig2_All, CL5207.Contig2_All, CL5391.Contig1_All, CL5391.Contig2_All, CL5399.Contig1_All, CL5399.Contig2_All, CL621.Contig2_All, CL621.Contig4_All, CL621.Contig5_All, CL648.Contig2_All, CL691.Contig1_All, CL940.Contig3_All, CL940.Contig5_All, Unigene10058_All, Unigene1007_All, Unigene10125_All, Unigene10126_All, Unigene10128_All, Unigene1016_All, Unigene10174_All, Unigene1017_All, Unigene1050_All, Unigene1099_All, Unigene11121_All, Unigene11130_All, Unigene11369_All, Unigene11395_All, Unigene11730_All, Unigene11992_All, Unigene12161_All, Unigene12203_All, Unigene12671_All, Unigene13421_All, Unigene15176_All, Unigene15308_All, Unigene1671_All, Unigene17987_All, Unigene18898_All, Unigene18993_All, Unigene19288_All, Unigene19920_All, Unigene2029_All, Unigene2209_All, Unigene2795_All, Unigene3715_All, Unigene3779_All, Unigene3861_All, Unigene4053_All, Unigene4070_All, Unigene4112_All, Unigene4133_All, Unigene420_All, Unigene4532_All, Unigene4765_All, Unigene4783_All, Unigene5448_All, Unigene5521_All, Unigene5896_All, Unigene615_All, Unigene6217_All, Unigene6242_All, Unigene699_All, Unigene7767_All, Unigene8046_All, Unigene807_All, Unigene8205_All, Unigene8422_All, Unigene9217_All, Unigene93_All, Unigene9437_All, Unigene9604_All, Unigene9733_All, Unigene9972_All, Unigene9973_All, Unigene9984_All
58	Shigellosis	CL1099.Contig3_All, CL1355.Contig1_All, CL1535.Contig3_All, CL156.Contig1_All, CL156.Contig3_All, CL156.Contig4_All, CL1723.Contig1_All, CL1723.Contig2_All, CL1723.Contig7_All, CL1803.Contig1_All, CL2.Contig1_All, CL2085.Contig1_All, CL2224.Contig2_All, CL2224.Contig3_All, CL2224.Contig4_All, CL2375.Contig2_All, CL271.Contig2_All, CL2764.Contig2_All, CL2833.Contig1_All, CL2838.Contig1_All, CL2838.Contig2_All, CL2838.Contig4_All, CL2975.Contig2_All, CL3027.Contig3_All, CL3114.Contig2_All, CL3315.Contig3_All, CL3315.Contig4_All, CL3510.Contig3_All, CL3823.Contig1_All, CL3823.Contig2_All, CL402.Contig1_All, CL4088.Contig2_All, CL4098.Contig3_All, CL4130.Contig1_All, CL4203.Contig3_All, CL4479.Contig2_All, CL4479.Contig3_All, CL4479.Contig5_All, CL4738.Contig1_All, CL4966.Contig1_All, CL5115.Contig1_All, CL5162.Contig1_All, CL5162.Contig2_All, CL5468.Contig3_All, Unigene10097_All, Unigene10448_All, Unigene10998_All, Unigene11082_All, Unigene11372_All, Unigene11459_All, Unigene11521_All, Unigene11597_All, Unigene11598_All, Unigene11599_All, Unigene11677_All, Unigene12201_All, Unigene12603_All, Unigene12684_All, Unigene12711_All, Unigene13036_All, Unigene13092_All, Unigene13147_All, Unigene1447_All, Unigene1496_All, Unigene16840_All, Unigene16963_All, Unigene1757_All, Unigene18598_All, Unigene18644_All, Unigene198_All, Unigene220_All, Unigene2741_All, Unigene278_All, Unigene3482_All, Unigene3945_All, Unigene4207_All, Unigene5303_All, Unigene5873_All, Unigene6014_All, Unigene6029_All, Unigene6994_All, Unigene7058_All, Unigene7063_All, Unigene7983_All, Unigene798_All, Unigene8184_All, Unigene9785_All, Unigene9787_All, Unigene9902_All, Unigene9947_All

59	ErbB signaling pathway	CL1066.Contig1_All, CL1066.Contig2_All, CL1066.Contig3_All, CL1548.Contig2_All, CL1548.Contig3_All, CL1807.Contig1_All, CL1807.Contig2_All, CL1843.Contig3_All, CL1874.Contig2_All, CL1953.Contig1_All, CL1966.Contig2_All, CL2038.Contig1_All, CL2038.Contig7_All, CL2066.Contig1_All, CL2182.Contig1_All, CL2182.Contig3_All, CL2182.Contig4_All, CL2722.Contig1_All, CL2977.Contig1_All, CL2977.Contig2_All, CL3632.Contig2_All, CL3632.Contig3_All, CL367.Contig1_All, CL367.Contig2_All, CL367.Contig3_All, CL3796.Contig2_All, CL3796.Contig3_All, CL39.Contig1_All, CL39.Contig2_All, CL39.Contig3_All, CL39.Contig4_All, CL3979.Contig1_All, CL3979.Contig2_All, CL400.Contig1_All, CL400.Contig2_All, CL44.Contig10_All, CL44.Contig12_All, CL44.Contig13_All, CL44.Contig16_All, CL44.Contig17_All, CL44.Contig18_All, CL44.Contig19_All, CL44.Contig1_All, CL44.Contig22_All, CL44.Contig23_All, CL44.Contig2_All, CL44.Contig3_All, CL44.Contig4_All, CL44.Contig5_All, CL44.Contig6_All, CL44.Contig8_All, CL44.Contig9_All, CL4439.Contig2_All, CL4559.Contig2_All, CL4559.Contig3_All, CL4736.Contig1_All, CL4736.Contig2_All, CL4787.Contig1_All, CL4815.Contig1_All, CL4815.Contig2_All, CL5005.Contig1_All, CL5005.Contig2_All, CL5115.Contig1_All, CL5391.Contig1_All, CL5391.Contig2_All, CL5469.Contig2_All, CL695.Contig16_All, CL748.Contig1_All, CL748.Contig3_All, CL940.Contig3_All, CL940.Contig5_All, Unigene10174_All, Unigene11446_All, Unigene11922_All, Unigene12671_All, Unigene13036_All, Unigene1479_All, Unigene18898_All, Unigene198_All, Unigene220_All, Unigene236_All, Unigene247_All, Unigene2749_All, Unigene2956_All, Unigene3168_All, Unigene4081_All, Unigene6101_All, Unigene6977_All, Unigene699_All, Unigene7016_All, Unigene7297_All, Unigene7745_All, Unigene7983_All, Unigene8046_All, Unigene8725_All, Unigene8747_All, Unigene8784_All, Unigene9719_All, Unigene9921_All
60	Fc gamma R-mediated phagocytosis	CL1066.Contig1_All, CL1066.Contig2_All, CL1066.Contig3_All, CL1535.Contig3_All, CL1723.Contig1_All, CL1723.Contig2_All, CL1723.Contig7_All, CL1954.Contig1_All, CL1954.Contig2_All, CL199.Contig1_All, CL2182.Contig1_All, CL2182.Contig3_All, CL2182.Contig4_All, CL2375.Contig2_All, CL2432.Contig2_All, CL271.Contig2_All, CL2975.Contig2_All, CL3027.Contig3_All, CL3047.Contig1_All, CL3047.Contig2_All, CL309.Contig2_All, CL309.Contig3_All, CL309.Contig4_All, CL3114.Contig2_All, CL3315.Contig3_All, CL3315.Contig4_All, CL344.Contig1_All, CL3510.Contig3_All, CL367.Contig1_All, CL367.Contig2_All, CL367.Contig3_All, CL39.Contig1_All, CL39.Contig2_All, CL39.Contig3_All, CL39.Contig4_All, CL3979.Contig1_All, CL3979.Contig2_All, CL399.Contig10_All, CL399.Contig1_All, CL399.Contig2_All, CL399.Contig3_All, CL399.Contig4_All, CL399.Contig5_All, CL399.Contig6_All, CL399.Contig7_All, CL399.Contig8_All, CL399.Contig9_All, CL4088.Contig2_All, CL4130.Contig1_All, CL4541.Contig1_All, CL4541.Contig2_All, CL4787.Contig1_All, CL4845.Contig1_All, CL4855.Contig1_All, CL4855.Contig2_All, CL495.Contig11_All, CL495.Contig1_All, CL495.Contig3_All, CL495.Contig4_All, CL495.Contig5_All, CL495.Contig6_All, CL5017.Contig2_All, CL5310.Contig2_All, CL5391.Contig1_All, CL5391.Contig2_All, CL5469.Contig2_All, CL695.Contig16_All, CL860.Contig10_All, CL860.Contig8_All, CL860.Contig9_All, CL940.Contig3_All, CL940.Contig5_All, Unigene10097_All, Unigene10448_All, Unigene10775_All, Unigene10998_All, Unigene11052_All, Unigene11082_All, Unigene11459_All, Unigene11521_All, Unigene11628_All, Unigene12201_All, Unigene12301_All, Unigene12603_All, Unigene13036_All, Unigene1447_All, Unigene16840_All, Unigene16963_All, Unigene18898_All, Unigene2749_All, Unigene2956_All, Unigene3029_All, Unigene3033_All, Unigene3034_All, Unigene3036_All, Unigene3037_All, Unigene3042_All, Unigene3482_All, Unigene4810_All, Unigene5303_All, Unigene5873_All, Unigene6014_All, Unigene6029_All, Unigene7018_All, Unigene7348_All, Unigene7745_All, Unigene7936_All, Unigene8056_All, Unigene8184_All, Unigene8585_All, Unigene9719_All, Unigene9785_All, Unigene9787_All, Unigene9902_All, Unigene9947_All
61	Synaptic vesicle cycle	CL1069.Contig1_All, CL1069.Contig2_All, CL1069.Contig3_All, CL1069.Contig4_All, CL1069.Contig5_All, CL1069.Contig6_All, CL1069.Contig7_All, CL1069.Contig8_All, CL182.Contig10_All, CL182.Contig11_All, CL182.Contig1_All, CL182.Contig2_All, CL182.Contig3_All, CL182.Contig4_All, CL182.Contig5_All, CL182.Contig6_All, CL182.Contig7_All, CL182.Contig8_All, CL182.Contig9_All, CL2019.Contig1_All, CL2019.Contig2_All, CL2068.Contig5_All, CL2108.Contig1_All, CL2108.Contig4_All, CL2150.Contig3_All, CL2382.Contig4_All, CL2450.Contig1_All, CL2450.Contig2_All, CL2634.Contig1_All, CL2634.Contig2_All, CL3091.Contig1_All, CL3091.Contig2_All, CL3091.Contig3_All, CL3091.Contig4_All, CL3200.Contig2_All, CL3226.Contig1_All, CL3226.Contig2_All, CL3286.Contig1_All, CL399.Contig10_All, CL399.Contig1_All, CL399.Contig2_All, CL399.Contig3_All, CL399.Contig4_All, CL399.Contig5_All, CL399.Contig6_All, CL399.Contig7_All, CL399.Contig8_All, CL399.Contig9_All, CL4001.Contig1_All, CL4001.Contig2_All, CL4087.Contig2_All, CL4090.Contig2_All, CL4197.Contig2_All, CL42.Contig2_All, CL4459.Contig1_All, CL4541.Contig1_All, CL4541.Contig2_All, CL4644.Contig2_All, CL4746.Contig1_All, CL495.Contig11_All, CL495.Contig1_All, CL495.Contig3_All, CL495.Contig4_All, CL495.Contig5_All, CL495.Contig6_All, CL677.Contig2_All, CL679.Contig1_All, CL679.Contig2_All, Unigene10026_All, Unigene10078_All, Unigene1044_All, Unigene10483_All, Unigene10931_All, Unigene11240_All, Unigene11899_All, Unigene1189_All, Unigene11973_All, Unigene12028_All, Unigene12168_All, Unigene12209_All, Unigene12302_All, Unigene12423_All, Unigene12433_All, Unigene12923_All, Unigene13225_All, Unigene13508_All, Unigene13589_All, Unigene13978_All, Unigene15969_All, Unigene16890_All, Unigene1741_All, Unigene17602_All, Unigene18707_All, Unigene2032_All, Unigene2193_All, Unigene3137_All, Unigene3351_All, Unigene3730_All, Unigene4299_All, Unigene5360_All, Unigene5716_All, Unigene6005_All, Unigene6697_All, Unigene6702_All, Unigene7298_All, Unigene7751_All, Unigene8071_All, Unigene8170_All, Unigene9216_All, Unigene925_All

62	mRNA surveillance pathway	CL1477.Contig8_All, CL1563.Contig2_All, CL1637.Contig1_All, CL1663.Contig2_All, CL1740.Contig4_All, CL1850.Contig1_All, CL1850.Contig2_All, CL2154.Contig1_All, CL2154.Contig2_All, CL2278.Contig1_All, CL2278.Contig2_All, CL2400.Contig5_All, CL244.Contig2_All, CL2496.Contig1_All, CL2502.Contig2_All, CL2502.Contig3_All, CL2657.Contig2_All, CL2719.Contig1_All, CL2719.Contig2_All, CL276.Contig3_All, CL276.Contig7_All, CL2761.Contig1_All, CL2761.Contig2_All, CL2873.Contig1_All, CL2873.Contig2_All, CL296.Contig4_All, CL3057.Contig1_All, CL3057.Contig2_All, CL3221.Contig1_All, CL3401.Contig2_All, CL361.Contig1_All, CL3616.Contig1_All, CL3681.Contig1_All, CL3681.Contig2_All, CL3689.Contig2_All, CL394.Contig1_All, CL394.Contig2_All, CL4002.Contig1_All, CL4051.Contig1_All, CL4127.Contig2_All, CL4155.Contig1_All, CL4295.Contig1_All, CL4302.Contig1_All, CL4302.Contig2_All, CL4302.Contig3_All, CL4302.Contig4_All, CL4375.Contig2_All, CL452.Contig2_All, CL4667.Contig1_All, CL4667.Contig3_All, CL4794.Contig1_All, CL4794.Contig3_All, CL4884.Contig2_All, CL4884.Contig4_All, CL497.Contig1_All, CL5067.Contig2_All, CL5078.Contig12_All, CL5096.Contig2_All, CL5107.Contig1_All, CL5107.Contig2_All, CL5150.Contig1_All, CL5150.Contig4_All, CL5214.Contig1_All, CL5214.Contig2_All, CL5459.Contig1_All, CL560.Contig1_All, CL560.Contig2_All, CL560.Contig4_All, CL700.Contig1_All, CL700.Contig2_All, CL741.Contig2_All, CL761.Contig1_All, CL761.Contig2_All, Unigene10053_All, Unigene10154_All, Unigene10242_All, Unigene10357_All, Unigene10528_All, Unigene1055_All, Unigene11025_All, Unigene11092_All, Unigene11099_All, Unigene11459_All, Unigene11478_All, Unigene11479_All, Unigene11623_All, Unigene1168_All, Unigene1172_All, Unigene11807_All, Unigene11939_All, Unigene12047_All, Unigene12088_All, Unigene12124_All, Unigene12294_All, Unigene12299_All, Unigene12322_All, Unigene12410_All, Unigene12414_All, Unigene12464_All, Unigene12608_All, Unigene1260_All, Unigene12618_All, Unigene12669_All, Unigene12798_All, Unigene12866_All, Unigene12949_All, Unigene13010_All, Unigene13030_All, Unigene1313_All, Unigene13265_All, Unigene13432_All, Unigene13541_All, Unigene1512_All, Unigene1768_All, Unigene1792_All, Unigene1941_All, Unigene1975_All, Unigene19925_All, Unigene19947_All, Unigene20230_All, Unigene2203_All, Unigene2233_All, Unigene2255_All, Unigene2682_All, Unigene3415_All, Unigene3554_All, Unigene3814_All, Unigene3991_All, Unigene4022_All, Unigene4106_All, Unigene4251_All, Unigene4571_All, Unigene4749_All, Unigene4777_All, Unigene4935_All, Unigene4954_All, Unigene4970_All, Unigene5067_All, Unigene5092_All, Unigene5177_All, Unigene5303_All, Unigene5361_All, Unigene554_All, Unigene6021_All, Unigene6236_All, Unigene63_All, Unigene6587_All, Unigene6655_All, Unigene6681_All, Unigene6704_All, Unigene6739_All, Unigene6746_All, Unigene7270_All, Unigene7646_All, Unigene7704_All, Unigene7743_All, Unigene7759_All, Unigene7764_All, Unigene7776_All, Unigene7981_All, Unigene8119_All, Unigene8228_All, Unigene8235_All, Unigene8686_All, Unigene8943_All, Unigene9252_All, Unigene9710_All, Unigene9742_All, Unigene9904_All

63	Regulation of actin cytoskeleton	CL1066.Contig1_All, CL1066.Contig2_All, CL1066.Contig3_All, CL1068.Contig5_All, CL1075.Contig3_All, CL1099.Contig3_All, CL1141.Contig2_All, CL119.Contig10_All, CL119.Contig11_All, CL119.Contig3_All, CL119.Contig8_All, CL119.Contig9_All, CL1355.Contig1_All, CL147.Contig6_All, CL1503.Contig3_All, CL1503.Contig4_All, CL1503.Contig5_All, CL1535.Contig3_All, CL1548.Contig2_All, CL1548.Contig3_All, CL156.Contig1_All, CL156.Contig3_All, CL156.Contig4_All, CL1563.Contig2_All, CL1564.Contig4_All, CL1564.Contig5_All, CL1687.Contig4_All, CL1723.Contig1_All, CL1723.Contig2_All, CL1723.Contig7_All, CL1803.Contig1_All, CL1807.Contig1_All, CL1807.Contig2_All, CL1954.Contig1_All, CL1954.Contig2_All, CL2039.Contig1_All, CL2061.Contig1_All, CL2066.Contig1_All, CL2224.Contig2_All, CL2224.Contig3_All, CL2224.Contig4_All, CL2265.Contig1_All, CL2265.Contig2_All, CL2331.Contig2_All, CL2331.Contig3_All, CL2336.Contig1_All, CL2375.Contig2_All, CL244.Contig2_All, CL2449.Contig1_All, CL2449.Contig2_All, CL2505.Contig3_All, CL2505.Contig4_All, CL254.Contig1_All, CL263.Contig3_All, CL263.Contig5_All, CL271.Contig2_All, CL2722.Contig1_All, CL2748.Contig2_All, CL2764.Contig2_All, CL2788.Contig2_All, CL2833.Contig1_All, CL2833.Contig3_All, CL2904.Contig1_All, CL2904.Contig2_All, CL2909.Contig1_All, CL2909.Contig3_All, CL2909.Contig4_All, CL296.Contig16_All, CL2975.Contig2_All, CL3011.Contig1_All, CL3027.Contig3_All, CL3046.Contig1_All, CL305.Contig2_All, CL309.Contig2_All, CL309.Contig3_All, CL309.Contig4_All, CL3092.Contig1_All, CL3092.Contig2_All, CL3114.Contig2_All, CL3116.Contig2_All, CL3260.Contig1_All, CL3260.Contig2_All, CL3260.Contig3_All, CL3260.Contig4_All, CL3260.Contig5_All, CL3260.Contig7_All, CL3294.Contig1_All, CL3295.Contig1_All, CL3297.Contig1_All, CL3297.Contig2_All, CL3315.Contig3_All, CL3315.Contig4_All, CL3347.Contig1_All, CL3347.Contig2_All, CL3500.Contig1_All, CL3510.Contig3_All, CL3525.Contig1_All, CL3525.Contig2_All, CL3543.Contig2_All, CL3543.Contig3_All, CL3543.Contig4_All, CL358.Contig1_All, CL367.Contig1_All, CL367.Contig2_All, CL367.Contig3_All, CL3672.Contig4_All, CL3689.Contig2_All, CL369.Contig2_All, CL3761.Contig1_All, CL3823.Contig1_All, CL3823.Contig2_All, CL3825.Contig1_All, CL3825.Contig2_All, CL392.Contig1_All, CL392.Contig2_All, CL392.Contig3_All, CL4002.Contig1_All, CL4062.Contig2_All, CL4069.Contig1_All, CL4088.Contig1_All, CL4088.Contig2_All, CL4130.Contig1_All, CL4187.Contig1_All, CL4203.Contig3_All, CL4216.Contig1_All, CL4216.Contig2_All, CL4250.Contig2_All, CL4402.Contig1_All, CL4439.Contig2_All, CL4454.Contig3_All, CL4596.Contig1_All, CL4637.Contig1_All, CL4699.Contig1_All, CL4787.Contig1_All, CL4862.Contig1_All, CL4896.Contig1_All, CL5017.Contig2_All, CL503.Contig2_All, CL503.Contig3_All, CL503.Contig4_All, CL503.Contig5_All, CL503.Contig6_All, CL503.Contig8_All, CL5061.Contig2_All, CL5162.Contig1_All, CL5162.Contig2_All, CL5246.Contig3_All, CL5330.Contig1_All, CL5330.Contig2_All, CL5361.Contig2_All, CL5361.Contig4_All, CL5468.Contig3_All, CL5469.Contig2_All, CL571.Contig2_All, CL573.Contig1_All, CL600.Contig1_All, CL600.Contig2_All, CL600.Contig3_All, CL600.Contig4_All, CL600.Contig6_All, CL644.Contig1_All, CL657.Contig1_All, CL657.Contig2_All, CL695.Contig16_All, CL741.Contig2_All, CL748.Contig1_All, CL748.Contig3_All, CL916.Contig2_All, CL92.Contig3_All, CL923.Contig1_All, CL923.Contig2_All, CL923.Contig3_All, CL970.Contig2_All, Unigene10097_All, Unigene10201_All, Unigene10202_All, Unigene10448_All, Unigene10682_All, Unigene10775_All, Unigene10998_All, Unigene11052_All, Unigene11082_All, Unigene11289_All, Unigene11298_All, Unigene11300_All, Unigene11307_All, Unigene1131_All, Unigene11372_All, Unigene1137_All, Unigene11459_All, Unigene11521_All, Unigene11597_All, Unigene11598_All, Unigene11599_All, Unigene11628_All, Unigene11677_All, Unigene1168_All, Unigene11935_All, Unigene12201_All, Unigene12255_All, Unigene12301_All, Unigene123_All, Unigene12418_All, Unigene1257_All, Unigene12603_All, Unigene12617_All, Unigene12658_All, Unigene12671_All, Unigene12684_All, Unigene12697_All, Unigene12711_All, Unigene12828_All, Unigene13036_All, Unigene13092_All, Unigene1447_All, Unigene1496_All, Unigene1512_All, Unigene15194_All, Unigene15195_All, Unigene15271_All, Unigene16098_All, Unigene1633_All, Unigene16435_All, Unigene16510_All, Unigene16716_All, Unigene16747_All, Unigene16840_All, Unigene16874_All, Unigene16963_All, Unigene16976_All, Unigene16994_All, Unigene1705_All, Unigene17117_All, Unigene17362_All, Unigene17551_All, Unigene1777_All, Unigene17815_All, Unigene17824_All, Unigene18076_All, Unigene18106_All, Unigene18178_All, Unigene18215_All, Unigene18276_All, Unigene18317_All, Unigene18378_All, Unigene1855_All, Unigene18598_All, Unigene18644_All, Unigene18645_All, Unigene18873_All, Unigene19289_All, Unigene19364_All, Unigene1941_All, Unigene19453_All, Unigene19601_All, Unigene19647_All, Unigene19745_All, Unigene1974_All, Unigene20065_All, Unigene20111_All, Unigene2015_All, Unigene20299_All, Unigene2052_All, Unigene20530_All, Unigene2053_All, Unigene20625_All, Unigene21008_All, Unigene210_All, Unigene2145_All, Unigene214_All, Unigene2233_All, Unigene237_All, Unigene24_All, Unigene2514_All, Unigene2741_All, Unigene278_All, Unigene3088_All, Unigene3153_All, Unigene3300_All, Unigene3328_All, Unigene3471_All, Unigene3482_All, Unigene3659_All, Unigene3696_All, Unigene3945_All, Unigene4022_All, Unigene4067_All, Unigene4161_All, Unigene4207_All, Unigene4269_All, Unigene4692_All, Unigene4920_All, Unigene4935_All, Unigene4970_All, Unigene4987_All, Unigene500_All, Unigene5088_All, Unigene5303_All, Unigene5305_All, Unigene5715_All, Unigene5753_All, Unigene5808_All, Unigene5813_All, Unigene5821_All, Unigene5873_All, Unigene5993_All, Unigene5994_All, Unigene6013_All, Unigene6014_All, Unigene6015_All, Unigene6029_All, Unigene6037_All, Unigene6059_All, Unigene6073_All, Unigene6136_All, Unigene6273_All, Unigene6283_All, Unigene6525_All, Unigene6655_All, Unigene6896_All, Unigene6907_All, Unigene6994_All, Unigene699_All, Unigene7063_All, Unigene7129_All, Unigene7250_All, Unigene7348_All, Unigene7547_All, Unigene7704_All, Unigene7759_All, Unigene7829_All, Unigene7854_All, Unigene785_All, Unigene7958_All, Unigene7993_All, Unigene8009_All, Unigene8017_All, Unigene8029_All, Unigene8184_All, Unigene8585_All, Unigene8615_All, Unigene8725_All, Unigene8747_All, Unigene8770_All, Unigene9014_All, Unigene9039_All, Unigene9042_All, Unigene9050_All, Unigene9059_All, Unigene9067_All, Unigene9076_All, Unigene90_All, Unigene9163_All, Unigene9379_All, Unigene9719_All, Unigene9722_All, Unigene9785_All, Unigene9787_All, Unigene9836_All, Unigene9877_All, Unigene9902_All, Unigene9904_All, Unigene9947_All

64	Phototransduction - fly	CL1165.Contig1_All, CL1165.Contig2_All, CL1165.Contig3_All, CL1165.Contig4_All, CL128.Contig10_All, CL1286.Contig2_All, CL1355.Contig1_All, CL156.Contig1_All, CL156.Contig3_All, CL156.Contig4_All, CL1803.Contig1_All, CL1823.Contig8_All, CL2131.Contig4_All, CL2131.Contig5_All, CL2131.Contig6_All, CL2131.Contig8_All, CL2182.Contig1_All, CL2182.Contig3_All, CL2182.Contig4_All, CL2468.Contig1_All, CL2468.Contig2_All, CL2930.Contig5_All, CL3224.Contig1_All, CL3323.Contig1_All, CL3323.Contig2_All, CL3667.Contig1_All, CL3667.Contig2_All, CL3667.Contig3_All, CL39.Contig1_All, CL39.Contig2_All, CL39.Contig3_All, CL39.Contig4_All, CL4395.Contig1_All, CL44.Contig10_All, CL44.Contig12_All, CL44.Contig13_All, CL44.Contig16_All, CL44.Contig17_All, CL44.Contig18_All, CL44.Contig19_All, CL44.Contig1_All, CL44.Contig22_All, CL44.Contig23_All, CL44.Contig2_All, CL44.Contig3_All, CL44.Contig4_All, CL44.Contig5_All, CL44.Contig6_All, CL44.Contig8_All, CL44.Contig9_All, CL4509.Contig1_All, CL4509.Contig2_All, CL5290.Contig1_All, CL5290.Contig2_All, CL5391.Contig1_All, CL5391.Contig2_All, CL5399.Contig1_All, CL5399.Contig2_All, CL5468.Contig3_All, CL691.Contig1_All, CL691.Contig5_All, CL772.Contig1_All, CL772.Contig2_All, CL90.Contig1_All, CL90.Contig2_All, CL940.Contig3_All, CL940.Contig5_All, Unigene10006_All, Unigene10574_All, Unigene11054_All, Unigene11597_All, Unigene11598_All, Unigene11599_All, Unigene12110_All, Unigene12315_All, Unigene12684_All, Unigene12719_All, Unigene13092_All, Unigene16498_All, Unigene16769_All, Unigene1726_All, Unigene18598_All, Unigene18898_All, Unigene18993_All, Unigene19947_All, Unigene2209_All, Unigene2741_All, Unigene278_All, Unigene2812_All, Unigene3996_All, Unigene4013_All, Unigene4053_All, Unigene4070_All, Unigene4780_All, Unigene555_All, Unigene5675_All, Unigene8422_All, Unigene9437_All
65	Inositol phosphate metabolism	CL2035.Contig1_All, CL2035.Contig4_All, CL2551.Contig1_All, CL2552.Contig3_All, CL2835.Contig1_All, CL2835.Contig2_All, CL2835.Contig3_All, CL2835.Contig4_All, CL2930.Contig5_All, CL2941.Contig1_All, CL2944.Contig1_All, CL2944.Contig2_All, CL309.Contig2_All, CL309.Contig3_All, CL309.Contig4_All, CL3142.Contig1_All, CL3142.Contig2_All, CL3163.Contig2_All, CL3410.Contig1_All, CL3410.Contig2_All, CL3505.Contig2_All, CL367.Contig1_All, CL367.Contig2_All, CL367.Contig3_All, CL3687.Contig1_All, CL3687.Contig2_All, CL3979.Contig1_All, CL3979.Contig2_All, CL4883.Contig1_All, CL4883.Contig2_All, CL5310.Contig2_All, CL542.Contig1_All, CL542.Contig2_All, CL688.Contig2_All, CL728.Contig1_All, CL737.Contig5_All, CL737.Contig6_All, CL959.Contig1_All, Unigene11003_All, Unigene11082_All, Unigene11319_All, Unigene11826_All, Unigene12619_All, Unigene12907_All, Unigene13034_All, Unigene17873_All, Unigene3149_All, Unigene4053_All, Unigene4070_All, Unigene4159_All, Unigene4332_All, Unigene5681_All, Unigene5811_All, Unigene6640_All, Unigene6996_All, Unigene6997_All, Unigene7000_All, Unigene8422_All, Unigene9067_All, Unigene9127_All, Unigene9805_All
66	Apoptosis	CL1066.Contig1_All, CL1066.Contig2_All, CL1066.Contig3_All, CL131.Contig1_All, CL131.Contig2_All, CL1452.Contig1_All, CL2882.Contig1_All, CL2882.Contig2_All, CL367.Contig1_All, CL367.Contig2_All, CL367.Contig3_All, CL4175.Contig1_All, CL4175.Contig2_All, CL4175.Contig3_All, CL4521.Contig1_All, CL4521.Contig2_All, CL4521.Contig3_All, CL4521.Contig5_All, CL5064.Contig2_All, CL5299.Contig1_All, CL695.Contig16_All, CL851.Contig3_All, CL940.Contig3_All, CL940.Contig5_All, Unigene11526_All, Unigene11724_All, Unigene11867_All, Unigene12188_All, Unigene12691_All, Unigene13853_All, Unigene14063_All, Unigene1757_All, Unigene17702_All, Unigene2749_All, Unigene3779_All, Unigene4112_All, Unigene4581_All, Unigene7049_All, Unigene7205_All, Unigene7220_All, Unigene7667_All, Unigene8246_All, Unigene951_All, Unigene9664_All, Unigene9733_All
67	Glioma	CL1066.Contig1_All, CL1066.Contig2_All, CL1066.Contig3_All, CL1286.Contig2_All, CL1548.Contig2_All, CL1548.Contig3_All, CL1807.Contig1_All, CL1807.Contig2_All, CL1823.Contig8_All, CL1874.Contig2_All, CL2131.Contig4_All, CL2131.Contig5_All, CL2131.Contig6_All, CL2131.Contig8_All, CL2182.Contig1_All, CL2182.Contig3_All, CL2182.Contig4_All, CL241.Contig1_All, CL241.Contig7_All, CL2468.Contig1_All, CL2468.Contig2_All, CL2552.Contig3_All, CL3373.Contig1_All, CL3373.Contig2_All, CL3667.Contig1_All, CL3667.Contig2_All, CL3667.Contig3_All, CL367.Contig1_All, CL367.Contig2_All, CL367.Contig3_All, CL3796.Contig2_All, CL3796.Contig3_All, CL39.Contig1_All, CL39.Contig2_All, CL39.Contig3_All, CL39.Contig4_All, CL3979.Contig1_All, CL3979.Contig2_All, CL4395.Contig1_All, CL44.Contig10_All, CL44.Contig12_All, CL44.Contig13_All, CL44.Contig16_All, CL44.Contig17_All, CL44.Contig18_All, CL44.Contig19_All, CL44.Contig1_All, CL44.Contig22_All, CL44.Contig23_All, CL44.Contig2_All, CL44.Contig3_All, CL44.Contig4_All, CL44.Contig5_All, CL44.Contig6_All, CL44.Contig8_All, CL44.Contig9_All, CL4439.Contig2_All, CL4509.Contig1_All, CL4509.Contig2_All, CL4559.Contig2_All, CL4559.Contig3_All, CL4787.Contig1_All, CL5391.Contig1_All, CL5391.Contig2_All, CL695.Contig16_All, CL772.Contig1_All, CL772.Contig2_All, CL940.Contig3_All, CL940.Contig5_All, CL95.Contig1_All, Unigene10006_All, Unigene10174_All, Unigene10574_All, Unigene11054_All, Unigene12099_All, Unigene12315_All, Unigene12671_All, Unigene12719_All, Unigene16769_All, Unigene18898_All, Unigene2374_All, Unigene2749_All, Unigene3996_All, Unigene4013_All, Unigene5675_All, Unigene699_All, Unigene7016_All, Unigene8046_All, Unigene8747_All
68	Basal transcription factors	CL1109.Contig1_All, CL1109.Contig2_All, CL1109.Contig3_All, CL1109.Contig4_All, CL1470.Contig1_All, CL1470.Contig2_All, CL1637.Contig1_All, CL1687.Contig4_All, CL1812.Contig1_All, CL1812.Contig2_All, CL185.Contig1_All, CL185.Contig2_All, CL185.Contig3_All, CL185.Contig4_All, CL2733.Contig2_All, CL2733.Contig3_All, CL2848.Contig4_All, CL3165.Contig1_All, CL3165.Contig2_All, CL3165.Contig3_All, CL3372.Contig1_All, CL3963.Contig1_All, CL4103.Contig1_All, CL4319.Contig2_All, CL4555.Contig1_All, CL5237.Contig1_All, CL5237.Contig2_All, CL856.Contig1_All, Unigene1137_All, Unigene12516_All, Unigene12806_All, Unigene12891_All, Unigene13197_All, Unigene1638_All, Unigene1696_All, Unigene17856_All, Unigene17875_All, Unigene17994_All, Unigene1857_All, Unigene236_All, Unigene2960_All, Unigene3871_All, Unigene4384_All, Unigene480_All, Unigene4817_All, Unigene4824_All, Unigene5009_All, Unigene5010_All, Unigene5011_All, Unigene5012_All, Unigene567_All, Unigene6046_All, Unigene7049_All, Unigene7699_All, Unigene7741_All, Unigene8025_All, Unigene8250_All, Unigene8784_All, Unigene8833_All, Unigene9061_All

69	Amyotrophic lateral sclerosis (ALS)	CL1042.Contig3_All, CL1048.Contig2_All, CL1535.Contig3_All, CL1549.Contig2_All, CL1850.Contig1_All, CL1850.Contig2_All, CL1884.Contig1_All, CL1884.Contig4_All, CL2129.Contig4_All, CL2405.Contig1_All, CL2426.Contig1_All, CL2426.Contig2_All, CL2730.Contig4_All, CL2733.Contig2_All, CL2882.Contig1_All, CL2882.Contig2_All, CL2986.Contig1_All, CL2986.Contig2_All, CL305.Contig2_All, CL3241.Contig1_All, CL3241.Contig2_All, CL3562.Contig6_All, CL36.Contig6_All, CL3635.Contig3_All, CL3651.Contig1_All, CL3651.Contig2_All, CL3657.Contig3_All, CL3789.Contig1_All, CL3789.Contig2_All, CL3803.Contig2_All, CL3886.Contig1_All, CL3886.Contig2_All, CL3886.Contig3_All, CL3886.Contig4_All, CL392.Contig1_All, CL392.Contig3_All, CL4094.Contig1_All, CL4094.Contig2_All, CL4216.Contig1_All, CL4216.Contig2_All, CL4340.Contig1_All, CL4340.Contig2_All, CL4521.Contig1_All, CL4521.Contig2_All, CL4521.Contig3_All, CL4521.Contig5_All, CL4686.Contig2_All, CL4803.Contig1_All, CL4966.Contig1_All, CL4983.Contig2_All, CL5064.Contig2_All, CL5136.Contig1_All, CL5136.Contig2_All, CL5299.Contig1_All, CL851.Contig3_All, CL870.Contig4_All, CL870.Contig8_All, CL870.Contig9_All, Unigene10058_All, Unigene10887_All, Unigene10948_All, Unigene1095_All, Unigene10981_All, Unigene1137_All, Unigene11623_All, Unigene1169_All, Unigene11724_All, Unigene12139_All, Unigene12175_All, Unigene12186_All, Unigene12188_All, Unigene12322_All, Unigene12468_All, Unigene12738_All, Unigene12888_All, Unigene1326_All, Unigene13853_All, Unigene13901_All, Unigene14063_All, Unigene1417_All, Unigene166_All, Unigene16_All, Unigene17577_All, Unigene17702_All, Unigene17856_All, Unigene18186_All, Unigene18260_All, Unigene18343_All, Unigene18761_All, Unigene190_All, Unigene19148_All, Unigene19880_All, Unigene2039_All, Unigene2552_All, Unigene2621_All, Unigene3457_All, Unigene3846_All, Unigene4085_All, Unigene4190_All, Unigene4406_All, Unigene4541_All, Unigene4581_All, Unigene5092_All, Unigene6069_All, Unigene6123_All, Unigene6169_All, Unigene661_All, Unigene6961_All, Unigene7049_All, Unigene7067_All, Unigene7074_All, Unigene715_All, Unigene7192_All, Unigene7205_All, Unigene7213_All, Unigene7508_All, Unigene8023_All, Unigene822_All, Unigene8251_All, Unigene9015_All, Unigene9132_All, Unigene9252_All, Unigene9664_All
70	Renin-angiotensin system	CL1175.Contig1_All, CL1175.Contig2_All, CL1537.Contig1_All, CL1537.Contig2_All, CL2028.Contig1_All, CL2028.Contig2_All, CL2267.Contig1_All, CL300.Contig7_All, CL3106.Contig1_All, CL3331.Contig1_All, CL4294.Contig1_All, CL4294.Contig2_All, CL4410.Contig1_All, CL4903.Contig1_All, Unigene10452_All, Unigene106_All, Unigene11378_All, Unigene11484_All, Unigene11660_All, Unigene12199_All, Unigene12922_All, Unigene15769_All, Unigene17741_All, Unigene2096_All, Unigene4326_All, Unigene4510_All, Unigene5034_All, Unigene5065_All, Unigene5225_All, Unigene5257_All, Unigene6036_All, Unigene6207_All, Unigene6692_All, Unigene7926_All, Unigene8011_All, Unigene8079_All, Unigene8166_All, Unigene8221_All, Unigene8504_All, Unigene8718_All, Unigene9038_All, Unigene9058_All
71	Small cell lung cancer	CL1055.Contig5_All, CL1066.Contig1_All, CL1066.Contig2_All, CL1066.Contig3_All, CL1091.Contig14_All, CL1202.Contig2_All, CL1328.Contig2_All, CL1436.Contig2_All, CL1495.Contig1_All, CL1495.Contig2_All, CL1741.Contig1_All, CL1960.Contig1_All, CL1960.Contig2_All, CL2052.Contig1_All, CL2052.Contig2_All, CL2066.Contig1_All, CL209.Contig1_All, CL209.Contig2_All, CL209.Contig3_All, CL209.Contig4_All, CL2099.Contig1_All, CL2331.Contig2_All, CL2331.Contig3_All, CL2552.Contig3_All, CL2882.Contig1_All, CL2882.Contig2_All, CL2927.Contig3_All, CL3327.Contig1_All, CL3327.Contig2_All, CL3371.Contig1_All, CL3373.Contig1_All, CL3373.Contig2_All, CL367.Contig1_All, CL367.Contig2_All, CL367.Contig3_All, CL470.Contig1_All, CL4992.Contig1_All, CL5048.Contig1_All, CL5204.Contig1_All, CL5204.Contig2_All, CL5268.Contig2_All, CL5299.Contig1_All, CL5373.Contig1_All, CL5373.Contig2_All, CL5464.Contig1_All, CL5464.Contig2_All, CL5464.Contig3_All, CL695.Contig16_All, CL748.Contig1_All, CL748.Contig3_All, CL851.Contig3_All, CL880.Contig3_All, Unigene10095_All, Unigene10096_All, Unigene10316_All, Unigene10724_All, Unigene11160_All, Unigene11358_All, Unigene11511_All, Unigene11538_All, Unigene11543_All, Unigene11544_All, Unigene11588_All, Unigene11724_All, Unigene12099_All, Unigene12155_All, Unigene12188_All, Unigene12533_All, Unigene12623_All, Unigene12692_All, Unigene12710_All, Unigene14177_All, Unigene15658_All, Unigene15840_All, Unigene1614_All, Unigene16913_All, Unigene16965_All, Unigene16966_All, Unigene1757_All, Unigene17702_All, Unigene17728_All, Unigene18488_All, Unigene18496_All, Unigene19237_All, Unigene19426_All, Unigene19840_All, Unigene2190_All, Unigene2374_All, Unigene2503_All, Unigene2560_All, Unigene2749_All, Unigene28_All, Unigene3123_All, Unigene3184_All, Unigene3193_All, Unigene3403_All, Unigene3889_All, Unigene3945_All, Unigene414_All, Unigene4207_All, Unigene4218_All, Unigene4418_All, Unigene4581_All, Unigene4922_All, Unigene5169_All, Unigene5799_All, Unigene5896_All, Unigene6041_All, Unigene6614_All, Unigene6780_All, Unigene7049_All, Unigene7054_All, Unigene7106_All, Unigene7205_All, Unigene7220_All, Unigene7299_All, Unigene740_All, Unigene7560_All, Unigene8102_All, Unigene8399_All, Unigene8514_All, Unigene8562_All, Unigene9284_All, Unigene9611_All, Unigene9664_All, Unigene9988_All
72	Taste transduction	CL1452.Contig1_All, CL1730.Contig2_All, CL260.Contig15_All, CL2930.Contig5_All, CL3234.Contig2_All, CL4085.Contig1_All, CL4085.Contig2_All, CL4119.Contig1_All, CL4262.Contig2_All, CL4316.Contig1_All, CL5148.Contig2_All, CL5280.Contig1_All, CL905.Contig1_All, CL905.Contig2_All, CL940.Contig3_All, CL940.Contig5_All, Unigene10058_All, Unigene12110_All, Unigene124_All, Unigene16125_All, Unigene18035_All, Unigene19476_All, Unigene19524_All, Unigene2029_All, Unigene2032_All, Unigene2209_All, Unigene3204_All, Unigene3779_All, Unigene4053_All, Unigene4070_All, Unigene4112_All, Unigene4532_All, Unigene4780_All, Unigene4950_All, Unigene6151_All, Unigene7322_All, Unigene7661_All, Unigene8081_All, Unigene8114_All, Unigene8422_All, Unigene93_All, Unigene957_All, Unigene9629_All, Unigene9733_All

73	Leukocyte transendothelial migration	CL1066.Contig1_All, CL1066.Contig2_All, CL1066.Contig3_All, CL119.Contig10_All, CL119.Contig11_All, CL119.Contig3_All, CL119.Contig4_All, CL119.Contig8_All, CL119.Contig9_All, CL1355.Contig1_All, CL1503.Contig3_All, CL1503.Contig4_All, CL1503.Contig5_All, CL1535.Contig3_All, CL156.Contig1_All, CL156.Contig3_All, CL156.Contig4_All, CL1803.Contig1_All, CL1954.Contig1_All, CL1954.Contig2_All, CL2034.Contig1_All, CL2034.Contig2_All, CL2066.Contig1_All, CL2182.Contig1_All, CL2182.Contig3_All, CL2182.Contig4_All, CL2222.Contig9_All, CL2224.Contig2_All, CL2224.Contig3_All, CL2224.Contig4_All, CL263.Contig3_All, CL263.Contig5_All, CL271.Contig2_All, CL3042.Contig1_All, CL3042.Contig3_All, CL3116.Contig2_All, CL3130.Contig1_All, CL3130.Contig2_All, CL3348.Contig1_All, CL3397.Contig1_All, CL3397.Contig3_All, CL3397.Contig4_All, CL3436.Contig1_All, CL3436.Contig4_All, CL3436.Contig9_All, CL3460.Contig2_All, CL3460.Contig3_All, CL361.Contig1_All, CL367.Contig1_All, CL367.Contig2_All, CL367.Contig3_All, CL3823.Contig1_All, CL3823.Contig2_All, CL39.Contig1_All, CL39.Contig2_All, CL39.Contig3_All, CL39.Contig4_All, CL3979.Contig1_All, CL3979.Contig2_All, CL4966.Contig1_All, CL523.Contig1_All, CL523.Contig2_All, CL5330.Contig1_All, CL5330.Contig2_All, CL5361.Contig2_All, CL5361.Contig4_All, CL5391.Contig1_All, CL5391.Contig2_All, CL5468.Contig3_All, CL695.Contig16_All, CL724.Contig1_All, CL748.Contig1_All, CL748.Contig3_All, CL940.Contig3_All, CL940.Contig5_All, Unigene11307_All, Unigene11597_All, Unigene11598_All, Unigene11599_All, Unigene11628_All, Unigene11874_All, Unigene12314_All, Unigene12684_All, Unigene13092_All, Unigene18317_All, Unigene18423_All, Unigene1855_All, Unigene18598_All, Unigene18644_All, Unigene18873_All, Unigene18898_All, Unigene19601_All, Unigene2741_All, Unigene278_All, Unigene3088_All, Unigene3135_All, Unigene3153_All, Unigene3300_All, Unigene3945_All, Unigene4207_All, Unigene5521_All, Unigene5727_All, Unigene5808_All, Unigene5813_All, Unigene6146_All, Unigene6217_All, Unigene6283_All, Unigene7063_All, Unigene7158_All, Unigene7547_All, Unigene783_All, Unigene8247_All, Unigene8585_All, Unigene9042_All, Unigene9050_All, Unigene9379_All, Unigene9877_All
74	Tuberculosis	CL1085.Contig3_All, CL1280.Contig1_All, CL1280.Contig2_All, CL1286.Contig2_All, CL1341.Contig2_All, CL1823.Contig8_All, CL188.Contig16_All, CL2131.Contig4_All, CL2131.Contig5_All, CL2131.Contig6_All, CL2131.Contig8_All, CL2150.Contig3_All, CL2170.Contig2_All, CL2170.Contig5_All, CL2224.Contig2_All, CL2224.Contig3_All, CL2224.Contig4_All, CL2468.Contig1_All, CL2468.Contig2_All, CL2634.Contig1_All, CL2634.Contig2_All, CL2711.Contig1_All, CL2711.Contig2_All, CL2750.Contig3_All, CL2882.Contig1_All, CL2882.Contig2_All, CL3091.Contig1_All, CL3091.Contig2_All, CL3091.Contig3_All, CL3091.Contig4_All, CL3221.Contig1_All, CL3274.Contig1_All, CL3667.Contig1_All, CL3667.Contig2_All, CL3667.Contig3_All, CL3865.Contig2_All, CL3884.Contig2_All, CL3920.Contig2_All, CL4090.Contig2_All, CL4187.Contig1_All, CL4250.Contig2_All, CL4317.Contig1_All, CL4395.Contig1_All, CL44.Contig10_All, CL44.Contig12_All, CL44.Contig13_All, CL44.Contig16_All, CL44.Contig17_All, CL44.Contig18_All, CL44.Contig19_All, CL44.Contig1_All, CL44.Contig22_All, CL44.Contig23_All, CL44.Contig2_All, CL44.Contig3_All, CL44.Contig4_All, CL44.Contig5_All, CL44.Contig6_All, CL44.Contig8_All, CL44.Contig9_All, CL4459.Contig1_All, CL4509.Contig1_All, CL4509.Contig2_All, CL4521.Contig1_All, CL4521.Contig2_All, CL4521.Contig3_All, CL4521.Contig5_All, CL4596.Contig2_All, CL4857.Contig1_All, CL4966.Contig1_All, CL5064.Contig2_All, CL5115.Contig1_All, CL5299.Contig1_All, CL5466.Contig1_All, CL724.Contig1_All, CL772.Contig1_All, CL772.Contig2_All, CL851.Contig3_All, Unigene10006_All, Unigene10007_All, Unigene10026_All, Unigene10041_All, Unigene10170_All, Unigene10296_All, Unigene10423_All, Unigene10424_All, Unigene10483_All, Unigene10503_All, Unigene1051_All, Unigene10574_All, Unigene10682_All, Unigene11054_All, Unigene1109_All, Unigene11270_All, Unigene11307_All, Unigene1131_All, Unigene11553_All, Unigene11554_All, Unigene11724_All, Unigene11865_All, Unigene11901_All, Unigene11919_All, Unigene12028_All, Unigene12172_All, Unigene12188_All, Unigene12209_All, Unigene12302_All, Unigene12315_All, Unigene12455_All, Unigene12634_All, Unigene12719_All, Unigene12840_All, Unigene12853_All, Unigene12923_All, Unigene13324_All, Unigene13732_All, Unigene13853_All, Unigene15243_All, Unigene15257_All, Unigene16747_All, Unigene16769_All, Unigene1741_All, Unigene1757_All, Unigene17702_All, Unigene18106_All, Unigene18675_All, Unigene18707_All, Unigene18950_All, Unigene1895_All, Unigene1913_All, Unigene20095_All, Unigene2015_All, Unigene2053_All, Unigene2096_All, Unigene2111_All, Unigene220_All, Unigene2275_All, Unigene2749_All, Unigene2982_All, Unigene313_All, Unigene3157_All, Unigene3730_All, Unigene3830_All, Unigene3996_All, Unigene4013_All, Unigene4159_All, Unigene44_All, Unigene4581_All, Unigene462_All, Unigene4677_All, Unigene54_All, Unigene5675_All, Unigene5781_All, Unigene5808_All, Unigene5890_All, Unigene6013_All, Unigene6015_All, Unigene6173_All, Unigene624_All, Unigene6907_All, Unigene7049_All, Unigene7056_All, Unigene7129_All, Unigene712_All, Unigene7205_All, Unigene7873_All, Unigene8151_All, Unigene8246_All, Unigene8504_All, Unigene8737_All, Unigene9050_All, Unigene9229_All, Unigene925_All, Unigene9664_All, Unigene9877_All
75	Chagas disease (American trypanosomiasis)	CL1066.Contig1_All, CL1066.Contig2_All, CL1066.Contig3_All, CL1085.Contig3_All, CL1776.Contig1_All, CL1966.Contig2_All, CL2873.Contig1_All, CL2873.Contig2_All, CL2930.Contig5_All, CL367.Contig1_All, CL367.Contig2_All, CL367.Contig3_All, CL4005.Contig2_All, CL4005.Contig3_All, CL4271.Contig1_All, CL4736.Contig1_All, CL4736.Contig2_All, CL4923.Contig2_All, CL4966.Contig1_All, CL5115.Contig1_All, CL5399.Contig1_All, CL5399.Contig2_All, CL695.Contig16_All, CL761.Contig1_All, CL761.Contig2_All, Unigene10058_All, Unigene10242_All, Unigene11845_All, Unigene13432_All, Unigene1479_All, Unigene1743_All, Unigene1757_All, Unigene176_All, Unigene20095_All, Unigene2196_All, Unigene2209_All, Unigene220_All, Unigene2749_All, Unigene4053_All, Unigene4070_All, Unigene4126_All, Unigene4532_All, Unigene4801_All, Unigene5521_All, Unigene6217_All, Unigene6977_All, Unigene69_All, Unigene8246_All, Unigene8422_All, Unigene9437_All

76	Wnt signaling pathway	CL1276.Contig2_All, CL1389.Contig2_All, CL1439.Contig1_All, CL1439.Contig2_All, CL1452.Contig1_All, CL1535.Contig3_All, CL1831.Contig1_All, CL1831.Contig2_All, CL1843.Contig3_All, CL1912.Contig1_All, CL1912.Contig2_All, CL2170.Contig2_All, CL2170.Contig5_All, CL2182.Contig1_All, CL2182.Contig3_All, CL2182.Contig4_All, CL2208.Contig1_All, CL2224.Contig2_All, CL2224.Contig3_All, CL2224.Contig4_All, CL2400.Contig5_All, CL2748.Contig1_All, CL2748.Contig2_All, CL2788.Contig2_All, CL2838.Contig1_All, CL2838.Contig2_All, CL2838.Contig4_All, CL2847.Contig2_All, CL2930.Contig5_All, CL3823.Contig1_All, CL3823.Contig2_All, CL39.Contig1_All, CL39.Contig2_All, CL39.Contig3_All, CL39.Contig4_All, CL4190.Contig1_All, CL44.Contig10_All, CL44.Contig12_All, CL44.Contig13_All, CL44.Contig16_All, CL44.Contig17_All, CL44.Contig18_All, CL44.Contig19_All, CL44.Contig1_All, CL44.Contig22_All, CL44.Contig23_All, CL44.Contig2_All, CL44.Contig3_All, CL44.Contig4_All, CL44.Contig5_All, CL44.Contig6_All, CL44.Contig8_All, CL44.Contig9_All, CL4514.Contig2_All, CL4521.Contig1_All, CL4521.Contig2_All, CL4521.Contig3_All, CL4521.Contig5_All, CL4657.Contig2_All, CL4736.Contig1_All, CL4736.Contig2_All, CL4795.Contig1_All, CL5064.Contig2_All, CL5111.Contig1_All, CL5115.Contig1_All, CL5391.Contig1_All, CL5391.Contig2_All, CL896.Contig1_All, CL896.Contig3_All, CL940.Contig3_All, CL940.Contig5_All, Unigene10242_All, Unigene1040_All, Unigene10619_All, Unigene10761_All, Unigene10876_All, Unigene11040_All, Unigene11263_All, Unigene11307_All, Unigene11411_All, Unigene11922_All, Unigene11952_All, Unigene12591_All, Unigene12740_All, Unigene12874_All, Unigene12903_All, Unigene13134_All, Unigene13362_All, Unigene13432_All, Unigene13853_All, Unigene1479_All, Unigene150_All, Unigene1680_All, Unigene18644_All, Unigene18898_All, Unigene2050_All, Unigene220_All, Unigene236_All, Unigene247_All, Unigene2554_All, Unigene296_All, Unigene3054_All, Unigene3100_All, Unigene3131_All, Unigene3212_All, Unigene3715_All, Unigene3742_All, Unigene3779_All, Unigene3975_All, Unigene4053_All, Unigene4070_All, Unigene4112_All, Unigene442_All, Unigene4731_All, Unigene4765_All, Unigene4795_All, Unigene4801_All, Unigene4893_All, Unigene4940_All, Unigene4975_All, Unigene54_All, Unigene5727_All, Unigene5781_All, Unigene5808_All, Unigene6029_All, Unigene6746_All, Unigene7063_All, Unigene7163_All, Unigene7297_All, Unigene766_All, Unigene7671_All, Unigene777_All, Unigene783_All, Unigene798_All, Unigene8014_All, Unigene8422_All, Unigene8784_All, Unigene8861_All, Unigene8972_All, Unigene9050_All, Unigene9379_All, Unigene9641_All, Unigene9733_All, Unigene9746_All, Unigene9785_All, Unigene9877_All
77	Systemic lupus erythematosus	CL1085.Contig3_All, CL1503.Contig3_All, CL1503.Contig4_All, CL1503.Contig5_All, CL2197.Contig3_All, CL2901.Contig2_All, CL2901.Contig3_All, CL2901.Contig4_All, CL2901.Contig5_All, CL2901.Contig8_All, CL3032.Contig2_All, CL326.Contig1_All, CL4418.Contig2_All, CL4890.Contig1_All, CL838.Contig2_All, CL838.Contig3_All, CL870.Contig4_All, CL870.Contig8_All, CL870.Contig9_All, CL988.Contig1_All, CL988.Contig3_All, Unigene10020_All, Unigene10045_All, Unigene1056_All, Unigene10831_All, Unigene10869_All, Unigene10905_All, Unigene11396_All, Unigene11660_All, Unigene11913_All, Unigene12078_All, Unigene12228_All, Unigene12335_All, Unigene12663_All, Unigene13039_All, Unigene13095_All, Unigene17185_All, Unigene18873_All, Unigene19148_All, Unigene19400_All, Unigene20090_All, Unigene3217_All, Unigene6223_All, Unigene8848_All, Unigene9042_All, Unigene9633_All
78	Maturity onset diabetes of the young	CL1246.Contig1_All, CL1246.Contig3_All, CL1246.Contig4_All, CL1357.Contig2_All, CL1357.Contig3_All, CL1502.Contig2_All, CL1648.Contig1_All, CL1702.Contig1_All, CL1702.Contig2_All, CL1872.Contig1_All, CL2574.Contig2_All, CL2574.Contig3_All, CL302.Contig1_All, CL302.Contig2_All, CL3527.Contig1_All, CL4829.Contig2_All, CL493.Contig2_All, CL5236.Contig4_All, CL5236.Contig5_All, Unigene12112_All, Unigene1238_All, Unigene12660_All, Unigene2012_All, Unigene2345_All, Unigene2670_All, Unigene3158_All, Unigene4217_All, Unigene695_All, Unigene705_All, Unigene7287_All, Unigene7647_All, Unigene7739_All, Unigene9001_All, Unigene9707_All
79	Rheumatoid arthritis	CL1358.Contig1_All, CL1358.Contig2_All, CL1358.Contig3_All, CL2150.Contig3_All, CL2634.Contig1_All, CL2634.Contig2_All, CL3091.Contig1_All, CL3091.Contig2_All, CL3091.Contig3_All, CL3091.Contig4_All, CL3706.Contig1_All, CL3884.Contig2_All, CL4057.Contig1_All, CL4090.Contig2_All, CL4459.Contig1_All, CL4576.Contig1_All, CL4736.Contig1_All, CL4736.Contig2_All, CL504.Contig1_All, CL504.Contig2_All, Unigene10026_All, Unigene10296_All, Unigene10483_All, Unigene11240_All, Unigene11282_All, Unigene11879_All, Unigene11899_All, Unigene1189_All, Unigene12028_All, Unigene12168_All, Unigene12209_All, Unigene12302_All, Unigene12423_All, Unigene12810_All, Unigene12923_All, Unigene13508_All, Unigene13589_All, Unigene1479_All, Unigene15969_All, Unigene1741_All, Unigene17602_All, Unigene18095_All, Unigene18114_All, Unigene18707_All, Unigene20095_All, Unigene2961_All, Unigene2982_All, Unigene3351_All, Unigene3730_All, Unigene3982_All, Unigene44_All, Unigene4921_All, Unigene6915_All, Unigene9028_All, Unigene925_All, Unigene9928_All

80	MAPK signaling pathway	CL1054.Contig1_All, CL1054.Contig2_All, CL1066.Contig1_All, CL1066.Contig2_All, CL1120.Contig2_All, CL1390.Contig4_All, CL1390.Contig5_All, CL1452.Contig1_All, CL1535.Contig3_All, CL1548.Contig2_All, CL1548.Contig3_All, CL1618.Contig2_All, CL1626.Contig4_All, CL1626.Contig8_All, CL1626.Contig9_All, CL1695.Contig8_All, CL173.Contig1_All, CL173.Contig2_All, CL1758.Contig2_All, CL1758.Contig3_All, CL1758.Contig4_All, CL1807.Contig1_All, CL1807.Contig2_All, CL1831.Contig1_All, CL1831.Contig2_All, CL1925.Contig1_All, CL1960.Contig1_All, CL1960.Contig2_All, CL1966.Contig2_All, CL199.Contig1_All, CL2052.Contig1_All, CL2052.Contig2_All, CL2129.Contig4_All, CL2165.Contig2_All, CL2182.Contig1_All, CL2182.Contig3_All, CL2182.Contig4_All, CL2380.Contig2_All, CL2426.Contig1_All, CL2426.Contig2_All, CL2432.Contig2_All, CL2505.Contig3_All, CL2505.Contig4_All, CL2522.Contig1_All, CL2522.Contig2_All, CL2522.Contig3_All, CL2642.Contig1_All, CL2642.Contig2_All, CL2686.Contig1_All, CL2686.Contig2_All, CL271.Contig2_All, CL3043.Contig2_All, CL3137.Contig2_All, CL3209.Contig1_All, CL3217.Contig2_All, CL3263.Contig1_All, CL3290.Contig2_All, CL3290.Contig3_All, CL3311.Contig1_All, CL3311.Contig2_All, CL358.Contig1_All, CL3622.Contig2_All, CL3673.Contig2_All, CL3678.Contig2_All, CL3868.Contig1_All, CL3868.Contig2_All, CL3868.Contig3_All, CL39.Contig1_All, CL39.Contig2_All, CL39.Contig3_All, CL39.Contig4_All, CL400.Contig1_All, CL400.Contig2_All, CL4177.Contig1_All, CL4190.Contig1_All, CL4204.Contig1_All, CL4204.Contig2_All, CL4224.Contig1_All, CL4224.Contig2_All, CL4224.Contig3_All, CL4271.Contig1_All, CL4439.Contig2_All, CL4521.Contig1_All, CL4521.Contig2_All, CL4521.Contig3_All, CL4521.Contig5_All, CL4736.Contig1_All, CL4736.Contig2_All, CL4787.Contig1_All, CL4815.Contig1_All, CL4815.Contig2_All, CL4896.Contig1_All, CL4918.Contig1_All, CL4966.Contig1_All, CL5064.Contig2_All, CL5115.Contig1_All, CL5232.Contig1_All, CL5232.Contig2_All, CL5256.Contig1_All, CL5256.Contig2_All, CL5256.Contig4_All, CL5310.Contig2_All, CL5337.Contig2_All, CL5382.Contig1_All, CL5391.Contig1_All, CL5391.Contig2_All, CL5469.Contig2_All, CL558.Contig1_All, CL558.Contig2_All, CL558.Contig4_All, CL642.Contig1_All, CL642.Contig2_All, CL821.Contig1_All, CL821.Contig2_All, CL860.Contig10_All, CL860.Contig8_All, CL860.Contig9_All, CL89.Contig1_All, CL89.Contig2_All, CL940.Contig3_All, CL940.Contig5_All, Unigene10174_All, Unigene10687_All, Unigene10766_All, Unigene10837_All, Unigene11222_All, Unigene1156_All, Unigene1169_All, Unigene11810_All, Unigene12139_All, Unigene12484_All, Unigene12671_All, Unigene12928_All, Unigene13036_All, Unigene13209_All, Unigene1326_All, Unigene13733_All, Unigene13853_All, Unigene1479_All, Unigene15587_All, Unigene15863_All, Unigene16093_All, Unigene17465_All, Unigene1757_All, Unigene17751_All, Unigene18535_All, Unigene18898_All, Unigene2032_All, Unigene20578_All, Unigene2067_All, Unigene220_All, Unigene2749_All, Unigene3029_All, Unigene3033_All, Unigene3034_All, Unigene3036_All, Unigene3037_All, Unigene3042_All, Unigene3070_All, Unigene3131_All, Unigene327_All, Unigene3724_All, Unigene3779_All, Unigene4112_All, Unigene4142_All, Unigene4359_All, Unigene4798_All, Unigene5246_All, Unigene5280_All, Unigene6017_All, Unigene6101_All, Unigene6265_All, Unigene6977_All, Unigene699_All, Unigene7136_All, Unigene7702_All, Unigene78_All, Unigene8046_All, Unigene8205_All, Unigene8621_All, Unigene8701_All, Unigene8747_All, Unigene90_All, Unigene9719_All, Unigene9733_All, Unigene9867_All, Unigene9971_All
81	Oocyte meiosis	CL1286.Contig2_All, CL1452.Contig1_All, CL1563.Contig2_All, CL1766.Contig1_All, CL1823.Contig8_All, CL2131.Contig4_All, CL2131.Contig5_All, CL2131.Contig6_All, CL2131.Contig8_All, CL2400.Contig5_All, CL241.Contig1_All, CL241.Contig7_All, CL2416.Contig1_All, CL244.Contig2_All, CL2468.Contig1_All, CL2468.Contig2_All, CL249.Contig1_All, CL249.Contig3_All, CL249.Contig5_All, CL249.Contig6_All, CL2546.Contig1_All, CL2711.Contig1_All, CL2711.Contig2_All, CL2749.Contig2_All, CL2809.Contig2_All, CL2809.Contig3_All, CL2809.Contig8_All, CL2838.Contig1_All, CL2838.Contig2_All, CL2838.Contig4_All, CL2893.Contig1_All, CL2893.Contig2_All, CL2978.Contig2_All, CL2978.Contig3_All, CL3263.Contig1_All, CL3667.Contig1_All, CL3667.Contig2_All, CL3667.Contig3_All, CL3689.Contig2_All, CL4002.Contig1_All, CL4005.Contig2_All, CL4005.Contig3_All, CL4262.Contig2_All, CL4395.Contig1_All, CL44.Contig10_All, CL44.Contig12_All, CL44.Contig13_All, CL44.Contig16_All, CL44.Contig17_All, CL44.Contig18_All, CL44.Contig19_All, CL44.Contig1_All, CL44.Contig22_All, CL44.Contig23_All, CL44.Contig2_All, CL44.Contig3_All, CL44.Contig4_All, CL44.Contig5_All, CL44.Contig6_All, CL44.Contig8_All, CL44.Contig9_All, CL440.Contig3_All, CL440.Contig5_All, CL440.Contig6_All, CL4402.Contig1_All, CL4509.Contig1_All, CL4509.Contig2_All, CL4514.Contig2_All, CL4521.Contig1_All, CL4521.Contig2_All, CL4521.Contig3_All, CL4521.Contig5_All, CL4787.Contig1_All, CL4966.Contig1_All, CL5064.Contig2_All, CL5206.Contig1_All, CL5218.Contig1_All, CL5218.Contig2_All, CL691.Contig1_All, CL710.Contig2_All, CL741.Contig2_All, CL772.Contig1_All, CL772.Contig2_All, CL940.Contig3_All, CL940.Contig5_All, CL95.Contig1_All, Unigene10006_All, Unigene10242_All, Unigene10252_All, Unigene10253_All, Unigene10574_All, Unigene10876_All, Unigene11040_All, Unigene11054_All, Unigene1109_All, Unigene11321_All, Unigene11411_All, Unigene1168_All, Unigene1181_All, Unigene12161_All, Unigene1229_All, Unigene12315_All, Unigene12469_All, Unigene12522_All, Unigene12719_All, Unigene12740_All, Unigene12903_All, Unigene13421_All, Unigene13432_All, Unigene13853_All, Unigene1512_All, Unigene1538_All, Unigene16769_All, Unigene18319_All, Unigene18584_All, Unigene18993_All, Unigene1941_All, Unigene2029_All, Unigene2233_All, Unigene2434_All, Unigene2700_All, Unigene2863_All, Unigene3103_All, Unigene3779_All, Unigene3996_All, Unigene4013_All, Unigene4022_All, Unigene4112_All, Unigene47_All, Unigene4808_All, Unigene4935_All, Unigene4970_All, Unigene4975_All, Unigene5196_All, Unigene5675_All, Unigene5896_All, Unigene5897_All, Unigene6041_All, Unigene6253_All, Unigene6526_All, Unigene6655_All, Unigene6746_All, Unigene700_All, Unigene7299_All, Unigene7671_All, Unigene7704_All, Unigene7747_All, Unigene7759_All, Unigene7869_All, Unigene798_All, Unigene8075_All, Unigene9076_All, Unigene9091_All, Unigene93_All, Unigene9579_All, Unigene9733_All, Unigene9904_All
82	Circadian rhythm - fly	CL1843.Contig3_All, CL3787.Contig1_All, CL3787.Contig2_All, Unigene11922_All, Unigene236_All, Unigene247_All, Unigene3715_All, Unigene7297_All, Unigene8784_All, Unigene9006_All
83	Leishmaniasis	CL1085.Contig3_All, CL2182.Contig1_All, CL2182.Contig3_All, CL2182.Contig4_All, CL39.Contig1_All, CL39.Contig2_All, CL39.Contig3_All, CL39.Contig4_All, CL400.Contig1_All, CL400.Contig2_All, CL4736.Contig1_All, CL4736.Contig2_All, CL4966.Contig1_All, CL5391.Contig1_All, CL5391.Contig2_All, CL940.Contig3_All, CL940.Contig5_All, Unigene1479_All, Unigene1757_All, Unigene18898_All, Unigene20095_All, Unigene3131_All, Unigene3945_All, Unigene4207_All, Unigene6101_All, Unigene8246_All

84	p53 signaling pathway	CL131.Contig1_All, CL131.Contig2_All, CL1766.Contig1_All, CL2022.Contig1_All, CL2176.Contig3_All, CL2207.Contig2_All, CL2552.Contig3_All, CL268.Contig2_All, CL2882.Contig1_All, CL2882.Contig2_All, CL372.Contig1_All, CL4366.Contig1_All, CL5299.Contig1_All, CL851.Contig3_All, Unigene10252_All, Unigene10253_All, Unigene10735_All, Unigene11384_All, Unigene11724_All, Unigene11895_All, Unigene12099_All, Unigene12161_All, Unigene12188_All, Unigene12469_All, Unigene13421_All, Unigene1396_All, Unigene17102_All, Unigene17428_All, Unigene17702_All, Unigene19772_All, Unigene2374_All, Unigene3171_All, Unigene3833_All, Unigene4581_All, Unigene5554_All, Unigene5896_All, Unigene5995_All, Unigene6041_All, Unigene700_All, Unigene7049_All, Unigene7205_All, Unigene7299_All, Unigene9664_All
85	Neurotrophin signaling pathway	CL1009.Contig1_All, CL1066.Contig1_All, CL1066.Contig2_All, CL1066.Contig3_All, CL1104.Contig1_All, CL1104.Contig2_All, CL1104.Contig3_All, CL1104.Contig4_All, CL1104.Contig5_All, CL1104.Contig6_All, CL1104.Contig7_All, CL1104.Contig8_All, CL1104.Contig9_All, CL1286.Contig2_All, CL1535.Contig3_All, CL1690.Contig1_All, CL1695.Contig8_All, CL1807.Contig1_All, CL1807.Contig2_All, CL1823.Contig8_All, CL1843.Contig3_All, CL1874.Contig2_All, CL2131.Contig4_All, CL2131.Contig5_All, CL2131.Contig6_All, CL2131.Contig8_All, CL2222.Contig9_All, CL2426.Contig1_All, CL2426.Contig2_All, CL2468.Contig1_All, CL2468.Contig2_All, CL249.Contig1_All, CL249.Contig3_All, CL249.Contig5_All, CL249.Contig6_All, CL2541.Contig1_All, CL2541.Contig2_All, CL271.Contig2_All, CL2820.Contig3_All, CL3047.Contig1_All, CL3047.Contig2_All, CL3263.Contig1_All, CL3667.Contig1_All, CL3667.Contig2_All, CL3667.Contig3_All, CL367.Contig1_All, CL367.Contig2_All, CL367.Contig3_All, CL3673.Contig2_All, CL3979.Contig1_All, CL3979.Contig2_All, CL4158.Contig4_All, CL4158.Contig5_All, CL4158.Contig7_All, CL4395.Contig1_All, CL44.Contig10_All, CL44.Contig12_All, CL44.Contig13_All, CL44.Contig16_All, CL44.Contig17_All, CL44.Contig18_All, CL44.Contig19_All, CL44.Contig1_All, CL44.Contig22_All, CL44.Contig23_All, CL44.Contig2_All, CL44.Contig3_All, CL44.Contig4_All, CL44.Contig5_All, CL44.Contig6_All, CL44.Contig8_All, CL44.Contig9_All, CL4439.Contig2_All, CL4509.Contig1_All, CL4509.Contig2_All, CL4559.Contig2_All, CL4559.Contig3_All, CL4736.Contig1_All, CL4736.Contig2_All, CL4787.Contig1_All, CL4815.Contig1_All, CL4815.Contig2_All, CL4840.Contig2_All, CL4855.Contig1_All, CL4855.Contig2_All, CL4966.Contig1_All, CL5115.Contig1_All, CL5117.Contig1_All, CL5117.Contig2_All, CL695.Contig16_All, CL772.Contig1_All, CL772.Contig2_All, CL821.Contig1_All, CL821.Contig2_All, CL903.Contig1_All, Unigene10006_All, Unigene10174_All, Unigene10574_All, Unigene11054_All, Unigene11307_All, Unigene11922_All, Unigene12315_All, Unigene1236_All, Unigene12484_All, Unigene12522_All, Unigene12671_All, Unigene12719_All, Unigene13036_All, Unigene1479_All, Unigene1538_All, Unigene16769_All, Unigene1757_All, Unigene198_All, Unigene220_All, Unigene236_All, Unigene247_All, Unigene2749_All, Unigene3836_All, Unigene3996_All, Unigene4013_All, Unigene4940_All, Unigene5675_All, Unigene5808_All, Unigene6526_All, Unigene7016_All, Unigene7266_All, Unigene7297_All, Unigene7983_All, Unigene8046_All, Unigene8056_All, Unigene8205_All, Unigene8246_All, Unigene8747_All, Unigene8784_All, Unigene9050_All, Unigene9877_All
86	MAPK signaling pathway - fly	CL1480.Contig1_All, CL1480.Contig2_All, CL1548.Contig2_All, CL1548.Contig3_All, CL1807.Contig1_All, CL1807.Contig2_All, CL2222.Contig9_All, CL3830.Contig2_All, CL4439.Contig2_All, CL4787.Contig1_All, Unigene10174_All, Unigene10424_All, Unigene12671_All, Unigene13266_All, Unigene16846_All, Unigene16847_All, Unigene17796_All, Unigene3700_All, Unigene4079_All, Unigene4137_All, Unigene5695_All, Unigene699_All, Unigene7248_All, Unigene8045_All, Unigene8046_All, Unigene8087_All, Unigene8747_All
87	Glycosaminoglycan degradation	CL151.Contig1_All, CL151.Contig2_All, CL151.Contig4_All, CL3965.Contig1_All, CL3965.Contig2_All, CL3965.Contig4_All, CL5403.Contig2_All, Unigene17953_All, Unigene2281_All, Unigene5429_All, Unigene5646_All, Unigene6034_All, Unigene9260_All, Unigene9314_All
88	Basal cell carcinoma	CL1843.Contig3_All, CL2208.Contig1_All, CL2782.Contig1_All, CL2782.Contig2_All, CL2788.Contig2_All, CL3293.Contig1_All, CL3315.Contig3_All, CL3315.Contig4_All, CL3923.Contig1_All, CL3923.Contig3_All, CL4657.Contig2_All, CL5106.Contig1_All, CL5111.Contig1_All, CL63.Contig10_All, CL63.Contig9_All, Unigene1188_All, Unigene11922_All, Unigene150_All, Unigene18477_All, Unigene18765_All, Unigene236_All, Unigene247_All, Unigene2905_All, Unigene3975_All, Unigene4314_All, Unigene4731_All, Unigene5727_All, Unigene7163_All, Unigene7297_All, Unigene766_All, Unigene777_All, Unigene783_All, Unigene8014_All, Unigene8784_All, Unigene8861_All, Unigene9026_All, Unigene9111_All, Unigene9279_All
89	Phosphatidylinositol signaling system	CL1066.Contig1_All, CL1066.Contig2_All, CL1066.Contig3_All, CL127.Contig13_All, CL1286.Contig2_All, CL1823.Contig8_All, CL2035.Contig1_All, CL2035.Contig4_All, CL2131.Contig4_All, CL2131.Contig5_All, CL2131.Contig6_All, CL2131.Contig8_All, CL2182.Contig1_All, CL2182.Contig3_All, CL2182.Contig4_All, CL2468.Contig1_All, CL2468.Contig2_All, CL2551.Contig1_All, CL2552.Contig3_All, CL2835.Contig1_All, CL2835.Contig2_All, CL2835.Contig3_All, CL2835.Contig4_All, CL2930.Contig5_All, CL2941.Contig1_All, CL2944.Contig1_All, CL2944.Contig2_All, CL309.Contig2_All, CL309.Contig3_All, CL309.Contig4_All, CL3142.Contig1_All, CL3142.Contig2_All, CL3163.Contig2_All, CL3410.Contig1_All, CL3410.Contig2_All, CL3505.Contig2_All, CL3578.Contig2_All, CL3578.Contig3_All, CL3578.Contig4_All, CL3667.Contig1_All, CL3667.Contig2_All, CL3667.Contig3_All, CL367.Contig1_All, CL367.Contig2_All, CL367.Contig3_All, CL3687.Contig1_All, CL3687.Contig2_All, CL39.Contig1_All, CL39.Contig2_All, CL39.Contig3_All, CL39.Contig4_All, CL3979.Contig1_All, CL3979.Contig2_All, CL4395.Contig1_All, CL4509.Contig1_All, CL4509.Contig2_All, CL4883.Contig1_All, CL4883.Contig2_All, CL5310.Contig2_All, CL5391.Contig1_All, CL5391.Contig2_All, CL542.Contig1_All, CL542.Contig2_All, CL688.Contig2_All, CL691.Contig1_All, CL691.Contig5_All, CL695.Contig16_All, CL725.Contig1_All, CL728.Contig1_All, CL737.Contig5_All, CL737.Contig6_All, CL772.Contig1_All, CL772.Contig2_All, CL940.Contig3_All, CL940.Contig5_All, CL959.Contig1_All, Unigene10006_All, Unigene10574_All, Unigene11054_All, Unigene11082_All, Unigene11319_All, Unigene12315_All, Unigene12719_All, Unigene12907_All, Unigene1290_All, Unigene13034_All, Unigene16769_All, Unigene18898_All, Unigene18993_All, Unigene3149_All, Unigene3996_All, Unigene4013_All, Unigene4053_All, Unigene4070_All, Unigene4159_All, Unigene4332_All, Unigene5675_All, Unigene5681_All, Unigene5811_All, Unigene6640_All, Unigene6996_All, Unigene6997_All, Unigene7000_All, Unigene8422_All, Unigene9067_All, Unigene9805_All

90	Fatty acid biosynthesis	CL1876.Contig1_All, CL3657.Contig1_All, CL3657.Contig3_All, CL3832.Contig1_All, Unigene11940_All, Unigene16141_All, Unigene16942_All, Unigene17683_All, Unigene17892_All, Unigene18748_All, Unigene20691_All, Unigene3282_All, Unigene3345_All
91	Lysine degradation	CL1433.Contig1_All, CL1433.Contig2_All, CL1687.Contig4_All, CL1946.Contig1_All, CL1946.Contig3_All, CL2029.Contig4_All, CL2029.Contig6_All, CL2309.Contig2_All, CL2309.Contig3_All, CL231.Contig2_All, CL233.Contig1_All, CL2694.Contig1_All, CL2694.Contig3_All, CL2751.Contig1_All, CL2751.Contig2_All, CL29.Contig3_All, CL3027.Contig2_All, CL3027.Contig3_All, CL3027.Contig4_All, CL3045.Contig2_All, CL3046.Contig1_All, CL325.Contig1_All, CL3651.Contig1_All, CL3963.Contig1_All, CL3992.Contig1_All, CL3992.Contig2_All, CL4144.Contig3_All, CL4144.Contig4_All, CL4451.Contig1_All, CL4451.Contig2_All, CL4721.Contig2_All, CL4753.Contig1_All, CL4926.Contig1_All, CL5078.Contig12_All, CL520.Contig2_All, CL5326.Contig14_All, CL5435.Contig1_All, CL598.Contig2_All, Unigene10282_All, Unigene10299_All, Unigene10475_All, Unigene10476_All, Unigene10526_All, Unigene10686_All, Unigene10908_All, Unigene1197_All, Unigene1205_All, Unigene12187_All, Unigene12219_All, Unigene1229_All, Unigene12688_All, Unigene13152_All, Unigene13254_All, Unigene13410_All, Unigene13673_All, Unigene13773_All, Unigene14320_All, Unigene15270_All, Unigene15429_All, Unigene16206_All, Unigene1683_All, Unigene17117_All, Unigene17527_All, Unigene18101_All, Unigene18121_All, Unigene186_All, Unigene18911_All, Unigene19038_All, Unigene19160_All, Unigene2039_All, Unigene2144_All, Unigene214_All, Unigene2212_All, Unigene2215_All, Unigene2514_All, Unigene2525_All, Unigene2853_All, Unigene3028_All, Unigene3030_All, Unigene3040_All, Unigene3071_All, Unigene3450_All, Unigene4088_All, Unigene4251_All, Unigene4269_All, Unigene4853_All, Unigene5053_All, Unigene5075_All, Unigene5153_All, Unigene5305_All, Unigene5554_All, Unigene5717_All, Unigene5892_All, Unigene5989_All, Unigene6052_All, Unigene6105_All, Unigene6167_All, Unigene6360_All, Unigene7348_All, Unigene8563_All, Unigene86_All, Unigene9045_All, Unigene9308_All, Unigene9850_All

92	Pathways in cancer	CL1049.Contig4_All, CL1055.Contig5_All, CL1066.Contig1_All, CL1066.Contig2_All, CL1066.Contig3_All, CL1091.Contig14_All, CL1104.Contig3_All, CL1104.Contig4_All, CL1104.Contig5_All, CL1104.Contig6_All, CL1104.Contig7_All, CL1104.Contig8_All, CL1104.Contig9_All, CL1170.Contig1_All, CL120.Contig2_All, CL1202.Contig2_All, CL1328.Contig2_All, CL1387.Contig1_All, CL1387.Contig3_All, CL1436.Contig2_All, CL1439.Contig1_All, CL1439.Contig2_All, CL1495.Contig1_All, CL1495.Contig2_All, CL1535.Contig3_All, CL1548.Contig2_All, CL1548.Contig3_All, CL1584.Contig2_All, CL1637.Contig1_All, CL1648.Contig1_All, CL1741.Contig1_All, CL1789.Contig1_All, CL1789.Contig2_All, CL1807.Contig1_All, CL1807.Contig2_All, CL1843.Contig3_All, CL1953.Contig1_All, CL1960.Contig1_All, CL1960.Contig2_All, CL2034.Contig1_All, CL2034.Contig2_All, CL2038.Contig1_All, CL2038.Contig7_All, CL2052.Contig1_All, CL2052.Contig2_All, CL2066.Contig1_All, CL209.Contig1_All, CL209.Contig2_All, CL209.Contig3_All, CL209.Contig4_All, CL2099.Contig1_All, CL2170.Contig2_All, CL2170.Contig5_All, CL2182.Contig1_All, CL2182.Contig3_All, CL2182.Contig4_All, CL2208.Contig1_All, CL2287.Contig2_All, CL2287.Contig4_All, CL2331.Contig2_All, CL2331.Contig3_All, CL2346.Contig1_All, CL2346.Contig2_All, CL241.Contig1_All, CL241.Contig7_All, CL2411.Contig1_All, CL2456.Contig3_All, CL2505.Contig3_All, CL2505.Contig4_All, CL2552.Contig3_All, CL2573.Contig2_All, CL2573.Contig3_All, CL2658.Contig1_All, CL2658.Contig2_All, CL271.Contig2_All, CL2711.Contig1_All, CL2711.Contig2_All, CL2782.Contig1_All, CL2782.Contig2_All, CL2788.Contig2_All, CL2793.Contig1_All, CL2793.Contig2_All, CL2882.Contig1_All, CL2882.Contig2_All, CL2927.Contig3_All, CL2935.Contig1_All, CL2935.Contig3_All, CL3078.Contig1_All, CL3162.Contig2_All, CL3235.Contig1_All, CL3235.Contig2_All, CL3270.Contig1_All, CL3270.Contig2_All, CL3293.Contig1_All, CL3315.Contig3_All, CL3315.Contig4_All, CL3327.Contig1_All, CL3327.Contig2_All, CL3371.Contig1_All, CL3373.Contig1_All, CL3373.Contig2_All, CL3436.Contig1_All, CL3436.Contig4_All, CL3436.Contig9_All, CL349.Contig12_All, CL349.Contig3_All, CL349.Contig5_All, CL3567.Contig1_All, CL3567.Contig2_All, CL3567.Contig3_All, CL3618.Contig1_All, CL3632.Contig2_All, CL3632.Contig3_All, CL367.Contig1_All, CL367.Contig2_All, CL367.Contig3_All, CL3788.Contig1_All, CL3796.Contig2_All, CL3796.Contig3_All, CL3802.Contig2_All, CL39.Contig1_All, CL39.Contig2_All, CL39.Contig3_All, CL39.Contig4_All, CL3923.Contig1_All, CL3923.Contig3_All, CL3979.Contig1_All, CL3979.Contig2_All, CL4158.Contig4_All, CL4158.Contig7_All, CL4271.Contig1_All, CL4381.Contig1_All, CL4439.Contig2_All, CL4453.Contig1_All, CL450.Contig10_All, CL4510.Contig1_All, CL4657.Contig2_All, CL470.Contig1_All, CL4736.Contig1_All, CL4736.Contig2_All, CL4787.Contig1_All, CL4896.Contig1_All, CL493.Contig2_All, CL4969.Contig1_All, CL4992.Contig1_All, CL5048.Contig1_All, CL5106.Contig1_All, CL5111.Contig1_All, CL5115.Contig1_All, CL5204.Contig1_All, CL5204.Contig2_All, CL524.Contig10_All, CL524.Contig11_All, CL524.Contig12_All, CL524.Contig13_All, CL524.Contig14_All, CL524.Contig15_All, CL524.Contig16_All, CL524.Contig17_All, CL524.Contig18_All, CL524.Contig1_All, CL524.Contig3_All, CL524.Contig4_All, CL524.Contig5_All, CL524.Contig6_All, CL524.Contig9_All, CL5268.Contig2_All, CL5373.Contig1_All, CL5373.Contig2_All, CL5391.Contig1_All, CL5391.Contig2_All, CL5464.Contig1_All, CL5464.Contig2_All, CL5464.Contig3_All, CL63.Contig10_All, CL63.Contig9_All, CL695.Contig16_All, CL702.Contig2_All, CL702.Contig3_All, CL748.Contig1_All, CL748.Contig3_All, CL880.Contig3_All, CL940.Contig3_All, CL940.Contig5_All, CL95.Contig1_All, Unigene10001_All, Unigene1000_All, Unigene10095_All, Unigene10096_All, Unigene10174_All, Unigene10316_All, Unigene10318_All, Unigene10381_All, Unigene10724_All, Unigene10876_All, Unigene11000_All, Unigene11020_All, Unigene11053_All, Unigene11160_All, Unigene11307_All, Unigene11358_All, Unigene1150_All, Unigene11511_All, Unigene11538_All, Unigene11543_All, Unigene11544_All, Unigene11588_All, Unigene11724_All, Unigene1188_All, Unigene11916_All, Unigene11922_All, Unigene11923_All, Unigene11949_All, Unigene12008_All, Unigene12099_All, Unigene12155_All, Unigene12188_All, Unigene12533_All, Unigene12660_All, Unigene12671_All, Unigene12692_All, Unigene12710_All, Unigene13036_All, Unigene13476_All, Unigene13505_All, Unigene13588_All, Unigene1385_All, Unigene14177_All, Unigene1479_All, Unigene150_All, Unigene15263_All, Unigene15265_All, Unigene15435_All, Unigene15451_All, Unigene15658_All, Unigene15840_All, Unigene1614_All, Unigene16499_All, Unigene1663_All, Unigene1679_All, Unigene16913_All, Unigene16965_All, Unigene16966_All, Unigene1699_All, Unigene1757_All, Unigene17702_All, Unigene17718_All, Unigene17728_All, Unigene17781_All, Unigene18477_All, Unigene18488_All, Unigene18496_All, Unigene18765_All, Unigene18898_All, Unigene19237_All, Unigene19310_All, Unigene19426_All, Unigene19840_All, Unigene198_All, Unigene2050_All, Unigene2190_All, Unigene220_All, Unigene236_All, Unigene2374_All, Unigene247_All, Unigene2503_All, Unigene2560_All, Unigene2749_All, Unigene28_All, Unigene2905_All, Unigene2914_All, Unigene3123_All, Unigene3168_All, Unigene3184_All, Unigene3193_All, Unigene3403_All, Unigene3689_All, Unigene3889_All, Unigene3945_All, Unigene3975_All, Unigene4084_All, Unigene4137_All, Unigene4142_All, Unigene414_All, Unigene4161_All, Unigene4207_All, Unigene4218_All, Unigene4314_All, Unigene4418_All, Unigene4581_All, Unigene4671_All, Unigene4731_All, Unigene4801_All, Unigene4922_All, Unigene5068_All, Unigene5169_All, Unigene5179_All, Unigene54_All, Unigene5727_All, Unigene5781_All, Unigene5789_All, Unigene5799_All, Unigene5808_All, Unigene5870_All, Unigene5896_All, Unigene6041_All, Unigene6222_All, Unigene6614_All, Unigene6780_All, Unigene6797_All, Unigene695_All, Unigene699_All, Unigene7018_All, Unigene7054_All, Unigene7106_All, Unigene7163_All, Unigene7220_All, Unigene7287_All, Unigene7297_All, Unigene7299_All, Unigene740_All, Unigene7548_All, Unigene7560_All, Unigene766_All, Unigene7777_All, Unigene777_All, Unigene783_All, Unigene7983_All, Unigene8003_All, Unigene8014_All, Unigene8046_All, Unigene8102_All, Unigene824_All, Unigene8277_All, Unigene8399_All, Unigene8514_All, Unigene8562_All, Unigene8747_All, Unigene8784_All, Unigene8861_All, Unigene8972_All, Unigene8997_All, Unigene9026_All, Unigene9028_All, Unigene9039_All, Unigene9050_All, Unigene908_All, Unigene90_All, Unigene9111_All, Unigene9279_All, Unigene9284_All, Unigene9298_All, Unigene9611_All, Unigene9641_All, Unigene9740_All, Unigene9877_All, Unigene9988_All

93	ABC transporters	CL197.Contig1_All, CL197.Contig2_All, CL197.Contig3_All, CL227.Contig2_All, CL227.Contig3_All, CL227.Contig4_All, CL227.Contig5_All, CL247.Contig1_All, CL247.Contig2_All, CL2589.Contig1_All, CL2589.Contig2_All, CL2834.Contig1_All, CL2834.Contig2_All, CL3010.Contig1_All, CL3954.Contig1_All, CL4272.Contig2_All, CL4461.Contig1_All, CL4461.Contig2_All, CL4461.Contig3_All, CL460.Contig1_All, CL460.Contig2_All, CL460.Contig3_All, CL4976.Contig2_All, CL5235.Contig1_All, CL5235.Contig2_All, CL5362.Contig2_All, CL738.Contig16_All, CL738.Contig2_All, CL738.Contig3_All, CL798.Contig1_All, CL798.Contig2_All, CL798.Contig3_All, CL89.Contig1_All, CL89.Contig2_All, Unigene11915_All, Unigene15238_All, Unigene15239_All, Unigene18165_All, Unigene18195_All, Unigene18196_All, Unigene18766_All, Unigene2032_All, Unigene2054_All, Unigene2699_All, Unigene3196_All, Unigene3824_All, Unigene4537_All, Unigene4_All, Unigene5058_All, Unigene5639_All, Unigene5768_All, Unigene5_All, Unigene6229_All, Unigene6_All, Unigene7006_All, Unigene7082_All, Unigene7211_All, Unigene7651_All, Unigene784_All, Unigene7979_All, Unigene7_All, Unigene8194_All, Unigene8621_All, Unigene8910_All, Unigene9025_All, Unigene9078_All
94	Collecting duct acid secretion	CL2150.Contig3_All, CL2634.Contig1_All, CL2634.Contig2_All, CL3091.Contig1_All, CL3091.Contig2_All, CL3091.Contig3_All, CL3091.Contig4_All, CL4090.Contig2_All, CL4459.Contig1_All, CL974.Contig2_All, Unigene10026_All, Unigene10483_All, Unigene11240_All, Unigene11899_All, Unigene1189_All, Unigene12028_All, Unigene12168_All, Unigene12209_All, Unigene12302_All, Unigene12423_All, Unigene13508_All, Unigene13589_All, Unigene15969_All, Unigene1741_All, Unigene17602_All, Unigene18707_All, Unigene2002_All, Unigene2003_All, Unigene3351_All, Unigene4211_All, Unigene7061_All, Unigene925_All, Unigene9668_All
95	Caffeine metabolism	CL1305.Contig1_All, CL1711.Contig1_All, CL1711.Contig2_All, CL2532.Contig1_All, CL2707.Contig1_All, CL3511.Contig2_All, CL731.Contig1_All, Unigene16322_All, Unigene18597_All, Unigene432_All, Unigene5060_All, Unigene5600_All, Unigene7663_All
96	Osteoclast differentiation	CL1066.Contig1_All, CL1066.Contig2_All, CL1066.Contig3_All, CL1535.Contig3_All, CL1831.Contig1_All, CL1831.Contig2_All, CL2129.Contig4_All, CL257.Contig1_All, CL257.Contig2_All, CL3079.Contig1_All, CL3079.Contig2_All, CL3079.Contig3_All, CL3079.Contig6_All, CL3567.Contig1_All, CL3567.Contig2_All, CL3567.Contig3_All, CL367.Contig1_All, CL367.Contig2_All, CL367.Contig3_All, CL3920.Contig2_All, CL4057.Contig1_All, CL4190.Contig1_All, CL4271.Contig1_All, CL4362.Contig1_All, CL4362.Contig2_All, CL4521.Contig1_All, CL4521.Contig2_All, CL4521.Contig3_All, CL4521.Contig5_All, CL4736.Contig1_All, CL4736.Contig2_All, CL4787.Contig1_All, CL4815.Contig1_All, CL4815.Contig2_All, CL4966.Contig1_All, CL504.Contig1_All, CL504.Contig2_All, CL5064.Contig2_All, CL5115.Contig1_All, CL695.Contig16_All, Unigene10174_All, Unigene10296_All, Unigene13853_All, Unigene1479_All, Unigene1757_All, Unigene18114_All, Unigene220_All, Unigene2749_All, Unigene3131_All, Unigene3982_All, Unigene5869_All, Unigene7217_All, Unigene763_All, Unigene8046_All
97	Pentose phosphate pathway	CL2512.Contig2_All, CL4091.Contig1_All, CL4091.Contig2_All, CL4358.Contig1_All, Unigene10489_All, Unigene11782_All, Unigene11830_All, Unigene11894_All, Unigene12035_All, Unigene12066_All, Unigene12144_All, Unigene12227_All, Unigene12267_All, Unigene12372_All, Unigene12580_All, Unigene13066_All, Unigene13088_All, Unigene13119_All, Unigene13375_All, Unigene14160_All, Unigene1538_All, Unigene16591_All, Unigene16824_All, Unigene17282_All, Unigene17700_All, Unigene17838_All, Unigene18003_All, Unigene18004_All, Unigene18248_All, Unigene18487_All, Unigene18651_All, Unigene19184_All, Unigene3105_All, Unigene4267_All, Unigene6526_All, Unigene7735_All, Unigene7814_All
98	Proteasome	CL2483.Contig2_All, CL2513.Contig1_All, CL2513.Contig2_All, CL2855.Contig1_All, CL2855.Contig3_All, CL3332.Contig1_All, CL4003.Contig1_All, CL4003.Contig2_All, CL5242.Contig1_All, CL619.Contig1_All, Unigene10165_All, Unigene10166_All, Unigene10224_All, Unigene10225_All, Unigene10239_All, Unigene10240_All, Unigene10407_All, Unigene10408_All, Unigene10602_All, Unigene11073_All, Unigene11928_All, Unigene11936_All, Unigene11956_All, Unigene11964_All, Unigene11999_All, Unigene12009_All, Unigene12165_All, Unigene12197_All, Unigene12323_All, Unigene12345_All, Unigene12364_All, Unigene12393_All, Unigene12467_All, Unigene12472_All, Unigene12787_All, Unigene13030_All, Unigene13077_All, Unigene13108_All, Unigene13158_All, Unigene13168_All, Unigene13185_All, Unigene14201_All, Unigene2306_All, Unigene4813_All, Unigene4891_All, Unigene6066_All, Unigene6964_All, Unigene7198_All, Unigene7327_All, Unigene7526_All, Unigene7817_All, Unigene7855_All, Unigene8804_All, Unigene8924_All, Unigene9194_All
99	Complement and coagulation cascades	CL1085.Contig3_All, CL1444.Contig3_All, CL2156.Contig2_All, CL326.Contig1_All, CL3298.Contig1_All, CL4597.Contig2_All, CL4690.Contig1_All, CL4690.Contig2_All, CL4699.Contig1_All, CL4984.Contig2_All, Unigene10345_All, Unigene10833_All, Unigene10848_All, Unigene10991_All, Unigene1103_All, Unigene1163_All, Unigene11660_All, Unigene12605_All, Unigene13162_All, Unigene15513_All, Unigene17970_All, Unigene17986_All, Unigene18112_All, Unigene352_All, Unigene3902_All, Unigene5877_All, Unigene6063_All, Unigene7108_All, Unigene71_All, Unigene8217_All, Unigene8848_All

100	Axon guidance	CL1156.Contig2_All, CL1156.Contig5_All, CL1227.Contig1_All, CL1227.Contig2_All, CL1335.Contig1_All, CL1535.Contig3_All, CL1681.Contig4_All, CL1681.Contig5_All, CL1764.Contig2_All, CL1831.Contig1_All, CL1831.Contig2_All, CL1843.Contig3_All, CL2066.Contig1_All, CL2224.Contig2_All, CL2224.Contig3_All, CL2224.Contig4_All, CL2253.Contig2_All, CL2346.Contig1_All, CL2346.Contig2_All, CL2541.Contig1_All, CL2541.Contig2_All, CL257.Contig1_All, CL257.Contig2_All, CL2626.Contig1_All, CL2658.Contig1_All, CL2658.Contig2_All, CL271.Contig2_All, CL2722.Contig1_All, CL2977.Contig1_All, CL2977.Contig2_All, CL3042.Contig1_All, CL3042.Contig3_All, CL3196.Contig1_All, CL3196.Contig2_All, CL3321.Contig3_All, CL3636.Contig1_All, CL3636.Contig4_All, CL3636.Contig5_All, CL3636.Contig6_All, CL3636.Contig8_All, CL3636.Contig9_All, CL3823.Contig1_All, CL3823.Contig2_All, CL4095.Contig1_All, CL4190.Contig1_All, CL4381.Contig1_All, CL4439.Contig2_All, CL4521.Contig1_All, CL4521.Contig2_All, CL4521.Contig3_All, CL4521.Contig5_All, CL4941.Contig1_All, CL5005.Contig1_All, CL5005.Contig2_All, CL5064.Contig2_All, CL5469.Contig2_All, CL566.Contig1_All, CL748.Contig1_All, CL748.Contig3_All, CL95.Contig1_All, CL986.Contig2_All, Unigene1000_All, Unigene1030_All, Unigene10682_All, Unigene10775_All, Unigene109_All, Unigene11052_All, Unigene1123_All, Unigene11307_All, Unigene11607_All, Unigene11922_All, Unigene1227_All, Unigene12344_All, Unigene12671_All, Unigene13853_All, Unigene1629_All, Unigene1679_All, Unigene16906_All, Unigene16965_All, Unigene17102_All, Unigene17637_All, Unigene17967_All, Unigene18644_All, Unigene198_All, Unigene2015_All, Unigene21521_All, Unigene236_All, Unigene247_All, Unigene2980_All, Unigene3945_All, Unigene4068_All, Unigene4081_All, Unigene4207_All, Unigene4244_All, Unigene5052_All, Unigene5120_All, Unigene5521_All, Unigene5808_All, Unigene6015_All, Unigene6017_All, Unigene6217_All, Unigene6245_All, Unigene6432_All, Unigene7011_All, Unigene7013_All, Unigene7063_All, Unigene7286_All, Unigene7297_All, Unigene7983_All, Unigene8044_All, Unigene8076_All, Unigene8725_All, Unigene8784_All, Unigene9050_All, Unigene9200_All, Unigene9719_All, Unigene9877_All
101	Base excision repair	CL1070.Contig6_All, CL1070.Contig7_All, CL1395.Contig2_All, CL2986.Contig2_All, CL4232.Contig1_All, CL4232.Contig2_All, CL4278.Contig2_All, CL4567.Contig2_All, CL4948.Contig1_All, CL4989.Contig1_All, CL4989.Contig2_All, CL5326.Contig17_All, CL5427.Contig2_All, CL91.Contig1_All, Unigene12361_All, Unigene13367_All, Unigene15790_All, Unigene17292_All, Unigene18127_All, Unigene18761_All, Unigene20279_All, Unigene2890_All, Unigene3632_All, Unigene4102_All, Unigene4925_All, Unigene5175_All, Unigene6911_All, Unigene8228_All, Unigene9237_All, Unigene9545_All
102	Progesterone-mediated oocyte maturation	CL1066.Contig1_All, CL1066.Contig2_All, CL1066.Contig3_All, CL1452.Contig1_All, CL1660.Contig10_All, CL1660.Contig15_All, CL1660.Contig16_All, CL1660.Contig1_All, CL1660.Contig3_All, CL1660.Contig5_All, CL1660.Contig6_All, CL1660.Contig7_All, CL1660.Contig8_All, CL1660.Contig9_All, CL1766.Contig1_All, CL241.Contig1_All, CL241.Contig7_All, CL2416.Contig1_All, CL2770.Contig1_All, CL2770.Contig2_All, CL2809.Contig2_All, CL2809.Contig3_All, CL2809.Contig8_All, CL2978.Contig2_All, CL2978.Contig3_All, CL3263.Contig1_All, CL367.Contig1_All, CL367.Contig2_All, CL367.Contig3_All, CL4005.Contig2_All, CL4005.Contig3_All, CL4262.Contig2_All, CL440.Contig3_All, CL440.Contig5_All, CL440.Contig6_All, CL4439.Contig2_All, CL4615.Contig2_All, CL4787.Contig1_All, CL4966.Contig1_All, CL5064.Contig2_All, CL5115.Contig1_All, CL5206.Contig1_All, CL5218.Contig1_All, CL5218.Contig2_All, CL5304.Contig1_All, CL570.Contig1_All, CL570.Contig2_All, CL695.Contig16_All, CL702.Contig2_All, CL702.Contig3_All, CL710.Contig2_All, CL940.Contig3_All, CL940.Contig5_All, CL95.Contig1_All, Unigene10019_All, Unigene10252_All, Unigene10253_All, Unigene11000_All, Unigene11053_All, Unigene1109_All, Unigene11227_All, Unigene11228_All, Unigene1125_All, Unigene1181_All, Unigene12161_All, Unigene1229_All, Unigene12469_All, Unigene12671_All, Unigene13421_All, Unigene15263_All, Unigene15265_All, Unigene16349_All, Unigene16499_All, Unigene17781_All, Unigene17863_All, Unigene18319_All, Unigene18584_All, Unigene20083_All, Unigene20226_All, Unigene2029_All, Unigene220_All, Unigene2700_All, Unigene2749_All, Unigene2863_All, Unigene3779_All, Unigene4112_All, Unigene47_All, Unigene4808_All, Unigene5521_All, Unigene5896_All, Unigene5897_All, Unigene6217_All, Unigene700_All, Unigene756_All, Unigene7747_All, Unigene7869_All, Unigene8075_All, Unigene8747_All, Unigene93_All, Unigene9579_All, Unigene9733_All
103	Measles	CL1054.Contig1_All, CL1054.Contig2_All, CL1066.Contig1_All, CL1066.Contig2_All, CL1066.Contig3_All, CL1843.Contig3_All, CL1925.Contig1_All, CL1953.Contig1_All, CL2038.Contig1_All, CL2038.Contig7_All, CL2165.Contig2_All, CL2306.Contig2_All, CL257.Contig1_All, CL257.Contig2_All, CL263.Contig3_All, CL263.Contig5_All, CL2686.Contig1_All, CL2686.Contig2_All, CL3047.Contig1_All, CL3047.Contig2_All, CL3083.Contig1_All, CL3083.Contig3_All, CL345.Contig1_All, CL3632.Contig2_All, CL3632.Contig3_All, CL367.Contig1_All, CL367.Contig2_All, CL367.Contig3_All, CL3853.Contig1_All, CL4855.Contig1_All, CL4855.Contig2_All, CL5051.Contig2_All, CL5256.Contig1_All, CL5256.Contig2_All, CL5256.Contig4_All, CL558.Contig1_All, CL558.Contig2_All, CL558.Contig4_All, CL695.Contig16_All, Unigene10015_All, Unigene10258_All, Unigene10619_All, Unigene10837_All, Unigene11152_All, Unigene11153_All, Unigene11781_All, Unigene11922_All, Unigene12006_All, Unigene12099_All, Unigene12259_All, Unigene1247_All, Unigene12591_All, Unigene12827_All, Unigene12894_All, Unigene13134_All, Unigene13513_All, Unigene14208_All, Unigene15587_All, Unigene15863_All, Unigene16093_All, Unigene1757_All, Unigene17810_All, Unigene18535_All, Unigene20095_All, Unigene236_All, Unigene2374_All, Unigene247_All, Unigene2749_All, Unigene3131_All, Unigene3153_All, Unigene3168_All, Unigene5896_All, Unigene6041_All, Unigene7297_All, Unigene7299_All, Unigene8056_All, Unigene8246_All, Unigene8784_All, Unigene9971_All
104	Nucleotide excision repair	CL1395.Contig2_All, CL2217.Contig1_All, CL2217.Contig2_All, CL2217.Contig3_All, CL2340.Contig2_All, CL2986.Contig2_All, CL3372.Contig1_All, CL4023.Contig1_All, CL4023.Contig2_All, CL4278.Contig2_All, CL4288.Contig1_All, CL4288.Contig2_All, CL4288.Contig3_All, CL4319.Contig2_All, CL4989.Contig1_All, CL4989.Contig2_All, CL856.Contig1_All, CL91.Contig1_All, Unigene10181_All, Unigene10182_All, Unigene10261_All, Unigene10876_All, Unigene12220_All, Unigene12361_All, Unigene12386_All, Unigene15790_All, Unigene1638_All, Unigene1696_All, Unigene17292_All, Unigene18127_All, Unigene18761_All, Unigene20279_All, Unigene2243_All, Unigene2862_All, Unigene2890_All, Unigene4102_All, Unigene567_All, Unigene6125_All, Unigene7699_All, Unigene8025_All, Unigene806_All, Unigene8228_All, Unigene8709_All, Unigene8784_All, Unigene9072_All, Unigene9245_All, Unigene9503_All

105	Glyoxylate and dicarboxylate metabolism	CL3283.Contig1_All, CL4534.Contig2_All, CL4534.Contig3_All, CL4686.Contig2_All, CL4971.Contig2_All, CL5301.Contig1_All, CL5301.Contig2_All, Unigene10287_All, Unigene10412_All, Unigene10732_All, Unigene10818_All, Unigene10909_All, Unigene10910_All, Unigene10929_All, Unigene10954_All, Unigene11253_All, Unigene11254_All, Unigene11277_All, Unigene11278_All, Unigene11279_All, Unigene11838_All, Unigene11854_All, Unigene12053_All, Unigene12071_All, Unigene12169_All, Unigene12219_All, Unigene12293_All, Unigene12362_All, Unigene12470_All, Unigene12585_All, Unigene12587_All, Unigene12738_All, Unigene12869_All, Unigene13080_All, Unigene13117_All, Unigene13431_All, Unigene13673_All, Unigene14111_All, Unigene15931_All, Unigene16508_All, Unigene17971_All, Unigene18234_All, Unigene18238_All, Unigene1980_All, Unigene3655_All, Unigene5684_All, Unigene7886_All
106	Pyrimidine metabolism	CL1156.Contig3_All, CL1258.Contig1_All, CL1258.Contig2_All, CL1356.Contig1_All, CL1356.Contig2_All, CL1395.Contig2_All, CL1411.Contig1_All, CL1411.Contig8_All, CL1683.Contig2_All, CL1683.Contig7_All, CL1986.Contig1_All, CL2272.Contig1_All, CL2702.Contig1_All, CL2702.Contig2_All, CL3028.Contig1_All, CL322.Contig4_All, CL322.Contig5_All, CL322.Contig6_All, CL3251.Contig1_All, CL3251.Contig2_All, CL3420.Contig1_All, CL3576.Contig1_All, CL3635.Contig3_All, CL3657.Contig3_All, CL3664.Contig1_All, CL3664.Contig2_All, CL372.Contig1_All, CL3792.Contig3_All, CL3878.Contig2_All, CL4195.Contig2_All, CL4278.Contig2_All, CL4617.Contig2_All, CL4850.Contig1_All, CL4850.Contig3_All, CL5004.Contig1_All, CL5266.Contig1_All, CL5266.Contig2_All, CL5461.Contig2_All, CL598.Contig1_All, CL598.Contig2_All, CL63.Contig10_All, CL63.Contig9_All, CL893.Contig1_All, CL91.Contig1_All, Unigene10164_All, Unigene10230_All, Unigene10294_All, Unigene10549_All, Unigene10735_All, Unigene10742_All, Unigene11026_All, Unigene11027_All, Unigene1105_All, Unigene11623_All, Unigene11741_All, Unigene11970_All, Unigene12017_All, Unigene12027_All, Unigene1205_All, Unigene12060_All, Unigene12080_All, Unigene12096_All, Unigene12117_All, Unigene12396_All, Unigene12479_All, Unigene12488_All, Unigene12530_All, Unigene12645_All, Unigene1278_All, Unigene12887_All, Unigene12915_All, Unigene13002_All, Unigene13218_All, Unigene13256_All, Unigene13284_All, Unigene13623_All, Unigene14002_All, Unigene14516_All, Unigene1464_All, Unigene1505_All, Unigene1570_All, Unigene15790_All, Unigene15827_All, Unigene17292_All, Unigene17448_All, Unigene18127_All, Unigene18801_All, Unigene19657_All, Unigene20279_All, Unigene2037_All, Unigene2131_All, Unigene2156_All, Unigene2451_All, Unigene24_All, Unigene2513_All, Unigene2754_All, Unigene3081_All, Unigene3142_All, Unigene3288_All, Unigene3390_All, Unigene33_All, Unigene3419_All, Unigene3659_All, Unigene3831_All, Unigene3890_All, Unigene4102_All, Unigene4251_All, Unigene5160_All, Unigene5642_All, Unigene5745_All, Unigene6374_All, Unigene6749_All, Unigene7192_All, Unigene7296_All, Unigene7357_All, Unigene7750_All, Unigene7822_All, Unigene7993_All, Unigene8008_All, Unigene8228_All, Unigene8236_All, Unigene8370_All, Unigene9107_All, Unigene9758_All
107	Protein processing in endoplasmic reticulum	CL1054.Contig1_All, CL1054.Contig2_All, CL1207.Contig2_All, CL1239.Contig2_All, CL1239.Contig4_All, CL1436.Contig1_All, CL1507.Contig1_All, CL1507.Contig2_All, CL1530.Contig1_All, CL1581.Contig1_All, CL1581.Contig2_All, CL1632.Contig1_All, CL1659.Contig1_All, CL1776.Contig1_All, CL1925.Contig1_All, CL2.Contig1_All, CL2032.Contig6_All, CL2165.Contig2_All, CL2237.Contig5_All, CL2403.Contig1_All, CL2426.Contig1_All, CL2426.Contig2_All, CL2503.Contig1_All, CL2608.Contig1_All, CL2686.Contig1_All, CL2686.Contig2_All, CL2826.Contig1_All, CL2924.Contig1_All, CL2924.Contig2_All, CL2924.Contig3_All, CL3027.Contig2_All, CL3055.Contig1_All, CL3071.Contig1_All, CL3071.Contig2_All, CL3092.Contig1_All, CL3092.Contig2_All, CL3290.Contig2_All, CL3290.Contig3_All, CL3332.Contig2_All, CL345.Contig1_All, CL3742.Contig1_All, CL3748.Contig1_All, CL383.Contig1_All, CL383.Contig2_All, CL4160.Contig1_All, CL4247.Contig2_All, CL4247.Contig3_All, CL4338.Contig1_All, CL4338.Contig2_All, CL4338.Contig3_All, CL4514.Contig2_All, CL4519.Contig1_All, CL4537.Contig1_All, CL4633.Contig1_All, CL4633.Contig2_All, CL4659.Contig1_All, CL4666.Contig1_All, CL4666.Contig2_All, CL4738.Contig1_All, CL4807.Contig1_All, CL4807.Contig2_All, CL4815.Contig1_All, CL4815.Contig2_All, CL4917.Contig2_All, CL4938.Contig1_All, CL5034.Contig1_All, CL5034.Contig2_All, CL5043.Contig1_All, CL5043.Contig2_All, CL5081.Contig2_All, CL5115.Contig1_All, CL5256.Contig1_All, CL5256.Contig2_All, CL5256.Contig4_All, CL5260.Contig1_All, CL558.Contig1_All, CL558.Contig2_All, CL558.Contig4_All, CL702.Contig2_All, CL702.Contig3_All, CL906.Contig3_All, CL906.Contig8_All, CL97.Contig1_All, Unigene10175_All, Unigene10245_All, Unigene10246_All, Unigene10374_All, Unigene10390_All, Unigene10420_All, Unigene10421_All, Unigene10433_All, Unigene10557_All, Unigene10585_All, Unigene10586_All, Unigene10702_All, Unigene10770_All, Unigene10837_All, Unigene10876_All, Unigene11000_All, Unigene11037_All, Unigene11040_All, Unigene11053_All, Unigene11112_All, Unigene11113_All, Unigene1119_All, Unigene11222_All, Unigene11258_All, Unigene11295_All, Unigene11342_All, Unigene11343_All, Unigene11411_All, Unigene11437_All, Unigene11438_All, Unigene11744_All, Unigene11777_All, Unigene11810_All, Unigene11828_All, Unigene11845_All, Unigene11884_All, Unigene11908_All, Unigene11911_All, Unigene12001_All, Unigene12006_All, Unigene12045_All, Unigene12072_All, Unigene12157_All, Unigene12259_All, Unigene12386_All, Unigene12461_All, Unigene12503_All, Unigene12542_All, Unigene12621_All, Unigene12713_All, Unigene12735_All, Unigene12740_All, Unigene12781_All, Unigene12786_All, Unigene12888_All, Unigene12890_All, Unigene12903_All, Unigene12989_All, Unigene12996_All, Unigene13172_All, Unigene13228_All, Unigene13331_All, Unigene13644_All, Unigene14208_All, Unigene14416_All, Unigene15263_All, Unigene15265_All, Unigene15566_All, Unigene15587_All, Unigene155_All, Unigene15863_All, Unigene16093_All, Unigene16098_All, Unigene16499_All, Unigene1661_All, Unigene16855_All, Unigene170_All, Unigene17781_All, Unigene18245_All, Unigene18246_All, Unigene18360_All, Unigene18361_All, Unigene18535_All, Unigene19481_All, Unigene19483_All, Unigene20308_All, Unigene220_All, Unigene2261_All, Unigene2656_All, Unigene2662_All, Unigene2759_All, Unigene3099_All, Unigene3138_All, Unigene363_All, Unigene3811_All, Unigene3814_All, Unigene3976_All, Unigene4171_All, Unigene4359_All, Unigene45_All, Unigene4975_All, Unigene5105_All, Unigene5907_All, Unigene6648_All, Unigene6797_All, Unigene6801_All, Unigene6931_All, Unigene7120_All, Unigene7245_All, Unigene7466_All, Unigene7667_All, Unigene7671_All, Unigene7716_All, Unigene7723_All, Unigene7732_All, Unigene782_All, Unigene7905_All, Unigene8291_All, Unigene8627_All, Unigene8710_All, Unigene9012_All, Unigene9157_All, Unigene9705_All, Unigene9859_All, Unigene9940_All, Unigene9957_All, Unigene9971_All, Unigene9993_All

108	Cytokine-cytokine receptor interaction	CL1548.Contig2_All, CL1548.Contig3_All, CL2658.Contig1_All, CL2658.Contig2_All, CL2992.Contig1_All, CL4271.Contig1_All, CL4271.Contig2_All, CL4381.Contig1_All, CL4496.Contig3_All, CL5106.Contig1_All, Unigene11589_All, Unigene12435_All, Unigene12810_All, Unigene1679_All, Unigene1767_All, Unigene20226_All, Unigene2110_All, Unigene3967_All, Unigene6916_All, Unigene699_All, Unigene833_All
109	Allograft rejection	Unigene20260_All
110	Graft-versus-host disease	Unigene20260_All
111	Selenocompound metabolism	CL3127.Contig1_All, CL4424.Contig1_All, CL4424.Contig2_All, CL4424.Contig3_All, CL4424.Contig4_All, Unigene10190_All, Unigene10191_All, Unigene10425_All, Unigene20765_All, Unigene3188_All, Unigene3831_All
112	Notch signaling pathway	CL1193.Contig1_All, CL1193.Contig2_All, CL1193.Contig3_All, CL1339.Contig2_All, CL1448.Contig1_All, CL1534.Contig2_All, CL2170.Contig2_All, CL2170.Contig5_All, CL2245.Contig1_All, CL2245.Contig2_All, CL2245.Contig3_All, CL2563.Contig2_All, CL2824.Contig3_All, CL320.Contig1_All, CL320.Contig2_All, CL3350.Contig1_All, CL3350.Contig2_All, CL371.Contig1_All, CL4657.Contig2_All, CL4802.Contig1_All, CL4802.Contig2_All, CL4880.Contig1_All, CL4880.Contig2_All, CL5432.Contig1_All, CL823.Contig1_All, CL874.Contig10_All, CL874.Contig8_All, Unigene1040_All, Unigene1663_All, Unigene18054_All, Unigene3080_All, Unigene32_All, Unigene3314_All, Unigene3359_All, Unigene4075_All, Unigene4244_All, Unigene4432_All, Unigene4940_All, Unigene5064_All, Unigene5179_All, Unigene5341_All, Unigene54_All, Unigene5781_All, Unigene7093_All, Unigene8986_All, Unigene9065_All, Unigene9097_All, Unigene9641_All
113	Insulin signaling pathway	CL1009.Contig1_All, CL1015.Contig11_All, CL1015.Contig27_All, CL1015.Contig9_All, CL102.Contig1_All, CL102.Contig2_All, CL1049.Contig4_All, CL1066.Contig1_All, CL1066.Contig2_All, CL1066.Contig3_All, CL1080.Contig2_All, CL1104.Contig3_All, CL1104.Contig4_All, CL1104.Contig5_All, CL1104.Contig6_All, CL1104.Contig7_All, CL1104.Contig8_All, CL1104.Contig9_All, CL1286.Contig2_All, CL1385.Contig1_All, CL1385.Contig2_All, CL1452.Contig1_All, CL1563.Contig2_All, CL1764.Contig2_All, CL1807.Contig1_All, CL1807.Contig2_All, CL1823.Contig8_All, CL1843.Contig3_All, CL1874.Contig2_All, CL1876.Contig1_All, CL1953.Contig1_All, CL2131.Contig4_All, CL2131.Contig5_All, CL2131.Contig6_All, CL2131.Contig8_All, CL2187.Contig2_All, CL2207.Contig2_All, CL241.Contig1_All, CL241.Contig7_All, CL2430.Contig3_All, CL244.Contig2_All, CL2468.Contig1_All, CL2468.Contig2_All, CL2505.Contig3_All, CL2505.Contig4_All, CL265.Contig2_All, CL265.Contig3_All, CL2770.Contig1_All, CL2770.Contig2_All, CL2839.Contig2_All, CL2839.Contig3_All, CL2839.Contig4_All, CL2839.Contig5_All, CL2839.Contig7_All, CL2843.Contig2_All, CL2941.Contig1_All, CL3093.Contig1_All, CL3112.Contig3_All, CL3657.Contig1_All, CL3657.Contig3_All, CL3667.Contig1_All, CL3667.Contig2_All, CL3667.Contig3_All, CL367.Contig1_All, CL367.Contig2_All, CL367.Contig3_All, CL3689.Contig2_All, CL3796.Contig2_All, CL3796.Contig3_All, CL3832.Contig1_All, CL400.Contig1_All, CL400.Contig2_All, CL4002.Contig1_All, CL4040.Contig1_All, CL408.Contig1_All, CL408.Contig3_All, CL408.Contig4_All, CL4158.Contig4_All, CL4158.Contig7_All, CL4175.Contig1_All, CL4175.Contig2_All, CL4175.Contig3_All, CL4224.Contig1_All, CL4224.Contig2_All, CL4224.Contig3_All, CL4395.Contig1_All, CL4439.Contig2_All, CL4509.Contig1_All, CL4509.Contig2_All, CL4529.Contig2_All, CL4559.Contig2_All, CL4559.Contig3_All, CL4687.Contig1_All, CL4687.Contig2_All, CL4787.Contig1_All, CL4813.Contig1_All, CL4813.Contig2_All, CL4813.Contig3_All, CL4883.Contig1_All, CL4883.Contig2_All, CL5115.Contig1_All, CL530.Contig1_All, CL5304.Contig1_All, CL5382.Contig1_All, CL578.Contig2_All, CL578.Contig3_All, CL695.Contig16_All, CL741.Contig2_All, CL772.Contig1_All, CL772.Contig2_All, CL903.Contig1_All, CL940.Contig3_All, CL940.Contig5_All, CL95.Contig1_All, CL95.Contig2_All, Unigene10006_All, Unigene10029_All, Unigene10174_All, Unigene10188_All, Unigene10574_All, Unigene10676_All, Unigene10677_All, Unigene10766_All, Unigene10875_All, Unigene11054_All, Unigene1168_All, Unigene11782_All, Unigene11811_All, Unigene11922_All, Unigene11940_All, Unigene12223_All, Unigene12295_All, Unigene12315_All, Unigene12671_All, Unigene12719_All, Unigene1279_All, Unigene12928_All, Unigene13036_All, Unigene13135_All, Unigene1512_All, Unigene16141_All, Unigene16769_All, Unigene16942_All, Unigene17683_All, Unigene17892_All, Unigene1819_All, Unigene18383_All, Unigene18748_All, Unigene1941_All, Unigene19424_All, Unigene20691_All, Unigene220_All, Unigene2233_All, Unigene236_All, Unigene247_All, Unigene2742_All, Unigene2749_All, Unigene2956_All, Unigene3282_All, Unigene3345_All, Unigene3779_All, Unigene3836_All, Unigene3996_All, Unigene4013_All, Unigene4022_All, Unigene4112_All, Unigene4935_All, Unigene4970_All, Unigene5675_All, Unigene5722_All, Unigene5811_All, Unigene6007_All, Unigene6101_All, Unigene6655_All, Unigene695_All, Unigene699_All, Unigene7016_All, Unigene7297_All, Unigene7704_All, Unigene7745_All, Unigene7759_All, Unigene8046_All, Unigene8747_All, Unigene8784_All, Unigene9730_All, Unigene9733_All, Unigene9765_All, Unigene9904_All, Unigene9987_All

114	Epstein-Barr virus infection	CL1054.Contig1_All, CL1054.Contig2_All, CL1066.Contig1_All, CL1066.Contig2_All, CL1066.Contig3_All, CL1188.Contig1_All, CL1188.Contig2_All, CL1339.Contig2_All, CL1411.Contig1_All, CL1411.Contig8_All, CL1452.Contig1_All, CL1683.Contig2_All, CL1683.Contig7_All, CL1843.Contig3_All, CL1925.Contig1_All, CL1966.Contig2_All, CL2068.Contig5_All, CL2129.Contig4_All, CL2144.Contig1_All, CL2144.Contig2_All, CL2165.Contig2_All, CL2170.Contig2_All, CL2170.Contig5_All, CL2245.Contig1_All, CL2245.Contig2_All, CL2245.Contig3_All, CL2306.Contig2_All, CL2483.Contig2_All, CL249.Contig1_All, CL249.Contig3_All, CL249.Contig5_All, CL249.Contig6_All, CL2513.Contig1_All, CL2513.Contig2_All, CL2686.Contig1_All, CL2686.Contig2_All, CL3165.Contig1_All, CL3165.Contig2_All, CL3165.Contig3_All, CL3215.Contig1_All, CL3215.Contig2_All, CL3215.Contig3_All, CL322.Contig4_All, CL322.Contig5_All, CL322.Contig6_All, CL3251.Contig1_All, CL3251.Contig2_All, CL3290.Contig2_All, CL3290.Contig3_All, CL3332.Contig1_All, CL3420.Contig1_All, CL345.Contig1_All, CL3561.Contig1_All, CL3561.Contig2_All, CL3635.Contig3_All, CL3657.Contig3_All, CL3664.Contig1_All, CL3664.Contig2_All, CL367.Contig1_All, CL367.Contig2_All, CL367.Contig3_All, CL3792.Contig3_All, CL3979.Contig1_All, CL3979.Contig2_All, CL4003.Contig1_All, CL4003.Contig2_All, CL4103.Contig1_All, CL4555.Contig1_All, CL4597.Contig2_All, CL4736.Contig1_All, CL4736.Contig2_All, CL4815.Contig1_All, CL4815.Contig2_All, CL4874.Contig1_All, CL4966.Contig1_All, CL5004.Contig1_All, CL5115.Contig1_All, CL5242.Contig1_All, CL5256.Contig1_All, CL5256.Contig2_All, CL5256.Contig4_All, CL5461.Contig2_All, CL558.Contig1_All, CL558.Contig2_All, CL558.Contig4_All, CL598.Contig1_All, CL598.Contig2_All, CL619.Contig1_All, CL695.Contig16_All, CL940.Contig3_All, CL940.Contig5_All, Unigene10224_All, Unigene10225_All, Unigene10407_All, Unigene10408_All, Unigene10602_All, Unigene10619_All, Unigene10837_All, Unigene11222_All, Unigene11623_All, Unigene11810_All, Unigene11922_All, Unigene11936_All, Unigene12017_All, Unigene1205_All, Unigene12060_All, Unigene12080_All, Unigene12096_All, Unigene12117_All, Unigene12139_All, Unigene12161_All, Unigene12197_All, Unigene12259_All, Unigene12323_All, Unigene12479_All, Unigene1247_All, Unigene12488_All, Unigene12522_All, Unigene12591_All, Unigene1278_All, Unigene13002_All, Unigene13077_All, Unigene13108_All, Unigene13134_All, Unigene13158_All, Unigene13185_All, Unigene1326_All, Unigene13284_All, Unigene13421_All, Unigene14201_All, Unigene14208_All, Unigene1479_All, Unigene1538_All, Unigene15587_All, Unigene15827_All, Unigene15863_All, Unigene16093_All, Unigene1663_All, Unigene1757_All, Unigene18535_All, Unigene18801_All, Unigene19657_All, Unigene20260_All, Unigene2131_All, Unigene220_All, Unigene236_All, Unigene247_All, Unigene24_All, Unigene2513_All, Unigene2749_All, Unigene3081_All, Unigene3131_All, Unigene3288_All, Unigene3390_All, Unigene33_All, Unigene3419_All, Unigene3659_All, Unigene3779_All, Unigene4112_All, Unigene4251_All, Unigene4359_All, Unigene5179_All, Unigene5198_All, Unigene54_All, Unigene5745_All, Unigene5781_All, Unigene5896_All, Unigene6066_All, Unigene6526_All, Unigene6964_All, Unigene6977_All, Unigene7192_All, Unigene7296_All, Unigene7297_All, Unigene7466_All, Unigene7526_All, Unigene7702_All, Unigene7750_All, Unigene7817_All, Unigene7993_All, Unigene8008_All, Unigene8152_All, Unigene8217_All, Unigene8228_All, Unigene8236_All, Unigene8784_All, Unigene8924_All, Unigene9107_All, Unigene9194_All, Unigene9733_All, Unigene9758_All, Unigene9971_All
115	Serotonergic synapse	CL1452.Contig1_All, CL1675.Contig2_All, CL1711.Contig3_All, CL1887.Contig1_All, CL1887.Contig2_All, CL199.Contig1_All, CL2161.Contig2_All, CL2182.Contig1_All, CL2182.Contig3_All, CL2182.Contig4_All, CL225.Contig1_All, CL225.Contig3_All, CL2432.Contig2_All, CL2590.Contig1_All, CL2703.Contig2_All, CL2930.Contig5_All, CL3043.Contig2_All, CL3137.Contig2_All, CL3286.Contig1_All, CL3362.Contig1_All, CL3362.Contig2_All, CL3554.Contig1_All, CL363.Contig2_All, CL39.Contig1_All, CL39.Contig2_All, CL39.Contig3_All, CL39.Contig4_All, CL3948.Contig1_All, CL3948.Contig3_All, CL4439.Contig2_All, CL4473.Contig1_All, CL4473.Contig2_All, CL4473.Contig3_All, CL4559.Contig2_All, CL4559.Contig3_All, CL4594.Contig1_All, CL467.Contig1_All, CL4787.Contig1_All, CL4923.Contig2_All, CL4997.Contig1_All, CL4997.Contig2_All, CL5310.Contig2_All, CL5352.Contig1_All, CL5352.Contig2_All, CL5391.Contig1_All, CL5391.Contig2_All, CL5399.Contig1_All, CL5399.Contig2_All, CL5458.Contig5_All, CL551.Contig1_All, CL551.Contig2_All, CL691.Contig1_All, CL731.Contig1_All, CL860.Contig10_All, CL860.Contig8_All, CL860.Contig9_All, CL894.Contig1_All, CL940.Contig3_All, CL940.Contig5_All, CL973.Contig1_All, Unigene10058_All, Unigene10687_All, Unigene1134_All, Unigene12110_All, Unigene12671_All, Unigene12937_All, Unigene1671_All, Unigene1743_All, Unigene17640_All, Unigene1766_All, Unigene176_All, Unigene17929_All, Unigene17987_All, Unigene18495_All, Unigene18521_All, Unigene18653_All, Unigene18654_All, Unigene18898_All, Unigene18993_All, Unigene1925_All, Unigene2029_All, Unigene2032_All, Unigene2078_All, Unigene2193_All, Unigene2196_All, Unigene2209_All, Unigene2724_All, Unigene3029_All, Unigene3033_All, Unigene3034_All, Unigene3036_All, Unigene3037_All, Unigene3042_All, Unigene3779_All, Unigene4053_All, Unigene4070_All, Unigene4112_All, Unigene4126_All, Unigene4532_All, Unigene4780_All, Unigene4884_All, Unigene5146_All, Unigene5521_All, Unigene6217_All, Unigene6265_All, Unigene69_All, Unigene7135_All, Unigene7239_All, Unigene7265_All, Unigene7298_All, Unigene812_All, Unigene8142_All, Unigene8422_All, Unigene8621_All, Unigene8747_All, Unigene8993_All, Unigene9437_All, Unigene9572_All, Unigene9733_All, Unigene9867_All

116	Adherens junction	CL102.Contig1_All, CL102.Contig2_All, CL1080.Contig2_All, CL1192.Contig1_All, CL1192.Contig2_All, CL1192.Contig3_All, CL1334.Contig2_All, CL1334.Contig3_All, CL1335.Contig1_All, CL1355.Contig1_All, CL140.Contig1_All, CL1439.Contig1_All, CL1439.Contig2_All, CL1503.Contig3_All, CL1503.Contig4_All, CL1503.Contig5_All, CL1535.Contig3_All, CL1548.Contig2_All, CL1548.Contig3_All, CL156.Contig1_All, CL156.Contig3_All, CL156.Contig4_All, CL160.Contig1_All, CL160.Contig2_All, CL160.Contig3_All, CL160.Contig4_All, CL160.Contig5_All, CL160.Contig6_All, CL160.Contig7_All, CL1723.Contig1_All, CL1723.Contig2_All, CL1723.Contig7_All, CL1803.Contig1_All, CL2034.Contig1_All, CL2034.Contig2_All, CL2170.Contig2_All, CL2170.Contig5_All, CL2339.Contig2_All, CL2368.Contig1_All, CL2375.Contig2_All, CL241.Contig1_All, CL241.Contig7_All, CL2505.Contig3_All, CL2505.Contig4_All, CL2541.Contig1_All, CL257.Contig1_All, CL257.Contig2_All, CL2658.Contig1_All, CL2658.Contig2_All, CL271.Contig2_All, CL2791.Contig2_All, CL2975.Contig2_All, CL3027.Contig3_All, CL3066.Contig2_All, CL3114.Contig2_All, CL3348.Contig1_All, CL3436.Contig1_All, CL3436.Contig4_All, CL3436.Contig9_All, CL3460.Contig2_All, CL3460.Contig3_All, CL3504.Contig1_All, CL4088.Contig2_All, CL4130.Contig1_All, CL4271.Contig1_All, CL4381.Contig1_All, CL4712.Contig2_All, CL4813.Contig1_All, CL4813.Contig2_All, CL4813.Contig3_All, CL5192.Contig1_All, CL523.Contig1_All, CL523.Contig2_All, CL5468.Contig3_All, CL622.Contig1_All, CL622.Contig2_All, CL622.Contig4_All, CL724.Contig1_All, CL95.Contig1_All, CL95.Contig2_All, Unigene10097_All, Unigene10619_All, Unigene10998_All, Unigene11082_All, Unigene11307_All, Unigene11459_All, Unigene11521_All, Unigene11597_All, Unigene11598_All, Unigene11599_All, Unigene12201_All, Unigene12340_All, Unigene12591_All, Unigene12603_All, Unigene12684_All, Unigene13092_All, Unigene13134_All, Unigene1447_All, Unigene1679_All, Unigene16840_All, Unigene16963_All, Unigene1819_All, Unigene18258_All, Unigene18423_All, Unigene18598_All, Unigene18873_All, Unigene1920_All, Unigene19424_All, Unigene2050_All, Unigene215_All, Unigene2741_All, Unigene278_All, Unigene3131_All, Unigene3135_All, Unigene4133_All, Unigene4801_All, Unigene4810_All, Unigene4998_All, Unigene5250_All, Unigene5303_All, Unigene54_All, Unigene5631_All, Unigene5727_All, Unigene5781_All, Unigene5808_All, Unigene5873_All, Unigene6014_All, Unigene6029_All, Unigene6290_All, Unigene699_All, Unigene7158_All, Unigene731_All, Unigene777_All, Unigene783_All, Unigene8184_All, Unigene8247_All, Unigene8673_All, Unigene8677_All, Unigene8972_All, Unigene9042_All, Unigene9050_All, Unigene949_All, Unigene9740_All, Unigene9785_All, Unigene9787_All, Unigene9877_All, Unigene9902_All
117	Glycosaminoglycan biosynthesis - heparan sulfate	CL1460.Contig2_All, CL1737.Contig1_All, CL1737.Contig2_All, CL1838.Contig1_All, CL2624.Contig4_All, CL3474.Contig1_All, CL3474.Contig2_All, CL424.Contig1_All, CL424.Contig2_All, CL5118.Contig2_All, Unigene12126_All, Unigene2178_All, Unigene4962_All, Unigene5903_All, Unigene6079_All, Unigene6182_All, Unigene6931_All, Unigene7375_All, Unigene8748_All
118	Prostate cancer	CL1066.Contig1_All, CL1066.Contig2_All, CL1066.Contig3_All, CL1104.Contig3_All, CL1104.Contig4_All, CL1104.Contig5_All, CL1104.Contig6_All, CL1104.Contig7_All, CL1104.Contig8_All, CL1104.Contig9_All, CL1548.Contig2_All, CL1548.Contig3_All, CL1584.Contig2_All, CL1807.Contig1_All, CL1807.Contig2_All, CL1843.Contig3_All, CL2170.Contig2_All, CL2170.Contig5_All, CL241.Contig1_All, CL241.Contig7_All, CL2411.Contig1_All, CL2456.Contig3_All, CL2505.Contig3_All, CL2505.Contig4_All, CL2552.Contig3_All, CL2711.Contig1_All, CL2711.Contig2_All, CL3078.Contig1_All, CL3373.Contig1_All, CL3373.Contig2_All, CL3618.Contig1_All, CL367.Contig1_All, CL367.Contig2_All, CL367.Contig3_All, CL3788.Contig1_All, CL3796.Contig2_All, CL3796.Contig3_All, CL3920.Contig2_All, CL4158.Contig4_All, CL4158.Contig7_All, CL4439.Contig2_All, CL4453.Contig1_All, CL450.Contig10_All, CL4787.Contig1_All, CL4969.Contig1_All, CL695.Contig16_All, CL702.Contig2_All, CL702.Contig3_All, CL95.Contig1_All, Unigene10174_All, Unigene10381_All, Unigene11000_All, Unigene11053_All, Unigene11916_All, Unigene11922_All, Unigene11949_All, Unigene12008_All, Unigene12671_All, Unigene1385_All, Unigene15263_All, Unigene15265_All, Unigene15435_All, Unigene15451_All, Unigene16499_All, Unigene1757_All, Unigene17781_All, Unigene19310_All, Unigene236_All, Unigene247_All, Unigene2742_All, Unigene2749_All, Unigene2760_All, Unigene2914_All, Unigene3689_All, Unigene54_All, Unigene5727_All, Unigene5781_All, Unigene5870_All, Unigene5896_All, Unigene6041_All, Unigene6797_All, Unigene699_All, Unigene7297_All, Unigene7299_All, Unigene7548_All, Unigene777_All, Unigene783_All, Unigene8046_All, Unigene8747_All, Unigene8784_All, Unigene8997_All, Unigene908_All, Unigene9298_All
119	Porphyrin and chlorophyll metabolism	CL1176.Contig4_All, CL2308.Contig1_All, CL2370.Contig2_All, CL2370.Contig3_All, CL2370.Contig4_All, CL2898.Contig1_All, CL2898.Contig2_All, CL2898.Contig3_All, CL3654.Contig1_All, CL3654.Contig2_All, CL4110.Contig1_All, CL4110.Contig2_All, CL4482.Contig2_All, CL490.Contig1_All, CL490.Contig2_All, CL490.Contig3_All, CL720.Contig1_All, CL720.Contig4_All, CL744.Contig1_All, CL744.Contig2_All, Unigene10617_All, Unigene1120_All, Unigene11804_All, Unigene11824_All, Unigene11996_All, Unigene12297_All, Unigene12756_All, Unigene147_All, Unigene15946_All, Unigene16575_All, Unigene18028_All, Unigene18489_All, Unigene19085_All, Unigene19677_All, Unigene2107_All, Unigene2312_All, Unigene2660_All, Unigene3012_All, Unigene3125_All, Unigene3132_All, Unigene3180_All, Unigene3701_All, Unigene3771_All, Unigene4342_All, Unigene4564_All, Unigene4675_All, Unigene526_All, Unigene5429_All, Unigene5637_All, Unigene5751_All, Unigene6129_All, Unigene6650_All, Unigene6673_All, Unigene671_All, Unigene8049_All, Unigene8218_All, Unigene9227_All
120	mTOR signaling pathway	CL1066.Contig1_All, CL1066.Contig2_All, CL1066.Contig3_All, CL2083.Contig1_All, CL2187.Contig2_All, CL2207.Contig2_All, CL3093.Contig1_All, CL3112.Contig3_All, CL3263.Contig1_All, CL367.Contig1_All, CL367.Contig2_All, CL367.Contig3_All, CL3796.Contig2_All, CL3796.Contig3_All, CL4529.Contig2_All, CL5158.Contig1_All, CL5158.Contig2_All, CL524.Contig10_All, CL524.Contig11_All, CL524.Contig12_All, CL524.Contig13_All, CL524.Contig14_All, CL524.Contig15_All, CL524.Contig16_All, CL524.Contig17_All, CL524.Contig18_All, CL524.Contig1_All, CL524.Contig3_All, CL524.Contig4_All, CL524.Contig5_All, CL524.Contig6_All, CL524.Contig9_All, CL557.Contig1_All, CL695.Contig16_All, Unigene10497_All, Unigene10676_All, Unigene10677_All, Unigene10875_All, Unigene1155_All, Unigene11811_All, Unigene13135_All, Unigene14175_All, Unigene18383_All, Unigene2742_All, Unigene2749_All, Unigene2956_All, Unigene6007_All, Unigene7745_All, Unigene8747_All

121	Non-small cell lung cancer	CL1066.Contig1_All, CL1066.Contig2_All, CL1066.Contig3_All, CL1091.Contig14_All, CL1104.Contig1_All, CL1104.Contig2_All, CL1104.Contig3_All, CL1104.Contig4_All, CL1104.Contig5_All, CL1104.Contig6_All, CL1104.Contig7_All, CL1104.Contig8_All, CL1104.Contig9_All, CL1548.Contig2_All, CL1548.Contig3_All, CL1741.Contig1_All, CL1807.Contig1_All, CL1807.Contig2_All, CL1960.Contig1_All, CL1960.Contig2_All, CL2182.Contig1_All, CL2182.Contig3_All, CL2182.Contig4_All, CL3373.Contig1_All, CL3373.Contig2_All, CL367.Contig1_All, CL367.Contig2_All, CL367.Contig3_All, CL39.Contig1_All, CL39.Contig2_All, CL39.Contig3_All, CL39.Contig4_All, CL3979.Contig1_All, CL3979.Contig2_All, CL4158.Contig4_All, CL4158.Contig5_All, CL4158.Contig7_All, CL4439.Contig2_All, CL4787.Contig1_All, CL5204.Contig1_All, CL5204.Contig2_All, CL5391.Contig1_All, CL5391.Contig2_All, CL695.Contig16_All, CL880.Contig3_All, CL940.Contig3_All, CL940.Contig5_All, Unigene10174_All, Unigene12099_All, Unigene12623_All, Unigene12671_All, Unigene18898_All, Unigene2374_All, Unigene2742_All, Unigene2749_All, Unigene699_All, Unigene740_All, Unigene8046_All, Unigene8747_All
122	Polyketide sugar unit biosynthesis	CL1371.Contig2_All, Unigene13457_All, Unigene5837_All
123	Amphetamine addiction	CL1066.Contig1_All, CL1066.Contig2_All, CL1286.Contig2_All, CL1452.Contig1_All, CL1563.Contig2_All, CL1823.Contig8_All, CL2131.Contig4_All, CL2131.Contig5_All, CL2131.Contig6_All, CL2131.Contig8_All, CL2182.Contig1_All, CL2182.Contig3_All, CL2182.Contig4_All, CL244.Contig2_All, CL2468.Contig1_All, CL2468.Contig2_All, CL3286.Contig1_All, CL3667.Contig1_All, CL3667.Contig2_All, CL3667.Contig3_All, CL3689.Contig2_All, CL39.Contig1_All, CL39.Contig2_All, CL39.Contig3_All, CL39.Contig4_All, CL3920.Contig2_All, CL4002.Contig1_All, CL42.Contig2_All, CL4395.Contig1_All, CL44.Contig10_All, CL44.Contig12_All, CL44.Contig13_All, CL44.Contig16_All, CL44.Contig17_All, CL44.Contig18_All, CL44.Contig19_All, CL44.Contig1_All, CL44.Contig22_All, CL44.Contig23_All, CL44.Contig2_All, CL44.Contig3_All, CL44.Contig4_All, CL44.Contig5_All, CL44.Contig6_All, CL44.Contig8_All, CL44.Contig9_All, CL4509.Contig1_All, CL4509.Contig2_All, CL4521.Contig1_All, CL4521.Contig2_All, CL4521.Contig3_All, CL4521.Contig5_All, CL4594.Contig1_All, CL4736.Contig1_All, CL4736.Contig2_All, CL5064.Contig2_All, CL5391.Contig1_All, CL5391.Contig2_All, CL741.Contig2_All, CL772.Contig1_All, CL772.Contig2_All, CL870.Contig4_All, CL870.Contig8_All, CL870.Contig9_All, CL940.Contig3_All, CL940.Contig5_All, Unigene10006_All, Unigene10058_All, Unigene10078_All, Unigene10574_All, Unigene11054_All, Unigene1168_All, Unigene12315_All, Unigene12719_All, Unigene13853_All, Unigene1417_All, Unigene1479_All, Unigene1512_All, Unigene1663_All, Unigene16769_All, Unigene18898_All, Unigene19148_All, Unigene1941_All, Unigene2029_All, Unigene2193_All, Unigene2233_All, Unigene2760_All, Unigene3779_All, Unigene3812_All, Unigene3996_All, Unigene4013_All, Unigene4022_All, Unigene4112_All, Unigene4116_All, Unigene4532_All, Unigene4935_All, Unigene4970_All, Unigene5179_All, Unigene5675_All, Unigene5869_All, Unigene6005_All, Unigene661_All, Unigene6655_All, Unigene7298_All, Unigene7702_All, Unigene7704_All, Unigene7759_All, Unigene8621_All, Unigene9733_All, Unigene9904_All
124	Glycolysis / Gluconeogenesis	CL1879.Contig2_All, CL1879.Contig3_All, CL1879.Contig4_All, CL2017.Contig4_All, CL2017.Contig5_All, CL2294.Contig4_All, CL2309.Contig2_All, CL2309.Contig3_All, CL2430.Contig3_All, CL2595.Contig1_All, CL2694.Contig1_All, CL2694.Contig3_All, CL4130.Contig1_All, CL4130.Contig2_All, CL4144.Contig3_All, CL4144.Contig4_All, CL4358.Contig1_All, CL4451.Contig1_All, CL4451.Contig2_All, CL4753.Contig1_All, CL898.Contig1_All, CL898.Contig2_All, Unigene10029_All, Unigene10098_All, Unigene10111_All, Unigene10282_All, Unigene10299_All, Unigene10526_All, Unigene10686_All, Unigene10908_All, Unigene11061_All, Unigene11062_All, Unigene11186_All, Unigene11187_All, Unigene11188_All, Unigene11380_All, Unigene11560_All, Unigene11561_All, Unigene11782_All, Unigene11788_All, Unigene11826_All, Unigene11830_All, Unigene11894_All, Unigene11930_All, Unigene1197_All, Unigene12066_All, Unigene12140_All, Unigene12300_All, Unigene12399_All, Unigene12459_All, Unigene12491_All, Unigene12509_All, Unigene12589_All, Unigene12643_All, Unigene12732_All, Unigene12801_All, Unigene13375_All, Unigene13410_All, Unigene13594_All, Unigene13727_All, Unigene13773_All, Unigene15270_All, Unigene1538_All, Unigene15851_All, Unigene16206_All, Unigene16290_All, Unigene16549_All, Unigene16591_All, Unigene16824_All, Unigene17053_All, Unigene17282_All, Unigene17410_All, Unigene17527_All, Unigene17838_All, Unigene17873_All, Unigene17939_All, Unigene18003_All, Unigene18004_All, Unigene18088_All, Unigene18101_All, Unigene18194_All, Unigene18248_All, Unigene18312_All, Unigene18656_All, Unigene18663_All, Unigene18666_All, Unigene186_All, Unigene18842_All, Unigene19024_All, Unigene19038_All, Unigene19153_All, Unigene19184_All, Unigene19340_All, Unigene19366_All, Unigene20152_All, Unigene20815_All, Unigene2322_All, Unigene236_All, Unigene2496_All, Unigene2928_All, Unigene3105_All, Unigene3450_All, Unigene4267_All, Unigene4626_All, Unigene5053_All, Unigene5180_All, Unigene5338_All, Unigene627_All, Unigene6360_All, Unigene6526_All, Unigene6618_All, Unigene6954_All, Unigene7735_All, Unigene8037_All, Unigene8347_All, Unigene8563_All, Unigene923_All
125	Ascorbate and aldarate metabolism	CL1176.Contig4_All, CL2145.Contig2_All, CL2308.Contig1_All, CL2309.Contig2_All, CL2309.Contig3_All, CL2370.Contig2_All, CL2370.Contig3_All, CL2370.Contig4_All, CL2694.Contig1_All, CL2694.Contig3_All, CL3654.Contig1_All, CL3654.Contig2_All, CL4110.Contig1_All, CL4110.Contig2_All, CL4144.Contig3_All, CL4144.Contig4_All, CL4451.Contig1_All, CL4451.Contig2_All, CL4753.Contig1_All, CL490.Contig1_All, CL490.Contig2_All, CL490.Contig3_All, CL720.Contig1_All, CL720.Contig4_All, CL744.Contig1_All, CL744.Contig2_All, Unigene10282_All, Unigene10299_All, Unigene10526_All, Unigene10686_All, Unigene10908_All, Unigene1120_All, Unigene1197_All, Unigene12297_All, Unigene12955_All, Unigene13410_All, Unigene13773_All, Unigene147_All, Unigene15270_All, Unigene15946_All, Unigene16206_All, Unigene17527_All, Unigene18028_All, Unigene18101_All, Unigene18489_All, Unigene186_All, Unigene19038_All, Unigene2107_All, Unigene2312_All, Unigene2660_All, Unigene3012_All, Unigene3125_All, Unigene3132_All, Unigene3450_All, Unigene3701_All, Unigene3771_All, Unigene4342_All, Unigene4564_All, Unigene4675_All, Unigene5053_All, Unigene526_All, Unigene5637_All, Unigene5751_All, Unigene6129_All, Unigene6360_All, Unigene6650_All, Unigene6673_All, Unigene8049_All, Unigene8218_All, Unigene9227_All
126	Vitamin B6 metabolism	CL3364.Contig2_All, Unigene10403_All, Unigene10404_All, Unigene12739_All, Unigene13154_All

127	Pertussis	CL1085.Contig3_All, CL1286.Contig2_All, CL1823.Contig8_All, CL2131.Contig4_All, CL2131.Contig5_All, CL2131.Contig6_All, CL2131.Contig8_All, CL2468.Contig1_All, CL2468.Contig2_All, CL326.Contig1_All, CL3667.Contig1_All, CL3667.Contig2_All, CL3667.Contig3_All, CL4395.Contig1_All, CL4509.Contig1_All, CL4509.Contig2_All, CL4736.Contig1_All, CL4736.Contig2_All, CL4966.Contig1_All, CL5115.Contig1_All, CL772.Contig1_All, CL772.Contig2_All, Unigene10006_All, Unigene10574_All, Unigene10775_All, Unigene11052_All, Unigene11054_All, Unigene11307_All, Unigene12315_All, Unigene12719_All, Unigene1479_All, Unigene16769_All, Unigene1757_All, Unigene20095_All, Unigene220_All, Unigene3945_All, Unigene3996_All, Unigene4013_All, Unigene4207_All, Unigene5521_All, Unigene5675_All, Unigene5808_All, Unigene6217_All, Unigene8246_All, Unigene9050_All, Unigene9877_All
128	Glycosphingolipid biosynthesis - ganglio series	CL3965.Contig1_All, CL3965.Contig2_All, CL3965.Contig4_All, CL5403.Contig2_All, Unigene17953_All, Unigene5646_All, Unigene9260_All, Unigene9314_All
129	Drug metabolism - other enzymes	CL1156.Contig3_All, CL1176.Contig4_All, CL1305.Contig1_All, CL1531.Contig1_All, CL1531.Contig2_All, CL1531.Contig3_All, CL1711.Contig1_All, CL1711.Contig2_All, CL2220.Contig1_All, CL2220.Contig2_All, CL2272.Contig1_All, CL2308.Contig1_All, CL2370.Contig2_All, CL2370.Contig3_All, CL2370.Contig4_All, CL2532.Contig1_All, CL2533.Contig3_All, CL258.Contig1_All, CL258.Contig2_All, CL2707.Contig1_All, CL3511.Contig2_All, CL3614.Contig1_All, CL3614.Contig2_All, CL3654.Contig1_All, CL3654.Contig2_All, CL3878.Contig2_All, CL3994.Contig1_All, CL4030.Contig3_All, CL4030.Contig4_All, CL4110.Contig1_All, CL4110.Contig2_All, CL4195.Contig2_All, CL4527.Contig1_All, CL4527.Contig2_All, CL485.Contig1_All, CL485.Contig2_All, CL4850.Contig1_All, CL4850.Contig3_All, CL490.Contig1_All, CL490.Contig2_All, CL490.Contig3_All, CL5266.Contig1_All, CL5266.Contig2_All, CL720.Contig1_All, CL720.Contig4_All, CL731.Contig1_All, CL744.Contig1_All, CL744.Contig2_All, CL900.Contig3_All, Unigene10164_All, Unigene10742_All, Unigene11026_All, Unigene11027_All, Unigene1120_All, Unigene11671_All, Unigene12297_All, Unigene12396_All, Unigene1254_All, Unigene14002_All, Unigene147_All, Unigene15946_All, Unigene16322_All, Unigene18028_All, Unigene18489_All, Unigene18597_All, Unigene19987_All, Unigene2107_All, Unigene2150_All, Unigene2260_All, Unigene2312_All, Unigene2660_All, Unigene2675_All, Unigene2686_All, Unigene3012_All, Unigene3125_All, Unigene3132_All, Unigene3701_All, Unigene3706_All, Unigene3707_All, Unigene3771_All, Unigene4096_All, Unigene432_All, Unigene4342_All, Unigene4564_All, Unigene4675_All, Unigene5060_All, Unigene5216_All, Unigene526_All, Unigene5429_All, Unigene5600_All, Unigene5637_All, Unigene5751_All, Unigene6080_All, Unigene6129_All, Unigene6650_All, Unigene6673_All, Unigene7023_All, Unigene7663_All, Unigene8049_All, Unigene8218_All, Unigene8370_All, Unigene8643_All, Unigene9227_All, Unigene9644_All
130	Nicotinate and nicotinamide metabolism	CL1258.Contig1_All, CL1258.Contig2_All, CL1388.Contig1_All, CL3028.Contig1_All, CL3040.Contig1_All, CL3228.Contig1_All, CL3576.Contig1_All, CL4346.Contig1_All, CL52.Contig11_All, CL52.Contig15_All, CL52.Contig24_All, CL52.Contig2_All, CL52.Contig5_All, Unigene12645_All, Unigene12887_All, Unigene20289_All, Unigene3890_All, Unigene7822_All, Unigene8726_All, Unigene8970_All, Unigene8971_All
131	D-Arginine and D-ornithine metabolism	CL3315.Contig3_All, CL3315.Contig4_All, CL348.Contig1_All, CL348.Contig2_All, CL580.Contig2_All, CL584.Contig10_All, CL584.Contig15_All, CL584.Contig17_All, CL584.Contig1_All, CL584.Contig3_All, CL584.Contig4_All, CL584.Contig6_All, CL584.Contig7_All, Unigene3482_All
132	Long-term potentiation	CL1286.Contig2_All, CL1452.Contig1_All, CL147.Contig6_All, CL1563.Contig2_All, CL1823.Contig8_All, CL2131.Contig4_All, CL2131.Contig5_All, CL2131.Contig6_All, CL2131.Contig8_All, CL2170.Contig2_All, CL2170.Contig5_All, CL2182.Contig1_All, CL2182.Contig3_All, CL2182.Contig4_All, CL244.Contig2_All, CL2468.Contig1_All, CL2468.Contig2_All, CL2930.Contig5_All, CL296.Contig16_All, CL3263.Contig1_All, CL3667.Contig1_All, CL3667.Contig2_All, CL3667.Contig3_All, CL3689.Contig2_All, CL39.Contig1_All, CL39.Contig2_All, CL39.Contig3_All, CL39.Contig4_All, CL4002.Contig1_All, CL4005.Contig2_All, CL4005.Contig3_All, CL4262.Contig2_All, CL4395.Contig1_All, CL44.Contig10_All, CL44.Contig12_All, CL44.Contig13_All, CL44.Contig16_All, CL44.Contig17_All, CL44.Contig18_All, CL44.Contig19_All, CL44.Contig1_All, CL44.Contig22_All, CL44.Contig23_All, CL44.Contig2_All, CL44.Contig3_All, CL44.Contig4_All, CL44.Contig5_All, CL44.Contig6_All, CL44.Contig8_All, CL44.Contig9_All, CL4439.Contig2_All, CL4509.Contig1_All, CL4509.Contig2_All, CL4521.Contig1_All, CL4521.Contig2_All, CL4521.Contig3_All, CL4521.Contig5_All, CL4787.Contig1_All, CL5064.Contig2_All, CL5098.Contig2_All, CL5391.Contig1_All, CL5391.Contig2_All, CL5399.Contig1_All, CL5399.Contig2_All, CL691.Contig1_All, CL741.Contig2_All, CL772.Contig1_All, CL772.Contig2_All, CL870.Contig4_All, CL870.Contig8_All, CL870.Contig9_All, CL940.Contig3_All, CL940.Contig5_All, Unigene10006_All, Unigene10574_All, Unigene11054_All, Unigene1168_All, Unigene12315_All, Unigene12671_All, Unigene12719_All, Unigene13853_All, Unigene1417_All, Unigene1512_All, Unigene16769_All, Unigene18898_All, Unigene18993_All, Unigene19148_All, Unigene19288_All, Unigene1941_All, Unigene2209_All, Unigene2233_All, Unigene3779_All, Unigene3996_All, Unigene4013_All, Unigene4022_All, Unigene4053_All, Unigene4070_All, Unigene4112_All, Unigene4935_All, Unigene4970_All, Unigene54_All, Unigene5675_All, Unigene5781_All, Unigene6242_All, Unigene661_All, Unigene6655_All, Unigene7704_All, Unigene7759_All, Unigene807_All, Unigene8422_All, Unigene8747_All, Unigene9437_All, Unigene9733_All, Unigene9904_All
133	Homologous recombination	CL1795.Contig3_All, CL2300.Contig1_All, CL2372.Contig1_All, CL2372.Contig2_All, CL2372.Contig3_All, CL4073.Contig1_All, CL4073.Contig2_All, CL91.Contig1_All, Unigene10181_All, Unigene10182_All, Unigene10838_All, Unigene11936_All, Unigene3089_All, Unigene4102_All, Unigene4187_All, Unigene4238_All, Unigene8036_All, Unigene8228_All, Unigene9194_All

134	Dorso-ventral axis formation	CL1099.Contig3_All, CL1193.Contig1_All, CL1193.Contig2_All, CL1193.Contig3_All, CL1548.Contig2_All, CL1548.Contig3_All, CL1807.Contig1_All, CL1807.Contig2_All, CL2375.Contig2_All, CL2563.Contig2_All, CL2824.Contig3_All, CL320.Contig1_All, CL320.Contig2_All, CL3206.Contig2_All, CL3223.Contig5_All, CL4439.Contig2_All, CL4622.Contig2_All, CL4787.Contig1_All, CL4994.Contig1_All, CL5296.Contig1_All, CL5432.Contig1_All, CL581.Contig7_All, CL581.Contig8_All, CL581.Contig9_All, Unigene10174_All, Unigene1040_All, Unigene11116_All, Unigene11119_All, Unigene11197_All, Unigene11521_All, Unigene11874_All, Unigene12034_All, Unigene12233_All, Unigene12359_All, Unigene1237_All, Unigene12523_All, Unigene12655_All, Unigene12661_All, Unigene12671_All, Unigene168_All, Unigene18046_All, Unigene18054_All, Unigene18262_All, Unigene18319_All, Unigene2106_All, Unigene2700_All, Unigene3080_All, Unigene32_All, Unigene3314_All, Unigene3423_All, Unigene4758_All, Unigene5064_All, Unigene5237_All, Unigene699_All, Unigene7093_All, Unigene7519_All, Unigene8046_All, Unigene8294_All, Unigene8747_All, Unigene8986_All, Unigene9065_All, Unigene9097_All, Unigene9527_All
135	Vasopressin-regulated water reabsorption	CL1452.Contig1_All, CL1920.Contig1_All, CL1920.Contig2_All, CL2382.Contig4_All, CL2437.Contig1_All, CL2437.Contig2_All, CL3282.Contig1_All, CL3282.Contig2_All, CL3282.Contig3_All, CL3920.Contig2_All, CL440.Contig3_All, CL440.Contig5_All, CL440.Contig6_All, CL4661.Contig1_All, CL4661.Contig2_All, CL940.Contig3_All, CL940.Contig5_All, CL969.Contig2_All, Unigene10058_All, Unigene1028_All, Unigene11033_All, Unigene11086_All, Unigene11935_All, Unigene11973_All, Unigene12330_All, Unigene12343_All, Unigene13618_All, Unigene13732_All, Unigene14005_All, Unigene16349_All, Unigene17864_All, Unigene18950_All, Unigene20226_All, Unigene2029_All, Unigene2058_All, Unigene2760_All, Unigene3094_All, Unigene3779_All, Unigene4112_All, Unigene4532_All, Unigene5688_All, Unigene6532_All, Unigene8002_All, Unigene9039_All, Unigene9733_All
136	NOD-like receptor signaling pathway	CL268.Contig2_All, CL3671.Contig1_All, CL478.Contig2_All, CL478.Contig3_All, CL478.Contig4_All, CL478.Contig5_All, CL4966.Contig1_All, CL5115.Contig1_All, CL702.Contig2_All, CL702.Contig3_All, CL709.Contig1_All, Unigene11000_All, Unigene11053_All, Unigene15263_All, Unigene15265_All, Unigene16499_All, Unigene1757_All, Unigene17781_All, Unigene220_All, Unigene245_All, Unigene3131_All, Unigene5973_All, Unigene5974_All, Unigene5995_All, Unigene6797_All, Unigene7220_All, Unigene7737_All, Unigene8156_All
137	Fc epsilon RI signaling pathway	CL1066.Contig1_All, CL1066.Contig2_All, CL1066.Contig3_All, CL1535.Contig3_All, CL1807.Contig1_All, CL1807.Contig2_All, CL1954.Contig1_All, CL1954.Contig2_All, CL1966.Contig2_All, CL199.Contig1_All, CL2129.Contig4_All, CL2182.Contig1_All, CL2182.Contig3_All, CL2182.Contig4_All, CL2432.Contig2_All, CL257.Contig1_All, CL257.Contig2_All, CL3047.Contig1_All, CL3047.Contig2_All, CL367.Contig1_All, CL367.Contig2_All, CL367.Contig3_All, CL39.Contig1_All, CL39.Contig2_All, CL39.Contig3_All, CL39.Contig4_All, CL3979.Contig1_All, CL3979.Contig2_All, CL4439.Contig2_All, CL4787.Contig1_All, CL4815.Contig1_All, CL4815.Contig2_All, CL4855.Contig1_All, CL4855.Contig2_All, CL4966.Contig1_All, CL5115.Contig1_All, CL5310.Contig2_All, CL5391.Contig1_All, CL5391.Contig2_All, CL695.Contig16_All, CL860.Contig10_All, CL860.Contig8_All, CL860.Contig9_All, CL940.Contig3_All, CL940.Contig5_All, Unigene10174_All, Unigene12139_All, Unigene12671_All, Unigene1326_All, Unigene18898_All, Unigene220_All, Unigene2749_All, Unigene3029_All, Unigene3033_All, Unigene3034_All, Unigene3036_All, Unigene3037_All, Unigene3042_All, Unigene6977_All, Unigene8046_All, Unigene8056_All

138	HTLV-I infection	CL1012.Contig4_All, CL1012.Contig5_All, CL1012.Contig6_All, CL1012.Contig7_All, CL1012.Contig9_All, CL1049.Contig4_All, CL1066.Contig1_All, CL1066.Contig2_All, CL1066.Contig3_All, CL1070.Contig6_All, CL1070.Contig7_All, CL131.Contig1_All, CL131.Contig2_All, CL1390.Contig4_All, CL1390.Contig5_All, CL1395.Contig2_All, CL1439.Contig1_All, CL1439.Contig2_All, CL1452.Contig1_All, CL1581.Contig1_All, CL1581.Contig2_All, CL1756.Contig1_All, CL1766.Contig1_All, CL1776.Contig1_All, CL1831.Contig1_All, CL1831.Contig2_All, CL1843.Contig3_All, CL1966.Contig2_All, CL1991.Contig2_All, CL1991.Contig3_All, CL1991.Contig4_All, CL2038.Contig1_All, CL2038.Contig7_All, CL2144.Contig1_All, CL2144.Contig2_All, CL2170.Contig2_All, CL2170.Contig5_All, CL2208.Contig1_All, CL2416.Contig1_All, CL2608.Contig1_All, CL2749.Contig2_All, CL2788.Contig2_All, CL2809.Contig2_All, CL2809.Contig3_All, CL2809.Contig8_All, CL2978.Contig2_All, CL2978.Contig3_All, CL3373.Contig1_All, CL3373.Contig2_All, CL3572.Contig1_All, CL358.Contig1_All, CL3632.Contig2_All, CL3632.Contig3_All, CL366.Contig1_All, CL367.Contig1_All, CL367.Contig2_All, CL367.Contig3_All, CL3695.Contig1_All, CL3695.Contig2_All, CL3920.Contig1_All, CL3920.Contig2_All, CL3920.Contig3_All, CL400.Contig1_All, CL400.Contig2_All, CL4005.Contig2_All, CL4005.Contig3_All, CL4105.Contig2_All, CL4190.Contig1_All, CL4247.Contig2_All, CL4247.Contig3_All, CL4262.Contig2_All, CL4271.Contig1_All, CL4278.Contig2_All, CL440.Contig3_All, CL440.Contig5_All, CL440.Contig6_All, CL4439.Contig2_All, CL4510.Contig1_All, CL4521.Contig1_All, CL4521.Contig2_All, CL4521.Contig3_All, CL4521.Contig5_All, CL4555.Contig1_All, CL4567.Contig2_All, CL4633.Contig1_All, CL4633.Contig2_All, CL4657.Contig2_All, CL4736.Contig1_All, CL4736.Contig2_All, CL475.Contig4_All, CL4874.Contig1_All, CL4887.Contig2_All, CL4896.Contig1_All, CL493.Contig2_All, CL5064.Contig2_All, CL5111.Contig1_All, CL5115.Contig1_All, CL5206.Contig1_All, CL5218.Contig1_All, CL5218.Contig2_All, CL5263.Contig1_All, CL5263.Contig2_All, CL5427.Contig2_All, CL695.Contig16_All, CL874.Contig10_All, CL874.Contig8_All, CL91.Contig1_All, CL940.Contig3_All, CL940.Contig5_All, Unigene10252_All, Unigene10253_All, Unigene10763_All, Unigene10817_All, Unigene11321_All, Unigene11357_All, Unigene11422_All, Unigene1181_All, Unigene11845_All, Unigene11860_All, Unigene11922_All, Unigene12099_All, Unigene1229_All, Unigene12361_All, Unigene12398_All, Unigene12469_All, Unigene12660_All, Unigene12664_All, Unigene12671_All, Unigene12713_All, Unigene12722_All, Unigene13853_All, Unigene1459_All, Unigene1479_All, Unigene15790_All, Unigene16098_All, Unigene17292_All, Unigene17478_All, Unigene1757_All, Unigene17674_All, Unigene17675_All, Unigene18118_All, Unigene18119_All, Unigene18127_All, Unigene1823_All, Unigene18284_All, Unigene18584_All, Unigene19116_All, Unigene1944_All, Unigene20260_All, Unigene20279_All, Unigene2029_All, Unigene20308_All, Unigene2050_All, Unigene220_All, Unigene22_All, Unigene230_All, Unigene236_All, Unigene2374_All, Unigene247_All, Unigene2749_All, Unigene3168_All, Unigene3171_All, Unigene3632_All, Unigene3779_All, Unigene3830_All, Unigene3975_All, Unigene4102_All, Unigene4112_All, Unigene4731_All, Unigene47_All, Unigene4801_All, Unigene5175_All, Unigene5196_All, Unigene54_All, Unigene5727_All, Unigene5781_All, Unigene5897_All, Unigene6048_All, Unigene6101_All, Unigene695_All, Unigene6977_All, Unigene700_All, Unigene7163_All, Unigene7297_All, Unigene766_All, Unigene7702_All, Unigene7747_All, Unigene783_All, Unigene7886_All, Unigene8014_All, Unigene8174_All, Unigene8228_All, Unigene8605_All, Unigene8784_All, Unigene8861_All, Unigene8972_All, Unigene9125_All, Unigene9237_All, Unigene93_All, Unigene9733_All, Unigene9989_All
139	Arginine and proline metabolism	CL1707.Contig1_All, CL1780.Contig1_All, CL1780.Contig2_All, CL1868.Contig1_All, CL1868.Contig2_All, CL1868.Contig4_All, CL1892.Contig1_All, CL1892.Contig2_All, CL1892.Contig3_All, CL1892.Contig5_All, CL2309.Contig2_All, CL2309.Contig3_All, CL2694.Contig1_All, CL2694.Contig3_All, CL3118.Contig1_All, CL3118.Contig2_All, CL313.Contig2_All, CL3235.Contig1_All, CL3235.Contig2_All, CL3283.Contig1_All, CL3315.Contig3_All, CL3315.Contig4_All, CL348.Contig1_All, CL348.Contig2_All, CL3643.Contig2_All, CL3643.Contig6_All, CL4144.Contig3_All, CL4144.Contig4_All, CL4446.Contig1_All, CL4446.Contig2_All, CL4451.Contig1_All, CL4451.Contig2_All, CL4753.Contig1_All, CL4873.Contig2_All, CL5083.Contig2_All, CL5197.Contig2_All, CL5288.Contig2_All, CL5320.Contig2_All, CL567.Contig4_All, CL580.Contig2_All, CL584.Contig10_All, CL584.Contig15_All, CL584.Contig17_All, CL584.Contig1_All, CL584.Contig3_All, CL584.Contig4_All, CL584.Contig6_All, CL584.Contig7_All, Unigene10278_All, Unigene10282_All, Unigene10299_All, Unigene10412_All, Unigene10526_All, Unigene10686_All, Unigene10908_All, Unigene10909_All, Unigene10910_All, Unigene11012_All, Unigene11013_All, Unigene11336_All, Unigene11575_All, Unigene1197_All, Unigene12216_All, Unigene12325_All, Unigene12470_All, Unigene12475_All, Unigene12600_All, Unigene12958_All, Unigene13121_All, Unigene13245_All, Unigene1327_All, Unigene13410_All, Unigene13773_All, Unigene13925_All, Unigene15270_All, Unigene16206_All, Unigene1623_All, Unigene17527_All, Unigene18101_All, Unigene18234_All, Unigene18440_All, Unigene186_All, Unigene19038_All, Unigene261_All, Unigene3134_All, Unigene3324_All, Unigene3450_All, Unigene3482_All, Unigene3655_All, Unigene5053_All, Unigene5433_All, Unigene6134_All, Unigene6358_All, Unigene6360_All, Unigene6708_All, Unigene6731_All, Unigene703_All, Unigene9060_All, Unigene9215_All, Unigene9262_All, Unigene9708_All
140	RNA polymerase	CL1356.Contig1_All, CL1356.Contig2_All, CL1411.Contig1_All, CL1411.Contig8_All, CL1683.Contig2_All, CL1683.Contig7_All, CL322.Contig4_All, CL322.Contig5_All, CL322.Contig6_All, CL3251.Contig1_All, CL3251.Contig2_All, CL3420.Contig1_All, CL3635.Contig3_All, CL3657.Contig3_All, CL3664.Contig1_All, CL3664.Contig2_All, CL3792.Contig3_All, CL5004.Contig1_All, CL5461.Contig2_All, CL598.Contig1_All, CL598.Contig2_All, CL63.Contig10_All, CL63.Contig9_All, Unigene11623_All, Unigene12017_All, Unigene1205_All, Unigene12060_All, Unigene12080_All, Unigene12096_All, Unigene12117_All, Unigene12479_All, Unigene12488_All, Unigene1278_All, Unigene13002_All, Unigene13284_All, Unigene15827_All, Unigene18801_All, Unigene19657_All, Unigene2037_All, Unigene2131_All, Unigene24_All, Unigene2513_All, Unigene3081_All, Unigene3288_All, Unigene3390_All, Unigene33_All, Unigene3419_All, Unigene3659_All, Unigene4251_All, Unigene5745_All, Unigene7192_All, Unigene7296_All, Unigene7750_All, Unigene7993_All, Unigene8008_All, Unigene8228_All, Unigene8236_All, Unigene9107_All, Unigene9758_All
141	Glycosphingolipid biosynthesis - globo series	CL3965.Contig1_All, CL3965.Contig2_All, CL3965.Contig4_All, Unigene17953_All, Unigene5646_All, Unigene8139_All, Unigene9260_All, Unigene9314_All

142	Circadian rhythm - mammal	CL2599.Contig1_All, CL2599.Contig2_All, CL2838.Contig1_All, CL2838.Contig2_All, CL2838.Contig4_All, CL3787.Contig1_All, CL3787.Contig2_All, CL4514.Contig2_All, Unigene10876_All, Unigene11040_All, Unigene11411_All, Unigene12740_All, Unigene12903_All, Unigene3715_All, Unigene4765_All, Unigene4975_All, Unigene7671_All, Unigene798_All, Unigene9006_All
143	Melanoma	CL1066.Contig1_All, CL1066.Contig2_All, CL1066.Contig3_All, CL1548.Contig2_All, CL1548.Contig3_All, CL241.Contig1_All, CL241.Contig7_All, CL2505.Contig3_All, CL2505.Contig4_All, CL2552.Contig3_All, CL2658.Contig1_All, CL2658.Contig2_All, CL3373.Contig1_All, CL3373.Contig2_All, CL3567.Contig1_All, CL3567.Contig2_All, CL3567.Contig3_All, CL367.Contig1_All, CL367.Contig2_All, CL367.Contig3_All, CL4381.Contig1_All, CL4439.Contig2_All, CL4787.Contig1_All, CL695.Contig16_All, CL95.Contig1_All, Unigene12099_All, Unigene12671_All, Unigene1679_All, Unigene2374_All, Unigene2749_All, Unigene699_All, Unigene8747_All, Unigene90_All, Unigene9740_All
144	Asthma	Unigene833_All
145	Nicotine addiction	CL1069.Contig1_All, CL1069.Contig2_All, CL1069.Contig3_All, CL1069.Contig4_All, CL1069.Contig5_All, CL1069.Contig6_All, CL1069.Contig7_All, CL1069.Contig8_All, CL2161.Contig1_All, CL2161.Contig2_All, CL225.Contig1_All, CL225.Contig3_All, CL2703.Contig2_All, CL2735.Contig1_All, CL3166.Contig1_All, CL3507.Contig1_All, CL5105.Contig1_All, CL5105.Contig2_All, CL677.Contig2_All, CL679.Contig1_All, CL679.Contig2_All, CL847.Contig3_All, CL870.Contig4_All, CL870.Contig8_All, CL870.Contig9_All, CL894.Contig1_All, CL973.Contig1_All, Unigene1044_All, Unigene10931_All, Unigene11615_All, Unigene12433_All, Unigene12535_All, Unigene13612_All, Unigene1417_All, Unigene16890_All, Unigene1766_All, Unigene17896_All, Unigene18199_All, Unigene19148_All, Unigene2032_All, Unigene3357_All, Unigene661_All, Unigene68_All, Unigene7042_All, Unigene8071_All, Unigene8170_All, Unigene8993_All, Unigene9167_All
146	Glycosaminoglycan biosynthesis - chondroitin sulfate	CL1070.Contig6_All, CL1070.Contig7_All, CL424.Contig1_All, CL424.Contig2_All, CL5118.Contig2_All, CL5417.Contig1_All, CL5417.Contig2_All, Unigene12126_All, Unigene5634_All, Unigene5903_All, Unigene6931_All, Unigene7375_All
147	Malaria	CL2658.Contig1_All, CL2658.Contig2_All, CL3236.Contig1_All, CL4381.Contig1_All, CL4535.Contig1_All, CL4597.Contig2_All, Unigene10373_All, Unigene10999_All, Unigene11371_All, Unigene13362_All, Unigene1679_All, Unigene19844_All, Unigene20095_All, Unigene3945_All, Unigene6791_All, Unigene8217_All, Unigene8915_All
148	Valine, leucine and isoleucine biosynthesis	CL1404.Contig2_All, CL2294.Contig4_All, Unigene11062_All, Unigene12491_All, Unigene12589_All, Unigene12801_All, Unigene12959_All, Unigene13041_All, Unigene17053_All, Unigene17410_All, Unigene18312_All, Unigene18842_All, Unigene5691_All, Unigene6618_All
149	Cell cycle	CL131.Contig1_All, CL131.Contig2_All, CL1439.Contig1_All, CL1439.Contig2_All, CL1590.Contig4_All, CL1660.Contig10_All, CL1660.Contig15_All, CL1660.Contig16_All, CL1660.Contig1_All, CL1660.Contig3_All, CL1660.Contig5_All, CL1660.Contig6_All, CL1660.Contig7_All, CL1660.Contig8_All, CL1660.Contig9_All, CL1766.Contig1_All, CL1843.Contig3_All, CL1949.Contig6_All, CL2170.Contig2_All, CL2170.Contig5_All, CL2171.Contig2_All, CL2235.Contig1_All, CL2235.Contig2_All, CL2416.Contig1_All, CL249.Contig1_All, CL249.Contig3_All, CL249.Contig5_All, CL249.Contig6_All, CL2515.Contig1_All, CL2546.Contig1_All, CL2749.Contig2_All, CL2809.Contig2_All, CL2809.Contig3_All, CL2809.Contig8_All, CL2893.Contig1_All, CL2893.Contig2_All, CL2978.Contig2_All, CL2978.Contig3_All, CL3373.Contig1_All, CL3373.Contig2_All, CL4025.Contig3_All, CL4025.Contig4_All, CL415.Contig1_All, CL4319.Contig2_All, CL4335.Contig1_All, CL4335.Contig2_All, CL4402.Contig1_All, CL4514.Contig2_All, CL4615.Contig2_All, CL5064.Contig2_All, CL5206.Contig1_All, CL5218.Contig1_All, CL5218.Contig2_All, CL5304.Contig1_All, CL570.Contig1_All, CL570.Contig2_All, Unigene10019_All, Unigene10252_All, Unigene10253_All, Unigene10876_All, Unigene11040_All, Unigene1109_All, Unigene11227_All, Unigene11228_All, Unigene1125_All, Unigene11411_All, Unigene1181_All, Unigene11922_All, Unigene12099_All, Unigene12142_All, Unigene12161_All, Unigene1229_All, Unigene12361_All, Unigene12469_All, Unigene12522_All, Unigene12664_All, Unigene12740_All, Unigene12903_All, Unigene13421_All, Unigene14019_All, Unigene1538_All, Unigene16349_All, Unigene1663_All, Unigene17863_All, Unigene18584_All, Unigene198_All, Unigene20083_All, Unigene20226_All, Unigene2050_All, Unigene236_All, Unigene2374_All, Unigene247_All, Unigene3103_All, Unigene3171_All, Unigene4140_All, Unigene4156_All, Unigene47_All, Unigene4801_All, Unigene4808_All, Unigene4975_All, Unigene5070_All, Unigene5179_All, Unigene54_All, Unigene5781_All, Unigene5896_All, Unigene5897_All, Unigene6041_All, Unigene6253_All, Unigene6526_All, Unigene6667_All, Unigene6981_All, Unigene700_All, Unigene7297_All, Unigene7299_All, Unigene756_All, Unigene7671_All, Unigene7747_All, Unigene7869_All, Unigene7983_All, Unigene8075_All, Unigene8784_All, Unigene8972_All, Unigene9076_All, Unigene9264_All
150	Type II diabetes mellitus	CL1009.Contig1_All, CL1049.Contig4_All, CL1066.Contig1_All, CL1066.Contig2_All, CL1066.Contig3_All, CL241.Contig1_All, CL241.Contig7_All, CL2505.Contig3_All, CL2505.Contig4_All, CL3047.Contig1_All, CL3047.Contig2_All, CL367.Contig1_All, CL367.Contig2_All, CL367.Contig3_All, CL3796.Contig2_All, CL3796.Contig3_All, CL4040.Contig1_All, CL4855.Contig1_All, CL4855.Contig2_All, CL493.Contig2_All, CL5115.Contig1_All, CL695.Contig16_All, CL95.Contig1_All, CL95.Contig2_All, Unigene12112_All, Unigene12660_All, Unigene1279_All, Unigene18663_All, Unigene2032_All, Unigene220_All, Unigene3836_All, Unigene695_All, Unigene699_All, Unigene8056_All, Unigene8621_All, Unigene9730_All

151	Hepatitis C	CL1066.Contig1_All, CL1066.Contig2_All, CL1066.Contig3_All, CL1387.Contig1_All, CL1387.Contig3_All, CL1548.Contig2_All, CL1548.Contig3_All, CL1807.Contig1_All, CL1807.Contig2_All, CL1843.Contig3_All, CL2049.Contig1_All, CL2049.Contig3_All, CL2306.Contig2_All, CL2789.Contig2_All, CL2873.Contig1_All, CL2873.Contig2_All, CL345.Contig1_All, CL367.Contig1_All, CL367.Contig2_All, CL367.Contig3_All, CL4439.Contig2_All, CL4966.Contig1_All, CL5115.Contig1_All, CL5204.Contig1_All, CL5204.Contig2_All, CL695.Contig16_All, CL761.Contig1_All, CL761.Contig2_All, CL880.Contig3_All, Unigene10001_All, Unigene10174_All, Unigene10242_All, Unigene11874_All, Unigene11922_All, Unigene12006_All, Unigene12259_All, Unigene12314_All, Unigene12393_All, Unigene1247_All, Unigene12671_All, Unigene13073_All, Unigene13432_All, Unigene14208_All, Unigene17046_All, Unigene1757_All, Unigene220_All, Unigene236_All, Unigene247_All, Unigene2749_All, Unigene6020_All, Unigene699_All, Unigene7286_All, Unigene7297_All, Unigene8046_All, Unigene8747_All, Unigene8784_All, Unigene9251_All
152	DNA replication	CL1395.Contig2_All, CL1590.Contig4_All, CL2702.Contig1_All, CL2702.Contig2_All, CL2827.Contig4_All, CL2986.Contig2_All, CL3770.Contig2_All, CL4025.Contig3_All, CL4025.Contig4_All, CL4278.Contig2_All, CL4989.Contig1_All, CL4989.Contig2_All, CL91.Contig1_All, Unigene10181_All, Unigene10182_All, Unigene10261_All, Unigene10549_All, Unigene10838_All, Unigene12220_All, Unigene12361_All, Unigene14019_All, Unigene1505_All, Unigene15790_All, Unigene17292_All, Unigene18127_All, Unigene18761_All, Unigene20279_All, Unigene2890_All, Unigene4102_All, Unigene4925_All, Unigene5992_All, Unigene704_All, Unigene8228_All, Unigene8709_All, Unigene996_All
153	Folate biosynthesis	CL1313.Contig4_All, CL15.Contig1_All, CL15.Contig2_All, CL15.Contig3_All, CL1521.Contig1_All, CL1521.Contig2_All, CL1521.Contig3_All, CL1521.Contig5_All, CL2053.Contig1_All, CL2053.Contig2_All, CL4195.Contig2_All, CL4256.Contig1_All, CL5368.Contig1_All, Unigene10303_All, Unigene13292_All, Unigene13418_All, Unigene17515_All, Unigene9941_All
154	Taurine and hypotaurine metabolism	CL143.Contig1_All, CL143.Contig2_All, CL1599.Contig1_All, CL1599.Contig2_All, CL2671.Contig2_All, CL317.Contig2_All, Unigene10419_All
155	Pantothenate and CoA biosynthesis	CL1156.Contig3_All, CL1388.Contig1_All, CL1404.Contig2_All, CL27.Contig5_All, CL3129.Contig1_All, CL3129.Contig2_All, CL3129.Contig5_All, CL4346.Contig1_All, CL4790.Contig1_All, CL4790.Contig2_All, Unigene11026_All, Unigene11027_All, Unigene12959_All, Unigene13035_All, Unigene18595_All, Unigene20289_All
156	Cocaine addiction	CL1066.Contig1_All, CL1066.Contig2_All, CL1302.Contig1_All, CL1302.Contig2_All, CL1452.Contig1_All, CL2253.Contig2_All, CL2801.Contig1_All, CL2801.Contig2_All, CL3023.Contig1_All, CL3023.Contig2_All, CL3023.Contig3_All, CL3023.Contig4_All, CL3023.Contig5_All, CL3286.Contig1_All, CL3920.Contig2_All, CL3948.Contig3_All, CL4594.Contig1_All, CL4736.Contig1_All, CL4736.Contig2_All, CL870.Contig4_All, CL870.Contig8_All, CL870.Contig9_All, CL940.Contig3_All, CL940.Contig5_All, Unigene10058_All, Unigene11915_All, Unigene1417_All, Unigene1479_All, Unigene1757_All, Unigene19148_All, Unigene2029_All, Unigene2193_All, Unigene2760_All, Unigene3779_All, Unigene4112_All, Unigene4116_All, Unigene4532_All, Unigene5074_All, Unigene5261_All, Unigene5521_All, Unigene5869_All, Unigene6217_All, Unigene661_All, Unigene7298_All, Unigene7702_All, Unigene9733_All
157	RNA degradation	CL1127.Contig2_All, CL1127.Contig8_All, CL1542.Contig1_All, CL1542.Contig2_All, CL1714.Contig4_All, CL2154.Contig1_All, CL2154.Contig2_All, CL2865.Contig1_All, CL2865.Contig2_All, CL3436.Contig1_All, CL3436.Contig4_All, CL3436.Contig9_All, CL3960.Contig1_All, CL3960.Contig2_All, CL4417.Contig1_All, CL443.Contig1_All, CL4549.Contig1_All, CL4549.Contig2_All, CL4900.Contig2_All, CL739.Contig2_All, CL848.Contig2_All, CL893.Contig1_All, CL980.Contig1_All, CL980.Contig3_All, Unigene10041_All, Unigene10111_All, Unigene103_All, Unigene11061_All, Unigene11099_All, Unigene11245_All, Unigene11246_All, Unigene11380_All, Unigene11919_All, Unigene12005_All, Unigene12046_All, Unigene12122_All, Unigene12129_All, Unigene12202_All, Unigene12383_All, Unigene12681_All, Unigene12696_All, Unigene12841_All, Unigene13060_All, Unigene13157_All, Unigene13261_All, Unigene153_All, Unigene1570_All, Unigene1711_All, Unigene1811_All, Unigene18194_All, Unigene18319_All, Unigene2035_All, Unigene2513_All, Unigene2753_All, Unigene2772_All, Unigene3083_All, Unigene3179_All, Unigene3750_All, Unigene43_All, Unigene7202_All, Unigene8032_All, Unigene8248_All, Unigene8353_All, Unigene8655_All, Unigene9000_All, Unigene9106_All, Unigene9182_All, Unigene9611_All
158	Other types of O-glycan biosynthesis	CL1176.Contig4_All, CL2308.Contig1_All, CL2370.Contig2_All, CL2370.Contig3_All, CL2370.Contig4_All, CL26.Contig3_All, CL3654.Contig1_All, CL3654.Contig2_All, CL3827.Contig2_All, CL4110.Contig1_All, CL4110.Contig2_All, CL490.Contig1_All, CL490.Contig2_All, CL490.Contig3_All, CL5334.Contig2_All, CL720.Contig1_All, CL720.Contig4_All, CL744.Contig1_All, CL744.Contig2_All, Unigene1120_All, Unigene1143_All, Unigene12098_All, Unigene12297_All, Unigene147_All, Unigene15946_All, Unigene18028_All, Unigene18489_All, Unigene2107_All, Unigene2312_All, Unigene2660_All, Unigene3012_All, Unigene3125_All, Unigene3132_All, Unigene3701_All, Unigene3771_All, Unigene4342_All, Unigene4564_All, Unigene4675_All, Unigene526_All, Unigene5637_All, Unigene5751_All, Unigene6129_All, Unigene6276_All, Unigene6650_All, Unigene6673_All, Unigene8049_All, Unigene8218_All, Unigene9227_All
159	Natural killer cell mediated cytotoxicity	CL1066.Contig1_All, CL1066.Contig2_All, CL1066.Contig3_All, CL1535.Contig3_All, CL1807.Contig1_All, CL1807.Contig2_All, CL1831.Contig1_All, CL1831.Contig2_All, CL1874.Contig2_All, CL1954.Contig1_All, CL1954.Contig2_All, CL2182.Contig1_All, CL2182.Contig3_All, CL2182.Contig4_All, CL2222.Contig9_All, CL257.Contig1_All, CL257.Contig2_All, CL367.Contig1_All, CL367.Contig2_All, CL367.Contig3_All, CL39.Contig1_All, CL39.Contig2_All, CL39.Contig3_All, CL39.Contig4_All, CL3979.Contig1_All, CL3979.Contig2_All, CL4190.Contig1_All, CL4439.Contig2_All, CL4521.Contig1_All, CL4521.Contig2_All, CL4521.Contig3_All, CL4521.Contig5_All, CL4559.Contig2_All, CL4559.Contig3_All, CL4787.Contig1_All, CL5064.Contig2_All, CL5391.Contig1_All, CL5391.Contig2_All, CL5469.Contig2_All, CL695.Contig16_All, CL940.Contig3_All, CL940.Contig5_All, Unigene10174_All, Unigene12671_All, Unigene13853_All, Unigene18898_All, Unigene20260_All, Unigene7016_All, Unigene8046_All, Unigene80_All, Unigene8747_All, Unigene9719_All

160	Pyruvate metabolism	CL2294.Contig4_All, CL2309.Contig2_All, CL2309.Contig3_All, CL2430.Contig3_All, CL2557.Contig1_All, CL2694.Contig1_All, CL2694.Contig3_All, CL3832.Contig1_All, CL4130.Contig1_All, CL4130.Contig2_All, CL4144.Contig3_All, CL4144.Contig4_All, CL4355.Contig1_All, CL4355.Contig2_All, CL4451.Contig1_All, CL4451.Contig2_All, CL4753.Contig1_All, CL4971.Contig2_All, CL5301.Contig1_All, CL5301.Contig2_All, CL957.Contig6_All, Unigene10029_All, Unigene10098_All, Unigene10282_All, Unigene10299_All, Unigene10526_All, Unigene10686_All, Unigene10818_All, Unigene10908_All, Unigene11062_All, Unigene11186_All, Unigene11187_All, Unigene11188_All, Unigene11838_All, Unigene11930_All, Unigene1197_All, Unigene12053_All, Unigene12140_All, Unigene12219_All, Unigene12399_All, Unigene12491_All, Unigene12589_All, Unigene12643_All, Unigene12732_All, Unigene12752_All, Unigene12801_All, Unigene12918_All, Unigene13410_All, Unigene13671_All, Unigene13673_All, Unigene13773_All, Unigene15270_All, Unigene16206_All, Unigene16508_All, Unigene16942_All, Unigene17053_All, Unigene17410_All, Unigene17527_All, Unigene17957_All, Unigene18101_All, Unigene1811_All, Unigene18238_All, Unigene18312_All, Unigene18663_All, Unigene186_All, Unigene18841_All, Unigene18842_All, Unigene19038_All, Unigene19153_All, Unigene19332_All, Unigene19366_All, Unigene19739_All, Unigene1980_All, Unigene20691_All, Unigene20727_All, Unigene20815_All, Unigene236_All, Unigene2496_All, Unigene3282_All, Unigene3450_All, Unigene5053_All, Unigene627_All, Unigene6360_All, Unigene6618_All, Unigene6695_All, Unigene6954_All, Unigene7271_All, Unigene8037_All, Unigene8563_All, Unigene8732_All, Unigene874_All, Unigene999_All
161	Endometrial cancer	CL1066.Contig1_All, CL1066.Contig2_All, CL1066.Contig3_All, CL1104.Contig1_All, CL1104.Contig2_All, CL1104.Contig3_All, CL1104.Contig4_All, CL1104.Contig5_All, CL1104.Contig6_All, CL1104.Contig7_All, CL1104.Contig8_All, CL1104.Contig9_All, CL120.Contig2_All, CL1548.Contig2_All, CL1548.Contig3_All, CL1795.Contig5_All, CL1807.Contig1_All, CL1807.Contig2_All, CL1843.Contig3_All, CL2034.Contig1_All, CL2034.Contig2_All, CL2552.Contig3_All, CL2788.Contig2_All, CL3436.Contig1_All, CL3436.Contig4_All, CL3436.Contig9_All, CL367.Contig1_All, CL367.Contig2_All, CL367.Contig3_All, CL400.Contig1_All, CL400.Contig2_All, CL4158.Contig4_All, CL4158.Contig5_All, CL4158.Contig7_All, CL4439.Contig2_All, CL4787.Contig1_All, CL5212.Contig1_All, CL5212.Contig2_All, CL695.Contig16_All, Unigene10174_All, Unigene11922_All, Unigene12671_All, Unigene150_All, Unigene236_All, Unigene247_All, Unigene2742_All, Unigene2749_All, Unigene5727_All, Unigene6101_All, Unigene699_All, Unigene7297_All, Unigene777_All, Unigene783_All, Unigene8046_All, Unigene8747_All, Unigene8784_All, Unigene9740_All
162	Hematopoietic cell lineage	CL1062.Contig2_All, CL1175.Contig1_All, CL1175.Contig2_All, CL1370.Contig4_All, CL2331.Contig2_All, CL2331.Contig3_All, CL300.Contig7_All, CL3106.Contig1_All, CL4588.Contig2_All, CL4597.Contig2_All, CL4903.Contig1_All, CL5394.Contig2_All, Unigene106_All, Unigene11484_All, Unigene12736_All, Unigene15320_All, Unigene15321_All, Unigene15769_All, Unigene16906_All, Unigene17741_All, Unigene20073_All, Unigene2105_All, Unigene3324_All, Unigene4510_All, Unigene4757_All, Unigene5034_All, Unigene5065_All, Unigene5257_All, Unigene6916_All, Unigene7353_All, Unigene7836_All, Unigene8166_All, Unigene8217_All, Unigene8221_All, Unigene833_All, Unigene8662_All, Unigene8718_All, Unigene9038_All
163	Fat digestion and absorption	CL1004.Contig1_All, CL1064.Contig1_All, CL1064.Contig2_All, CL1751.Contig1_All, CL1911.Contig1_All, CL1911.Contig3_All, CL199.Contig1_All, CL2432.Contig2_All, CL2433.Contig10_All, CL2704.Contig1_All, CL2790.Contig2_All, CL2990.Contig1_All, CL2990.Contig2_All, CL344.Contig1_All, CL3541.Contig3_All, CL3541.Contig5_All, CL3897.Contig1_All, CL3897.Contig2_All, CL39.Contig1_All, CL39.Contig2_All, CL39.Contig3_All, CL39.Contig4_All, CL4485.Contig1_All, CL4485.Contig3_All, CL4602.Contig1_All, CL4845.Contig1_All, CL5235.Contig1_All, CL5235.Contig2_All, CL5310.Contig2_All, CL860.Contig10_All, CL860.Contig8_All, CL860.Contig9_All, Unigene10901_All, Unigene11336_All, Unigene12219_All, Unigene12475_All, Unigene13121_All, Unigene13673_All, Unigene17889_All, Unigene184_All, Unigene2699_All, Unigene3029_All, Unigene3033_All, Unigene3034_All, Unigene3036_All, Unigene3037_All, Unigene3042_All, Unigene4060_All, Unigene40_All, Unigene4873_All, Unigene5266_All, Unigene5713_All, Unigene7757_All, Unigene7936_All, Unigene8065_All, Unigene8187_All, Unigene8194_All, Unigene9026_All, Unigene9055_All, Unigene9238_All, Unigene9251_All
164	Pancreatic cancer	CL1066.Contig1_All, CL1066.Contig2_All, CL1066.Contig3_All, CL1439.Contig1_All, CL1439.Contig2_All, CL1535.Contig3_All, CL1548.Contig2_All, CL1548.Contig3_All, CL1789.Contig1_All, CL1789.Contig2_All, CL2573.Contig2_All, CL2573.Contig3_All, CL271.Contig2_All, CL3373.Contig1_All, CL3373.Contig2_All, CL367.Contig1_All, CL367.Contig2_All, CL367.Contig3_All, CL4271.Contig1_All, CL4439.Contig2_All, CL4787.Contig1_All, CL4896.Contig1_All, CL5115.Contig1_All, CL695.Contig16_All, Unigene10318_All, Unigene12099_All, Unigene12671_All, Unigene1757_All, Unigene2050_All, Unigene220_All, Unigene2374_All, Unigene2749_All, Unigene4671_All, Unigene4801_All, Unigene5789_All, Unigene699_All, Unigene7018_All, Unigene8747_All, Unigene8972_All
165	Propanoate metabolism	CL1353.Contig1_All, CL1353.Contig3_All, CL2309.Contig2_All, CL2309.Contig3_All, CL2694.Contig1_All, CL2694.Contig3_All, CL3832.Contig1_All, CL3963.Contig1_All, CL4144.Contig3_All, CL4144.Contig4_All, CL4451.Contig1_All, CL4451.Contig2_All, CL4570.Contig1_All, CL4646.Contig2_All, CL4753.Contig1_All, Unigene10157_All, Unigene10158_All, Unigene10282_All, Unigene10287_All, Unigene10299_All, Unigene10311_All, Unigene10526_All, Unigene10599_All, Unigene10686_All, Unigene10908_All, Unigene11003_All, Unigene11854_All, Unigene11887_All, Unigene1197_All, Unigene12140_All, Unigene12169_All, Unigene12187_All, Unigene12219_All, Unigene12293_All, Unigene12412_All, Unigene12643_All, Unigene13080_All, Unigene13152_All, Unigene13410_All, Unigene13431_All, Unigene13445_All, Unigene13673_All, Unigene13773_All, Unigene14111_All, Unigene15270_All, Unigene15429_All, Unigene16206_All, Unigene16942_All, Unigene17527_All, Unigene18101_All, Unigene186_All, Unigene19038_All, Unigene19153_All, Unigene19366_All, Unigene20691_All, Unigene2853_All, Unigene3282_All, Unigene3450_All, Unigene5053_All, Unigene6360_All, Unigene7886_All, Unigene8037_All

166	Carbohydrate digestion and absorption	CL1066.Contig1_All, CL1066.Contig2_All, CL1066.Contig3_All, CL2137.Contig1_All, CL2137.Contig2_All, CL2182.Contig1_All, CL2182.Contig3_All, CL2182.Contig4_All, CL2474.Contig2_All, CL2930.Contig5_All, CL367.Contig1_All, CL367.Contig2_All, CL367.Contig3_All, CL3776.Contig2_All, CL39.Contig1_All, CL39.Contig2_All, CL39.Contig3_All, CL39.Contig4_All, CL41.Contig3_All, CL41.Contig4_All, CL41.Contig8_All, CL493.Contig2_All, CL5115.Contig3_All, CL5391.Contig1_All, CL5391.Contig2_All, CL695.Contig16_All, CL940.Contig3_All, CL940.Contig5_All, Unigene10594_All, Unigene11480_All, Unigene11482_All, Unigene11483_All, Unigene12660_All, Unigene17323_All, Unigene17324_All, Unigene18652_All, Unigene18898_All, Unigene20899_All, Unigene2209_All, Unigene2749_All, Unigene3010_All, Unigene4053_All, Unigene4070_All, Unigene4323_All, Unigene5047_All, Unigene5724_All, Unigene6215_All, Unigene695_All, Unigene7206_All, Unigene8422_All, Unigene8621_All
167	Insect hormone biosynthesis	CL5125.Contig1_All, CL887.Contig1_All, Unigene11671_All, Unigene17598_All
168	PPAR signaling pathway	CL102.Contig1_All, CL102.Contig2_All, CL1091.Contig14_All, CL1353.Contig1_All, CL1353.Contig3_All, CL1387.Contig1_All, CL1387.Contig3_All, CL1751.Contig1_All, CL1795.Contig5_All, CL1911.Contig1_All, CL1911.Contig3_All, CL1939.Contig1_All, CL205.Contig1_All, CL205.Contig2_All, CL2430.Contig3_All, CL257.Contig5_All, CL2808.Contig1_All, CL2943.Contig1_All, CL2943.Contig2_All, CL2990.Contig1_All, CL2990.Contig2_All, CL35.Contig11_All, CL35.Contig1_All, CL35.Contig7_All, CL3536.Contig1_All, CL3536.Contig2_All, CL3536.Contig5_All, CL4427.Contig2_All, CL4427.Contig3_All, CL4646.Contig2_All, CL4813.Contig1_All, CL4813.Contig2_All, CL4813.Contig3_All, CL507.Contig7_All, CL5204.Contig1_All, CL5204.Contig2_All, CL5212.Contig1_All, CL5212.Contig2_All, CL5264.Contig2_All, CL63.Contig10_All, CL63.Contig9_All, CL866.Contig1_All, CL866.Contig2_All, CL880.Contig3_All, Unigene10001_All, Unigene10029_All, Unigene10157_All, Unigene10158_All, Unigene10311_All, Unigene10866_All, Unigene10867_All, Unigene10896_All, Unigene10924_All, Unigene11164_All, Unigene11394_All, Unigene11645_All, Unigene11772_All, Unigene11815_All, Unigene11839_All, Unigene11862_All, Unigene11955_All, Unigene11960_All, Unigene11969_All, Unigene11975_All, Unigene11997_All, Unigene12021_All, Unigene13271_All, Unigene16671_All, Unigene16926_All, Unigene17202_All, Unigene1819_All, Unigene19070_All, Unigene19332_All, Unigene19739_All, Unigene20727_All, Unigene2742_All, Unigene350_All, Unigene40_All, Unigene693_All, Unigene7046_All, Unigene761_All, Unigene818_All, Unigene8860_All, Unigene9028_All, Unigene9154_All, Unigene9238_All, Unigene9634_All, Unigene9714_All, Unigene9974_All
169	Riboflavin metabolism	CL1124.Contig1_All, CL1388.Contig1_All, CL161.Contig4_All, CL1685.Contig2_All, CL252.Contig2_All, CL252.Contig3_All, CL3156.Contig1_All, CL4346.Contig1_All, CL4393.Contig1_All, CL4393.Contig2_All, CL4605.Contig1_All, CL4605.Contig2_All, Unigene10226_All, Unigene10747_All, Unigene10994_All, Unigene16438_All, Unigene20289_All, Unigene2915_All, Unigene3159_All, Unigene4148_All, Unigene4637_All, Unigene5774_All, Unigene6162_All, Unigene8213_All, Unigene9034_All
170	Cytosolic DNA-sensing pathway	CL1411.Contig1_All, CL1411.Contig8_All, CL1683.Contig2_All, CL1683.Contig7_All, CL2306.Contig2_All, CL322.Contig4_All, CL322.Contig5_All, CL322.Contig6_All, CL3251.Contig1_All, CL3251.Contig2_All, CL3635.Contig3_All, CL3657.Contig3_All, CL3792.Contig3_All, CL5004.Contig1_All, CL5051.Contig2_All, CL5461.Contig2_All, Unigene11623_All, Unigene1205_All, Unigene12060_All, Unigene12096_All, Unigene12479_All, Unigene1247_All, Unigene13002_All, Unigene1757_All, Unigene18801_All, Unigene19657_All, Unigene2131_All, Unigene24_All, Unigene3081_All, Unigene3288_All, Unigene3390_All, Unigene33_All, Unigene3659_All, Unigene7192_All, Unigene7296_All, Unigene7750_All, Unigene7993_All, Unigene8008_All, Unigene8228_All, Unigene8236_All, Unigene9107_All, Unigene9758_All
171	Mineral absorption	CL1212.Contig1_All, CL1540.Contig1_All, CL1591.Contig2_All, CL1880.Contig1_All, CL1880.Contig2_All, CL1880.Contig3_All, CL190.Contig1_All, CL2137.Contig1_All, CL2137.Contig2_All, CL2474.Contig2_All, CL2709.Contig1_All, CL2709.Contig2_All, CL2711.Contig1_All, CL2711.Contig2_All, CL3160.Contig2_All, CL3776.Contig2_All, CL4084.Contig1_All, CL4084.Contig2_All, CL41.Contig3_All, CL41.Contig4_All, CL41.Contig8_All, CL4450.Contig2_All, CL5115.Contig3_All, CL531.Contig1_All, CL5440.Contig1_All, CL573.Contig6_All, CL703.Contig2_All, CL703.Contig3_All, CL703.Contig5_All, CL703.Contig6_All, CL878.Contig1_All, CL878.Contig2_All, CL878.Contig3_All, CL878.Contig4_All, CL878.Contig5_All, Unigene10594_All, Unigene11480_All, Unigene11482_All, Unigene11483_All, Unigene11804_All, Unigene11824_All, Unigene11897_All, Unigene12077_All, Unigene12254_All, Unigene12427_All, Unigene12594_All, Unigene12749_All, Unigene16321_All, Unigene16575_All, Unigene19085_All, Unigene19677_All, Unigene2079_All, Unigene20899_All, Unigene2101_All, Unigene2274_All, Unigene2763_All, Unigene3010_All, Unigene3177_All, Unigene4163_All, Unigene4323_All, Unigene5047_All, Unigene5724_All, Unigene6215_All, Unigene62_All, Unigene6597_All, Unigene6809_All, Unigene7075_All, Unigene7206_All, Unigene808_All, Unigene8700_All, Unigene9163_All
172	Chronic myeloid leukemia	CL1066.Contig1_All, CL1066.Contig2_All, CL1066.Contig3_All, CL1439.Contig1_All, CL1439.Contig2_All, CL1807.Contig1_All, CL1807.Contig2_All, CL1874.Contig2_All, CL1953.Contig1_All, CL2038.Contig1_All, CL2038.Contig7_All, CL2222.Contig9_All, CL2287.Contig2_All, CL2287.Contig4_All, CL2346.Contig1_All, CL2346.Contig2_All, CL3373.Contig1_All, CL3373.Contig2_All, CL3632.Contig2_All, CL3632.Contig3_All, CL367.Contig1_All, CL367.Contig2_All, CL367.Contig3_All, CL4271.Contig1_All, CL4439.Contig2_All, CL4559.Contig2_All, CL4559.Contig3_All, CL4787.Contig1_All, CL695.Contig16_All, Unigene1000_All, Unigene10174_All, Unigene12099_All, Unigene12671_All, Unigene13036_All, Unigene1663_All, Unigene1757_All, Unigene198_All, Unigene2050_All, Unigene2374_All, Unigene2749_All, Unigene3168_All, Unigene4084_All, Unigene4142_All, Unigene4801_All, Unigene5179_All, Unigene6222_All, Unigene7016_All, Unigene7983_All, Unigene8046_All, Unigene8747_All, Unigene8972_All, Unigene9641_All
173	Biosynthesis of unsaturated fatty acids	CL1353.Contig1_All, CL1353.Contig3_All, CL1939.Contig1_All, CL3963.Contig1_All, CL4646.Contig2_All, CL5264.Contig2_All, CL63.Contig10_All, CL63.Contig9_All, Unigene10157_All, Unigene10158_All, Unigene10311_All, Unigene10693_All, Unigene10866_All, Unigene10867_All, Unigene10890_All, Unigene11394_All, Unigene11808_All, Unigene11839_All, Unigene11842_All, Unigene11862_All, Unigene11975_All, Unigene12108_All, Unigene12123_All, Unigene12578_All, Unigene13152_All, Unigene13211_All, Unigene15429_All, Unigene19070_All, Unigene4011_All, Unigene4845_All, Unigene4858_All, Unigene5230_All, Unigene6980_All, Unigene9927_All

174	TGF-beta signaling pathway	CL1439.Contig1_All, CL1439.Contig2_All, CL2170.Contig2_All, CL2170.Contig5_All, CL2224.Contig2_All, CL2224.Contig3_All, CL2224.Contig4_All, CL2235.Contig1_All, CL2235.Contig2_All, CL2537.Contig1_All, CL2684.Contig1_All, CL2684.Contig2_All, CL3183.Contig2_All, CL3222.Contig2_All, CL3786.Contig6_All, CL3823.Contig1_All, CL3823.Contig2_All, CL4271.Contig1_All, CL4271.Contig2_All, CL4496.Contig3_All, CL4514.Contig2_All, CL5106.Contig1_All, CL915.Contig3_All, CL915.Contig5_All, Unigene10242_All, Unigene10876_All, Unigene11040_All, Unigene11307_All, Unigene11411_All, Unigene12740_All, Unigene12903_All, Unigene13432_All, Unigene1767_All, Unigene18644_All, Unigene2050_All, Unigene2956_All, Unigene3216_All, Unigene4144_All, Unigene4801_All, Unigene4975_All, Unigene5493_All, Unigene54_All, Unigene5781_All, Unigene5808_All, Unigene7063_All, Unigene7671_All, Unigene7745_All, Unigene8972_All, Unigene9050_All, Unigene9172_All, Unigene9877_All
175	Primary bile acid biosynthesis	CL1300.Contig4_All, CL1300.Contig6_All, CL1842.Contig1_All, CL1842.Contig3_All, CL257.Contig5_All, CL3212.Contig1_All, CL3991.Contig1_All, CL4171.Contig1_All, CL4427.Contig2_All, CL4427.Contig3_All, CL4700.Contig1_All, Unigene10250_All, Unigene11969_All, Unigene12910_All, Unigene13271_All, Unigene666_All, Unigene9724_All
176	T cell receptor signaling pathway	CL1066.Contig1_All, CL1066.Contig2_All, CL1066.Contig3_All, CL1807.Contig1_All, CL1807.Contig2_All, CL1831.Contig1_All, CL1831.Contig2_All, CL1843.Contig3_All, CL188.Contig16_All, CL1953.Contig1_All, CL1954.Contig1_All, CL1954.Contig2_All, CL257.Contig1_All, CL257.Contig2_All, CL271.Contig2_All, CL2722.Contig1_All, CL2977.Contig1_All, CL2977.Contig2_All, CL3047.Contig1_All, CL3047.Contig2_All, CL367.Contig1_All, CL367.Contig2_All, CL367.Contig3_All, CL3979.Contig1_All, CL3979.Contig2_All, CL4190.Contig1_All, CL4439.Contig2_All, CL4521.Contig1_All, CL4521.Contig2_All, CL4521.Contig3_All, CL4521.Contig5_All, CL4736.Contig1_All, CL4736.Contig2_All, CL4787.Contig1_All, CL4815.Contig1_All, CL4815.Contig2_All, CL4855.Contig1_All, CL4855.Contig2_All, CL4966.Contig1_All, CL5005.Contig1_All, CL5005.Contig2_All, CL5064.Contig2_All, CL5115.Contig1_All, CL5469.Contig2_All, CL695.Contig16_All, Unigene10174_All, Unigene11307_All, Unigene11922_All, Unigene12099_All, Unigene12671_All, Unigene13853_All, Unigene1479_All, Unigene15257_All, Unigene1757_All, Unigene220_All, Unigene236_All, Unigene2374_All, Unigene247_All, Unigene2749_All, Unigene3131_All, Unigene4081_All, Unigene5808_All, Unigene7297_All, Unigene8046_All, Unigene8056_All, Unigene8725_All, Unigene8784_All, Unigene9050_All, Unigene9719_All, Unigene9877_All
177	Ubiquitin mediated proteolysis	CL1080.Contig2_All, CL1124.Contig1_All, CL1612.Contig1_All, CL1612.Contig2_All, CL1660.Contig10_All, CL1660.Contig15_All, CL1660.Contig16_All, CL1660.Contig1_All, CL1660.Contig3_All, CL1660.Contig5_All, CL1660.Contig6_All, CL1660.Contig7_All, CL1660.Contig8_All, CL1660.Contig9_All, CL1953.Contig1_All, CL2.Contig1_All, CL2068.Contig5_All, CL2159.Contig2_All, CL2217.Contig1_All, CL2217.Contig2_All, CL2217.Contig3_All, CL2416.Contig1_All, CL2716.Contig2_All, CL2749.Contig2_All, CL2793.Contig1_All, CL2793.Contig2_All, CL2809.Contig2_All, CL2809.Contig3_All, CL2809.Contig8_All, CL2838.Contig1_All, CL2838.Contig2_All, CL2838.Contig4_All, CL2869.Contig3_All, CL2978.Contig2_All, CL2978.Contig3_All, CL2986.Contig1_All, CL2986.Contig2_All, CL3153.Contig1_All, CL3153.Contig2_All, CL3162.Contig2_All, CL3517.Contig1_All, CL3713.Contig1_All, CL4162.Contig1_All, CL4162.Contig2_All, CL4203.Contig4_All, CL4366.Contig1_All, CL4511.Contig1_All, CL4514.Contig2_All, CL4558.Contig1_All, CL4558.Contig2_All, CL4558.Contig3_All, CL4615.Contig2_All, CL4738.Contig1_All, CL4757.Contig2_All, CL4804.Contig1_All, CL5064.Contig2_All, CL5218.Contig1_All, CL5218.Contig2_All, CL5264.Contig2_All, CL5304.Contig1_All, CL570.Contig1_All, CL570.Contig2_All, CL862.Contig1_All, CL862.Contig2_All, CL862.Contig3_All, CL862.Contig4_All, CL987.Contig2_All, Unigene10019_All, Unigene100_All, Unigene1022_All, Unigene1038_All, Unigene1056_All, Unigene1061_All, Unigene10876_All, Unigene11040_All, Unigene11227_All, Unigene11228_All, Unigene1125_All, Unigene11411_All, Unigene1176_All, Unigene1181_All, Unigene11828_All, Unigene11839_All, Unigene11877_All, Unigene118_All, Unigene11944_All, Unigene11957_All, Unigene11975_All, Unigene12157_All, Unigene1229_All, Unigene12363_All, Unigene12740_All, Unigene12868_All, Unigene12903_All, Unigene1322_All, Unigene13588_All, Unigene16349_All, Unigene1769_All, Unigene17863_All, Unigene18584_All, Unigene18752_All, Unigene20083_All, Unigene20226_All, Unigene2069_All, Unigene3791_All, Unigene47_All, Unigene4975_All, Unigene5037_All, Unigene5897_All, Unigene6020_All, Unigene6856_All, Unigene6931_All, Unigene7044_All, Unigene7070_All, Unigene7088_All, Unigene7120_All, Unigene7220_All, Unigene754_All, Unigene756_All, Unigene7671_All, Unigene7747_All, Unigene7780_All, Unigene798_All, Unigene806_All, Unigene8277_All, Unigene83_All, Unigene8710_All, Unigene8_All, Unigene9115_All
178	Thyroid cancer	CL1091.Contig14_All, CL1637.Contig1_All, CL3235.Contig1_All, CL3235.Contig2_All, CL3270.Contig1_All, CL3270.Contig2_All, CL349.Contig12_All, CL349.Contig3_All, CL349.Contig5_All, CL4381.Contig1_All, CL4439.Contig2_All, CL4787.Contig1_All, CL5204.Contig1_All, CL5204.Contig2_All, CL880.Contig3_All, Unigene1150_All, Unigene11923_All, Unigene12671_All, Unigene1699_All, Unigene17718_All, Unigene4137_All, Unigene4161_All, Unigene5068_All, Unigene5727_All, Unigene7287_All, Unigene777_All, Unigene783_All, Unigene8003_All, Unigene8747_All, Unigene9039_All, Unigene9740_All
179	Epithelial cell signaling in Helicobacter pylori infection	CL1535.Contig3_All, CL1548.Contig2_All, CL1548.Contig3_All, CL1966.Contig2_All, CL2150.Contig3_All, CL2222.Contig9_All, CL2634.Contig1_All, CL2634.Contig2_All, CL2658.Contig1_All, CL2658.Contig2_All, CL271.Contig2_All, CL3091.Contig1_All, CL3091.Contig2_All, CL3091.Contig3_All, CL3091.Contig4_All, CL3657.Contig1_All, CL3884.Contig2_All, CL3979.Contig1_All, CL3979.Contig2_All, CL4090.Contig2_All, CL4381.Contig1_All, CL4459.Contig1_All, CL4628.Contig1_All, CL4736.Contig1_All, CL4736.Contig2_All, CL4966.Contig1_All, CL5115.Contig1_All, CL5469.Contig2_All, CL823.Contig1_All, Unigene10026_All, Unigene10483_All, Unigene11240_All, Unigene11899_All, Unigene1189_All, Unigene12028_All, Unigene12168_All, Unigene12209_All, Unigene12302_All, Unigene12423_All, Unigene12923_All, Unigene13508_All, Unigene13589_All, Unigene1479_All, Unigene15969_All, Unigene1667_All, Unigene1679_All, Unigene1741_All, Unigene1757_All, Unigene17602_All, Unigene18707_All, Unigene220_All, Unigene3351_All, Unigene3730_All, Unigene4133_All, Unigene6920_All, Unigene6977_All, Unigene699_All, Unigene8107_All, Unigene925_All, Unigene9719_All

180	Protein export	CL2403.Contig1_All, CL3742.Contig1_All, CL4338.Contig1_All, CL4338.Contig2_All, CL4338.Contig3_All, CL4666.Contig1_All, CL4666.Contig2_All, CL5034.Contig1_All, CL5034.Contig2_All, CL5225.Contig1_All, CL678.Contig17_All, Unigene10374_All, Unigene10433_All, Unigene11884_All, Unigene11908_All, Unigene11988_All, Unigene12204_All, Unigene12457_All, Unigene12460_All, Unigene12709_All, Unigene12890_All, Unigene12920_All, Unigene13331_All, Unigene13352_All, Unigene7723_All, Unigene8950_All
181	Aldosterone-regulated sodium reabsorption	CL1009.Contig1_All, CL1066.Contig1_All, CL1066.Contig2_All, CL1066.Contig3_All, CL1730.Contig2_All, CL2137.Contig1_All, CL2137.Contig2_All, CL2182.Contig1_All, CL2182.Contig3_All, CL2182.Contig4_All, CL241.Contig1_All, CL241.Contig7_All, CL2474.Contig2_All, CL2505.Contig3_All, CL2505.Contig4_All, CL367.Contig1_All, CL367.Contig2_All, CL367.Contig3_All, CL3776.Contig2_All, CL39.Contig1_All, CL39.Contig2_All, CL39.Contig3_All, CL39.Contig4_All, CL4085.Contig1_All, CL4085.Contig2_All, CL41.Contig3_All, CL41.Contig4_All, CL41.Contig8_All, CL4119.Contig1_All, CL4316.Contig1_All, CL4439.Contig2_All, CL5280.Contig1_All, CL5391.Contig1_All, CL5391.Contig2_All, CL695.Contig16_All, CL940.Contig3_All, CL940.Contig5_All, CL95.Contig1_All, CL95.Contig2_All, Unigene10594_All, Unigene11480_All, Unigene11482_All, Unigene11483_All, Unigene124_All, Unigene12671_All, Unigene16125_All, Unigene18035_All, Unigene18898_All, Unigene19476_All, Unigene19524_All, Unigene207_All, Unigene2742_All, Unigene3010_All, Unigene3836_All, Unigene4323_All, Unigene4950_All, Unigene49_All, Unigene5724_All, Unigene6151_All, Unigene6215_All, Unigene699_All, Unigene7206_All, Unigene7322_All, Unigene8081_All, Unigene8114_All
182	beta-Alanine metabolism	CL1156.Contig3_All, CL1353.Contig1_All, CL1353.Contig3_All, CL1599.Contig1_All, CL1599.Contig2_All, CL2309.Contig2_All, CL2309.Contig3_All, CL2694.Contig1_All, CL2694.Contig3_All, CL313.Contig2_All, CL3963.Contig1_All, CL4144.Contig3_All, CL4144.Contig4_All, CL4451.Contig1_All, CL4451.Contig2_All, CL4646.Contig2_All, CL4753.Contig1_All, Unigene10157_All, Unigene10158_All, Unigene10282_All, Unigene10299_All, Unigene10311_All, Unigene10526_All, Unigene10686_All, Unigene10908_All, Unigene11003_All, Unigene11026_All, Unigene11027_All, Unigene11887_All, Unigene1197_All, Unigene12187_All, Unigene12412_All, Unigene13152_All, Unigene13410_All, Unigene13445_All, Unigene13773_All, Unigene15270_All, Unigene15429_All, Unigene16206_All, Unigene17527_All, Unigene18101_All, Unigene186_All, Unigene19038_All, Unigene2853_All, Unigene3450_All, Unigene5053_All, Unigene6360_All
183	Glutathione metabolism	CL1175.Contig1_All, CL1175.Contig2_All, CL143.Contig1_All, CL143.Contig2_All, CL1584.Contig2_All, CL16.Contig10_All, CL2411.Contig1_All, CL2445.Contig1_All, CL2456.Contig3_All, CL2512.Contig2_All, CL300.Contig7_All, CL3078.Contig1_All, CL317.Contig2_All, CL3618.Contig1_All, CL372.Contig1_All, CL3784.Contig1_All, CL3788.Contig1_All, CL4091.Contig1_All, CL4091.Contig2_All, CL4453.Contig1_All, CL450.Contig10_All, CL4903.Contig1_All, CL4969.Contig1_All, Unigene10381_All, Unigene10418_All, Unigene106_All, Unigene10735_All, Unigene10887_All, Unigene11286_All, Unigene11484_All, Unigene11916_All, Unigene11949_All, Unigene12008_All, Unigene12143_All, Unigene12227_All, Unigene12281_All, Unigene12325_All, Unigene12530_All, Unigene12565_All, Unigene12818_All, Unigene13093_All, Unigene1385_All, Unigene1464_All, Unigene15435_All, Unigene15451_All, Unigene1694_All, Unigene17741_All, Unigene18651_All, Unigene19310_All, Unigene2156_All, Unigene2914_All, Unigene3689_All, Unigene3936_All, Unigene4869_All, Unigene5034_All, Unigene5834_All, Unigene5870_All, Unigene6056_All, Unigene6708_All, Unigene7200_All, Unigene7258_All, Unigene7548_All, Unigene7814_All, Unigene8166_All, Unigene8718_All, Unigene8997_All, Unigene9038_All, Unigene908_All, Unigene9298_All, Unigene9637_All
184	VEGF signaling pathway	CL1066.Contig1_All, CL1066.Contig2_All, CL1066.Contig3_All, CL1535.Contig3_All, CL1831.Contig1_All, CL1831.Contig2_All, CL1874.Contig2_All, CL199.Contig1_All, CL2066.Contig1_All, CL2182.Contig1_All, CL2182.Contig3_All, CL2182.Contig4_All, CL2432.Contig2_All, CL271.Contig2_All, CL3116.Contig2_All, CL3290.Contig2_All, CL3290.Contig3_All, CL367.Contig1_All, CL367.Contig2_All, CL367.Contig3_All, CL3673.Contig2_All, CL39.Contig1_All, CL39.Contig2_All, CL39.Contig3_All, CL39.Contig4_All, CL3979.Contig1_All, CL3979.Contig2_All, CL4190.Contig1_All, CL4439.Contig2_All, CL4521.Contig1_All, CL4521.Contig2_All, CL4521.Contig3_All, CL4521.Contig5_All, CL4559.Contig2_All, CL4559.Contig3_All, CL4787.Contig1_All, CL4966.Contig1_All, CL5064.Contig2_All, CL5310.Contig2_All, CL5391.Contig1_All, CL5391.Contig2_All, CL695.Contig16_All, CL748.Contig1_All, CL748.Contig3_All, CL821.Contig1_All, CL821.Contig2_All, CL860.Contig10_All, CL860.Contig8_All, CL860.Contig9_All, CL940.Contig3_All, CL940.Contig5_All, Unigene11222_All, Unigene11810_All, Unigene12484_All, Unigene12671_All, Unigene13853_All, Unigene1855_All, Unigene18898_All, Unigene19601_All, Unigene2749_All, Unigene3029_All, Unigene3033_All, Unigene3034_All, Unigene3036_All, Unigene3037_All, Unigene3042_All, Unigene3088_All, Unigene4359_All, Unigene5813_All, Unigene7016_All, Unigene7547_All, Unigene9379_All
185	Toll-like receptor signaling pathway	CL1066.Contig1_All, CL1066.Contig2_All, CL1066.Contig3_All, CL1535.Contig3_All, CL1966.Contig2_All, CL2049.Contig1_All, CL2049.Contig3_All, CL2129.Contig4_All, CL2789.Contig2_All, CL367.Contig1_All, CL367.Contig2_All, CL367.Contig3_All, CL4057.Contig1_All, CL4320.Contig3_All, CL4736.Contig1_All, CL4736.Contig2_All, CL4787.Contig1_All, CL4815.Contig1_All, CL4815.Contig2_All, CL4966.Contig1_All, CL504.Contig1_All, CL504.Contig2_All, CL5115.Contig1_All, CL695.Contig16_All, Unigene10296_All, Unigene12139_All, Unigene1326_All, Unigene1479_All, Unigene17046_All, Unigene1757_All, Unigene18114_All, Unigene20095_All, Unigene220_All, Unigene2749_All, Unigene3131_All, Unigene3982_All, Unigene6977_All, Unigene7286_All, Unigene8246_All
186	Mucin type O-Glycan biosynthesis	CL3006.Contig2_All, CL3756.Contig1_All, CL3756.Contig2_All, CL3945.Contig4_All, CL3945.Contig6_All, CL3945.Contig8_All, CL5085.Contig2_All, Unigene1579_All, Unigene17052_All, Unigene3174_All, Unigene5073_All, Unigene6714_All, Unigene7057_All, Unigene7650_All, Unigene8723_All
187	Non-homologous end-joining	CL2300.Contig1_All, CL4777.Contig1_All, Unigene3089_All, Unigene35_All, Unigene383_All, Unigene4925_All
188	Bladder cancer	CL1170.Contig1_All, CL1548.Contig2_All, CL1548.Contig3_All, CL1695.Contig8_All, CL2935.Contig1_All, CL2935.Contig3_All, CL3373.Contig1_All, CL3373.Contig2_All, CL3802.Contig2_All, CL4439.Contig2_All, CL4787.Contig1_All, Unigene12099_All, Unigene12671_All, Unigene1757_All, Unigene2374_All, Unigene699_All, Unigene8747_All, Unigene9028_All, Unigene9740_All

189	Terpenoid backbone biosynthesis	CL2246.Contig3_All, CL2246.Contig6_All, CL2246.Contig8_All, CL4773.Contig1_All, CL4773.Contig2_All, Unigene10488_All, Unigene12219_All, Unigene12374_All, Unigene12646_All, Unigene13129_All, Unigene13673_All, Unigene5207_All, Unigene765_All, Unigene8174_All, Unigene9634_All
190	Sulfur metabolism	CL57.Contig2_All, Unigene12541_All, Unigene12560_All, Unigene12760_All, Unigene12843_All, Unigene4739_All, Unigene5758_All, Unigene6831_All, Unigene6996_All, Unigene6997_All, Unigene7000_All
191	Fatty acid elongation	CL3963.Contig1_All, CL4673.Contig1_All, CL4673.Contig2_All, CL4673.Contig4_All, Unigene10475_All, Unigene10476_All, Unigene10693_All, Unigene10890_All, Unigene11230_All, Unigene11231_All, Unigene11232_All, Unigene12108_All, Unigene12123_All, Unigene12187_All, Unigene12419_All, Unigene12422_All, Unigene12480_All, Unigene12578_All, Unigene13152_All, Unigene13211_All, Unigene14496_All, Unigene15429_All, Unigene1575_All, Unigene2215_All, Unigene2853_All, Unigene4011_All, Unigene4845_All, Unigene4853_All, Unigene4858_All, Unigene5230_All, Unigene6980_All
192	N-Glycan biosynthesis	CL1135.Contig2_All, CL1478.Contig1_All, CL1478.Contig2_All, CL1508.Contig1_All, CL1508.Contig2_All, CL1513.Contig1_All, CL1961.Contig2_All, CL1990.Contig1_All, CL1990.Contig2_All, CL2305.Contig1_All, CL2305.Contig3_All, CL2305.Contig5_All, CL2401.Contig1_All, CL2626.Contig1_All, CL2648.Contig3_All, CL3453.Contig4_All, CL4132.Contig2_All, CL4221.Contig1_All, CL4659.Contig1_All, CL5081.Contig2_All, CL906.Contig3_All, CL906.Contig8_All, Unigene10420_All, Unigene10421_All, Unigene114_All, Unigene12001_All, Unigene12453_All, Unigene12503_All, Unigene12621_All, Unigene12750_All, Unigene13047_All, Unigene13644_All, Unigene155_All, Unigene3726_All, Unigene5770_All, Unigene7234_All, Unigene7732_All, Unigene782_All, Unigene8291_All
193	Butanoate metabolism	CL1000.Contig2_All, CL1599.Contig1_All, CL1599.Contig2_All, CL2294.Contig4_All, CL2831.Contig1_All, CL2956.Contig2_All, CL3963.Contig1_All, Unigene10315_All, Unigene10475_All, Unigene10476_All, Unigene11062_All, Unigene11887_All, Unigene12187_All, Unigene12219_All, Unigene12236_All, Unigene12245_All, Unigene12491_All, Unigene12589_All, Unigene12801_All, Unigene12835_All, Unigene13152_All, Unigene13495_All, Unigene13673_All, Unigene15429_All, Unigene17053_All, Unigene17410_All, Unigene18312_All, Unigene18842_All, Unigene2215_All, Unigene2257_All, Unigene2853_All, Unigene2945_All, Unigene3281_All, Unigene4011_All, Unigene4853_All, Unigene4967_All, Unigene6618_All, Unigene9090_All, Unigene9634_All
194	Tryptophan metabolism	CL1650.Contig1_All, CL1707.Contig1_All, CL1711.Contig1_All, CL1711.Contig2_All, CL1711.Contig3_All, CL1711.Contig4_All, CL2309.Contig2_All, CL2309.Contig3_All, CL2532.Contig1_All, CL2532.Contig2_All, CL2590.Contig1_All, CL2590.Contig2_All, CL2694.Contig1_All, CL2694.Contig3_All, CL2707.Contig1_All, CL3127.Contig1_All, CL3497.Contig1_All, CL3511.Contig1_All, CL3511.Contig2_All, CL364.Contig1_All, CL364.Contig4_All, CL364.Contig7_All, CL3643.Contig2_All, CL3643.Contig6_All, CL3963.Contig1_All, CL4144.Contig3_All, CL4144.Contig4_All, CL4451.Contig1_All, CL4451.Contig2_All, CL4594.Contig1_All, CL4686.Contig2_All, CL4753.Contig1_All, CL5197.Contig2_All, CL5288.Contig2_All, CL551.Contig1_All, CL551.Contig2_All, Unigene10282_All, Unigene10299_All, Unigene10475_All, Unigene10476_All, Unigene10526_All, Unigene10686_All, Unigene10908_All, Unigene1134_All, Unigene1197_All, Unigene12187_All, Unigene12219_All, Unigene12252_All, Unigene12688_All, Unigene12738_All, Unigene12937_All, Unigene13152_All, Unigene13410_All, Unigene13441_All, Unigene13673_All, Unigene13773_All, Unigene15270_All, Unigene15429_All, Unigene16206_All, Unigene1623_All, Unigene16322_All, Unigene17527_All, Unigene18101_All, Unigene18521_All, Unigene18597_All, Unigene186_All, Unigene19038_All, Unigene20494_All, Unigene2078_All, Unigene2215_All, Unigene2724_All, Unigene27_All, Unigene2853_All, Unigene3105_All, Unigene3134_All, Unigene3450_All, Unigene4853_All, Unigene4884_All, Unigene5053_All, Unigene5060_All, Unigene5433_All, Unigene5600_All, Unigene5908_All, Unigene6134_All, Unigene6360_All, Unigene7135_All, Unigene7239_All, Unigene7663_All, Unigene8012_All, Unigene848_All, Unigene9060_All
195	Fanconi anemia pathway	CL120.Contig2_All, CL1484.Contig6_All, CL1484.Contig7_All, CL1493.Contig1_All, CL1795.Contig3_All, CL2340.Contig2_All, CL2372.Contig1_All, CL2372.Contig2_All, CL2372.Contig3_All, CL3669.Contig1_All, CL4073.Contig1_All, CL4073.Contig2_All, CL4237.Contig1_All, CL4849.Contig1_All, CL4849.Contig2_All, CL4849.Contig3_All, CL4925.Contig1_All, Unigene10181_All, Unigene10182_All, Unigene1056_All, Unigene1947_All, Unigene2243_All, Unigene3171_All, Unigene4187_All, Unigene4789_All, Unigene61_All, Unigene6289_All, Unigene7649_All, Unigene9245_All
196	Histidine metabolism	CL1736.Contig1_All, CL1736.Contig2_All, CL2309.Contig2_All, CL2309.Contig3_All, CL2377.Contig2_All, CL2694.Contig1_All, CL2694.Contig3_All, CL313.Contig2_All, CL4144.Contig3_All, CL4144.Contig4_All, CL4451.Contig1_All, CL4451.Contig2_All, CL4594.Contig1_All, CL4753.Contig1_All, Unigene10282_All, Unigene10299_All, Unigene10526_All, Unigene10686_All, Unigene10908_All, Unigene1197_All, Unigene13410_All, Unigene13773_All, Unigene15270_All, Unigene16206_All, Unigene17527_All, Unigene18101_All, Unigene18183_All, Unigene186_All, Unigene19038_All, Unigene3146_All, Unigene3450_All, Unigene5053_All, Unigene6360_All
197	Thiamine metabolism	CL4242.Contig1_All
198	NF-kappa B signaling pathway	CL1075.Contig2_All, CL1075.Contig3_All, CL131.Contig1_All, CL131.Contig2_All, CL188.Contig16_All, CL2182.Contig1_All, CL2182.Contig3_All, CL2182.Contig4_All, CL2306.Contig2_All, CL268.Contig2_All, CL3047.Contig1_All, CL3047.Contig2_All, CL39.Contig1_All, CL39.Contig2_All, CL39.Contig3_All, CL39.Contig4_All, CL3979.Contig1_All, CL3979.Contig2_All, CL4232.Contig1_All, CL4232.Contig2_All, CL4855.Contig1_All, CL4855.Contig2_All, CL5326.Contig17_All, CL5391.Contig1_All, CL5391.Contig2_All, CL940.Contig3_All, CL940.Contig5_All, Unigene10619_All, Unigene1247_All, Unigene12591_All, Unigene12868_All, Unigene13134_All, Unigene15257_All, Unigene1757_All, Unigene18898_All, Unigene20095_All, Unigene3131_All, Unigene5995_All, Unigene6911_All, Unigene7220_All, Unigene8056_All, Unigene8246_All, Unigene8988_All

199	RIG-I-like receptor signaling pathway	CL2306.Contig2_All, CL2654.Contig3_All, CL4017.Contig3_All, CL4966.Contig1_All, CL5115.Contig1_All, CL88.Contig2_All, CL88.Contig3_All, Unigene10069_All, Unigene10897_All, Unigene1247_All, Unigene13060_All, Unigene13252_All, Unigene1757_All, Unigene220_All, Unigene3131_All, Unigene5650_All, Unigene6973_All
200	Cyanoamino acid metabolism	CL143.Contig1_All, CL143.Contig2_All, CL1707.Contig1_All, CL317.Contig2_All, CL3643.Contig2_All, CL3643.Contig6_All, CL5197.Contig2_All, CL5288.Contig2_All, Unigene10732_All, Unigene1623_All, Unigene3134_All, Unigene5433_All, Unigene6134_All, Unigene848_All, Unigene9060_All
201	Acute myeloid leukemia	CL1066.Contig1_All, CL1066.Contig2_All, CL1066.Contig3_All, CL1387.Contig1_All, CL1387.Contig3_All, CL1648.Contig1_All, CL1807.Contig1_All, CL1807.Contig2_All, CL2038.Contig1_All, CL2038.Contig7_All, CL3632.Contig2_All, CL3632.Contig3_All, CL367.Contig1_All, CL367.Contig2_All, CL367.Contig3_All, CL3796.Contig2_All, CL3796.Contig3_All, CL4439.Contig2_All, CL4787.Contig1_All, CL695.Contig16_All, Unigene10001_All, Unigene10174_All, Unigene12671_All, Unigene1757_All, Unigene2749_All, Unigene2956_All, Unigene3168_All, Unigene6222_All, Unigene7745_All, Unigene777_All, Unigene8046_All, Unigene8747_All, Unigene9738_All
202	Alanine, aspartate and glutamate metabolism	CL1000.Contig2_All, CL1599.Contig1_All, CL1599.Contig2_All, CL3118.Contig1_All, CL3118.Contig2_All, CL3127.Contig1_All, CL3283.Contig1_All, CL5320.Contig2_All, CL567.Contig4_All, CL580.Contig2_All, CL580.Contig3_All, Unigene10168_All, Unigene10169_All, Unigene10204_All, Unigene10205_All, Unigene10412_All, Unigene10883_All, Unigene10884_All, Unigene10909_All, Unigene10910_All, Unigene11336_All, Unigene1145_All, Unigene11887_All, Unigene12466_All, Unigene12470_All, Unigene12475_All, Unigene12835_All, Unigene13121_All, Unigene13174_All, Unigene18234_All, Unigene3655_All, Unigene4139_All, Unigene66_All, Unigene6731_All, Unigene6749_All, Unigene848_All, Unigene9215_All
203	Regulation of autophagy	CL1273.Contig1_All, CL1273.Contig2_All, CL1579.Contig4_All, CL2321.Contig1_All, CL2321.Contig2_All, CL3112.Contig3_All, CL3990.Contig6_All, CL557.Contig1_All, Unigene1155_All, Unigene11856_All, Unigene13621_All, Unigene4159_All, Unigene7113_All
204	Ubiquinone and other terpenoid-quinone biosynthesis	CL1214.Contig2_All, CL2164.Contig1_All, CL2164.Contig2_All, CL238.Contig3_All, CL3127.Contig1_All, CL3599.Contig2_All, CL4176.Contig2_All, Unigene11217_All, Unigene11218_All, Unigene11226_All, Unigene11310_All, Unigene12424_All, Unigene14433_All, Unigene15509_All, Unigene15510_All, Unigene18265_All, Unigene4098_All, Unigene4197_All, Unigene4383_All, Unigene5996_All, Unigene6674_All
205	Drug metabolism - cytochrome P450	CL1176.Contig4_All, CL1584.Contig2_All, CL1711.Contig1_All, CL1711.Contig2_All, CL1711.Contig3_All, CL1879.Contig2_All, CL1879.Contig3_All, CL1879.Contig4_All, CL2308.Contig1_All, CL2370.Contig2_All, CL2370.Contig3_All, CL2370.Contig4_All, CL2411.Contig1_All, CL2456.Contig3_All, CL2532.Contig1_All, CL2595.Contig1_All, CL2707.Contig1_All, CL3078.Contig1_All, CL3511.Contig2_All, CL3618.Contig1_All, CL3654.Contig1_All, CL3654.Contig2_All, CL3784.Contig1_All, CL3788.Contig1_All, CL4110.Contig1_All, CL4110.Contig2_All, CL4451.Contig1_All, CL4451.Contig2_All, CL4453.Contig1_All, CL450.Contig10_All, CL490.Contig1_All, CL490.Contig2_All, CL490.Contig3_All, CL4969.Contig1_All, CL5198.Contig1_All, CL5198.Contig2_All, CL720.Contig1_All, CL720.Contig4_All, CL731.Contig1_All, CL744.Contig1_All, CL744.Contig2_All, CL949.Contig3_All, Unigene10381_All, Unigene1120_All, Unigene1134_All, Unigene11788_All, Unigene11916_All, Unigene11949_All, Unigene1197_All, Unigene12008_All, Unigene12272_All, Unigene12297_All, Unigene12509_All, Unigene12961_All, Unigene13410_All, Unigene13727_All, Unigene13773_All, Unigene1385_All, Unigene147_All, Unigene15435_All, Unigene15451_All, Unigene15946_All, Unigene16322_All, Unigene18028_All, Unigene18489_All, Unigene18597_All, Unigene19024_All, Unigene19310_All, Unigene19340_All, Unigene19987_All, Unigene2078_All, Unigene2107_All, Unigene2294_All, Unigene2312_All, Unigene2322_All, Unigene2660_All, Unigene2675_All, Unigene27_All, Unigene2914_All, Unigene2928_All, Unigene3012_All, Unigene3125_All, Unigene3132_All, Unigene3654_All, Unigene3689_All, Unigene3701_All, Unigene3771_All, Unigene3936_All, Unigene432_All, Unigene4342_All, Unigene4564_All, Unigene4626_All, Unigene4675_All, Unigene5053_All, Unigene5060_All, Unigene5180_All, Unigene526_All, Unigene5338_All, Unigene5600_All, Unigene5637_All, Unigene5751_All, Unigene5834_All, Unigene5870_All, Unigene6129_All, Unigene6360_All, Unigene6650_All, Unigene6673_All, Unigene7135_All, Unigene7548_All, Unigene7663_All, Unigene8049_All, Unigene8218_All, Unigene8997_All, Unigene908_All, Unigene9227_All, Unigene923_All, Unigene9298_All, Unigene9654_All
206	Valine, leucine and isoleucine degradation	CL1353.Contig1_All, CL1353.Contig3_All, CL1404.Contig2_All, CL2309.Contig2_All, CL2309.Contig3_All, CL2694.Contig1_All, CL2694.Contig3_All, CL3963.Contig1_All, CL4091.Contig1_All, CL4091.Contig2_All, CL4144.Contig3_All, CL4144.Contig4_All, CL4451.Contig1_All, CL4451.Contig2_All, CL4646.Contig2_All, CL4753.Contig1_All, Unigene10157_All, Unigene10158_All, Unigene10247_All, Unigene10248_All, Unigene10282_All, Unigene10287_All, Unigene10299_All, Unigene10311_All, Unigene10475_All, Unigene10476_All, Unigene10526_All, Unigene10686_All, Unigene10908_All, Unigene11003_All, Unigene11230_All, Unigene11231_All, Unigene11232_All, Unigene1139_All, Unigene11854_All, Unigene11887_All, Unigene1197_All, Unigene12169_All, Unigene12187_All, Unigene12211_All, Unigene12219_All, Unigene12227_All, Unigene12259_All, Unigene12293_All, Unigene12407_All, Unigene12412_All, Unigene12419_All, Unigene12422_All, Unigene12480_All, Unigene12732_All, Unigene12959_All, Unigene13080_All, Unigene13152_All, Unigene13410_All, Unigene13431_All, Unigene13445_All, Unigene13673_All, Unigene13773_All, Unigene14111_All, Unigene15270_All, Unigene15429_All, Unigene16206_All, Unigene17527_All, Unigene18101_All, Unigene18651_All, Unigene186_All, Unigene19038_All, Unigene2215_All, Unigene2853_All, Unigene2945_All, Unigene3383_All, Unigene3450_All, Unigene4853_All, Unigene5053_All, Unigene6360_All, Unigene7153_All, Unigene7814_All, Unigene7834_All, Unigene7886_All, Unigene9634_All, Unigene9773_All

207	Steroid hormone biosynthesis	CL1176.Contig4_All, CL1902.Contig2_All, CL1997.Contig2_All, CL2308.Contig1_All, CL2370.Contig2_All, CL2370.Contig3_All, CL2370.Contig4_All, CL2386.Contig4_All, CL3654.Contig1_All, CL3654.Contig2_All, CL3950.Contig2_All, CL4110.Contig1_All, CL4110.Contig2_All, CL4585.Contig1_All, CL490.Contig1_All, CL490.Contig2_All, CL490.Contig3_All, CL720.Contig1_All, CL720.Contig4_All, CL744.Contig1_All, CL744.Contig2_All, Unigene10147_All, Unigene1120_All, Unigene12297_All, Unigene12597_All, Unigene13537_All, Unigene147_All, Unigene15946_All, Unigene18028_All, Unigene18489_All, Unigene19987_All, Unigene2107_All, Unigene2312_All, Unigene2660_All, Unigene2675_All, Unigene3012_All, Unigene3125_All, Unigene3132_All, Unigene3701_All, Unigene3771_All, Unigene4011_All, Unigene4342_All, Unigene4564_All, Unigene4675_All, Unigene4739_All, Unigene526_All, Unigene5637_All, Unigene5751_All, Unigene6034_All, Unigene6129_All, Unigene6650_All, Unigene6673_All, Unigene6821_All, Unigene8049_All, Unigene8218_All, Unigene9227_All
208	D-Glutamine and D-glutamate metabolism	CL3118.Contig1_All, CL3118.Contig2_All, CL5320.Contig2_All, CL567.Contig4_All, Unigene9215_All
209	Autoimmune thyroid disease	Unigene20260_All, Unigene4489_All, Unigene5493_All, Unigene6916_All, Unigene833_All
210	Synthesis and degradation of ketone bodies	Unigene12219_All, Unigene13673_All, Unigene2945_All, Unigene9634_All
211	Glycosylphosphatidylinositol(GPI)-anchor biosynthesis	CL1.Contig1_All, CL2607.Contig1_All, CL2607.Contig2_All, CL3107.Contig1_All, CL396.Contig25_All, CL396.Contig26_All, CL396.Contig28_All, CL396.Contig29_All, CL396.Contig30_All, CL396.Contig31_All, CL396.Contig33_All, CL396.Contig34_All, CL396.Contig35_All, Unigene1773_All, Unigene4796_All
212	Glycine, serine and threonine metabolism	CL33.Contig1_All, CL3315.Contig3_All, CL3315.Contig4_All, CL3364.Contig2_All, CL348.Contig1_All, CL348.Contig2_All, CL3490.Contig1_All, CL3490.Contig2_All, CL4971.Contig2_All, CL580.Contig2_All, CL584.Contig10_All, CL584.Contig15_All, CL584.Contig17_All, CL584.Contig1_All, CL584.Contig3_All, CL584.Contig4_All, CL584.Contig6_All, CL584.Contig7_All, Unigene10190_All, Unigene10191_All, Unigene10306_All, Unigene10403_All, Unigene10404_All, Unigene10732_All, Unigene11180_All, Unigene11181_All, Unigene11182_All, Unigene12300_All, Unigene12732_All, Unigene13027_All, Unigene13041_All, Unigene13254_All, Unigene14042_All, Unigene18088_All, Unigene3482_All, Unigene4809_All, Unigene5691_All, Unigene5758_All, Unigene6074_All, Unigene799_All, Unigene9204_All
213	Citrate cycle (TCA cycle)	CL2294.Contig4_All, CL2430.Contig3_All, CL3808.Contig2_All, CL4130.Contig1_All, CL4130.Contig2_All, CL4534.Contig2_All, CL4534.Contig3_All, CL4570.Contig1_All, CL5078.Contig12_All, CL5078.Contig8_All, CL5301.Contig1_All, CL5301.Contig2_All, CL957.Contig6_All, Unigene10029_All, Unigene10098_All, Unigene10418_All, Unigene10505_All, Unigene10599_All, Unigene10818_All, Unigene10929_All, Unigene10954_All, Unigene11020_All, Unigene11028_All, Unigene11062_All, Unigene11186_All, Unigene11187_All, Unigene11188_All, Unigene11277_All, Unigene11278_All, Unigene11279_All, Unigene11838_All, Unigene11930_All, Unigene12004_All, Unigene12281_All, Unigene12286_All, Unigene12384_All, Unigene12397_All, Unigene12399_All, Unigene12421_All, Unigene12491_All, Unigene12502_All, Unigene12585_All, Unigene12587_All, Unigene12589_All, Unigene12688_All, Unigene12732_All, Unigene12800_All, Unigene12801_All, Unigene12869_All, Unigene13117_All, Unigene13177_All, Unigene13505_All, Unigene16508_All, Unigene17053_All, Unigene17410_All, Unigene17564_All, Unigene17971_All, Unigene18238_All, Unigene18312_All, Unigene18841_All, Unigene18842_All, Unigene1980_All, Unigene236_All, Unigene5554_All, Unigene6618_All, Unigene8563_All, Unigene999_All
214	Alcoholism	CL1066.Contig1_All, CL1066.Contig2_All, CL1286.Contig2_All, CL1452.Contig1_All, CL1525.Contig15_All, CL1525.Contig9_All, CL1563.Contig2_All, CL1675.Contig2_All, CL1807.Contig1_All, CL1807.Contig2_All, CL1823.Contig8_All, CL187.Contig3_All, CL1874.Contig2_All, CL2131.Contig4_All, CL2131.Contig5_All, CL2131.Contig6_All, CL2131.Contig8_All, CL2197.Contig3_All, CL244.Contig2_All, CL2468.Contig1_All, CL2468.Contig2_All, CL2762.Contig4_All, CL2901.Contig2_All, CL2901.Contig3_All, CL2901.Contig4_All, CL2901.Contig5_All, CL2901.Contig8_All, CL3032.Contig2_All, CL3215.Contig1_All, CL3215.Contig2_All, CL3215.Contig3_All, CL3286.Contig1_All, CL3667.Contig1_All, CL3667.Contig2_All, CL3667.Contig3_All, CL3689.Contig2_All, CL3920.Contig2_All, CL3948.Contig3_All, CL4002.Contig1_All, CL4004.Contig1_All, CL4004.Contig2_All, CL4395.Contig1_All, CL4418.Contig2_All, CL4439.Contig2_All, CL4509.Contig1_All, CL4509.Contig2_All, CL4559.Contig2_All, CL4559.Contig3_All, CL4594.Contig1_All, CL4787.Contig1_All, CL4890.Contig1_All, CL4923.Contig2_All, CL741.Contig2_All, CL772.Contig1_All, CL772.Contig2_All, CL838.Contig2_All, CL838.Contig3_All, CL870.Contig4_All, CL870.Contig8_All, CL870.Contig9_All, CL940.Contig3_All, CL940.Contig5_All, CL988.Contig1_All, CL988.Contig3_All, Unigene10006_All, Unigene10020_All, Unigene10045_All, Unigene10058_All, Unigene10174_All, Unigene10574_All, Unigene10831_All, Unigene11054_All, Unigene11396_All, Unigene1168_All, Unigene11913_All, Unigene12110_All, Unigene12228_All, Unigene12315_All, Unigene12671_All, Unigene12680_All, Unigene12719_All, Unigene13107_All, Unigene1417_All, Unigene1512_All, Unigene1663_All, Unigene16769_All, Unigene17185_All, Unigene1743_All, Unigene176_All, Unigene19148_All, Unigene1925_All, Unigene19400_All, Unigene1941_All, Unigene20090_All, Unigene2029_All, Unigene217_All, Unigene2193_All, Unigene2196_All, Unigene2233_All, Unigene2398_All, Unigene2760_All, Unigene3217_All, Unigene3779_All, Unigene3996_All, Unigene4013_All, Unigene4022_All, Unigene4112_All, Unigene4116_All, Unigene4126_All, Unigene4532_All, Unigene4780_All, Unigene4935_All, Unigene4970_All, Unigene5179_All, Unigene5521_All, Unigene5675_All, Unigene5869_All, Unigene6217_All, Unigene6223_All, Unigene661_All, Unigene6655_All, Unigene69_All, Unigene7016_All, Unigene706_All, Unigene7298_All, Unigene7702_All, Unigene7704_All, Unigene7759_All, Unigene8046_All, Unigene8210_All, Unigene8747_All, Unigene9733_All, Unigene9904_All
215	Phenylalanine, tyrosine and tryptophan biosynthesis	CL3127.Contig1_All, Unigene10773_All, Unigene11226_All, Unigene11336_All, Unigene12475_All, Unigene13121_All, Unigene15509_All, Unigene15510_All

216	Steroid biosynthesis	CL1004.Contig1_All, CL1580.Contig1_All, CL3897.Contig1_All, CL4485.Contig1_All, CL4485.Contig3_All, CL4602.Contig1_All, Unigene2740_All, Unigene4866_All, Unigene4873_All, Unigene5266_All, Unigene5713_All, Unigene666_All, Unigene8065_All
217	Tyrosine metabolism	CL137.Contig1_All, CL137.Contig2_All, CL1685.Contig2_All, CL1736.Contig1_All, CL1736.Contig2_All, CL1879.Contig2_All, CL1879.Contig3_All, CL1879.Contig4_All, CL2377.Contig2_All, CL252.Contig2_All, CL252.Contig3_All, CL2595.Contig1_All, CL3127.Contig1_All, CL3156.Contig1_All, CL3818.Contig1_All, CL4451.Contig1_All, CL4451.Contig2_All, CL4489.Contig1_All, CL4594.Contig1_All, CL5230.Contig2_All, CL5315.Contig1_All, Unigene10226_All, Unigene10747_All, Unigene11038_All, Unigene11039_All, Unigene11217_All, Unigene11218_All, Unigene11226_All, Unigene11336_All, Unigene11788_All, Unigene1197_All, Unigene12292_All, Unigene12475_All, Unigene12509_All, Unigene13121_All, Unigene13410_All, Unigene13727_All, Unigene13773_All, Unigene13880_All, Unigene14369_All, Unigene15509_All, Unigene15510_All, Unigene18183_All, Unigene18265_All, Unigene19024_All, Unigene19340_All, Unigene2322_All, Unigene2915_All, Unigene2928_All, Unigene3146_All, Unigene3417_All, Unigene417_All, Unigene4626_All, Unigene4901_All, Unigene5053_All, Unigene5180_All, Unigene5338_All, Unigene6060_All, Unigene6162_All, Unigene6360_All, Unigene6387_All, Unigene6916_All, Unigene8213_All, Unigene833_All, Unigene923_All
218	Metabolism of xenobiotics by cytochrome P450	CL1176.Contig4_All, CL1584.Contig2_All, CL1684.Contig1_All, CL1711.Contig1_All, CL1711.Contig2_All, CL1711.Contig3_All, CL1879.Contig2_All, CL1879.Contig3_All, CL1879.Contig4_All, CL2308.Contig1_All, CL2370.Contig2_All, CL2370.Contig3_All, CL2370.Contig4_All, CL2411.Contig1_All, CL2456.Contig3_All, CL2532.Contig1_All, CL2595.Contig1_All, CL2707.Contig1_All, CL2894.Contig1_All, CL2894.Contig2_All, CL2894.Contig3_All, CL3078.Contig1_All, CL3511.Contig2_All, CL3618.Contig1_All, CL3654.Contig1_All, CL3654.Contig2_All, CL3784.Contig1_All, CL3788.Contig1_All, CL4110.Contig1_All, CL4110.Contig2_All, CL4451.Contig1_All, CL4451.Contig2_All, CL4453.Contig1_All, CL450.Contig10_All, CL490.Contig1_All, CL490.Contig2_All, CL490.Contig3_All, CL4969.Contig1_All, CL720.Contig1_All, CL720.Contig4_All, CL731.Contig1_All, CL744.Contig1_All, CL744.Contig2_All, Unigene10381_All, Unigene1120_All, Unigene1134_All, Unigene11788_All, Unigene11916_All, Unigene11949_All, Unigene1197_All, Unigene12008_All, Unigene12297_All, Unigene12509_All, Unigene13410_All, Unigene13727_All, Unigene13773_All, Unigene1385_All, Unigene147_All, Unigene15435_All, Unigene15451_All, Unigene15946_All, Unigene16322_All, Unigene17598_All, Unigene18028_All, Unigene18489_All, Unigene18597_All, Unigene19024_All, Unigene19310_All, Unigene19340_All, Unigene19987_All, Unigene2078_All, Unigene2107_All, Unigene2312_All, Unigene2322_All, Unigene239_All, Unigene2660_All, Unigene2675_All, Unigene27_All, Unigene2914_All, Unigene2928_All, Unigene3012_All, Unigene3125_All, Unigene3132_All, Unigene3689_All, Unigene3701_All, Unigene3771_All, Unigene3936_All, Unigene432_All, Unigene4342_All, Unigene4564_All, Unigene4626_All, Unigene4675_All, Unigene4991_All, Unigene5053_All, Unigene5060_All, Unigene5180_All, Unigene526_All, Unigene5338_All, Unigene5600_All, Unigene5637_All, Unigene5751_All, Unigene5834_All, Unigene5870_All, Unigene6104_All, Unigene6129_All, Unigene6360_All, Unigene6650_All, Unigene6673_All, Unigene7135_All, Unigene7548_All, Unigene7663_All, Unigene8049_All, Unigene8218_All, Unigene8997_All, Unigene908_All, Unigene9227_All, Unigene923_All, Unigene9298_All, Unigene9950_All
219	B cell receptor signaling pathway	CL1066.Contig1_All, CL1066.Contig2_All, CL1066.Contig3_All, CL1535.Contig3_All, CL1618.Contig2_All, CL1807.Contig1_All, CL1807.Contig2_All, CL1831.Contig1_All, CL1831.Contig2_All, CL1843.Contig3_All, CL188.Contig16_All, CL1954.Contig1_All, CL1954.Contig2_All, CL2182.Contig1_All, CL2182.Contig3_All, CL2182.Contig4_All, CL3311.Contig1_All, CL3311.Contig2_All, CL367.Contig1_All, CL367.Contig2_All, CL367.Contig3_All, CL39.Contig1_All, CL39.Contig2_All, CL39.Contig3_All, CL39.Contig4_All, CL4190.Contig1_All, CL4439.Contig2_All, CL4521.Contig1_All, CL4521.Contig2_All, CL4521.Contig3_All, CL4521.Contig5_All, CL4597.Contig2_All, CL4736.Contig1_All, CL4736.Contig2_All, CL4787.Contig1_All, CL5064.Contig2_All, CL5391.Contig1_All, CL5391.Contig2_All, CL695.Contig16_All, CL940.Contig3_All, CL940.Contig5_All, Unigene10174_All, Unigene11922_All, Unigene12671_All, Unigene13853_All, Unigene1479_All, Unigene15257_All, Unigene1757_All, Unigene18898_All, Unigene236_All, Unigene247_All, Unigene2749_All, Unigene7297_All, Unigene8046_All, Unigene8217_All, Unigene8784_All
220	Renal cell carcinoma	CL1049.Contig4_All, CL1066.Contig1_All, CL1066.Contig2_All, CL1066.Contig3_All, CL1535.Contig3_All, CL1807.Contig1_All, CL1807.Contig2_All, CL2170.Contig2_All, CL2170.Contig5_All, CL2222.Contig9_All, CL2658.Contig1_All, CL2658.Contig2_All, CL271.Contig2_All, CL2722.Contig1_All, CL2793.Contig1_All, CL2793.Contig2_All, CL3162.Contig2_All, CL367.Contig1_All, CL367.Contig2_All, CL367.Contig3_All, CL4381.Contig1_All, CL4439.Contig2_All, CL4510.Contig1_All, CL4736.Contig1_All, CL4736.Contig2_All, CL4787.Contig1_All, CL493.Contig2_All, CL524.Contig10_All, CL524.Contig11_All, CL524.Contig12_All, CL524.Contig13_All, CL524.Contig14_All, CL524.Contig15_All, CL524.Contig16_All, CL524.Contig17_All, CL524.Contig18_All, CL524.Contig1_All, CL524.Contig3_All, CL524.Contig4_All, CL524.Contig5_All, CL524.Contig6_All, CL524.Contig9_All, CL5469.Contig2_All, CL614.Contig2_All, CL695.Contig16_All, CL903.Contig1_All, Unigene10174_All, Unigene10876_All, Unigene11020_All, Unigene12660_All, Unigene12671_All, Unigene13036_All, Unigene13505_All, Unigene13588_All, Unigene1479_All, Unigene1679_All, Unigene2749_All, Unigene54_All, Unigene5781_All, Unigene695_All, Unigene7777_All, Unigene8046_All, Unigene8277_All, Unigene8725_All, Unigene8747_All, Unigene9719_All
221	One carbon pool by folate	CL1855.Contig1_All, CL3650.Contig2_All, Unigene10732_All, Unigene10880_All, Unigene10997_All, Unigene11730_All, Unigene13418_All, Unigene3792_All, Unigene3818_All

222	Purine metabolism	CL1184.Contig1_All, CL1184.Contig2_All, CL1184.Contig3_All, CL1184.Contig4_All, CL1184.Contig5_All, CL1258.Contig1_All, CL1258.Contig2_All, CL126.Contig2_All, CL1305.Contig1_All, CL134.Contig1_All, CL1356.Contig1_All, CL1356.Contig2_All, CL1388.Contig1_All, CL1395.Contig2_All, CL1411.Contig1_All, CL1411.Contig8_All, CL1418.Contig6_All, CL1418.Contig8_All, CL1559.Contig1_All, CL1683.Contig2_All, CL1683.Contig7_All, CL1986.Contig1_All, CL2272.Contig1_All, CL2301.Contig2_All, CL2301.Contig4_All, CL2326.Contig1_All, CL2326.Contig2_All, CL2326.Contig3_All, CL2326.Contig4_All, CL258.Contig1_All, CL258.Contig2_All, CL260.Contig15_All, CL2604.Contig1_All, CL2702.Contig1_All, CL2702.Contig2_All, CL2770.Contig1_All, CL2770.Contig2_All, CL2805.Contig1_All, CL2949.Contig2_All, CL3028.Contig1_All, CL322.Contig4_All, CL322.Contig5_All, CL322.Contig6_All, CL3251.Contig1_All, CL3251.Contig2_All, CL3420.Contig1_All, CL3576.Contig1_All, CL3635.Contig3_All, CL3657.Contig3_All, CL3664.Contig1_All, CL3664.Contig2_All, CL372.Contig1_All, CL3792.Contig3_All, CL3836.Contig1_All, CL3836.Contig2_All, CL4005.Contig2_All, CL4005.Contig3_All, CL4180.Contig3_All, CL4262.Contig2_All, CL4278.Contig2_All, CL4346.Contig1_All, CL440.Contig3_All, CL440.Contig5_All, CL440.Contig6_All, CL4617.Contig2_All, CL4904.Contig1_All, CL4904.Contig2_All, CL5004.Contig1_All, CL52.Contig17_All, CL52.Contig28_All, CL52.Contig35_All, CL52.Contig3_All, CL52.Contig41_All, CL5203.Contig1_All, CL5203.Contig2_All, CL5461.Contig2_All, CL593.Contig1_All, CL598.Contig1_All, CL598.Contig2_All, CL63.Contig10_All, CL63.Contig9_All, CL648.Contig2_All, CL787.Contig5_All, CL893.Contig1_All, CL91.Contig1_All, CL911.Contig1_All, Unigene10230_All, Unigene10231_All, Unigene10436_All, Unigene10549_All, Unigene10735_All, Unigene10883_All, Unigene10884_All, Unigene1105_All, Unigene11601_All, Unigene11623_All, Unigene11741_All, Unigene11812_All, Unigene11967_All, Unigene11970_All, Unigene12017_All, Unigene1205_All, Unigene12060_All, Unigene12080_All, Unigene12096_All, Unigene12117_All, Unigene12268_All, Unigene12311_All, Unigene12365_All, Unigene12388_All, Unigene12479_All, Unigene12488_All, Unigene1248_All, Unigene12530_All, Unigene12567_All, Unigene12580_All, Unigene12623_All, Unigene12645_All, Unigene12655_All, Unigene1278_All, Unigene12887_All, Unigene13002_All, Unigene13088_All, Unigene13119_All, Unigene13284_All, Unigene143_All, Unigene1464_All, Unigene1505_All, Unigene15176_All, Unigene1570_All, Unigene15790_All, Unigene15827_All, Unigene16033_All, Unigene16134_All, Unigene16238_All, Unigene16824_All, Unigene16860_All, Unigene17253_All, Unigene17282_All, Unigene17292_All, Unigene17301_All, Unigene17448_All, Unigene17488_All, Unigene17890_All, Unigene18003_All, Unigene18004_All, Unigene18006_All, Unigene18037_All, Unigene18038_All, Unigene18127_All, Unigene18200_All, Unigene18663_All, Unigene18801_All, Unigene19291_All, Unigene19657_All, Unigene20198_All, Unigene20279_All, Unigene20289_All, Unigene2029_All, Unigene2037_All, Unigene21204_All, Unigene2131_All, Unigene2156_All, Unigene2451_All, Unigene245_All, Unigene24_All, Unigene2513_All, Unigene2686_All, Unigene2754_All, Unigene3081_All, Unigene3288_All, Unigene3390_All, Unigene33_All, Unigene3419_All, Unigene3659_All, Unigene3792_All, Unigene3890_All, Unigene4102_All, Unigene4221_All, Unigene4251_All, Unigene5063_All, Unigene5745_All, Unigene6374_All, Unigene7183_All, Unigene7192_All, Unigene7296_All, Unigene7750_All, Unigene7822_All, Unigene7869_All, Unigene7993_All, Unigene8008_All, Unigene8160_All, Unigene8228_All, Unigene8236_All, Unigene8558_All, Unigene9094_All, Unigene9107_All, Unigene9222_All, Unigene9285_All, Unigene9287_All, Unigene93_All, Unigene9758_All
223	Fatty acid metabolism	CL1353.Contig1_All, CL1353.Contig3_All, CL1650.Contig1_All, CL1711.Contig1_All, CL1711.Contig2_All, CL1711.Contig3_All, CL1711.Contig4_All, CL1879.Contig2_All, CL1879.Contig3_All, CL1879.Contig4_All, CL2309.Contig2_All, CL2309.Contig3_All, CL2532.Contig1_All, CL2532.Contig2_All, CL2590.Contig1_All, CL2590.Contig2_All, CL2595.Contig1_All, CL2694.Contig1_All, CL2694.Contig3_All, CL2707.Contig1_All, CL2943.Contig1_All, CL2943.Contig2_All, CL3511.Contig1_All, CL3511.Contig2_All, CL3536.Contig1_All, CL3536.Contig2_All, CL3536.Contig5_All, CL364.Contig1_All, CL364.Contig4_All, CL364.Contig7_All, CL3963.Contig1_All, CL4144.Contig3_All, CL4144.Contig4_All, CL4451.Contig1_All, CL4451.Contig2_All, CL4646.Contig2_All, CL4753.Contig1_All, CL507.Contig7_All, CL866.Contig1_All, CL866.Contig2_All, Unigene10157_All, Unigene10158_All, Unigene10282_All, Unigene10299_All, Unigene10311_All, Unigene10475_All, Unigene10476_All, Unigene10526_All, Unigene10686_All, Unigene10908_All, Unigene11230_All, Unigene11231_All, Unigene11232_All, Unigene1134_All, Unigene11788_All, Unigene1197_All, Unigene12187_All, Unigene12211_All, Unigene12219_All, Unigene12252_All, Unigene12419_All, Unigene12422_All, Unigene12480_All, Unigene12509_All, Unigene12937_All, Unigene13152_All, Unigene13410_All, Unigene13673_All, Unigene13727_All, Unigene13773_All, Unigene15270_All, Unigene15429_All, Unigene16206_All, Unigene16322_All, Unigene17527_All, Unigene18101_All, Unigene18521_All, Unigene18597_All, Unigene186_All, Unigene19024_All, Unigene19038_All, Unigene19340_All, Unigene20494_All, Unigene2078_All, Unigene2215_All, Unigene2322_All, Unigene2724_All, Unigene27_All, Unigene2853_All, Unigene2928_All, Unigene3383_All, Unigene3450_All, Unigene4626_All, Unigene4853_All, Unigene4884_All, Unigene5053_All, Unigene5060_All, Unigene5180_All, Unigene5338_All, Unigene5600_All, Unigene5908_All, Unigene6360_All, Unigene693_All, Unigene7046_All, Unigene7135_All, Unigene7153_All, Unigene7239_All, Unigene761_All, Unigene7663_All, Unigene7834_All, Unigene8012_All, Unigene818_All, Unigene826_All, Unigene923_All
224	Pentose and glucuronate interconversions	CL1176.Contig4_All, CL1604.Contig2_All, CL1684.Contig1_All, CL2145.Contig2_All, CL2308.Contig1_All, CL2309.Contig2_All, CL2309.Contig3_All, CL2370.Contig2_All, CL2370.Contig3_All, CL2370.Contig4_All, CL2471.Contig3_All, CL2557.Contig1_All, CL2846.Contig2_All, CL3654.Contig1_All, CL3654.Contig2_All, CL370.Contig1_All, CL4110.Contig1_All, CL4110.Contig2_All, CL443.Contig1_All, CL4451.Contig1_All, CL4451.Contig2_All, CL4753.Contig1_All, CL490.Contig1_All, CL490.Contig2_All, CL490.Contig3_All, CL720.Contig1_All, CL720.Contig4_All, CL744.Contig1_All, CL744.Contig2_All, Unigene10282_All, Unigene10686_All, Unigene10908_All, Unigene1120_All, Unigene1197_All, Unigene12144_All, Unigene12297_All, Unigene12955_All, Unigene13410_All, Unigene13557_All, Unigene13671_All, Unigene13773_All, Unigene147_All, Unigene15270_All, Unigene15946_All, Unigene15981_All, Unigene16206_All, Unigene17196_All, Unigene17527_All, Unigene17957_All, Unigene18028_All, Unigene18101_All, Unigene18489_All, Unigene19038_All, Unigene20226_All, Unigene20387_All, Unigene20815_All, Unigene2107_All, Unigene2312_All, Unigene2496_All, Unigene2660_All, Unigene3012_All, Unigene3125_All, Unigene3132_All, Unigene3450_All, Unigene3701_All, Unigene3771_All, Unigene4342_All, Unigene4564_All, Unigene4675_All, Unigene4991_All, Unigene5053_All, Unigene526_All, Unigene5429_All, Unigene5637_All, Unigene5751_All, Unigene6129_All, Unigene627_All, Unigene6360_All, Unigene6650_All, Unigene6673_All, Unigene6954_All, Unigene7271_All, Unigene8049_All, Unigene8218_All, Unigene8732_All, Unigene9227_All

225	Cell adhesion molecules (CAMs)	CL1192.Contig1_All, CL1192.Contig2_All, CL1192.Contig3_All, CL1367.Contig3_All, CL1367.Contig4_All, CL1531.Contig1_All, CL1531.Contig2_All, CL1531.Contig3_All, CL2328.Contig2_All, CL2331.Contig2_All, CL2331.Contig3_All, CL2478.Contig3_All, CL2533.Contig3_All, CL3236.Contig1_All, CL3810.Contig1_All, CL4009.Contig1_All, CL4009.Contig2_All, CL4030.Contig3_All, CL4030.Contig4_All, CL4535.Contig1_All, CL4597.Contig2_All, CL5125.Contig1_All, CL5400.Contig1_All, CL5400.Contig2_All, CL920.Contig13_All, CL920.Contig15_All, CL920.Contig1_All, Unigene11874_All, Unigene12314_All, Unigene12810_All, Unigene133_All, Unigene17327_All, Unigene1764_All, Unigene17842_All, Unigene18906_All, Unigene19424_All, Unigene2018_All, Unigene20260_All, Unigene20583_All, Unigene2980_All, Unigene3684_All, Unigene3945_All, Unigene4207_All, Unigene5076_All, Unigene6080_All, Unigene7011_All, Unigene7196_All, Unigene8217_All, Unigene8915_All, Unigene9200_All, Unigene9740_All
226	Proximal tubule bicarbonate reclamation	CL190.Contig1_All, CL2137.Contig1_All, CL2137.Contig2_All, CL2430.Contig3_All, CL2474.Contig2_All, CL2854.Contig1_All, CL3118.Contig1_All, CL3118.Contig2_All, CL3160.Contig2_All, CL3776.Contig2_All, CL41.Contig3_All, CL41.Contig4_All, CL41.Contig8_All, CL5301.Contig1_All, CL5301.Contig2_All, CL5320.Contig2_All, CL5440.Contig1_All, CL567.Contig4_All, CL974.Contig2_All, Unigene10029_All, Unigene10594_All, Unigene10926_All, Unigene11480_All, Unigene11482_All, Unigene11483_All, Unigene11934_All, Unigene16508_All, Unigene2002_All, Unigene2003_All, Unigene3010_All, Unigene4211_All, Unigene4323_All, Unigene5724_All, Unigene6215_All, Unigene6639_All, Unigene7061_All, Unigene7206_All, Unigene9163_All, Unigene9215_All, Unigene9668_All, Unigene9711_All
227	Glycosaminoglycan biosynthesis - keratan sulfate	CL2708.Contig2_All, Unigene16539_All, Unigene4758_All, Unigene5770_All, Unigene8059_All
228	Galactose metabolism	CL2471.Contig3_All, CL2557.Contig1_All, CL5115.Contig3_All, CL5403.Contig2_All, Unigene12731_All, Unigene13557_All, Unigene13671_All, Unigene1538_All, Unigene15960_All, Unigene15981_All, Unigene16824_All, Unigene17282_All, Unigene17957_All, Unigene18003_All, Unigene18004_All, Unigene20387_All, Unigene20815_All, Unigene20899_All, Unigene2496_All, Unigene5047_All, Unigene627_All, Unigene6526_All, Unigene6954_All, Unigene7271_All, Unigene7735_All, Unigene8139_All, Unigene8732_All
229	SNARE interactions in vesicular transport	CL2153.Contig3_All, CL3506.Contig1_All, CL3506.Contig2_All, CL3794.Contig1_All, CL42.Contig2_All, Unigene10078_All, Unigene11973_All, Unigene13396_All, Unigene14430_All, Unigene3968_All, Unigene6005_All, Unigene6662_All
230	Starch and sucrose metabolism	CL1176.Contig4_All, CL1388.Contig1_All, CL2145.Contig2_All, CL2308.Contig1_All, CL2370.Contig2_All, CL2370.Contig3_All, CL2370.Contig4_All, CL2471.Contig3_All, CL3450.Contig1_All, CL3450.Contig2_All, CL3450.Contig3_All, CL3654.Contig1_All, CL3654.Contig2_All, CL4110.Contig1_All, CL4110.Contig2_All, CL4346.Contig1_All, CL490.Contig1_All, CL490.Contig2_All, CL490.Contig3_All, CL5115.Contig3_All, CL5322.Contig2_All, CL720.Contig1_All, CL720.Contig4_All, CL744.Contig1_All, CL744.Contig2_All, Unigene10167_All, Unigene10188_All, Unigene1120_All, Unigene1207_All, Unigene12223_All, Unigene12295_All, Unigene12297_All, Unigene12955_All, Unigene13375_All, Unigene13557_All, Unigene147_All, Unigene15946_All, Unigene15960_All, Unigene15981_All, Unigene16591_All, Unigene16824_All, Unigene17282_All, Unigene17535_All, Unigene17536_All, Unigene18003_All, Unigene18004_All, Unigene18028_All, Unigene18489_All, Unigene18652_All, Unigene20289_All, Unigene20387_All, Unigene20899_All, Unigene2107_All, Unigene21129_All, Unigene2312_All, Unigene2660_All, Unigene3012_All, Unigene3125_All, Unigene3132_All, Unigene3701_All, Unigene3771_All, Unigene4342_All, Unigene4564_All, Unigene4675_All, Unigene5047_All, Unigene526_All, Unigene5429_All, Unigene5637_All, Unigene5751_All, Unigene6129_All, Unigene6650_All, Unigene6673_All, Unigene6718_All, Unigene8049_All, Unigene8218_All, Unigene9227_All, Unigene9765_All, Unigene9987_All
231	Retinol metabolism	CL1176.Contig4_All, CL1329.Contig1_All, CL1353.Contig2_All, CL1604.Contig2_All, CL1711.Contig1_All, CL1711.Contig2_All, CL1711.Contig3_All, CL1879.Contig2_All, CL1879.Contig3_All, CL1879.Contig4_All, CL1902.Contig2_All, CL1997.Contig2_All, CL2308.Contig1_All, CL2370.Contig2_All, CL2370.Contig3_All, CL2370.Contig4_All, CL2532.Contig1_All, CL2595.Contig1_All, CL2707.Contig1_All, CL2846.Contig2_All, CL3158.Contig2_All, CL3158.Contig3_All, CL3511.Contig2_All, CL356.Contig20_All, CL3581.Contig2_All, CL3654.Contig1_All, CL3654.Contig2_All, CL370.Contig1_All, CL3727.Contig1_All, CL4110.Contig1_All, CL4110.Contig2_All, CL4585.Contig1_All, CL4810.Contig2_All, CL490.Contig1_All, CL490.Contig2_All, CL490.Contig3_All, CL553.Contig2_All, CL720.Contig1_All, CL720.Contig4_All, CL731.Contig1_All, CL744.Contig1_All, CL744.Contig2_All, Unigene10147_All, Unigene10153_All, Unigene10482_All, Unigene1120_All, Unigene1134_All, Unigene11788_All, Unigene12114_All, Unigene12245_All, Unigene12297_All, Unigene12509_All, Unigene12597_All, Unigene12721_All, Unigene12899_All, Unigene13270_All, Unigene13537_All, Unigene13661_All, Unigene13727_All, Unigene147_All, Unigene15946_All, Unigene16322_All, Unigene18028_All, Unigene18489_All, Unigene18597_All, Unigene19024_All, Unigene19340_All, Unigene19987_All, Unigene2078_All, Unigene2107_All, Unigene2312_All, Unigene2322_All, Unigene2660_All, Unigene2675_All, Unigene27_All, Unigene2928_All, Unigene3012_All, Unigene3019_All, Unigene3125_All, Unigene3132_All, Unigene3701_All, Unigene3771_All, Unigene3888_All, Unigene4100_All, Unigene4169_All, Unigene432_All, Unigene4342_All, Unigene4564_All, Unigene4626_All, Unigene4675_All, Unigene5060_All, Unigene5180_All, Unigene526_All, Unigene5338_All, Unigene5600_All, Unigene5637_All, Unigene5751_All, Unigene5801_All, Unigene6129_All, Unigene6425_All, Unigene6650_All, Unigene6673_All, Unigene6821_All, Unigene6833_All, Unigene6843_All, Unigene7135_All, Unigene7273_All, Unigene7275_All, Unigene7663_All, Unigene8049_All, Unigene8218_All, Unigene9227_All, Unigene923_All, Unigene9825_All

232	Lysosome	CL1004.Contig1_All, CL106.Contig35_All, CL1098.Contig1_All, CL1166.Contig1_All, CL1166.Contig2_All, CL1210.Contig2_All, CL1225.Contig6_All, CL1257.Contig10_All, CL1257.Contig11_All, CL1257.Contig12_All, CL1257.Contig1_All, CL1257.Contig2_All, CL1257.Contig3_All, CL1257.Contig4_All, CL1257.Contig6_All, CL1257.Contig8_All, CL1300.Contig1_All, CL1300.Contig2_All, CL1300.Contig5_All, CL1300.Contig7_All, CL1323.Contig3_All, CL1341.Contig2_All, CL1358.Contig1_All, CL1358.Contig2_All, CL1358.Contig3_All, CL1382.Contig6_All, CL151.Contig1_All, CL151.Contig2_All, CL151.Contig4_All, CL1537.Contig1_All, CL1537.Contig2_All, CL1557.Contig1_All, CL1557.Contig2_All, CL1565.Contig1_All, CL1580.Contig1_All, CL1591.Contig2_All, CL161.Contig4_All, CL1614.Contig6_All, CL1724.Contig1_All, CL1750.Contig1_All, CL2011.Contig1_All, CL2019.Contig1_All, CL2019.Contig2_All, CL2028.Contig1_All, CL2028.Contig2_All, CL2037.Contig5_All, CL2140.Contig1_All, CL2150.Contig3_All, CL2171.Contig2_All, CL2240.Contig1_All, CL2267.Contig1_All, CL2277.Contig2_All, CL2277.Contig3_All, CL228.Contig11_All, CL228.Contig1_All, CL228.Contig6_All, CL2328.Contig1_All, CL2351.Contig1_All, CL2351.Contig2_All, CL2362.Contig1_All, CL2362.Contig2_All, CL247.Contig1_All, CL247.Contig2_All, CL2547.Contig2_All, CL2634.Contig1_All, CL2634.Contig2_All, CL2704.Contig1_All, CL3049.Contig2_All, CL3049.Contig3_All, CL3091.Contig1_All, CL3091.Contig2_All, CL3091.Contig3_All, CL3091.Contig4_All, CL3119.Contig1_All, CL3119.Contig2_All, CL3293.Contig1_All, CL3302.Contig2_All, CL3351.Contig2_All, CL3706.Contig1_All, CL3865.Contig2_All, CL388.Contig1_All, CL3884.Contig2_All, CL3897.Contig1_All, CL3965.Contig1_All, CL3965.Contig2_All, CL3965.Contig4_All, CL4033.Contig1_All, CL4057.Contig1_All, CL4090.Contig2_All, CL4259.Contig1_All, CL4259.Contig2_All, CL4272.Contig2_All, CL4276.Contig2_All, CL4294.Contig1_All, CL4294.Contig2_All, CL432.Contig3_All, CL432.Contig4_All, CL432.Contig6_All, CL4337.Contig3_All, CL4337.Contig6_All, CL4393.Contig1_All, CL4393.Contig2_All, CL4413.Contig2_All, CL4459.Contig1_All, CL4485.Contig1_All, CL4485.Contig3_All, CL4562.Contig2_All, CL4576.Contig1_All, CL4602.Contig1_All, CL4605.Contig1_All, CL4605.Contig2_All, CL4617.Contig2_All, CL4673.Contig1_All, CL4673.Contig2_All, CL4673.Contig4_All, CL484.Contig1_All, CL497.Contig1_All, CL497.Contig2_All, CL497.Contig3_All, CL5019.Contig2_All, CL504.Contig1_All, CL504.Contig2_All, CL5135.Contig1_All, CL5135.Contig2_All, CL5353.Contig1_All, CL5353.Contig2_All, CL5395.Contig1_All, CL5395.Contig2_All, CL5403.Contig2_All, CL5407.Contig1_All, CL5407.Contig2_All, CL548.Contig1_All, CL785.Contig1_All, CL829.Contig1_All, CL829.Contig2_All, CL862.Contig1_All, Unigene10007_All, Unigene10026_All, Unigene10129_All, Unigene10162_All, Unigene10170_All, Unigene10296_All, Unigene10452_All, Unigene10483_All, Unigene10507_All, Unigene1051_All, Unigene10709_All, Unigene10977_All, Unigene10982_All, Unigene10994_All, Unigene11166_All, Unigene11282_All, Unigene11360_All, Unigene11361_All, Unigene11488_All, Unigene11513_All, Unigene11553_All, Unigene11554_All, Unigene11633_All, Unigene11660_All, Unigene11863_All, Unigene11865_All, Unigene11876_All, Unigene11879_All, Unigene11891_All, Unigene11925_All, Unigene11971_All, Unigene12028_All, Unigene12148_All, Unigene12170_All, Unigene12172_All, Unigene12209_All, Unigene12302_All, Unigene12447_All, Unigene12455_All, Unigene12508_All, Unigene12634_All, Unigene12736_All, Unigene12840_All, Unigene12922_All, Unigene12923_All, Unigene12973_All, Unigene13219_All, Unigene13225_All, Unigene13324_All, Unigene13624_All, Unigene13978_All, Unigene15238_All, Unigene15239_All, Unigene15243_All, Unigene15494_All, Unigene15726_All, Unigene16264_All, Unigene16438_All, Unigene164_All, Unigene16993_All, Unigene17327_All, Unigene17368_All, Unigene1741_All, Unigene17470_All, Unigene17889_All, Unigene17953_All, Unigene18095_All, Unigene18114_All, Unigene18189_All, Unigene18288_All, Unigene18675_All, Unigene18707_All, Unigene1895_All, Unigene1913_All, Unigene1922_All, Unigene19672_All, Unigene2074_All, Unigene2096_All, Unigene2160_All, Unigene2270_All, Unigene2281_All, Unigene244_All, Unigene2699_All, Unigene2905_All, Unigene2959_All, Unigene2961_All, Unigene2982_All, Unigene3043_All, Unigene313_All, Unigene3157_All, Unigene3159_All, Unigene3297_All, Unigene3483_All, Unigene3651_All, Unigene3730_All, Unigene391_All, Unigene3982_All, Unigene4060_All, Unigene4103_All, Unigene4148_All, Unigene4155_All, Unigene4326_All, Unigene44_All, Unigene462_All, Unigene4637_All, Unigene4846_All, Unigene4866_All, Unigene4873_All, Unigene4921_All, Unigene5110_All, Unigene5111_All, Unigene5187_All, Unigene5193_All, Unigene5266_All, Unigene5429_All, Unigene55_All, Unigene5639_All, Unigene5646_All, Unigene5713_All, Unigene5716_All, Unigene5774_All, Unigene5890_All, Unigene5923_All, Unigene5935_All, Unigene6034_All, Unigene6173_All, Unigene6207_All, Unigene624_All, Unigene646_All, Unigene6692_All, Unigene6707_All, Unigene6915_All, Unigene7078_All, Unigene712_All, Unigene7134_All, Unigene7642_All, Unigene7665_All, Unigene7734_All, Unigene7751_All, Unigene7757_All, Unigene7873_All, Unigene7979_All, Unigene8011_All, Unigene8065_All, Unigene8079_All, Unigene8139_All, Unigene813_All, Unigene8167_All, Unigene8504_All, Unigene850_All, Unigene8540_All, Unigene860_All, Unigene8739_All, Unigene8973_All, Unigene9034_All, Unigene9058_All, Unigene9141_All, Unigene9251_All, Unigene925_All, Unigene9260_All, Unigene9314_All, Unigene962_All, Unigene9636_All, Unigene9928_All, Unigene9946_All
233	Fructose and mannose metabolism	CL1666.Contig1_All, CL1666.Contig2_All, CL2557.Contig1_All, CL2562.Contig2_All, CL280.Contig1_All, CL3128.Contig2_All, CL40.Contig10_All, CL40.Contig1_All, CL40.Contig2_All, CL40.Contig6_All, CL4358.Contig1_All, CL959.Contig1_All, Unigene10315_All, Unigene10364_All, Unigene10383_All, Unigene11782_All, Unigene11826_All, Unigene11830_All, Unigene11894_All, Unigene12066_All, Unigene12236_All, Unigene12245_All, Unigene12554_All, Unigene13470_All, Unigene13671_All, Unigene1538_All, Unigene15674_All, Unigene17838_All, Unigene17873_All, Unigene17957_All, Unigene18248_All, Unigene19184_All, Unigene19580_All, Unigene20815_All, Unigene2257_All, Unigene2496_All, Unigene3105_All, Unigene3281_All, Unigene4011_All, Unigene4267_All, Unigene4967_All, Unigene6258_All, Unigene627_All, Unigene6526_All, Unigene6913_All, Unigene6954_All, Unigene7271_All, Unigene7656_All, Unigene7735_All, Unigene8732_All, Unigene8994_All, Unigene9090_All, Unigene9826_All

234	Amino sugar and nucleotide sugar metabolism	CL1371.Contig2_All, CL1666.Contig1_All, CL1666.Contig2_All, CL2145.Contig2_All, CL2232.Contig2_All, CL2417.Contig1_All, CL2471.Contig3_All, CL2543.Contig2_All, CL2808.Contig1_All, CL3053.Contig1_All, CL3128.Contig2_All, CL3965.Contig1_All, CL3965.Contig2_All, CL3965.Contig4_All, Unigene10131_All, Unigene10204_All, Unigene10205_All, Unigene10535_All, Unigene11617_All, Unigene11944_All, Unigene11954_All, Unigene12327_All, Unigene12402_All, Unigene12466_All, Unigene12731_All, Unigene12955_All, Unigene13174_All, Unigene13375_All, Unigene13557_All, Unigene15981_All, Unigene16591_All, Unigene16824_All, Unigene17282_All, Unigene17953_All, Unigene18003_All, Unigene18004_All, Unigene18239_All, Unigene20387_All, Unigene20586_All, Unigene2202_All, Unigene2291_All, Unigene2709_All, Unigene4136_All, Unigene5131_All, Unigene5646_All, Unigene7656_All, Unigene7658_All, Unigene8039_All, Unigene8121_All, Unigene9022_All, Unigene9260_All, Unigene9314_All
235	Mismatch repair	CL120.Contig2_All, CL2986.Contig2_All, CL4989.Contig1_All, CL4989.Contig2_All, CL91.Contig1_All, Unigene10181_All, Unigene10182_All, Unigene10261_All, Unigene10838_All, Unigene12220_All, Unigene12361_All, Unigene18761_All, Unigene1947_All, Unigene2890_All, Unigene4102_All, Unigene7649_All, Unigene8228_All, Unigene8709_All
236	Sphingolipid metabolism	CL1300.Contig1_All, CL1300.Contig2_All, CL1300.Contig5_All, CL1300.Contig7_All, CL148.Contig2_All, CL148.Contig3_All, CL1670.Contig1_All, CL1724.Contig1_All, CL2259.Contig1_All, CL2280.Contig1_All, CL2525.Contig4_All, CL3292.Contig1_All, CL3292.Contig2_All, CL344.Contig1_All, CL4337.Contig3_All, CL4337.Contig6_All, CL4845.Contig1_All, CL4889.Contig2_All, CL5403.Contig2_All, CL885.Contig2_All, CL920.Contig13_All, CL920.Contig15_All, CL920.Contig1_All, Unigene10129_All, Unigene4342_All, Unigene5773_All, Unigene6034_All, Unigene692_All, Unigene7936_All, Unigene8139_All, Unigene8167_All
237	Glycosphingolipid biosynthesis - lacto and neolacto series	CL2562.Contig2_All, CL2708.Contig2_All, CL629.Contig1_All, CL629.Contig6_All, Unigene16539_All, Unigene2204_All, Unigene4758_All, Unigene5023_All, Unigene6258_All, Unigene6913_All, Unigene7057_All, Unigene8059_All, Unigene8994_All, Unigene9333_All, Unigene9826_All
238	Cysteine and methionine metabolism	CL1486.Contig3_All, CL2429.Contig1_All, CL2671.Contig2_All, CL305.Contig2_All, CL3127.Contig1_All, CL33.Contig1_All, CL4152.Contig2_All, CL5338.Contig2_All, CL57.Contig2_All, CL970.Contig2_All, Unigene10190_All, Unigene10191_All, Unigene11180_All, Unigene11181_All, Unigene11182_All, Unigene11226_All, Unigene11336_All, Unigene12140_All, Unigene12308_All, Unigene12475_All, Unigene12541_All, Unigene12544_All, Unigene12560_All, Unigene12760_All, Unigene12843_All, Unigene13121_All, Unigene14063_All, Unigene15509_All, Unigene15510_All, Unigene17244_All, Unigene18999_All, Unigene20111_All, Unigene20765_All, Unigene2208_All, Unigene2512_All, Unigene3252_All, Unigene5758_All, Unigene6046_All, Unigene6358_All, Unigene6831_All, Unigene6894_All, Unigene7717_All, Unigene9262_All, Unigene9716_All
239	Linoleic acid metabolism	CL1711.Contig3_All, CL199.Contig1_All, CL2432.Contig2_All, CL2590.Contig1_All, CL39.Contig1_All, CL39.Contig2_All, CL39.Contig3_All, CL39.Contig4_All, CL464.Contig8_All, CL5310.Contig2_All, CL731.Contig1_All, CL860.Contig10_All, CL860.Contig8_All, CL860.Contig9_All, Unigene10315_All, Unigene1134_All, Unigene12236_All, Unigene12245_All, Unigene12937_All, Unigene18521_All, Unigene19987_All, Unigene2078_All, Unigene2257_All, Unigene2675_All, Unigene2724_All, Unigene3029_All, Unigene3033_All, Unigene3034_All, Unigene3036_All, Unigene3037_All, Unigene3042_All, Unigene3281_All, Unigene4011_All, Unigene4884_All, Unigene4967_All, Unigene7135_All, Unigene7239_All, Unigene9090_All
240	Colorectal cancer	CL1066.Contig1_All, CL1066.Contig2_All, CL1066.Contig3_All, CL120.Contig2_All, CL1439.Contig1_All, CL1439.Contig2_All, CL1535.Contig3_All, CL1843.Contig3_All, CL2573.Contig2_All, CL2573.Contig3_All, CL2788.Contig2_All, CL2882.Contig1_All, CL2882.Contig2_All, CL367.Contig1_All, CL367.Contig2_All, CL367.Contig3_All, CL4271.Contig1_All, CL4439.Contig2_All, CL4736.Contig1_All, CL4736.Contig2_All, CL4787.Contig1_All, CL5115.Contig1_All, CL695.Contig16_All, Unigene11307_All, Unigene11724_All, Unigene11922_All, Unigene12188_All, Unigene12671_All, Unigene13476_All, Unigene1479_All, Unigene150_All, Unigene17702_All, Unigene2050_All, Unigene220_All, Unigene236_All, Unigene247_All, Unigene2749_All, Unigene4581_All, Unigene4801_All, Unigene5727_All, Unigene5808_All, Unigene7297_All, Unigene777_All, Unigene783_All, Unigene824_All, Unigene8747_All, Unigene8784_All, Unigene8972_All, Unigene9050_All, Unigene9877_All
241	Primary immunodeficiency	Unigene18195_All
242	Jak-STAT signaling pathway	CL1066.Contig1_All, CL1066.Contig2_All, CL1066.Contig3_All, CL1225.Contig6_All, CL1807.Contig1_All, CL1807.Contig2_All, CL1953.Contig1_All, CL2038.Contig1_All, CL2038.Contig7_All, CL2170.Contig2_All, CL2170.Contig5_All, CL2222.Contig9_All, CL3460.Contig2_All, CL3460.Contig3_All, CL3632.Contig2_All, CL3632.Contig3_All, CL367.Contig1_All, CL367.Contig2_All, CL367.Contig3_All, CL695.Contig16_All, Unigene10174_All, Unigene1279_All, Unigene2749_All, Unigene3168_All, Unigene54_All, Unigene5781_All, Unigene6020_All, Unigene6916_All, Unigene8046_All, Unigene833_All, Unigene9738_All
243	Phenylalanine metabolism	CL1707.Contig1_All, CL238.Contig3_All, CL3127.Contig1_All, CL3599.Contig2_All, CL3643.Contig2_All, CL3643.Contig6_All, CL4176.Contig2_All, CL4451.Contig1_All, CL4451.Contig2_All, CL4594.Contig1_All, CL5197.Contig2_All, CL5288.Contig2_All, Unigene10773_All, Unigene11217_All, Unigene11218_All, Unigene11226_All, Unigene11336_All, Unigene1197_All, Unigene12183_All, Unigene12292_All, Unigene12475_All, Unigene13121_All, Unigene13410_All, Unigene13773_All, Unigene15509_All, Unigene15510_All, Unigene1623_All, Unigene18265_All, Unigene3134_All, Unigene3417_All, Unigene4098_All, Unigene417_All, Unigene4197_All, Unigene5053_All, Unigene5433_All, Unigene5996_All, Unigene6134_All, Unigene6360_All, Unigene6387_All, Unigene6674_All, Unigene9060_All
244	Ether lipid metabolism	CL1624.Contig1_All, CL199.Contig1_All, CL2432.Contig2_All, CL2716.Contig2_All, CL3190.Contig2_All, CL344.Contig1_All, CL39.Contig1_All, CL39.Contig2_All, CL39.Contig3_All, CL39.Contig4_All, CL464.Contig8_All, CL4691.Contig2_All, CL4845.Contig1_All, CL5310.Contig2_All, CL860.Contig10_All, CL860.Contig8_All, CL860.Contig9_All, Unigene12214_All, Unigene12946_All, Unigene20289_All, Unigene2755_All, Unigene3029_All, Unigene3033_All, Unigene3034_All, Unigene3036_All, Unigene3037_All, Unigene3042_All, Unigene7018_All, Unigene7936_All

245	Arachidonic acid metabolism	CL143.Contig1_All, CL143.Contig2_All, CL1584.Contig2_All, CL1711.Contig3_All, CL199.Contig1_All, CL2411.Contig1_All, CL2432.Contig2_All, CL2456.Contig3_All, CL2590.Contig1_All, CL2814.Contig1_All, CL3074.Contig2_All, CL3078.Contig1_All, CL317.Contig2_All, CL3554.Contig1_All, CL3618.Contig1_All, CL364.Contig1_All, CL3788.Contig1_All, CL39.Contig1_All, CL39.Contig2_All, CL39.Contig3_All, CL39.Contig4_All, CL4453.Contig1_All, CL450.Contig10_All, CL464.Contig8_All, CL4969.Contig1_All, CL5310.Contig2_All, CL5352.Contig1_All, CL5352.Contig2_All, CL731.Contig1_All, CL860.Contig10_All, CL860.Contig8_All, CL860.Contig9_All, Unigene10157_All, Unigene10887_All, Unigene1134_All, Unigene12008_All, Unigene12937_All, Unigene1385_All, Unigene13883_All, Unigene15435_All, Unigene15451_All, Unigene17640_All, Unigene17929_All, Unigene18521_All, Unigene20494_All, Unigene2078_All, Unigene2724_All, Unigene27_All, Unigene2914_All, Unigene3029_All, Unigene3033_All, Unigene3034_All, Unigene3036_All, Unigene3037_All, Unigene3042_All, Unigene432_All, Unigene4884_All, Unigene5870_All, Unigene5908_All, Unigene6118_All, Unigene6858_All, Unigene7135_All, Unigene7239_All, Unigene7548_All, Unigene8997_All, Unigene908_All, Unigene9950_All
246	alpha-Linolenic acid metabolism	CL1353.Contig1_All, CL1353.Contig3_All, CL1580.Contig1_All, CL1939.Contig1_All, CL199.Contig1_All, CL2432.Contig2_All, CL2433.Contig10_All, CL39.Contig1_All, CL39.Contig2_All, CL39.Contig3_All, CL39.Contig4_All, CL4602.Contig1_All, CL464.Contig8_All, CL4646.Contig2_All, CL5264.Contig2_All, CL5310.Contig2_All, CL860.Contig10_All, CL860.Contig8_All, CL860.Contig9_All, Unigene10157_All, Unigene10158_All, Unigene10311_All, Unigene11839_All, Unigene11862_All, Unigene11975_All, Unigene3029_All, Unigene3033_All, Unigene3034_All, Unigene3036_All, Unigene3037_All, Unigene3042_All, Unigene5713_All
247	Vitamin digestion and absorption	CL3666.Contig1_All, CL3666.Contig2_All, CL3866.Contig2_All, CL464.Contig8_All, CL4692.Contig1_All, CL4692.Contig2_All, CL4720.Contig2_All, CL4980.Contig1_All, Unigene16595_All, Unigene17100_All, Unigene171_All, Unigene17447_All, Unigene184_All, Unigene2054_All, Unigene4219_All, Unigene442_All, Unigene6002_All, Unigene7779_All, Unigene8662_All, Unigene9229_All, Unigene9251_All
248	Other glycan degradation	CL106.Contig35_All, CL1382.Contig6_All, CL3049.Contig2_All, CL3049.Contig3_All, CL3965.Contig1_All, CL3965.Contig2_All, CL3965.Contig4_All, CL4270.Contig1_All, CL4270.Contig2_All, CL5403.Contig2_All, Unigene17953_All, Unigene5646_All, Unigene8167_All, Unigene850_All, Unigene918_All, Unigene9260_All, Unigene9314_All
249	Glycerolipid metabolism	CL1004.Contig1_All, CL127.Contig13_All, CL1580.Contig1_All, CL195.Contig2_All, CL2010.Contig1_All, CL2010.Contig2_All, CL2309.Contig2_All, CL2309.Contig3_All, CL2433.Contig10_All, CL2557.Contig1_All, CL2694.Contig1_All, CL2694.Contig3_All, CL344.Contig1_All, CL3463.Contig3_All, CL3578.Contig2_All, CL3578.Contig3_All, CL3578.Contig4_All, CL3897.Contig1_All, CL3897.Contig2_All, CL3966.Contig2_All, CL4144.Contig3_All, CL4144.Contig4_All, CL4451.Contig1_All, CL4451.Contig2_All, CL4485.Contig1_All, CL4485.Contig3_All, CL4602.Contig1_All, CL4753.Contig1_All, CL4845.Contig1_All, CL725.Contig1_All, CL88.Contig2_All, CL88.Contig3_All, Unigene10069_All, Unigene10282_All, Unigene10299_All, Unigene10526_All, Unigene10686_All, Unigene10908_All, Unigene1197_All, Unigene13410_All, Unigene13671_All, Unigene13773_All, Unigene15270_All, Unigene16206_All, Unigene16671_All, Unigene17527_All, Unigene17957_All, Unigene18101_All, Unigene18656_All, Unigene186_All, Unigene19038_All, Unigene20815_All, Unigene2496_All, Unigene3450_All, Unigene4873_All, Unigene5053_All, Unigene5266_All, Unigene5713_All, Unigene627_All, Unigene6360_All, Unigene6954_All, Unigene7271_All, Unigene7936_All, Unigene8065_All, Unigene8139_All, Unigene8719_All, Unigene8732_All, Unigene9188_All, Unigene9714_All
250	Bile secretion	CL1049.Contig4_All, CL1176.Contig4_All, CL1364.Contig4_All, CL1452.Contig1_All, CL1746.Contig1_All, CL1746.Contig2_All, CL190.Contig1_All, CL197.Contig1_All, CL197.Contig2_All, CL197.Contig3_All, CL2137.Contig1_All, CL2137.Contig2_All, CL2308.Contig1_All, CL2370.Contig2_All, CL2370.Contig3_All, CL2370.Contig4_All, CL2474.Contig2_All, CL2854.Contig1_All, CL2894.Contig1_All, CL2894.Contig2_All, CL2894.Contig3_All, CL3030.Contig1_All, CL3030.Contig3_All, CL3160.Contig2_All, CL363.Contig2_All, CL3654.Contig1_All, CL3654.Contig2_All, CL3776.Contig2_All, CL4005.Contig2_All, CL4005.Contig3_All, CL41.Contig3_All, CL41.Contig4_All, CL41.Contig8_All, CL4110.Contig1_All, CL4110.Contig2_All, CL420.Contig1_All, CL420.Contig3_All, CL4262.Contig2_All, CL4334.Contig1_All, CL4334.Contig2_All, CL440.Contig3_All, CL440.Contig5_All, CL440.Contig6_All, CL4510.Contig1_All, CL4559.Contig2_All, CL4559.Contig3_All, CL4621.Contig2_All, CL4773.Contig1_All, CL4773.Contig2_All, CL490.Contig1_All, CL490.Contig2_All, CL490.Contig3_All, CL493.Contig2_All, CL5204.Contig1_All, CL5204.Contig2_All, CL5362.Contig2_All, CL5440.Contig1_All, CL720.Contig1_All, CL720.Contig4_All, CL744.Contig1_All, CL744.Contig2_All, CL798.Contig1_All, CL798.Contig2_All, CL798.Contig3_All, CL880.Contig3_All, CL940.Contig3_All, CL940.Contig5_All, CL974.Contig2_All, Unigene10058_All, Unigene10594_All, Unigene1120_All, Unigene11480_All, Unigene11482_All, Unigene11483_All, Unigene1170_All, Unigene11915_All, Unigene12297_All, Unigene12660_All, Unigene147_All, Unigene15946_All, Unigene17598_All, Unigene18028_All, Unigene18195_All, Unigene18366_All, Unigene18489_All, Unigene18766_All, Unigene19987_All, Unigene2002_All, Unigene2003_All, Unigene2029_All, Unigene2054_All, Unigene2107_All, Unigene2312_All, Unigene239_All, Unigene2660_All, Unigene2675_All, Unigene3010_All, Unigene3012_All, Unigene3125_All, Unigene3132_All, Unigene3701_All, Unigene3771_All, Unigene3779_All, Unigene3824_All, Unigene4112_All, Unigene4211_All, Unigene4323_All, Unigene4342_All, Unigene4532_All, Unigene4537_All, Unigene4564_All, Unigene4675_All, Unigene4700_All, Unigene4_All, Unigene526_All, Unigene5637_All, Unigene5724_All, Unigene5751_All, Unigene5768_All, Unigene5_All, Unigene6104_All, Unigene6129_All, Unigene6215_All, Unigene6639_All, Unigene6650_All, Unigene6673_All, Unigene690_All, Unigene6936_All, Unigene695_All, Unigene6_All, Unigene7006_All, Unigene7061_All, Unigene7206_All, Unigene784_All, Unigene7_All, Unigene8049_All, Unigene8218_All, Unigene8464_All, Unigene8910_All, Unigene9025_All, Unigene9078_All, Unigene9163_All, Unigene9227_All, Unigene9251_All, Unigene93_All, Unigene9523_All, Unigene9668_All, Unigene9733_All

251	Adipocytokine signaling pathway	CL1009.Contig1_All, CL1049.Contig4_All, CL1091.Contig14_All, CL2222.Contig9_All, CL2430.Contig3_All, CL2843.Contig2_All, CL2943.Contig1_All, CL2943.Contig2_All, CL3047.Contig1_All, CL3047.Contig2_All, CL3112.Contig3_All, CL3536.Contig1_All, CL3536.Contig2_All, CL3536.Contig5_All, CL3796.Contig2_All, CL3796.Contig3_All, CL3832.Contig1_All, CL408.Contig1_All, CL408.Contig3_All, CL408.Contig4_All, CL4510.Contig1_All, CL4855.Contig1_All, CL4855.Contig2_All, CL493.Contig2_All, CL507.Contig7_All, CL5115.Contig1_All, CL5158.Contig1_All, CL5158.Contig2_All, CL5204.Contig1_All, CL5204.Contig2_All, CL866.Contig1_All, CL866.Contig2_All, CL880.Contig3_All, Unigene10029_All, Unigene12660_All, Unigene12680_All, Unigene14046_All, Unigene16942_All, Unigene1757_All, Unigene20691_All, Unigene220_All, Unigene2749_All, Unigene3282_All, Unigene3836_All, Unigene5722_All, Unigene693_All, Unigene695_All, Unigene7046_All, Unigene706_All, Unigene761_All, Unigene8056_All, Unigene818_All
252	Peroxisome	CL1300.Contig4_All, CL1300.Contig6_All, CL1305.Contig1_All, CL1353.Contig1_All, CL1353.Contig3_All, CL1391.Contig2_All, CL1604.Contig2_All, CL1732.Contig1_All, CL1732.Contig2_All, CL1842.Contig1_All, CL1842.Contig3_All, CL2246.Contig3_All, CL2246.Contig6_All, CL2246.Contig8_All, CL257.Contig5_All, CL2846.Contig2_All, CL2943.Contig1_All, CL2943.Contig2_All, CL3114.Contig2_All, CL3158.Contig2_All, CL3158.Contig3_All, CL3212.Contig1_All, CL3315.Contig3_All, CL3315.Contig4_All, CL348.Contig1_All, CL348.Contig2_All, CL3536.Contig1_All, CL3536.Contig2_All, CL3536.Contig5_All, CL3581.Contig2_All, CL370.Contig1_All, CL3784.Contig1_All, CL3991.Contig1_All, CL4171.Contig1_All, CL4427.Contig2_All, CL4427.Contig3_All, CL4585.Contig1_All, CL46.Contig10_All, CL4646.Contig2_All, CL4686.Contig2_All, CL4700.Contig1_All, CL472.Contig5_All, CL507.Contig7_All, CL5341.Contig3_All, CL5411.Contig2_All, CL553.Contig2_All, CL580.Contig2_All, CL580.Contig3_All, CL584.Contig10_All, CL584.Contig15_All, CL584.Contig17_All, CL584.Contig1_All, CL584.Contig3_All, CL584.Contig4_All, CL584.Contig6_All, CL584.Contig7_All, CL818.Contig1_All, CL818.Contig2_All, CL866.Contig1_All, CL866.Contig2_All, Unigene10009_All, Unigene10157_All, Unigene10158_All, Unigene10311_All, Unigene10375_All, Unigene10418_All, Unigene10948_All, Unigene11412_All, Unigene11577_All, Unigene11816_All, Unigene11874_All, Unigene11969_All, Unigene12069_All, Unigene12175_All, Unigene12186_All, Unigene12214_All, Unigene12281_All, Unigene12359_All, Unigene12738_All, Unigene12763_All, Unigene12804_All, Unigene1280_All, Unigene12910_All, Unigene12946_All, Unigene13254_All, Unigene13270_All, Unigene13271_All, Unigene19880_All, Unigene20696_All, Unigene2945_All, Unigene3019_All, Unigene3305_All, Unigene3482_All, Unigene3689_All, Unigene3888_All, Unigene3936_All, Unigene4007_All, Unigene4100_All, Unigene5281_All, Unigene5834_All, Unigene6425_All, Unigene6518_All, Unigene6821_All, Unigene6833_All, Unigene6843_All, Unigene693_All, Unigene7046_All, Unigene7062_All, Unigene7273_All, Unigene7275_All, Unigene761_All, Unigene765_All, Unigene7989_All, Unigene818_All, Unigene826_All, Unigene8531_All, Unigene8704_All, Unigene8821_All, Unigene9724_All

	CL1.Contig1_All, CL1000.Contig2_All, CL1004.Contig1_All, CL1070.Contig6_All, CL1070.Contig7_All, CL1135.Contig2_All, CL1146.Contig2_All, CL1146.Contig3_All, CL1156.Contig3_All, CL1175.Contig1_All, CL1175.Contig2_All, CL1176.Contig4_All, CL1214.Contig2_All, CL1258.Contig1_All, CL1258.Contig2_All, CL1260.Contig1_All, CL127.Contig13_All, CL1300.Contig1_All, CL1300.Contig2_All, CL1300.Contig4_All, CL1300.Contig5_All, CL1300.Contig6_All, CL1300.Contig7_All, CL1305.Contig1_All, CL1313.Contig4_All, CL1333.Contig1_All, CL1333.Contig2_All, CL134.Contig1_All, CL1353.Contig1_All, CL1353.Contig3_All, CL1356.Contig1_All, CL1356.Contig2_All, CL137.Contig1_All, CL137.Contig2_All, CL1371.Contig2_All, CL1388.Contig1_All, CL1395.Contig2_All, CL1404.Contig2_All, CL1411.Contig1_All, CL1411.Contig8_All, CL1418.Contig6_All, CL1418.Contig8_All, CL143.Contig1_All, CL143.Contig2_All, CL1447.Contig2_All, CL1478.Contig1_All, CL1478.Contig2_All, CL148.Contig2_All, CL148.Contig3_All, CL1484.Contig6_All, CL1484.Contig7_All, CL1486.Contig3_All, CL15.Contig1_All, CL15.Contig2_All, CL15.Contig3_All, CL1508.Contig1_All, CL1508.Contig2_All, CL151.Contig1_All, CL151.Contig2_All, CL151.Contig4_All, CL1513.Contig1_All, CL1521.Contig1_All, CL1521.Contig2_All, CL1521.Contig3_All, CL1521.Contig5_All, CL1531.Contig1_All, CL1531.Contig2_All, CL1531.Contig3_All, CL1580.Contig1_All, CL1584.Contig2_All, CL1594.Contig1_All, CL1599.Contig1_All, CL1599.Contig2_All, CL1604.Contig2_All, CL1624.Contig1_All, CL1666.Contig1_All, CL1666.Contig2_All, CL1670.Contig1_All, CL1683.Contig2_All, CL1683.Contig7_All, CL1685.Contig2_All, CL1711.Contig1_All, CL1711.Contig2_All, CL1711.Contig3_All, CL1724.Contig1_All, CL1737.Contig1_All, CL1737.Contig2_All, CL1780.Contig1_All, CL1780.Contig2_All, CL1798.Contig1_All, CL1798.Contig2_All, CL1838.Contig1_All, CL1842.Contig1_All, CL1842.Contig3_All, CL1855.Contig1_All, CL1868.Contig1_All, CL1868.Contig2_All, CL1868.Contig4_All, CL1876.Contig1_All, CL1879.Contig2_All, CL1879.Contig3_All, CL1879.Contig4_All, CL1892.Contig1_All, CL1892.Contig2_All, CL1892.Contig3_All, CL1892.Contig5_All, CL1902.Contig2_All, CL195.Contig2_All, CL1961.Contig2_All, CL199.Contig1_All, CL1990.Contig1_All, CL1990.Contig2_All, CL1995.Contig1_All, CL1995.Contig2_All, CL1995.Contig3_All, CL1997.Contig2_All, CL2010.Contig1_All, CL2010.Contig2_All, CL2017.Contig4_All, CL2017.Contig5_All, CL2035.Contig1_All, CL2035.Contig4_All, CL2053.Contig1_All, CL2053.Contig2_All, CL2128.Contig1_All, CL2145.Contig2_All, CL2150.Contig3_All, CL2164.Contig1_All, CL2164.Contig2_All, CL2220.Contig1_All, CL2220.Contig2_All, CL2246.Contig3_All, CL2246.Contig6_All, CL2246.Contig8_All, CL2259.Contig1_All, CL2272.Contig1_All, CL2280.Contig1_All, CL2294.Contig4_All, CL2305.Contig1_All, CL2305.Contig3_All, CL2305.Contig5_All, CL2308.Contig1_All, CL2309.Contig2_All, CL2309.Contig3_All, CL2326.Contig1_All, CL2326.Contig2_All, CL2326.Contig3_All, CL2326.Contig4_All, CL2370.Contig2_All, CL2370.Contig3_All, CL2370.Contig4_All, CL238.Contig3_All, CL2386.Contig4_All, CL2401.Contig1_All, CL2406.Contig3_All, CL2411.Contig1_All, CL2417.Contig1_All, CL2430.Contig3_All, CL2432.Contig2_All, CL2433.Contig10_All, CL2445.Contig1_All, CL2456.Contig3_All, CL2471.Contig3_All, CL2512.Contig2_All, CL252.Contig2_All, CL252.Contig3_All, CL2525.Contig4_All, CL2532.Contig1_All, CL2533.Contig3_All, CL2551.Contig1_All, CL2557.Contig1_All, CL2562.Contig2_All, CL257.Contig5_All, CL258.Contig1_All, CL258.Contig2_All, CL2590.Contig1_All, CL2595.Contig1_All, CL2604.Contig1_All, CL2607.Contig1_All, CL2607.Contig2_All, CL2624.Contig4_All, CL2626.Contig1_All, CL2634.Contig1_All, CL2634.Contig2_All, CL2648.Contig3_All, CL2671.Contig2_All, CL2678.Contig1_All, CL2678.Contig2_All, CL2694.Contig1_All, CL2694.Contig3_All, CL27.Contig5_All, CL2702.Contig1_All, CL2702.Contig2_All, CL2707.Contig1_All, CL2708.Contig2_All, CL2716.Contig2_All, CL280.Contig1_All, CL2814.Contig1_All, CL2835.Contig1_All, CL2835.Contig2_All, CL2835.Contig3_All, CL2835.Contig4_All, CL2846.Contig2_All, CL2898.Contig1_All, CL2898.Contig2_All, CL2898.Contig3_All, CL2930.Contig5_All, CL2941.Contig1_All, CL2943.Contig1_All, CL2943.Contig2_All, CL2944.Contig1_All, CL2944.Contig2_All, CL2951.Contig1_All, CL2951.Contig4_All, CL2951.Contig7_All, CL2983.Contig1_All, CL300.Contig7_All, CL3006.Contig2_All, CL3019.Contig2_All, CL3019.Contig3_All, CL3019.Contig4_All, CL3028.Contig1_All, CL3040.Contig1_All, CL3074.Contig2_All, CL3078.Contig1_All, CL309.Contig2_All, CL309.Contig3_All, CL309.Contig4_All, CL3091.Contig1_All, CL3091.Contig2_All, CL3091.Contig3_All, CL3091.Contig4_All, CL3107.Contig1_All, CL3118.Contig1_All, CL3118.Contig2_All, CL3127.Contig1_All, CL3128.Contig2_All, CL3129.Contig1_All, CL3129.Contig2_All, CL3129.Contig5_All, CL313.Contig2_All, CL3135.Contig1_All, CL3135.Contig2_All, CL3135.Contig3_All, CL3142.Contig1_All, CL3142.Contig2_All, CL3156.Contig1_All, CL3158.Contig2_All, CL3158.Contig3_All, CL3163.Contig2_All, CL317.Contig2_All, CL3212.Contig1_All, CL3228.Contig1_All, CL3235.Contig1_All, CL3235.Contig2_All, CL3251.Contig1_All, CL3251.Contig2_All, CL3283.Contig1_All, CL3292.Contig1_All, CL3292.Contig2_All, CL33.Contig1_All, CL3314.Contig2_All, CL3314.Contig4_All, CL3315.Contig3_All, CL3315.Contig4_All, CL3364.Contig2_All, CL3410.Contig1_All, CL3410.Contig2_All, CL3420.Contig1_All, CL344.Contig1_All, CL3453.Contig4_All, CL3463.Contig3_All, CL348.Contig1_All, CL348.Contig2_All, CL3497.Contig1_All, CL3505.Contig2_All, CL3511.Contig2_All, CL3536.Contig1_All, CL3536.Contig2_All, CL3536.Contig5_All, CL3554.Contig1_All, CL3576.Contig1_All, CL3578.Contig2_All, CL3578.Contig3_All, CL3578.Contig4_All, CL3581.Contig2_All, CL3599.Contig2_All, CL3614.Contig1_All, CL3614.Contig2_All, CL3618.Contig1_All, CL364.Contig1_All, CL3650.Contig2_All, CL3654.Contig1_All, CL3654.Contig2_All, CL3657.Contig1_All, CL3657.Contig3_All, CL3664.Contig1_All, CL3664.Contig2_All, CL3687.Contig1_All, CL3687.Contig2_All, CL370.Contig1_All, CL372.Contig1_All, CL3727.Contig1_All, CL3730.Contig1_All, CL3756.Contig1_All, CL3756.Contig2_All, CL3788.Contig1_All, CL3792.Contig3_All, CL3808.Contig2_All, CL3832.Contig1_All, CL3878.Contig2_All, CL3884.Contig2_All, CL3897.Contig1_All, CL3897.Contig2_All, CL39.Contig1_All, CL39.Contig2_All, CL39.Contig3_All, CL39.Contig4_All, CL3945.Contig4_All, CL3945.Contig6_All, CL3945.Contig8_All, CL396.Contig25_All, CL396.Contig26_All, CL396.Contig28_All, CL396.Contig29_All, CL396.Contig30_All, CL396.Contig31_All, CL396.Contig33_All, CL396.Contig34_All, CL396.Contig35_All, CL3963.Contig1_All, CL3965.Contig1_All, CL3965.Contig2_All, CL3965.Contig4_All, CL3966.Contig2_All, CL3979.Contig1_All, CL3979.Contig2_All, CL3991.Contig1_All, CL3994.Contig1_All, CL4030.Contig3_All, CL4030.Contig4_All, CL4090.Contig2_All, CL4091.Contig1_All, CL4091.Contig2_All, CL4110.Contig1_All, CL4110.Contig2_All, CL4130.Contig1_All, CL4130.Contig2_All, CL4132.Contig2_All, CL4144.Contig3_All, CL4144.Contig4_All, CL4152.Contig2_All, CL4171.Contig1_All, CL4176.Contig2_All, CL4195.Contig2_All, CL4221.Contig1_All, CL424.Contig1_All, CL424.Contig2_All, CL4256.Contig1_All, CL4278.Contig2_All, CL4304.Contig1_All, CL4304.Contig2_All, CL4337.Contig3_All, CL4337.Contig6_All, CL4346.Contig1_All, CL4355.Contig1_All, CL4355.Contig2_All, CL4358.Contig1_All, CL4365.Contig1_All, CL4427.Contig2_All, CL4427.Contig3_All, CL443.Contig1_All, CL4446.Contig1_All, CL4446.Contig2_All, CL4451.Contig1_All, CL4451.Contig2_All, CL4453.Contig1_All, CL4459.Contig1_All, CL4460.Contig1_All, CL4482.Contig2_All, CL4485.Contig1_All, CL4485.Contig3_All, CL450.Contig10_All, CL4527.Contig1_All, CL4527.Contig2_All, CL4534.Contig2_All, CL4534.Contig3_All, CL4570.Contig1_All, CL4585.Contig1_All, CL4594.Contig1_All, CL4602.Contig1_All, CL464.Contig8_All, CL4646.Contig2_All, CL4659.Contig1_All, CL4663.Contig1_All, CL4673.Contig1_All, CL4673.Contig2_All, CL4673.Contig4_All, CL4686.Contig2_All, CL4691.Contig2_All, CL4700.Contig1_All, CL4753.Contig1_All, CL4773.Contig1_All, CL4773.Contig2_All, CL4790.Contig1_All, CL4790.Contig2_All, CL4845.Contig1_All, CL485.Contig1_All, CL485.Contig2_All, CL4850.Contig1_All, CL4850.Contig3_All, CL4872.Contig1_All, CL4872.Contig2_All, CL4873.Contig2_All, CL4881.Contig2_All, CL4883.Contig1_All, CL4883.Contig2_All, CL4889.Contig2_All, CL490.Contig1_All, CL490.Contig2_All, CL490.Contig3_All, CL4903.Contig1_All, CL4969.Contig1_All, CL4971.Contig2_All, CL5012.Contig1_All, CL5027.Contig2_All, CL507.Contig7_All, CL5078.Contig12_All, CL5078.Contig8_All, CL5081.Contig2_All, CL5083.Contig2_All, CL5085.Contig2_All, CL5115.Contig3_All, CL5118.Contig2_All, CL52.Contig11_All, CL52.Contig15_All, CL52.Contig24_All, CL52.Contig2_All, CL52.Contig5_All, CL5230.Contig2_All, CL5266.Contig1_All, CL5266.Contig2_All, CL527.Contig2_All,

254	Glycerophospholipid metabolism	CL1260.Contig1_All, CL127.Contig13_All, CL1545.Contig1_All, CL1580.Contig1_All, CL195.Contig2_All, CL199.Contig1_All, CL2010.Contig1_All, CL2010.Contig2_All, CL2432.Contig2_All, CL2433.Contig10_All, CL2951.Contig1_All, CL2951.Contig4_All, CL2951.Contig7_All, CL3314.Contig2_All, CL3314.Contig4_All, CL344.Contig1_All, CL3463.Contig3_All, CL3578.Contig2_All, CL3578.Contig3_All, CL3578.Contig4_All, CL3818.Contig1_All, CL39.Contig1_All, CL39.Contig2_All, CL39.Contig3_All, CL39.Contig4_All, CL3966.Contig2_All, CL4197.Contig1_All, CL4197.Contig2_All, CL4489.Contig1_All, CL46.Contig10_All, CL4602.Contig1_All, CL464.Contig8_All, CL4691.Contig2_All, CL4845.Contig1_All, CL5122.Contig1_All, CL5122.Contig2_All, CL5125.Contig1_All, CL5291.Contig1_All, CL5310.Contig2_All, CL5315.Contig1_All, CL608.Contig2_All, CL608.Contig3_All, CL725.Contig1_All, CL860.Contig10_All, CL860.Contig8_All, CL860.Contig9_All, Unigene10633_All, Unigene11326_All, Unigene11327_All, Unigene11397_All, Unigene12266_All, Unigene12719_All, Unigene1290_All, Unigene12945_All, Unigene12985_All, Unigene13320_All, Unigene14369_All, Unigene18833_All, Unigene2072_All, Unigene2199_All, Unigene3029_All, Unigene3033_All, Unigene3034_All, Unigene3036_All, Unigene3037_All, Unigene3042_All, Unigene3362_All, Unigene4096_All, Unigene4741_All, Unigene5713_All, Unigene6060_All, Unigene7018_All, Unigene7023_All, Unigene7897_All, Unigene7936_All, Unigene8643_All, Unigene8727_All, Unigene9188_All, Unigene9644_All

Table S6. KEGG pathway enrichment analysis of differentially expressed proteins in high virulence (Bm5) and low virulence (Bm7) *Bursaphelenchus mucronarus* isolates.

#	Pathway	Sample1 (1118)	Sample2 (3776)	*p*-Value	Pathway ID
1	Metabolic pathways	444	1119	2.96×10^{-18}	ko01100
2	Valine, leucine and isoleucine degradation	61	111	1.38×10^{-8}	ko00280
3	Glycolysis/Gluconeogenesis	67	134	3.60×10^{-7}	ko00010
4	Fatty acid metabolism	67	136	7.27×10^{-7}	ko00071
5	Pyruvate metabolism	51	98	2.05×10^{-6}	ko00620
6	Citrate cycle (TCA cycle)	42	76	2.17×10^{-6}	ko00020
7	beta-Alanine metabolism	38	70	1.17×10^{-6}	ko00410
8	Aminoacyl-tRNA biosynthesis	26	42	1.28×10^{-5}	ko00970
9	Oxidative phosphorylation	71	160	3.71×10^{-5}	ko00190
10	Propanoate metabolism	42	84	5.73×10^{-5}	ko00640
11	Parkinson's disease	63	150	6.58×10^{-4}	ko05012
12	Peroxisome	71	174	8.22×10^{-4}	ko04146
13	Alzheimer's disease	67	167	1.93×10^{-3}	ko05010
14	Butanoate metabolism	28	58	1.94×10^{-3}	ko00650
15	Retinol metabolism	48	113	2.12×10^{-3}	ko00830
16	Tryptophan metabolism	41	94	2.44×10^{-3}	ko00380
17	Galactose metabolism	21	41	2.85×10^{-3}	ko00052
18	Huntington's disease	72	184	2.89×10^{-3}	ko05016
19	Arginine and proline metabolism	40	92	2.94×10^{-3}	ko00330
20	Glyoxylate and dicarboxylate metabolism	23	47	3.88×10^{-3}	ko00630
21	PPAR signaling pathway	29	63	3.98×10^{-3}	ko03320
22	Metabolism of xenobiotics by cytochrome P450	48	116	4.01×10^{-3}	ko00980
23	Pentose phosphate pathway	23	48	5.41×10^{-3}	ko00030
24	Drug metabolism—cytochrome P450	44	107	6.58×10^{-3}	ko00982
25	Lysine degradation	27	60	7.82×10^{-3}	ko00310
26	Biosynthesis of unsaturated fatty acids	17	34	9.56×10^{-3}	ko01040
27	Glutathione metabolism	40	98	1.07×10^{-2}	ko00480
28	Phenylalanine metabolism	26	59	1.23×10^{-2}	ko00360
29	Glycerolipid metabolism	26	59	1.23×10^{-2}	ko00561
30	Starch and sucrose metabolism	22	49	1.60×10^{-2}	ko00500
31	Synthesis and degradation of ketone bodies	7	11	1.99×10^{-2}	ko00072
32	Alanine, aspartate and glutamate metabolism	20	45	2.39×10^{-2}	ko00250
33	Tyrosine metabolism	27	65	2.58×10^{-2}	ko00350
34	Mismatch repair	4	5	2.92×10^{-2}	ko03430
35	Pentose and glucuronate interconversions	33	83	2.92×10^{-2}	ko00040
36	Arachidonic acid metabolism	30	75	3.36×10^{-2}	ko00590
37	Lysosome	47	128	4.70×10^{-2}	ko04142
38	alpha-Linolenic acid metabolism	11	23	4.94×10^{-2}	ko00592
39	Fatty acid elongation	15	37	1.01×10^{-1}	ko00062
40	Rheumatoid arthritis	15	37	1.01×10^{-1}	ko05323
41	ECM-receptor interaction	17	43	1.04×10^{-1}	ko04512
42	Porphyrin and chlorophyll metabolism	10	23	1.11×10^{-1}	ko00860
43	DNA replication	4	7	1.20×10^{-1}	ko03030
44	Fructose and mannose metabolism	24	65	1.22×10^{-1}	ko00051
45	Glycine, serine and threonine metabolism	15	38	1.24×10^{-1}	ko00260
46	Histidine metabolism	14	36	1.48×10^{-1}	ko00340
47	Ascorbate and aldarate metabolism	16	42	1.48×10^{-1}	ko00053
48	Terpenoid backbone biosynthesis	7	16	1.65×10^{-1}	ko00900
49	Cyanoamino acid metabolism	8	19	1.71×10^{-1}	ko00460
50	Antigen processing and presentation	16	43	1.75×10^{-1}	ko04612
51	Cysteine and methionine metabolism	13	35	2.10×10^{-1}	ko00270
52	Small cell lung cancer	13	35	2.10×10^{-1}	ko05222
53	Fat digestion and absorption	8	20	2.15×10^{-1}	ko04975
54	Tuberculosis	20	57	2.19×10^{-1}	ko05152
55	Cardiac muscle contraction	36	108	2.23×10^{-1}	ko04260
56	Riboflavin metabolism	3	6	2.48×10^{-1}	ko00740
57	Homologous recombination	3	6	2.48×10^{-1}	ko03440
58	Phenylalanine, tyrosine and tryptophan biosynthesis	3	6	2.48×10^{-1}	ko00400
59	Polyketide sugar unit biosynthesis	1	1	2.96×10^{-1}	ko00523

60	Collecting duct acid secretion	10	28	3.00×10^{-1}	ko04966
61	Sphingolipid metabolism	8	22	3.13×10^{-1}	ko00600
62	Renin-angiotensin system	13	38	3.21×10^{-1}	ko04614
63	Steroid hormone biosynthesis	12	35	3.28×10^{-1}	ko00140
64	Nicotinate and nicotinamide metabolism	5	13	3.33×10^{-1}	ko00760
65	Neuroactive ligand-receptor interaction	2	4	3.41×10^{-1}	ko04080
66	D-Arginine and D-ornithine metabolism	2	4	3.41×10^{-1}	ko00472
67	Selenocompound metabolism	3	7	3.43×10^{-1}	ko00450
68	Toxoplasmosis	18	55	3.52×10^{-1}	ko05145
69	Synaptic vesicle cycle	17	52	3.61×10^{-1}	ko04721
70	Drug metabolism—other enzymes	20	63	4.00×10^{-1}	ko00983
71	Pyrimidine metabolism	13	40	4.01×10^{-1}	ko00240
72	Fatty acid biosynthesis	4	11	4.18×10^{-1}	ko00061
73	Valine, leucine and isoleucine biosynthesis	4	11	4.18×10^{-1}	ko00290
74	Prostate cancer	18	57	4.20×10^{-1}	ko05215
75	Phagosome	32	104	4.33×10^{-1}	ko04145
76	Amino sugar and nucleotide sugar metabolism	13	41	4.41×10^{-1}	ko00520
77	Linoleic acid metabolism	9	28	4.54×10^{-1}	ko00591
78	Other glycan degradation	5	15	4.71×10^{-1}	ko00511
79	One carbon pool by folate	5	15	4.71×10^{-1}	ko00670
80	Protein digestion and absorption	15	49	4.92×10^{-1}	ko04974
81	Dorso-ventral axis formation	4	12	4.95×10^{-1}	ko04320
82	Lysine biosynthesis	1	2	5.04×10^{-1}	ko00300
83	Staphylococcus aureus infection	1	2	5.04×10^{-1}	ko05150
84	Epithelial cell signaling in Helicobacter pylori infection	11	36	5.13×10^{-1}	ko05120
85	Amoebiasis	15	50	5.29×10^{-1}	ko05146
86	Protein processing in endoplasmic reticulum	41	140	5.66×10^{-1}	ko04141
87	N-Glycan biosynthesis	4	13	5.67×10^{-1}	ko00510
88	Other types of O-glycan biosynthesis	4	13	5.67×10^{-1}	ko00514
89	Taurine and hypotaurine metabolism	2	6	5.71×10^{-1}	ko00430
90	Nucleotide excision repair	2	6	5.71×10^{-1}	ko03420
91	Steroid biosynthesis	2	6	5.71×10^{-1}	ko00100
92	Folate biosynthesis	2	6	5.714×10^{-1}	ko00790
93	Adipocytokine signaling pathway	10	34	5.74×10^{-1}	ko04920
94	Pathways in cancer	35	120	5.77×10^{-1}	ko05200
95	Dilated cardiomyopathy	37	127	5.81×10^{-1}	ko05414
96	Hypertrophic cardiomyopathy (HCM)	37	127	5.81×10^{-1}	ko05410
97	Arrhythmogenic right ventricular cardiomyopathy (ARVC)	14	48	5.81×10^{-1}	ko05412
98	Regulation of actin cytoskeleton	39	134	5.84×10^{-1}	ko04810
99	Mineral absorption	7	24	5.95×10^{-1}	ko04978
100	Hematopoietic cell lineage	9	31	5.95×10^{-1}	ko04640
101	Proximal tubule bicarbonate reclamation	9	31	5.95×10^{-1}	ko04964
102	Primary bile acid biosynthesis	8	28	6.18×10^{-1}	ko00120
103	Glycosphingolipid biosynthesis - ganglio series	1	3	6.51×10^{-1}	ko00604
104	Vitamin B6 metabolism	1	3	6.51×10^{-1}	ko00750
105	Inositol phosphate metabolism	5	18	6.54×10^{-1}	ko00562
106	Type I diabetes mellitus	2	7	6.62×10^{-1}	ko04940
107	Cell adhesion molecules (CAMs)	6	22	6.72×10^{-1}	ko04514
108	Viral myocarditis	22	79	6.76×10^{-1}	ko05416
109	NOD-like receptor signaling pathway	3	11	6.77×10^{-1}	ko04621
110	Systemic lupus erythematosus	4	15	6.92×10^{-1}	ko05322
111	Retrograde endocannabinoid signaling	10	37	6.94×10^{-1}	ko04723
112	Vibrio cholerae infection	16	59	7.09×10^{-1}	ko05110
113	Sulfur metabolism	2	8	7.37×10^{-1}	ko00920
114	Pantothenate and CoA biosynthesis	3	12	7.38×10^{-1}	ko00770
115	Ether lipid metabolism	3	12	7.38×10^{-1}	ko00565
116	Focal adhesion	33	121	7.47×10^{-1}	ko04510
117	Purine metabolism	22	82	7.48×10^{-1}	ko00230
118	Glycosaminoglycan degradation	1	4	7.54×10^{-1}	ko00531
119	Autoimmune thyroid disease	1	4	7.54×10^{-1}	ko05320
120	Caffeine metabolism	1	4	7.54×10^{-1}	ko00232

121	Ubiquinone and other terpenoid-quinone biosynthesis	7	28	7.67×10^{-1}	ko00130
122	Shigellosis	12	47	7.78×10^{-1}	ko05131
123	RNA degradation	12	47	7.78×10^{-1}	ko03018
124	Bile secretion	13	51	7.86×10^{-1}	ko04976
125	Type II diabetes mellitus	3	13	7.89×10^{-1}	ko04930
126	Ribosome	48	177	7.95×10^{-1}	ko03010
127	Leishmaniasis	2	9	7.97×10^{-1}	ko05140
128	p53 signaling pathway	2	9	7.97×10^{-1}	ko04115
129	Endocytosis	23	89	8.16×10^{-1}	ko04144
130	Pertussis	4	18	8.27×10^{-1}	ko05133
131	Malaria	1	5	8.27×10^{-1}	ko05144
132	D-Glutamine and D-glutamate metabolism	1	5	8.27×10^{-1}	ko00471
133	Sulfur relay system	1	5	8.27×10^{-1}	ko04122
134	Glycosphingolipid biosynthesis - globo series	1	5	8.27×10^{-1}	ko00603
135	Prion diseases	6	26	8.27×10^{-1}	ko05020
136	Bacterial invasion of epithelial cells	13	53	8.33×10^{-1}	ko05100
137	Proteasome	13	54	8.53×10^{-1}	ko03050
138	Carbohydrate digestion and absorption	5	23	8.56×10^{-1}	ko04973
139	Base excision repair	1	6	8.78×10^{-1}	ko03410
140	Mucin type O-Glycan biosynthesis	1	6	8.78×10^{-1}	ko00512
141	Amyotrophic lateral sclerosis (ALS)	9	40	8.79×10^{-1}	ko05014
142	Salmonella infection	25	101	8.85×10^{-1}	ko05132
143	Insulin signaling pathway	15	65	9.05×10^{-1}	ko04910
144	Aldosterone-regulated sodium reabsorption	4	21	9.08×10^{-1}	ko04960
145	mRNA surveillance pathway	12	54	9.14×10^{-1}	ko03015
146	Vitamin digestion and absorption	1	7	9.14×10^{-1}	ko04977
147	Bladder cancer	1	7	9.14×10^{-1}	ko05219
148	Protein export	5	26	9.21×10^{-1}	ko03060
149	RNA transport	28	117	9.31×10^{-1}	ko03013
150	Calcium signaling pathway	17	77	9.47×10^{-1}	ko04020
151	Ribosome biogenesis in eukaryotes	9	45	9.48×10^{-1}	ko03008
152	Transcriptional misregulation in cancer	3	19	9.50×10^{-1}	ko05202
153	Apoptosis	3	19	9.50×10^{-1}	ko04210
154	Legionellosis	7	37	9.51×10^{-1}	ko05134
155	RIG-I-like receptor signaling pathway	1	9	9.57×10^{-1}	ko04622
156	Taste transduction	1	9	9.57×10^{-1}	ko04742
157	Glycerophospholipid metabolism	7	38	9.60×10^{-1}	ko00564
158	Fc gamma R-mediated phagocytosis	9	47	9.63×10^{-1}	ko04666
159	Herpes simplex infection	8	44	9.71×10^{-1}	ko05168
160	mTOR signaling pathway	2	16	9.72×10^{-1}	ko04150
161	Measles	5	31	9.73×10^{-1}	ko05162
162	Vascular smooth muscle contraction	21	99	9.78×10^{-1}	ko04270
163	Melanoma	1	11	9.79×10^{-1}	ko05218
164	HTLV-I infection	13	67	9.79×10^{-1}	ko05166
165	Endocrine and other factor-regulated calcium reabsorption	8	46	9.81×10^{-1}	ko04961
166	Gastric acid secretion	11	59	9.81×10^{-1}	ko04971
167	Vasopressin-regulated water reabsorption	3	23	9.82×10^{-1}	ko04962
168	Leukocyte transendothelial migration	9	51	9.83×10^{-1}	ko04670
169	Renal cell carcinoma	2	18	9.84×10^{-1}	ko05211
170	Pancreatic cancer	2	18	9.84×10^{-1}	ko05212
171	Thyroid cancer	1	12	9.85×10^{-1}	ko05216
172	ABC transporters	3	24	9.87×10^{-1}	ko02010
173	Serotonergic synapse	6	39	9.87×10^{-1}	ko04726
174	Toll-like receptor signaling pathway	1	13	9.89×10^{-1}	ko04620
175	GABAergic synapse	4	30	9.89×10^{-1}	ko04727
176	Cell cycle	4	30	9.89×10^{-1}	ko04110
177	NF-kappa B signaling pathway	1	14	9.92×10^{-1}	ko04064
178	Jak-STAT signaling pathway	1	14	9.92×10^{-1}	ko04630
179	VEGF signaling pathway	4	32	9.93×10^{-1}	ko04370
180	Phototransduction - fly	4	32	9.93×10^{-1}	ko04745
181	Influenza A	13	74	9.94×10^{-1}	ko05164
182	Alcoholism	5	38	9.95×10^{-1}	ko05034
183	Progesterone-mediated oocyte maturation	2	22	9.95×10^{-1}	ko04914

184	Chronic myeloid leukemia	1	16	9.96×10^{-1}	ko05220
185	Spliceosome	22	115	9.96×10^{-1}	ko03040
186	Adherens junction	5	40	9.97×10^{-1}	ko04520
187	Tight junction	24	125	9.97×10^{-1}	ko04530
188	MAPK signaling pathway	12	74	9.97×10^{-1}	ko04010
189	Endometrial cancer	1	18	9.98×10^{-1}	ko05213
190	Colorectal cancer	1	18	9.98×10^{-1}	ko05210
191	Pathogenic Escherichia coli infection	11	71	9.98×10^{-1}	ko05130
192	Amphetamine addiction	2	26	9.98×10^{-1}	ko05031
193	Oocyte meiosis	4	38	9.98×10^{-1}	ko04114
194	Epstein-Barr virus infection	12	78	9.98×10^{-1}	ko05169
195	Non-small cell lung cancer	1	20	9.99×10^{-1}	ko05223
196	Pancreatic secretion	6	51	9.99×10^{-1}	ko04972
197	Morphine addiction	1	21	9.99×10^{-1}	ko05032
198	Glioma	1	21	9.99×10^{-1}	ko05214
199	Axon guidance	3	35	9.99×10^{-1}	ko04360
200	Fc epsilon RI signaling pathway	2	29	9.99×10^{-1}	ko04664
201	Hepatitis C	1	22	9.99×10^{-1}	ko05160
202	Salivary secretion	4	42	9.99×10^{-1}	ko04970
203	Ubiquitin mediated proteolysis	6	53	9.99×10^{-1}	ko04120
204	ErbB signaling pathway	2	30	9.99×10^{-1}	ko04012
205	Long-term potentiation	2	30	9.99×10^{-1}	ko04720
206	Chagas disease (American trypanosomiasis)	1	23	9.99×10^{-1}	ko05142
207	Natural killer cell mediated cytotoxicity	1	24	9.99×10^{-1}	ko04650
208	Phosphatidylinositol signaling system	1	24	9.99×10^{-1}	ko04070
209	Wnt signaling pathway	3	39	9.99×10^{-1}	ko04310
210	Neurotrophin signaling pathway	2	34	9.99×10^{-1}	ko04722
211	T cell receptor signaling pathway	2	37	9.99×10^{-1}	ko04660
212	GnRH signaling pathway	2	38	9.99×10^{-1}	ko04912
213	Gap junction	3	46	9.99×10^{-1}	ko04540
214	Chemokine signaling pathway	3	47	9.99×10^{-1}	ko04062
215	Dopaminergic synapse	3	48	9.99×10^{-1}	ko04728
216	Glutamatergic synapse	2	42	9.99×10^{-1}	ko04724
217	Cholinergic synapse	1	35	9.99×10^{-1}	ko04725

#	Pathway	Proteins
1	Metabolic pathways (no map in kegg database)	CL3577.Contig2_All, CL2780.Contig3_All, CL9784.Contig1_All, CL1623.Contig1_All, Unigene1665_All, Unigene4206_All, CL1181.Contig3_All, CL5661.Contig1_All, CL7964.Contig2_All, CL9723.Contig2_All, CL5576.Contig2_All, CL3543.Contig2_All, Unigene8070_All, Unigene22279_All, CL6939.Contig2_All, CL4988.Contig1_All, CL2664.Contig1_All, CL9384.Contig2_All, CL3932.Contig2_All, CL2312.Contig1_All, CL7841.Contig1_All, CL7944.Contig3_All, CL7107.Contig2_All, CL5383.Contig2_All, CL9064.Contig1_All, CL1521.Contig2_All, CL7130.Contig1_All, CL2464.Contig3_All, CL3798.Contig1_All, CL573.Contig1_All, Unigene7838_All, Unigene2609_All, CL3108.Contig1_All, Unigene27409_All, CL1012.Contig10_All, CL2660.Contig1_All, Unigene28027_All, CL2717.Contig2_All, Unigene118_All, CL2330.Contig1_All, CL860.Contig3_All, CL3815.Contig1_All, CL6328.Contig3_All, CL8564.Contig2_All, Unigene26224_All, CL4004.Contig3_All, Unigene21846_All, CL8538.Contig1_All, Unigene24033_All, Unigene11200_All, CL3833.Contig2_All, CL9241.Contig2_All, CL9975.Contig1_All, CL4391.Contig2_All, CL731.Contig1_All, Unigene3299_All, Unigene28419_All, Unigene22338_All, CL573.Contig2_All, CL5977.Contig2_All, CL7504.Contig1_All, Unigene7583_All, CL9768.Contig1_All, CL3063.Contig1_All, CL7557.Contig4_All, CL7104.Contig1_All, CL6788.Contig1_All, CL9609.Contig1_All, Unigene22665_All, CL3037.Contig5_All, Unigene6389_All, Unigene104_All, Unigene3398_All, CL5374.Contig3_All, CL1868.Contig3_All, CL200.Contig3_All, Unigene6758_All, CL9025.Contig2_All, CL5398.Contig1_All, CL837.Contig2_All, CL6788.Contig2_All, Unigene1879_All, CL846.Contig2_All, CL5897.Contig1_All, CL7766.Contig1_All, CL9394.Contig2_All, CL4031.Contig2_All, CL554.Contig1_All, CL8398.Contig1_All, CL4516.Contig1_All,

	Unigene28933_All, CL7504.Contig2_All, CL9087.Contig1_All, CL3305.Contig4_All, CL3204.Contig1_All, CL3677.Contig3_All, CL7647.Contig1_All, CL9117.Contig2_All, CL7976.Contig1_All, Unigene6334_All, CL3136.Contig3_All, Unigene7473_All, CL1079.Contig1_All, CL2270.Contig1_All, CL3311.Contig1_All, CL5005.Contig5_All, CL6614.Contig2_All, CL7505.Contig2_All, Unigene1464_All, CL3579.Contig2_All, Unigene2532_All, CL4435.Contig1_All, CL6584.Contig1_All, CL7213.Contig1_All, CL9626.Contig1_All, CL8398.Contig3_All, CL9475.Contig2_All, CL9241.Contig1_All, Unigene144_All, CL1341.Contig2_All, CL9413.Contig1_All, CL5462.Contig1_All, Unigene21985_All, Unigene3204_All, CL9247.Contig2_All, CL846.Contig4_All, CL3677.Contig1_All, CL6130.Contig1_All, CL7291.Contig1_All, CL9544.Contig2_All, Unigene22956_All, CL3687.Contig3_All, CL6343.Contig2_All, CL9412.Contig2_All, Unigene708_All, CL9955.Contig1_All, Unigene2447_All, CL3414.Contig2_All, CL5525.Contig1_All, CL8363.Contig1_All, Unigene27153_All, CL1452.Contig2_All, Unigene28629_All, Unigene5129_All, CL9406.Contig1_All, CL5782.Contig2_All, Unigene6068_All, CL9862.Contig1_All, CL3469.Contig5_All, CL8146.Contig4_All, CL5436.Contig1_All, CL9247.Contig1_All, CL551.Contig34_All, CL3185.Contig1_All, CL3037.Contig1_All, CL6249.Contig2_All, CL3033.Contig1_All, CL8538.Contig2_All, Unigene29448_All, Unigene7840_All, CL1054.Contig1_All, CL9379.Contig2_All, CL6506.Contig1_All, CL8471.Contig1_All, Unigene29100_All, CL6973.Contig1_All, CL3888.Contig1_All, CL3010.Contig1_All, CL3108.Contig2_All, CL2825.Contig2_All, CL3414.Contig5_All, CL5352.Contig2_All, Unigene1640_All, Unigene6769_All, CL5436.Contig6_All, CL6224.Contig2_All, Unigene8619_All, Unigene6644_All, Unigene21637_All, CL6328.Contig1_All, Unigene26422_All, Unigene1599_All, Unigene29335_All, CL1803.Contig1_All, Unigene6820_All, CL5782.Contig1_All, CL5142.Contig1_All, CL8564.Contig1_All, CL1060.Contig3_All, CL3893.Contig4_All, CL4939.Contig1_All, Unigene29299_All, Unigene27273_All, Unigene3119_All, CL7557.Contig1_All, Unigene3256_All, CL7557.Contig2_All, CL9739.Contig2_All, CL986.Contig2_All, CL4126.Contig1_All, CL953.Contig1_All, Unigene27764_All, CL4013.Contig1_All, CL152.Contig2_All, Unigene26750_All, CL2476.Contig2_All, Unigene3371_All, CL1530.Contig2_All, CL3070.Contig4_All, CL1452.Contig1_All, Unigene6213_All, Unigene1853_All, CL2170.Contig4_All, CL7008.Contig2_All, Unigene27103_All, CL2981.Contig1_All, Unigene4883_All, Unigene26386_All, CL5369.Contig1_All, CL837.Contig1_All, CL2764.Contig2_All, Unigene28320_All, CL4316.Contig2_All, Unigene8408_All, CL1151.Contig3_All, CL234.Contig2_All, CL2200.Contig1_All, CL4988.Contig3_All, Unigene4625_All, CL3242.Contig2_All, Unigene11218_All, CL3414.Contig4_All, CL959.Contig2_All, Unigene2480_All, CL417.Contig5_All, CL3034.Contig2_All, Unigene7204_All, CL8392.Contig1_All, CL200.Contig1_All, Unigene3383_All, Unigene4114_All, Unigene14682_All, CL454.Contig2_All, Unigene5020_All, Unigene26641_All, CL5095.Contig1_All, CL3332.Contig1_All, CL331.Contig1_All, Unigene26244_All, CL2490.Contig1_All, Unigene10036_All, Unigene4061_All, CL445.Contig1_All, CL7324.Contig1_All, CL4316.Contig1_All, CL674.Contig5_All, CL9787.Contig2_All, CL1959.Contig2_All, CL7124.Contig2_All, CL6219.Contig1_All, CL3821.Contig1_All, Unigene492_All, CL6637.Contig1_All, CL5525.Contig2_All, Unigene26311_All, Unigene794_All, Unigene3179_All, CL6129.Contig1_All, CL6916.Contig1_All, CL6295.Contig2_All, CL9953.Contig1_All, Unigene28268_All, CL3833.Contig1_All, CL6614.Contig1_All, Unigene6197_All, CL7452.Contig3_All, Unigene27314_All, CL7286.Contig1_All, CL4841.Contig2_All, CL5475.Contig3_All, Unigene8351_All, CL7503.Contig1_All, CL4585.Contig1_All, CL2170.Contig3_All, CL9849.Contig2_All,

		Unigene25608_All, CL3070.Contig5_All, Unigene1544_All, CL1803.Contig2_All, CL5476.Contig2_All, Unigene5482_All, CL7664.Contig1_All, CL6341.Contig2_All, CL6339.Contig3_All, Unigene4741_All, CL3469.Contig1_All, CL1735.Contig2_All, CL2598.Contig1_All, Unigene25786_All, CL7841.Contig3_All, Unigene29389_All, Unigene27313_All, CL1803.Contig3_All, CL9281.Contig1_All, CL9281.Contig2_All, Unigene26882_All, Unigene7581_All, CL8987.Contig2_All, CL8268.Contig2_All, CL5345.Contig1_All, Unigene8149_All, CL3204.Contig3_All, CL8622.Contig1_All, CL3342.Contig3_All, Unigene6696_All, CL8268.Contig3_All, CL5638.Contig2_All, CL3809.Contig2_All, Unigene3207_All, CL443.Contig1_All, CL1660.Contig4_All, CL9379.Contig1_All, CL2088.Contig1_All, Unigene5872_All, Unigene6249_All, CL6712.Contig1_All, CL8664.Contig3_All, CL8508.Contig1_All, CL1959.Contig1_All, CL9394.Contig1_All, Unigene5748_All, CL8306.Contig1_All, Unigene25872_All, CL4442.Contig1_All, CL6826.Contig1_All, CL8984.Contig3_All, CL2291.Contig2_All, CL9064.Contig2_All, CL5369.Contig3_All, CL3342.Contig1_All, Unigene2804_All, CL4667.Contig1_All, CL7282.Contig3_All, CL2464.Contig2_All, CL9078.Contig1_All, CL7107.Contig1_All, Unigene6212_All, CL6224.Contig1_All, Unigene3291_All, Unigene749_All, CL2540.Contig1_All, CL9240.Contig1_All, CL3289.Contig1_All, Unigene26168_All, CL7342.Contig1_All, CL1157.Contig2_All, CL3894.Contig2_All, Unigene26585_All, Unigene6153_All, CL222.Contig1_All, CL1028.Contig2_All, CL5016.Contig2_All, CL7831.Contig2_All, Unigene4471_All, CL1936.Contig1_All, CL5445.Contig1_All, CL3382.Contig2_All, CL824.Contig2_All, CL6382.Contig1_All, CL4098.Contig2_All, CL6826.Contig2_All, CL8471.Contig2_All, Unigene8059_All, CL6712.Contig2_All, CL2367.Contig1_All, CL3543.Contig1_All, CL8664.Contig1_All, CL3034.Contig1_All, CL6098.Contig1_All, CL10058.Contig2_All, CL9544.Contig1_All, CL4904.Contig1_All, CL7409.Contig1_All, CL4606.Contig2_All, CL8754.Contig2_All, Unigene28039_All, CL5550.Contig3_All, CL5476.Contig1_All, CL4016.Contig2_All, Unigene27679_All, CL9739.Contig1_All, Unigene25939_All, Unigene601_All, Unigene3110_All, CL8306.Contig2_All, CL3579.Contig1_All, CL3022.Contig1_All, CL7002.Contig2_All, CL9915.Contig2_All, CL6584.Contig3_All, CL443.Contig3_All, CL5550.Contig1_All, CL3136.Contig1_All, CL923.Contig1_All, CL554.Contig2_All, CL5445.Contig3_All, CL2088.Contig2_All, CL7124.Contig1_All, CL9081.Contig1_All, Unigene5837_All, CL4695.Contig3_All, Unigene27524_All, CL7831.Contig1_All, CL2972.Contig2_All, Unigene7481_All, CL6786.Contig1_All, CL4606.Contig1_All, Unigene8364_All, CL4474.Contig1_All, CL5346.Contig1_All, CL3932.Contig1_All, Unigene4972_All, Unigene4852_All, Unigene27434_All, CL4013.Contig2_All, CL3641.Contig1_All, CL5986.Contig2_All, Unigene6172_All, CL5445.Contig2_All, CL5082.Contig2_All, CL4169.Contig1_All, CL4657.Contig1_All, Unigene3171_All, Unigene7850_All, Unigene21428_All, CL5044.Contig1_All, CL8984.Contig2_All, CL5986.Contig1_All, Unigene7623_All, Unigene2497_All, CL9338.Contig1_All, Unigene326_All, Unigene27974_All, CL9081.Contig2_All
2	Valine, leucine and isoleucine degradation	CL9784.Contig1_All, Unigene1665_All, Unigene8070_All, CL2312.Contig1_All, CL9241.Contig2_All, Unigene28419_All, CL7504.Contig1_All, CL3037.Contig5_All, Unigene6758_All, CL9394.Contig2_All, CL4031.Contig2_All, CL8398.Contig1_All, CL4516.Contig1_All, CL7504.Contig2_All, CL3677.Contig3_All, CL1079.Contig1_All, CL3311.Contig1_All, CL6614.Contig2_All, CL8398.Contig3_All, CL9241.Contig1_All, CL1341.Contig2_All, CL3677.Contig1_All, CL6343.Contig2_All, CL9955.Contig1_All, CL1452.Contig2_All, CL3037.Contig1_All, CL6249.Contig2_All, Unigene1640_All, CL6224.Contig2_All, CL1803.Contig1_All, CL244.Contig3_All, CL1452.Contig1_All, Unigene27103_All, Unigene8408_All, CL417.Contig5_All, CL331.Contig1_All,

		CL2490.Contig1_All, Unigene794_All, CL6916.Contig1_All, CL6614.Contig1_All, CL7286.Contig1_All, CL1803.Contig2_All, Unigene4741_All, CL2598.Contig1_All, Unigene25786_All, CL1803.Contig3_All, CL6712.Contig1_All, CL9394.Contig1_All, Unigene25872_All, CL6224.Contig1_All, CL3894.Contig2_All, CL1028.Contig2_All, CL6712.Contig2_All, CL2367.Contig1_All, CL6098.Contig1_All, CL7409.Contig1_All, CL4606.Contig2_All, CL923.Contig1_All, CL4606.Contig1_All, CL5986.Contig2_All, CL5986.Contig1_All
3	Glycolysis / Gluconeogenesis	CL9784.Contig1_All, CL7964.Contig2_All, CL6939.Contig2_All, CL2464.Contig3_All, CL3815.Contig1_All, CL8538.Contig1_All, Unigene22338_All, CL200.Contig3_All, Unigene6758_All, CL837.Contig2_All, CL7766.Contig1_All, CL8398.Contig1_All, CL9087.Contig1_All, CL3204.Contig1_All, CL1079.Contig1_All, Unigene1464_All, CL8398.Contig3_All, CL9247.Contig2_All, CL9544.Contig2_All, Unigene22956_All, CL9955.Contig1_All, CL8363.Contig1_All, Unigene5129_All, Unigene6068_All, CL9862.Contig1_All, CL9247.Contig1_All, CL8538.Contig2_All, Unigene7840_All, Unigene8619_All, CL1060.Contig3_All, CL3070.Contig4_All, Unigene6213_All, CL837.Contig1_All, Unigene8408_All, CL200.Contig1_All, Unigene14682_All, Unigene26641_All, CL5095.Contig1_All, CL331.Contig1_All, CL7124.Contig2_All, Unigene492_All, Unigene6197_All, CL5475.Contig3_All, CL3070.Contig5_All, Unigene1544_All, CL6339.Contig3_All, CL2598.Contig1_All, CL3204.Contig3_All, CL3342.Contig3_All, CL3809.Contig2_All, CL3342.Contig1_All, CL2464.Contig2_All, CL7342.Contig1_All, CL1028.Contig2_All, CL6098.Contig1_All, CL9544.Contig1_All, CL7409.Contig1_All, CL7002.Contig2_All, CL923.Contig1_All, CL7124.Contig1_All, CL9081.Contig1_All, Unigene5837_All, CL6786.Contig1_All, CL4474.Contig1_All, Unigene7850_All, Unigene2497_All, CL9081.Contig2_All
4	Fatty acid metabolism	CL9784.Contig1_All, Unigene1665_All, CL5661.Contig1_All, Unigene8070_All, CL2312.Contig1_All, Unigene7838_All, Unigene27962_All, Unigene953_All, Unigene28419_All, CL7504.Contig1_All, Unigene6758_All, CL8398.Contig1_All, CL4516.Contig1_All, CL7504.Contig2_All, CL9087.Contig1_All, CL3204.Contig1_All, Unigene7473_All, CL1079.Contig1_All, CL8398.Contig3_All, CL1341.Contig2_All, CL6343.Contig2_All, CL9955.Contig1_All, CL8363.Contig1_All, CL5436.Contig1_All, CL6249.Contig2_All, Unigene29448_All, CL3010.Contig1_All, CL5436.Contig6_All, CL6224.Contig2_All, Unigene22136_All, CL1803.Contig1_All, CL7093.Contig1_All, Unigene6213_All, Unigene4883_All, Unigene8408_All, CL417.Contig5_All, CL3034.Contig2_All, CL331.Contig1_All, CL3821.Contig1_All, Unigene492_All, Unigene794_All, CL6916.Contig1_All, CL9849.Contig2_All, CL1803.Contig2_All, CL6339.Contig3_All, Unigene4741_All, CL2598.Contig1_All, CL1803.Contig3_All, CL3204.Contig3_All, Unigene6696_All, CL3953.Contig2_All, CL6712.Contig1_All, Unigene25872_All, CL3507.Contig1_All, CL4667.Contig1_All, CL6224.Contig1_All, CL1028.Contig2_All, CL6712.Contig2_All, CL2367.Contig1_All, CL3034.Contig1_All, CL6098.Contig1_All, CL7002.Contig2_All, CL923.Contig1_All, Unigene5837_All, CL5986.Contig2_All, CL4169.Contig1_All, CL5986.Contig1_All
5	Pyruvate metabolism	CL9784.Contig1_All, CL3543.Contig2_All, Unigene6758_All, CL837.Contig2_All, CL7766.Contig1_All, CL8398.Contig1_All, CL7647.Contig1_All, CL1079.Contig1_All, Unigene1464_All, CL8398.Contig3_All, Unigene21985_All, Unigene3204_All, CL9544.Contig2_All, CL9955.Contig1_All, CL6679.Contig1_All, CL9862.Contig1_All, CL551.Contig34_All, CL6249.Contig2_All, CL6224.Contig2_All, CL1060.Contig3_All, CL4939.Contig1_All, CL986.Contig2_All, CL3070.Contig4_All, CL837.Contig1_All, Unigene8408_All, Unigene26641_All, CL5095.Contig1_All, CL331.Contig1_All, CL7124.Contig2_All, Unigene6197_All,

		Unigene27314_All, CL5475.Contig3_All, CL3070.Contig5_All, CL2598.Contig1_All, Unigene27313_All, Unigene7581_All, CL8268.Contig2_All, CL8268.Contig3_All, CL6224.Contig1_All, CL7342.Contig1_All, CL1028.Contig2_All, CL3543.Contig1_All, CL6098.Contig1_All, CL9544.Contig1_All, CL7409.Contig1_All, Unigene25939_All, Unigene3110_All, CL923.Contig1_All, CL7124.Contig1_All, CL4474.Contig1_All, Unigene2497_All
6	Citrate cycle (TCA cycle)	CL3543.Contig2_All, CL5383.Contig2_All, CL7130.Contig1_All, CL1012.Contig10_All, CL731.Contig1_All, Unigene3299_All, CL5374.Contig3_All, Unigene1464_All, Unigene21985_All, Unigene3204_All, CL9544.Contig2_All, CL9862.Contig1_All, CL551.Contig34_All, CL5142.Contig1_All, CL1060.Contig3_All, Unigene3119_All, CL4013.Contig1_All, CL1530.Contig2_All, Unigene10036_All, CL9787.Contig2_All, Unigene27314_All, CL5475.Contig3_All, CL5476.Contig2_All, Unigene27313_All, Unigene7581_All, CL8268.Contig2_All, CL8268.Contig3_All, CL9078.Contig1_All, CL3289.Contig1_All, CL7342.Contig1_All, CL7831.Contig2_All, CL3543.Contig1_All, CL9544.Contig1_All, CL7409.Contig1_All, CL5476.Contig1_All, Unigene25939_All, Unigene3110_All, Unigene27524_All, CL7831.Contig1_All, CL4474.Contig1_All, Unigene4972_All, CL4013.Contig2_All
7	beta-Alanine metabolism	CL9784.Contig1_All, Unigene8070_All, CL2312.Contig1_All, Unigene28419_All, CL3037.Contig5_All, Unigene6758_All, CL8398.Contig1_All, CL4516.Contig1_All, CL1079.Contig1_All, CL6614.Contig2_All, CL8398.Contig3_All, CL1341.Contig2_All, CL9413.Contig1_All, CL6343.Contig2_All, CL9955.Contig1_All, CL3037.Contig1_All, Unigene1640_All, CL1803.Contig1_All, Unigene27103_All, Unigene8408_All, CL417.Contig5_All, CL331.Contig1_All, CL6916.Contig1_All, CL6614.Contig1_All, CL1803.Contig2_All, Unigene4741_All, CL2598.Contig1_All, CL1803.Contig3_All, Unigene3207_All, CL6712.Contig1_All, CL8306.Contig1_All, CL1028.Contig2_All, CL6712.Contig2_All, CL2367.Contig1_All, CL6098.Contig1_All, CL4016.Contig2_All, CL8306.Contig2_All, CL923.Contig1_All
8	Aminoacyl-tRNA biosynthesis	CL2771.Contig1_All, CL2004.Contig1_All, CL1096.Contig6_All, CL4404.Contig2_All, CL2265.Contig1_All, Unigene24027_All, CL2265.Contig3_All, CL8383.Contig3_All, CL7340.Contig1_All, CL5782.Contig2_All, CL6376.Contig2_All, Unigene4153_All, CL2004.Contig2_All, CL5782.Contig1_All, CL1096.Contig2_All, CL1473.Contig1_All, CL9434.Contig1_All, CL6690.Contig1_All, Unigene28268_All, CL8383.Contig1_All, CL770.Contig1_All, CL6416.Contig1_All, Unigene5774_All, CL2771.Contig3_All, CL6376.Contig1_All, CL7639.Contig3_All
9	Oxidative phosphorylation	CL1623.Contig1_All, CL9723.Contig2_All, Unigene22279_All, CL1521.Contig2_All, CL1012.Contig10_All, Unigene28027_All, CL2717.Contig2_All, Unigene11200_All, Unigene3299_All, CL5977.Contig2_All, CL3063.Contig1_All, CL6788.Contig1_All, CL3504.Contig8_All, CL6788.Contig2_All, CL846.Contig2_All, CL554.Contig1_All, CL3305.Contig4_All, Unigene6334_All, Unigene2532_All, CL9475.Contig2_All, CL846.Contig4_All, Unigene2447_All, CL3414.Contig2_All, CL5525.Contig1_All, CL9379.Contig2_All, CL2825.Contig2_All, CL3414.Contig5_All, Unigene6769_All, Unigene10607_All, Unigene1599_All, Unigene29335_All, CL5142.Contig1_All, Unigene29299_All, CL4013.Contig1_All, CL2170.Contig4_All, Unigene28320_All, CL1151.Contig3_All, Unigene4625_All, Unigene11218_All, CL3414.Contig4_All, Unigene2480_All, Unigene7204_All, Unigene26244_All, CL1959.Contig2_All, CL5525.Contig2_All, Unigene26311_All, CL4841.Contig2_All, CL2170.Contig3_All, Unigene25608_All, CL9281.Contig1_All, CL9281.Contig2_All, CL9379.Contig1_All, CL2088.Contig1_All, CL1959.Contig1_All, CL6826.Contig1_All, Unigene6212_All, Unigene749_All, Unigene26585_All, CL3382.Contig2_All, CL4098.Contig2_All, CL6826.Contig2_All, Unigene8059_All, Unigene27679_All, CL3022.Contig1_All, CL9915.Contig2_All, CL554.Contig2_All,

		CL2088.Contig2_All, Unigene27524_All, Unigene7481_All, CL4013.Contig2_All, Unigene7623_All
10	Propanoate metabolism	CL9784.Contig1_All, Unigene8070_All, CL2312.Contig1_All, CL7130.Contig1_All, Unigene28419_All, CL3037.Contig5_All, Unigene6758_All, CL7766.Contig1_All, CL8398.Contig1_All, CL4516.Contig1_All, CL1079.Contig1_All, CL6614.Contig2_All, CL8398.Contig3_All, CL1341.Contig2_All, CL6343.Contig2_All, CL9955.Contig1_All, CL3037.Contig1_All, CL6249.Contig2_All, Unigene1640_All, CL6224.Contig2_All, CL1803.Contig1_All, CL986.Contig2_All, Unigene27103_All, Unigene8408_All, CL417.Contig5_All, CL331.Contig1_All, CL2490.Contig1_All, CL6916.Contig1_All, CL6614.Contig1_All, CL7286.Contig1_All, CL1803.Contig2_All, Unigene4741_All, CL2598.Contig1_All, CL1803.Contig3_All, CL6712.Contig1_All, CL9078.Contig1_All, CL6224.Contig1_All, CL1028.Contig2_All, CL6712.Contig2_All, CL2367.Contig1_All, CL6098.Contig1_All, CL923.Contig1_All
11	Parkinson's disease	Unigene835_All, CL1623.Contig1_All, Unigene22279_All, Unigene5913_All, CL1521.Contig2_All, CL1012.Contig10_All, Unigene28027_All, Unigene11200_All, Unigene3299_All, CL5977.Contig2_All, CL3063.Contig1_All, CL6788.Contig1_All, CL6788.Contig2_All, CL3305.Contig4_All, Unigene6334_All, Unigene2532_All, CL9475.Contig2_All, CL9189.Contig2_All, Unigene2447_All, CL3414.Contig2_All, CL5525.Contig1_All, Unigene686_All, CL2825.Contig2_All, CL3414.Contig5_All, Unigene6769_All, Unigene1599_All, Unigene29335_All, CL5142.Contig1_All, Unigene29299_All, CL4013.Contig1_All, Unigene28320_All, CL1151.Contig3_All, Unigene4625_All, Unigene11218_All, CL3414.Contig4_All, Unigene2480_All, CL9189.Contig1_All, Unigene7204_All, Unigene26244_All, CL1959.Contig2_All, CL5525.Contig2_All, Unigene26311_All, CL4841.Contig2_All, Unigene25608_All, CL9281.Contig1_All, CL9281.Contig2_All, CL4242.Contig1_All, CL2088.Contig1_All, CL4242.Contig2_All, CL1959.Contig1_All, Unigene749_All, Unigene26585_All, CL4098.Contig2_All, Unigene8059_All, Unigene22661_All, Unigene27679_All, CL3022.Contig1_All, CL9915.Contig2_All, CL2088.Contig2_All, Unigene27524_All, Unigene7481_All, CL4013.Contig2_All, Unigene7623_All
12	Peroxisome	Unigene22560_All, CL3932.Contig2_All, CL2312.Contig1_All, Unigene2609_All, Unigene27962_All, Unigene953_All, CL860.Contig3_All, Unigene21846_All, CL9975.Contig1_All, CL4391.Contig2_All, Unigene28419_All, CL7557.Contig4_All, CL9609.Contig1_All, Unigene3398_All, CL9104.Contig2_All, Unigene2112_All, CL7213.Contig1_All, CL1341.Contig2_All, CL9412.Contig2_All, Unigene708_All, CL3469.Contig5_All, CL5436.Contig1_All, CL3010.Contig1_All, CL5436.Contig6_All, Unigene22136_All, CL1803.Contig1_All, CL7093.Contig1_All, Unigene9167_All, Unigene27273_All, CL7557.Contig1_All, Unigene3256_All, CL529.Contig3_All, CL7557.Contig2_All, Unigene27764_All, CL3291.Contig1_All, CL4247.Contig1_All, CL417.Contig5_All, CL3034.Contig2_All, Unigene3383_All, CL454.Contig2_All, Unigene5020_All, CL7258.Contig2_All, CL7324.Contig1_All, CL7165.Contig1_All, CL1803.Contig2_All, CL49.Contig1_All, Unigene4741_All, CL3469.Contig1_All, CL1803.Contig3_All, CL8987.Contig2_All, CL5225.Contig1_All, Unigene5872_All, CL6712.Contig1_All, Unigene5748_All, CL4667.Contig1_All, CL7020.Contig2_All, CL5488.Contig1_All, Unigene3291_All, CL1157.Contig2_All, CL7831.Contig2_All, CL5445.Contig1_All, CL824.Contig2_All, CL6712.Contig2_All, CL3034.Contig1_All, CL5445.Contig3_All, CL7831.Contig1_All, CL3932.Contig1_All, Unigene6172_All, CL5445.Contig2_All, Unigene326_All, Unigene27974_All
13	Alzheimer's disease	CL1623.Contig1_All, Unigene22279_All, CL3787.Contig1_All, Unigene5913_All, CL1521.Contig2_All, CL1012.Contig10_All, Unigene28027_All, Unigene11200_All, Unigene3299_All, CL5977.Contig2_All, CL3063.Contig1_All, CL6788.Contig1_All,

		CL9692.Contig2_All, CL997.Contig1_All, CL6788.Contig2_All, CL3305.Contig4_All, Unigene6334_All, Unigene2532_All, CL9475.Contig2_All, Unigene22956_All, Unigene2447_All, CL3414.Contig2_All, CL5525.Contig1_All, Unigene7840_All, CL2825.Contig2_All, CL3414.Contig5_All, Unigene6769_All, Unigene8619_All, Unigene1599_All, Unigene29335_All, CL5142.Contig1_All, Unigene29299_All, CL4013.Contig1_All, CL2891.Contig3_All, Unigene28320_All, CL1151.Contig3_All, Unigene4625_All, Unigene11218_All, CL3414.Contig4_All, Unigene2480_All, Unigene7204_All, Unigene14682_All, Unigene26244_All, CL1959.Contig2_All, CL5525.Contig2_All, Unigene26311_All, CL4841.Contig2_All, Unigene25608_All, Unigene25786_All, CL9281.Contig1_All, CL9281.Contig2_All, CL2088.Contig1_All, CL1959.Contig1_All, CL2891.Contig1_All, Unigene749_All, CL3894.Contig2_All, Unigene26585_All, CL4098.Contig2_All, Unigene8059_All, Unigene27679_All, CL3022.Contig1_All, CL9915.Contig2_All, CL2088.Contig2_All, Unigene27524_All, Unigene7481_All, CL4013.Contig2_All, Unigene7623_All
14	Butanoate metabolism	Unigene1665_All, Unigene8070_All, Unigene118_All, Unigene104_All, CL9394.Contig2_All, CL4516.Contig1_All, CL6614.Contig2_All, CL6343.Contig2_All, CL9862.Contig1_All, CL6249.Contig2_All, CL6224.Contig2_All, CL7557.Contig1_All, CL244.Contig3_All, CL5369.Contig1_All, CL2586.Contig1_All, Unigene794_All, CL6916.Contig1_All, CL6614.Contig1_All, CL5475.Contig3_All, CL1735.Contig2_All, CL9394.Contig1_All, Unigene25872_All, CL5369.Contig3_All, CL6224.Contig1_All, CL7342.Contig1_All, CL2367.Contig1_All, CL4474.Contig1_All, CL4657.Contig1_All
15	Retinol metabolism	Unigene4206_All, CL9384.Contig2_All, Unigene2609_All, Unigene21846_All, CL9975.Contig1_All, CL7557.Contig4_All, CL9609.Contig1_All, Unigene3398_All, CL9087.Contig1_All, CL3204.Contig1_All, Unigene7473_All, CL4435.Contig1_All, CL7213.Contig1_All, Unigene144_All, CL3687.Contig3_All, Unigene708_All, CL8363.Contig1_All, CL3185.Contig1_All, CL3888.Contig1_All, CL2756.Contig1_All, CL7557.Contig1_All, Unigene3256_All, CL7557.Contig2_All, CL9739.Contig2_All, Unigene6213_All, CL454.Contig2_All, Unigene5020_All, CL1341.Contig1_All, CL331.Contig1_All, Unigene492_All, CL6295.Contig2_All, Unigene3287_All, CL6339.Contig3_All, CL1341.Contig3_All, CL3204.Contig3_All, Unigene5872_All, Unigene2804_All, CL1157.Contig2_All, CL5445.Contig1_All, CL9739.Contig1_All, CL7002.Contig2_All, CL5445.Contig3_All, Unigene5837_All, Unigene27434_All, Unigene6172_All, CL5445.Contig2_All, Unigene3171_All, Unigene27974_All
16	Tryptophan metabolism	CL9784.Contig1_All, Unigene1665_All, CL5661.Contig1_All, Unigene8070_All, Unigene7838_All, CL731.Contig1_All, Unigene6758_All, CL8398.Contig1_All, CL4516.Contig1_All, CL4623.Contig1_All, Unigene7473_All, CL1079.Contig1_All, CL8398.Contig3_All, CL6343.Contig2_All, CL9955.Contig1_All, CL6249.Contig2_All, Unigene29448_All, CL6224.Contig2_All, CL8736.Contig1_All, Unigene27273_All, Unigene24991_All, Unigene4883_All, Unigene8408_All, CL331.Contig1_All, CL3821.Contig1_All, Unigene794_All, CL6916.Contig1_All, CL2598.Contig1_All, Unigene909_All, Unigene6696_All, Unigene25872_All, CL5903.Contig1_All, CL6224.Contig1_All, CL1028.Contig2_All, CL824.Contig2_All, Unigene22507_All, CL2367.Contig1_All, CL6098.Contig1_All, CL923.Contig1_All, Unigene4972_All, CL4169.Contig1_All
17	Galactose metabolism	CL2780.Contig3_All, CL2664.Contig1_All, CL200.Contig3_All, CL837.Contig2_All, CL4768.Contig1_All, Unigene29100_All, Unigene6820_All, Unigene26386_All, CL837.Contig1_All, CL200.Contig1_All, Unigene26641_All, CL5095.Contig1_All, CL7124.Contig2_All, Unigene6197_All, CL3342.Contig3_All, CL3342.Contig1_All, Unigene4471_All, CL7124.Contig1_All,

18	Huntington's disease	CL9081.Contig1_All, Unigene2497_All, CL9081.Contig2_All CL1623.Contig1_All, Unigene5244_All, Unigene22279_All, Unigene5913_All, CL1521.Contig2_All, CL1012.Contig10_All, Unigene28027_All, Unigene11200_All, Unigene3299_All, CL5977.Contig2_All, CL3063.Contig1_All, CL6788.Contig1_All, CL2220.Contig1_All, CL6788.Contig2_All, Unigene2081_All, CL3305.Contig4_All, Unigene6334_All, Unigene2532_All, CL9475.Contig2_All, CL9189.Contig2_All, Unigene2447_All, CL3414.Contig2_All, CL5525.Contig1_All, CL2220.Contig2_All, Unigene686_All, Unigene21797_All, CL2825.Contig2_All, CL3414.Contig5_All, Unigene6769_All, Unigene1599_All, Unigene29335_All, Unigene9167_All, CL5142.Contig1_All, Unigene29299_All, CL56.Contig2_All, CL4013.Contig1_All, CL56.Contig1_All, Unigene28320_All, CL1151.Contig3_All, Unigene4625_All, Unigene11218_All, CL3414.Contig4_All, Unigene2480_All, CL9189.Contig1_All, Unigene7204_All, Unigene28505_All, CL7258.Contig2_All, Unigene26244_All, CL1959.Contig2_All, CL5525.Contig2_All, Unigene26311_All, CL4841.Contig2_All, Unigene25608_All, CL9281.Contig1_All, CL9281.Contig2_All, CL4242.Contig1_All, CL2535.Contig2_All, CL2088.Contig1_All, CL4242.Contig2_All, CL1959.Contig1_All, Unigene749_All, Unigene26585_All, CL4098.Contig2_All, Unigene8059_All, Unigene27679_All, CL3022.Contig1_All, CL9915.Contig2_All, CL2088.Contig2_All, Unigene27524_All, Unigene7481_All, CL4013.Contig2_All, Unigene7623_All
19	Arginine and proline metabolism	CL9784.Contig1_All, CL7841.Contig1_All, CL3798.Contig1_All, CL8564.Contig2_All, CL1868.Contig3_All, Unigene6758_All, CL5897.Contig1_All, CL8398.Contig1_All, CL4623.Contig1_All, CL1079.Contig1_All, CL2270.Contig1_All, CL8398.Contig3_All, CL9955.Contig1_All, CL3469.Contig5_All, CL1054.Contig1_All, CL8736.Contig1_All, CL8564.Contig1_All, Unigene24991_All, Unigene8408_All, CL8392.Contig1_All, CL331.Contig1_All, CL3469.Contig1_All, CL2598.Contig1_All, CL7841.Contig3_All, Unigene909_All, Unigene8149_All, Unigene3207_All, CL8664.Contig3_All, CL8508.Contig1_All, CL8306.Contig1_All, CL2291.Contig2_All, CL5903.Contig1_All, CL1028.Contig2_All, CL1936.Contig1_All, Unigene22507_All, CL8664.Contig1_All, CL6098.Contig1_All, CL8306.Contig2_All, CL923.Contig1_All, CL5082.Contig2_All
20	Glyoxylate and dicarboxylate metabolism	CL3543.Contig2_All, CL5383.Contig2_All, CL4391.Contig2_All, CL7647.Contig1_All, Unigene21985_All, CL6249.Contig2_All, CL6224.Contig2_All, CL4939.Contig1_All, Unigene27273_All, Unigene27764_All, CL2490.Contig1_All, Unigene10036_All, CL7286.Contig1_All, CL5476.Contig2_All, Unigene7581_All, CL8268.Contig2_All, CL8268.Contig3_All, Unigene5748_All, CL6224.Contig1_All, CL824.Contig2_All, CL3543.Contig1_All, CL5476.Contig1_All, Unigene3110_All
21	PPAR signaling pathway	CL3932.Contig2_All, CL2312.Contig1_All, CL1167.Contig1_All, Unigene28419_All, Unigene9626_All, CL9394.Contig2_All, Unigene26016_All, CL1341.Contig2_All, CL9400.Contig1_All, CL5436.Contig1_All, CL3010.Contig1_All, CL5436.Contig6_All, CL1803.Contig1_All, CL1060.Contig3_All, CL417.Contig5_All, CL3034.Contig2_All, CL5718.Contig2_All, CL1803.Contig2_All, Unigene4741_All, CL1803.Contig3_All, CL3953.Contig2_All, CL6712.Contig1_All, Unigene29387_All, CL9394.Contig1_All, CL3507.Contig1_All, CL4667.Contig1_All, CL6712.Contig2_All, CL3034.Contig1_All, CL3932.Contig1_All
22	Metabolism of xenobiotics by cytochrome P450	CL9384.Contig2_All, CL611.Contig2_All, Unigene27409_All, Unigene28889_All, CL3833.Contig2_All, Unigene6389_All, Unigene6758_All, CL8398.Contig1_All, Unigene28933_All, CL5647.Contig3_All, CL9087.Contig1_All, CL3204.Contig1_All, Unigene7473_All, CL4435.Contig1_All, CL8398.Contig3_All, CL5462.Contig1_All, CL3687.Contig3_All, CL8363.Contig1_All, Unigene27153_All, CL4808.Contig1_All, Unigene21637_All, CL5647.Contig4_All, CL5647.Contig1_All, CL2476.Contig2_All,

		CL8338.Contig2_All, Unigene6213_All, Unigene8408_All, Unigene7663_All, CL331.Contig1_All, Unigene492_All, CL6295.Contig2_All, CL3833.Contig1_All, CL5522.Contig1_All, CL6435.Contig1_All, Unigene5482_All, CL6339.Contig3_All, CL2598.Contig1_All, Unigene28054_All, CL3204.Contig3_All, CL8622.Contig1_All, CL5638.Contig2_All, Unigene2804_All, Unigene26168_All, CL6098.Contig1_All, CL7002.Contig2_All, CL923.Contig1_All, Unigene5837_All, Unigene21428_All
23	Pentose phosphate pathway	CL9767.Contig2_All, CL3815.Contig1_All, CL9241.Contig2_All, Unigene22338_All, Unigene4651_All, CL200.Contig3_All, CL4031.Contig2_All, CL9241.Contig1_All, CL9247.Contig2_All, Unigene6068_All, CL9247.Contig1_All, CL200.Contig1_All, CL4585.Contig1_All, Unigene1544_All, CL3342.Contig3_All, CL3809.Contig2_All, CL3342.Contig1_All, CL2540.Contig1_All, CL5016.Contig2_All, CL9081.Contig1_All, CL2972.Contig2_All, Unigene7850_All, CL9081.Contig2_All
24	Drug metabolism - cytochrome P450	CL9384.Contig2_All, Unigene27409_All, CL3833.Contig2_All, Unigene6389_All, Unigene6758_All, CL8398.Contig1_All, Unigene28933_All, CL1670.Contig6_All, CL9087.Contig1_All, CL3204.Contig1_All, Unigene7473_All, CL4435.Contig1_All, CL8398.Contig3_All, CL5462.Contig1_All, CL3687.Contig3_All, CL8363.Contig1_All, Unigene27153_All, CL1670.Contig5_All, Unigene21637_All, CL6660.Contig1_All, Unigene28056_All, CL2476.Contig2_All, CL8338.Contig2_All, Unigene6213_All, Unigene8408_All, CL331.Contig1_All, Unigene492_All, CL6295.Contig2_All, CL3833.Contig1_All, Unigene5482_All, CL6339.Contig3_All, CL2598.Contig1_All, Unigene28054_All, CL3204.Contig3_All, CL8622.Contig1_All, CL5638.Contig2_All, Unigene26168_All, CL6098.Contig1_All, Unigene6866_All, CL7002.Contig2_All, CL923.Contig1_All, Unigene5837_All, CL1670.Contig4_All, Unigene21428_All
25	Lysine degradation	CL9784.Contig1_All, Unigene1665_All, Unigene8070_All, CL731.Contig1_All, Unigene6758_All, CL8398.Contig1_All, CL4516.Contig1_All, CL4134.Contig5_All, CL1079.Contig1_All, CL8398.Contig3_All, CL6343.Contig2_All, CL9955.Contig1_All, CL6249.Contig2_All, Unigene29448_All, CL6224.Contig2_All, Unigene8408_All, CL331.Contig1_All, Unigene794_All, CL6916.Contig1_All, CL2598.Contig1_All, Unigene25872_All, CL6224.Contig1_All, CL1028.Contig2_All, CL2367.Contig1_All, CL6098.Contig1_All, CL923.Contig1_All, Unigene4972_All
26	Biosynthesis of unsaturated fatty acids (no map in kegg database)	Unigene8070_All, CL2312.Contig1_All, CL2047.Contig2_All, Unigene28419_All, CL4516.Contig1_All, CL1341.Contig2_All, CL1803.Contig1_All, CL417.Contig5_All, CL1803.Contig2_All, Unigene4741_All, CL1735.Contig2_All, CL1803.Contig3_All, CL6712.Contig1_All, Unigene29387_All, CL5445.Contig1_All, CL6712.Contig2_All, CL5445.Contig2_All
27	Glutathione metabolism	Unigene5244_All, CL4988.Contig1_All, Unigene27409_All, CL2660.Contig1_All, CL3833.Contig2_All, CL9241.Contig2_All, Unigene6389_All, CL4031.Contig2_All, Unigene2081_All, Unigene28933_All, CL9241.Contig1_All, CL5462.Contig1_All, Unigene24067_All, Unigene27153_All, Unigene21797_All, CL8471.Contig1_All, Unigene21637_All, CL2476.Contig2_All, CL8338.Contig2_All, CL234.Contig2_All, CL4988.Contig3_All, CL8479.Contig1_All, CL959.Contig2_All, Unigene28505_All, Unigene7563_All, CL3332.Contig1_All, CL3833.Contig1_All, CL4585.Contig1_All, Unigene5482_All, Unigene28054_All, CL8622.Contig1_All, CL5638.Contig2_All, Unigene3207_All, Unigene6249_All, CL8479.Contig2_All, Unigene26168_All, CL7831.Contig2_All, CL8471.Contig2_All, CL7831.Contig1_All, Unigene21428_All
28	Phenylalanine metabolism	CL573.Contig1_All, CL8564.Contig2_All, CL573.Contig2_All, Unigene6758_All, CL8398.Contig1_All, CL4623.Contig1_All, Unigene1201_All, CL6584.Contig1_All, CL8398.Contig3_All, Unigene28629_All, CL8736.Contig1_All, CL8564.Contig1_All, Unigene24991_All, Unigene8408_All, Unigene27775_All,

		Unigene4612_All, CL331.Contig1_All, CL2598.Contig1_All, Unigene909_All, CL5903.Contig1_All, Unigene22507_All, CL6098.Contig1_All, CL8754.Contig2_All, CL6584.Contig3_All, CL923.Contig1_All, CL5082.Contig2_All
29	Glycerolipid metabolism	CL9784.Contig1_All, CL1181.Contig3_All, Unigene6758_All, CL837.Contig2_All, CL8398.Contig1_All, CL1079.Contig1_All, CL8398.Contig3_All, CL9955.Contig1_All, CL4768.Contig1_All, Unigene5129_All, CL4126.Contig1_All, CL837.Contig1_All, Unigene8408_All, CL3242.Contig2_All, Unigene26641_All, CL5095.Contig1_All, CL331.Contig1_All, CL7124.Contig2_All, Unigene6197_All, CL2598.Contig1_All, CL1028.Contig2_All, CL6098.Contig1_All, Unigene601_All, CL923.Contig1_All, CL7124.Contig1_All, Unigene2497_All
30	Starch and sucrose metabolism	CL2780.Contig3_All, CL9384.Contig2_All, CL213.Contig4_All, CL6902.Contig4_All, CL200.Contig3_All, CL3579.Contig2_All, CL4435.Contig1_All, CL9247.Contig2_All, CL3687.Contig3_All, CL6902.Contig1_All, CL9247.Contig1_All, CL213.Contig3_All, Unigene29100_All, CL200.Contig1_All, CL6295.Contig2_All, CL3342.Contig3_All, CL8984.Contig3_All, CL3342.Contig1_All, Unigene4471_All, CL3579.Contig1_All, Unigene22681_All, CL8984.Contig2_All
31	Synthesis and degradation of ketone bodies	CL9394.Contig2_All, CL6249.Contig2_All, CL6224.Contig2_All, CL7557.Contig1_All, CL244.Contig3_All, CL9394.Contig1_All, CL6224.Contig1_All
32	Alanine, aspartate and glutamate metabolism	CL7107.Contig2_All, CL3798.Contig1_All, CL8564.Contig2_All, Unigene22665_All, CL5398.Contig1_All, CL6614.Contig2_All, CL3033.Contig1_All, CL8564.Contig1_All, Unigene27764_All, CL5369.Contig1_All, CL6614.Contig1_All, CL443.Contig1_All, CL8664.Contig3_All, Unigene5748_All, CL5369.Contig3_All, CL7107.Contig1_All, CL8664.Contig1_All, CL443.Contig3_All, CL4695.Contig3_All, CL5082.Contig2_All
33	Tyrosine metabolism	CL573.Contig1_All, CL8564.Contig2_All, Unigene26224_All, CL573.Contig2_All, Unigene6758_All, CL8398.Contig1_All, CL9087.Contig1_All, CL3204.Contig1_All, Unigene1201_All, CL8398.Contig3_All, CL8363.Contig1_All, CL8564.Contig1_All, Unigene6213_All, Unigene8408_All, Unigene27775_All, Unigene4612_All, CL331.Contig1_All, Unigene492_All, CL6339.Contig3_All, CL2598.Contig1_All, CL3204.Contig3_All, CL6098.Contig1_All, CL10058.Contig2_All, CL7002.Contig2_All, CL923.Contig1_All, Unigene5837_All, CL5082.Contig2_All
34	Mismatch repair	Unigene7602_All, Unigene22773_All, CL3399.Contig3_All, Unigene2458_All
35	Pentose and glucuronate interconversions	CL2780.Contig3_All, CL9384.Contig2_All, CL611.Contig2_All, Unigene28889_All, Unigene6758_All, CL837.Contig2_All, CL8398.Contig1_All, CL4435.Contig1_All, CL8398.Contig3_All, CL3687.Contig3_All, CL9955.Contig1_All, CL4808.Contig1_All, CL7557.Contig1_All, Unigene3256_All, CL7557.Contig2_All, CL837.Contig1_All, Unigene8408_All, Unigene7663_All, Unigene26641_All, CL5095.Contig1_All, CL331.Contig1_All, CL7124.Contig2_All, CL6295.Contig2_All, Unigene6197_All, CL2598.Contig1_All, Unigene29389_All, CL1028.Contig2_All, CL5445.Contig1_All, CL6098.Contig1_All, CL923.Contig1_All, CL5445.Contig3_All, CL7124.Contig1_All, Unigene2497_All
36	Arachidonic acid metabolism	Unigene5244_All, CL5661.Contig1_All, Unigene7838_All, Unigene27409_All, CL2660.Contig1_All, CL3833.Contig2_All, CL7104.Contig1_All, Unigene6389_All, Unigene2081_All, Unigene28933_All, CL5462.Contig1_All, Unigene27153_All, Unigene21797_All, CL5352.Contig2_All, Unigene21637_All, CL2476.Contig2_All, Unigene4883_All, CL2200.Contig1_All, Unigene28505_All, CL3332.Contig1_All, CL3821.Contig1_All, CL3833.Contig1_All, Unigene5482_All, CL8622.Contig1_All, Unigene6696_All, CL5638.Contig2_All, Unigene2804_All, Unigene26168_All, CL4169.Contig1_All, Unigene21428_All
37	Lysosome	CL2029.Contig1_All, Unigene27831_All, CL2664.Contig1_All, CL768.Contig3_All, CL2717.Contig2_All, Unigene22014_All,

		CL4004.Contig3_All, CL9293.Contig2_All, Unigene24033_All, CL4271.Contig1_All, Unigene2393_All, Unigene4264_All, Unigene7683_All, CL3279.Contig1_All, CL2220.Contig1_All, CL8183.Contig3_All, CL846.Contig2_All, CL3330.Contig1_All, CL2258.Contig1_All, CL3400.Contig1_All, CL846.Contig4_All, Unigene3191_All, Unigene2513_All, CL9588.Contig1_All, CL4768.Contig1_All, CL2220.Contig2_All, Unigene74_All, CL9252.Contig1_All, CL3388.Contig1_All, CL4281.Contig2_All, CL4271.Contig3_All, CL608.Contig4_All, CL445.Contig1_All, Unigene6964_All, Unigene2773_All, CL6059.Contig2_All, CL2258.Contig13_All, CL4456.Contig3_All, CL818.Contig1_All, Unigene917_All, CL7288.Contig1_All, CL701.Contig1_All, CL9776.Contig1_All, Unigene11930_All, Unigene29428_All, CL7190.Contig1_All, Unigene4235_All
38	alpha-Linolenic acid metabolism	CL2312.Contig1_All, Unigene28419_All, CL1341.Contig2_All, CL1803.Contig1_All, CL417.Contig5_All, CL1803.Contig2_All, Unigene4741_All, CL1803.Contig3_All, CL6712.Contig1_All, Unigene29387_All, CL6712.Contig2_All
39	Fatty acid elongation	Unigene1665_All, Unigene8070_All, CL2047.Contig2_All, CL7504.Contig1_All, CL4516.Contig1_All, CL7504.Contig2_All, CL6343.Contig2_All, Unigene794_All, CL6916.Contig1_All, CL1735.Contig2_All, Unigene25872_All, CL2367.Contig1_All, Unigene8364_All, CL5986.Contig2_All, CL5986.Contig1_All
40	Rheumatoid arthritis	CL9723.Contig2_All, CL2717.Contig2_All, CL846.Contig2_All, CL554.Contig1_All, CL846.Contig4_All, Unigene2513_All, CL9252.Contig1_All, Unigene4416_All, CL2170.Contig4_All, CL2170.Contig3_All, CL6059.Contig2_All, CL6826.Contig1_All, CL3382.Contig2_All, CL6826.Contig2_All, CL554.Contig2_All
41	ECM-receptor interaction	CL8738.Contig2_All, Unigene26102_All, CL5886.Contig1_All, CL328.Contig2_All, CL5497.Contig1_All, Unigene23556_All, Unigene4416_All, CL4294.Contig3_All, Unigene26870_All, CL785.Contig2_All, CL2070.Contig1_All, CL170.Contig5_All, Unigene636_All, CL3920.Contig3_All, CL342.Contig5_All, CL572.Contig1_All, CL8274.Contig3_All
42	Porphyrin and chlorophyll metabolism	CL9384.Contig2_All, CL5108.Contig2_All, CL4435.Contig1_All, CL3687.Contig3_All, CL7299.Contig1_All, CL7299.Contig2_All, CL5782.Contig2_All, CL5782.Contig1_All, CL6295.Contig2_All, Unigene28268_All
43	DNA replication	Unigene7602_All, Unigene22773_All, CL3399.Contig3_All, Unigene2458_All
44	Fructose and mannose metabolism	Unigene118_All, CL3815.Contig1_All, CL8538.Contig1_All, Unigene22338_All, Unigene104_All, CL837.Contig2_All, CL7505.Contig2_All, Unigene6068_All, CL8538.Contig2_All, CL837.Contig1_All, Unigene26641_All, CL5095.Contig1_All, CL7124.Contig2_All, Unigene6197_All, Unigene1544_All, CL1735.Contig2_All, CL3809.Contig2_All, Unigene28039_All, CL7124.Contig1_All, CL9081.Contig1_All, CL4657.Contig1_All, Unigene7850_All, Unigene2497_All, CL9081.Contig2_All
45	Glycine, serine and threonine metabolism	CL9784.Contig1_All, CL6939.Contig2_All, CL7944.Contig3_All, CL5398.Contig1_All, CL3469.Contig5_All, CL8146.Contig4_All, CL6506.Contig1_All, Unigene26422_All, CL4939.Contig1_All, Unigene27764_All, CL674.Contig5_All, CL3469.Contig1_All, Unigene5748_All, CL7409.Contig1_All, CL5346.Contig1_All
46	Histidine metabolism	CL9784.Contig1_All, Unigene6758_All, CL8398.Contig1_All, CL1079.Contig1_All, CL8398.Contig3_All, CL9955.Contig1_All, Unigene8408_All, CL331.Contig1_All, CL2598.Contig1_All, CL8306.Contig1_All, CL1028.Contig2_All, CL6098.Contig1_All, CL8306.Contig2_All, CL923.Contig1_All
47	Ascorbate and aldarate metabolism	CL9784.Contig1_All, CL9384.Contig2_All, Unigene6758_All, CL8398.Contig1_All, CL1079.Contig1_All, CL4435.Contig1_All, CL8398.Contig3_All, CL3687.Contig3_All, CL9955.Contig1_All, Unigene8408_All, CL331.Contig1_All, CL6295.Contig2_All, CL2598.Contig1_All, CL1028.Contig2_All, CL6098.Contig1_All, CL923.Contig1_All
48	Terpenoid backbone biosynthesis	CL9394.Contig2_All, CL6249.Contig2_All, CL6224.Contig2_All,

		Unigene1853_All, CL9394.Contig1_All, CL6224.Contig1_All, Unigene3291_All
49	Cyanoamino acid metabolism	CL2660.Contig1_All, CL4623.Contig1_All, CL8736.Contig1_All, Unigene24991_All, CL3332.Contig1_All, Unigene909_All, CL5903.Contig1_All, Unigene22507_All
50	Antigen processing and presentation	Unigene2628_All, CL8369.Contig2_All, Unigene4264_All, Unigene7683_All, CL2702.Contig3_All, CL2258.Contig1_All, Unigene2513_All, CL3263.Contig2_All, CL924.Contig2_All, CL2139.Contig2_All, Unigene4979_All, CL9252.Contig1_All, Unigene7737_All, CL6059.Contig2_All, CL2258.Contig13_All, CL7190.Contig1_All
51	Cysteine and methionine metabolism	CL6328.Contig3_All, CL8564.Contig2_All, CL6130.Contig1_All, CL8146.Contig4_All, CL6328.Contig1_All, Unigene26422_All, CL8564.Contig1_All, Unigene28617_All, Unigene3207_All, CL7282.Contig3_All, Unigene6153_All, CL5346.Contig1_All, CL5082.Contig2_All
52	Small cell lung cancer	CL8738.Contig2_All, Unigene26102_All, Unigene5913_All, CL5497.Contig1_All, Unigene4416_All, CL4294.Contig3_All, Unigene26870_All, CL170.Contig5_All, Unigene636_All, CL3920.Contig3_All, CL342.Contig5_All, CL572.Contig1_All, CL9534.Contig1_All
53	Fat digestion and absorption	Unigene8379_All, CL9400.Contig1_All, CL6249.Contig2_All, CL6224.Contig2_All, CL4126.Contig1_All, CL3242.Contig2_All, CL6224.Contig1_All, CL5082.Contig2_All
54	Tuberculosis	CL7321.Contig2_All, Unigene5913_All, CL768.Contig3_All, CL2717.Contig2_All, CL9293.Contig2_All, CL3279.Contig1_All, CL846.Contig2_All, CL9626.Contig1_All, CL846.Contig4_All, Unigene74_All, Unigene4416_All, CL3471.Contig2_All, Unigene6964_All, CL3471.Contig1_All, Unigene917_All, CL9776.Contig1_All, Unigene11930_All, CL6161.Contig2_All, CL7321.Contig1_All, CL7190.Contig1_All
55	Cardiac muscle contraction	Unigene22279_All, Unigene23921_All, CL1393.Contig2_All, CL834.Contig6_All, CL817.Contig7_All, CL4233.Contig1_All, Unigene11200_All, CL3904.Contig1_All, CL5977.Contig2_All, CL9631.Contig2_All, Unigene18768_All, CL3305.Contig4_All, Unigene6334_All, Unigene23599_All, Unigene2447_All, CL1393.Contig4_All, Unigene4349_All, CL2825.Contig2_All, Unigene18266_All, CL9631.Contig1_All, Unigene26416_All, CL2891.Contig3_All, Unigene4625_All, Unigene26244_All, CL834.Contig3_All, Unigene6024_All, CL2891.Contig1_All, Unigene26585_All, Unigene940_All, Unigene22202_All, CL4098.Contig2_All, CL9915.Contig2_All, CL1573.Contig11_All, CL5253.Contig1_All, CL9932.Contig2_All, Unigene7623_All
56	Riboflavin metabolism	CL2029.Contig1_All, Unigene3191_All, CL7288.Contig1_All
57	Homologous recombination	Unigene7602_All, Unigene22773_All, Unigene2458_All
58	Phenylalanine, tyrosine and tryptophan biosynthesis	CL8564.Contig2_All, CL8564.Contig1_All, CL5082.Contig2_All
59	Polyketide sugar unit biosynthesis	CL5044.Contig1_All
60	Collecting duct acid secretion	CL9723.Contig2_All, CL846.Contig2_All, CL554.Contig1_All, CL846.Contig4_All, CL2170.Contig4_All, CL2170.Contig3_All, CL6826.Contig1_All, CL3382.Contig2_All, CL6826.Contig2_All, CL554.Contig2_All
61	Sphingolipid metabolism	CL2664.Contig1_All, CL4004.Contig3_All, Unigene24033_All, CL9626.Contig1_All, CL2713.Contig5_All, CL4768.Contig1_All, CL445.Contig1_All, Unigene8351_All
62	Renin-angiotensin system	CL3787.Contig1_All, Unigene22014_All, CL4271.Contig1_All, CL9692.Contig2_All, CL997.Contig1_All, CL8471.Contig1_All, CL234.Contig2_All, CL4271.Contig3_All, CL959.Contig2_All, Unigene2773_All, Unigene6249_All, CL4456.Contig3_All, CL8471.Contig2_All
63	Steroid hormone biosynthesis	Unigene4206_All, CL9384.Contig2_All, CL4435.Contig1_All, CL3687.Contig3_All, CL3888.Contig1_All, CL7557.Contig1_All, CL9739.Contig2_All, CL6295.Contig2_All, CL7452.Contig3_All, CL1735.Contig2_All, Unigene2804_All, CL9739.Contig1_All
64	Nicotinate and nicotinamide metabolism	Unigene7583_All, Unigene6644_All, CL3291.Contig1_All,

		CL4442.Contig1_All, CL3641.Contig1_All
65	Neuroactive ligand-receptor interaction	Unigene3239_All, CL8578.Contig1_All
66	D-Arginine and D-ornithine metabolism	CL3469.Contig5_All, CL3469.Contig1_All
67	Selenocompound metabolism	CL1096.Contig6_All, CL8146.Contig4_All, CL1096.Contig2_All
68	Toxoplasmosis	CL8738.Contig2_All, Unigene26102_All, Unigene5913_All, CL4520.Contig1_All, CL2702.Contig3_All, CL9189.Contig2_All, CL5497.Contig1_All, Unigene4416_All, CL4294.Contig3_All, CL8520.Contig1_All, CL9189.Contig1_All, Unigene26870_All, Unigene7737_All, CL170.Contig5_All, Unigene636_All, CL3920.Contig3_All, CL342.Contig5_All, CL572.Contig1_All
69	Synaptic vesicle cycle	CL9723.Contig2_All, CL2717.Contig2_All, CL7292.Contig4_All, CL2220.Contig1_All, CL846.Contig2_All, CL554.Contig1_All, CL846.Contig4_All, CL2220.Contig2_All, CL56.Contig2_All, CL56.Contig1_All, CL2170.Contig4_All, CL2170.Contig3_All, CL6826.Contig1_All, CL3382.Contig2_All, CL6826.Contig2_All, CL554.Contig2_All, CL7292.Contig2_All
70	Drug metabolism—other enzymes	CL9384.Contig2_All, CL7976.Contig1_All, Unigene7473_All, CL5005.Contig5_All, CL4435.Contig1_All, CL9413.Contig1_All, CL3687.Contig3_All, CL9406.Contig1_All, CL6973.Contig1_All, CL953.Contig1_All, CL4316.Contig2_All, CL3242.Contig2_All, Unigene4061_All, CL4316.Contig1_All, CL6295.Contig2_All, CL7503.Contig1_All, CL7664.Contig1_All, Unigene26882_All, CL4016.Contig2_All, CL9338.Contig1_All
71	Pyrimidine metabolism	CL2570.Contig3_All, Unigene7583_All, CL9768.Contig1_All, CL9413.Contig1_All, Unigene26750_All, CL6341.Contig2_All, Unigene26882_All, CL4442.Contig1_All, CL9240.Contig1_All, CL4904.Contig1_All, CL4016.Contig2_All, CL4695.Contig3_All, Unigene4852_All
72	Fatty acid biosynthesis	CL3136.Contig3_All, CL986.Contig2_All, CL6637.Contig1_All, CL3136.Contig1_All
73	Valine, leucine and isoleucine biosynthesis	CL9862.Contig1_All, CL5475.Contig3_All, CL7342.Contig1_All, CL4474.Contig1_All
74	Prostate cancer	Unigene27409_All, CL3833.Contig2_All, Unigene6389_All, Unigene28933_All, CL5462.Contig1_All, Unigene27153_All, Unigene21637_All, CL2476.Contig2_All, CL8338.Contig2_All, CL3833.Contig1_All, Unigene5482_All, Unigene28054_All, CL8622.Contig1_All, CL5638.Contig2_All, Unigene26168_All, CL1922.Contig2_All, Unigene21428_All, CL1922.Contig1_All
75	Phagosome	Unigene2628_All, CL9723.Contig2_All, CL768.Contig3_All, CL2717.Contig2_All, CL8369.Contig2_All, CL846.Contig2_All, CL554.Contig1_All, CL846.Contig4_All, CL4807.Contig4_All, CL4807.Contig1_All, Unigene2513_All, CL7.Contig1_All, CL3263.Contig2_All, Unigene18599_All, CL9252.Contig1_All, Unigene23556_All, Unigene4416_All, CL2170.Contig4_All, CL1117.Contig2_All, CL2170.Contig3_All, CL6059.Contig2_All, CL170.Contig5_All, CL6826.Contig1_All, CL7.Contig8_All, CL1117.Contig1_All, CL3382.Contig2_All, CL6826.Contig2_All, Unigene5916_All, CL6161.Contig2_All, CL7945.Contig2_All, CL554.Contig2_All, CL7190.Contig1_All
76	Amino sugar and nucleotide sugar metabolism	CL2780.Contig3_All, CL200.Contig3_All, CL7505.Contig2_All, CL9247.Contig2_All, CL9247.Contig1_All, Unigene6820_All, Unigene26386_All, Unigene22288_All, CL200.Contig1_All, CL3342.Contig3_All, CL3342.Contig1_All, Unigene28039_All, CL5044.Contig1_All
77	Linoleic acid metabolism	CL5661.Contig1_All, Unigene7838_All, Unigene118_All, Unigene104_All, CL3821.Contig1_All, CL1735.Contig2_All, Unigene6696_All, CL4169.Contig1_All, CL4657.Contig1_All
78	Other glycan degradation (no map in kegg database)	Unigene27831_All, CL2664.Contig1_All, CL3400.Contig1_All, CL445.Contig1_All, Unigene4235_All
79	One carbon pool by folate	CL9173.Contig2_All, CL7944.Contig3_All, CL9025.Contig2_All, CL8152.Contig1_All, CL9173.Contig1_All
80	Protein digestion and absorption	CL3787.Contig1_All, CL3123.Contig2_All, CL3904.Contig1_All, CL9692.Contig2_All, CL997.Contig1_All, Unigene4221_All, CL7620.Contig1_All, CL785.Contig2_All, Unigene6964_All, CL2070.Contig1_All, CL7620.Contig2_All, Unigene3343_All,

81	Dorso-ventral axis formation	CL1573.Contig11_All, CL5253.Contig1_All, CL9932.Contig2_All CL7073.Contig3_All, Unigene643_All, CL7073.Contig1_All, CL2683.Contig2_All
82	Lysine biosynthesis	CL9784.Contig1_All
83	Staphylococcus aureus infection	Unigene4416_All
84	Epithelial cell signaling in Helicobacter pylori infection	CL9723.Contig2_All, CL2717.Contig2_All, CL846.Contig2_All, CL554.Contig1_All, CL846.Contig4_All, CL2170.Contig4_All, CL2170.Contig3_All, CL6826.Contig1_All, CL3382.Contig2_All, CL6826.Contig2_All, CL554.Contig2_All
85	Amoebiasis	CL8738.Contig2_All, Unigene26102_All, CL2444.Contig1_All, CL5497.Contig1_All, Unigene4416_All, CL4294.Contig3_All, CL8906.Contig1_All, Unigene26870_All, Unigene9848_All, Unigene636_All, CL3920.Contig3_All, CL6112.Contig1_All, CL6092.Contig4_All, CL342.Contig5_All, CL572.Contig1_All
86	Protein processing in endoplasmic reticulum	Unigene2628_All, CL8369.Contig2_All, Unigene258_All, CL2702.Contig3_All, CL9117.Contig2_All, CL7455.Contig2_All, Unigene1029_All, CL1271.Contig1_All, CL3263.Contig2_All, CL924.Contig2_All, CL2139.Contig2_All, CL5962.Contig1_All, CL5634.Contig1_All, CL3691.Contig1_All, CL5962.Contig3_All, CL2896.Contig1_All, Unigene2739_All, CL4380.Contig1_All, Unigene9848_All, Unigene7737_All, CL2896.Contig2_All, Unigene24026_All, CL7455.Contig1_All, Unigene4556_All, Unigene161_All, CL2139.Contig1_All, CL2034.Contig1_All, CL6092.Contig4_All, Unigene3641_All, Unigene5916_All, CL6999.Contig1_All, CL5550.Contig3_All, CL954.Contig1_All, CL8075.Contig1_All, CL8659.Contig2_All, CL3585.Contig1_All, CL5550.Contig1_All, CL9458.Contig1_All, CL1922.Contig2_All, CL1922.Contig1_All, CL179.Contig1_All
87	N-Glycan biosynthesis	CL3577.Contig2_All, CL9117.Contig2_All, CL5550.Contig3_All, CL5550.Contig1_All
88	Other types of O-glycan biosynthesis (no map in kegg database)	CL9384.Contig2_All, CL4435.Contig1_All, CL3687.Contig3_All, CL6295.Contig2_All
89	Taurine and hypotaurine metabolism	CL2660.Contig1_All, CL3332.Contig1_All
90	Nucleotide excision repair	CL3399.Contig3_All, CL954.Contig1_All
91	Steroid biosynthesis	CL2981.Contig1_All, CL3242.Contig2_All
92	Folate biosynthesis	CL6219.Contig1_All, CL9953.Contig1_All
93	Adipocytokine signaling pathway	CL6377.Contig3_All, CL5436.Contig1_All, CL3010.Contig1_All, CL5436.Contig6_All, CL1060.Contig3_All, CL986.Contig2_All, CL3034.Contig2_All, CL3953.Contig2_All, CL4667.Contig1_All, CL3034.Contig1_All
94	Pathways in cancer	CL8738.Contig2_All, Unigene26102_All, Unigene5913_All, Unigene27409_All, CL817.Contig7_All, CL3833.Contig2_All, Unigene6389_All, CL5374.Contig3_All, Unigene28933_All, CL5462.Contig1_All, Unigene27153_All, CL1925.Contig4_All, CL5497.Contig1_All, Unigene21637_All, CL2476.Contig2_All, Unigene4416_All, CL8338.Contig2_All, CL4294.Contig3_All, Unigene26870_All, CL3833.Contig1_All, Unigene5482_All, Unigene28054_All, CL8622.Contig1_All, CL5638.Contig2_All, CL170.Contig5_All, Unigene636_All, Unigene26168_All, CL3920.Contig3_All, CL8485.Contig1_All, CL342.Contig5_All, CL572.Contig1_All, CL1922.Contig2_All, CL9534.Contig1_All, Unigene21428_All, CL1922.Contig1_All
95	Dilated cardiomyopathy	CL8738.Contig2_All, Unigene23921_All, CL1393.Contig2_All, CL834.Contig6_All, CL817.Contig7_All, CL4233.Contig1_All, CL9631.Contig2_All, Unigene18768_All, CL5886.Contig1_All, Unigene23599_All, CL1393.Contig4_All, Unigene4349_All, CL601.Contig1_All, Unigene18266_All, CL9631.Contig1_All, Unigene23556_All, Unigene26416_All, Unigene4416_All, CL1117.Contig2_All, CL2891.Contig3_All, CL4294.Contig3_All, Unigene31479_All, Unigene26870_All, CL2986.Contig1_All, CL834.Contig3_All, CL8953.Contig2_All, Unigene6024_All, CL170.Contig5_All, CL2891.Contig1_All, Unigene940_All, CL1117.Contig1_All, Unigene22202_All, CL342.Contig5_All, CL7945.Contig2_All, Unigene4305_All, CL1573.Contig11_All, CL601.Contig2_All

96	Hypertrophic cardiomyopathy (HCM)	CL8738.Contig2_All, Unigene23921_All, CL1393.Contig2_All, CL834.Contig6_All, CL817.Contig7_All, CL4233.Contig1_All, CL9631.Contig2_All, Unigene18768_All, CL5886.Contig1_All, Unigene23599_All, CL1393.Contig4_All, Unigene4349_All, CL601.Contig1_All, Unigene18266_All, CL9631.Contig1_All, Unigene23556_All, Unigene26416_All, Unigene4416_All, CL1117.Contig2_All, CL2891.Contig3_All, CL4294.Contig3_All, Unigene31479_All, Unigene26870_All, CL2986.Contig1_All, CL834.Contig3_All, CL8953.Contig2_All, Unigene6024_All, CL170.Contig5_All, CL2891.Contig1_All, Unigene940_All, CL1117.Contig1_All, Unigene22202_All, CL342.Contig5_All, CL7945.Contig2_All, Unigene4305_All, CL1573.Contig11_All, CL601.Contig2_All
97	Arrhythmogenic right ventricular cardiomyopathy (ARVC)	CL8738.Contig2_All, CL5886.Contig1_All, Unigene23556_All, Unigene4416_All, CL1117.Contig2_All, CL2891.Contig3_All, CL4294.Contig3_All, Unigene26870_All, CL170.Contig5_All, CL2891.Contig1_All, CL1117.Contig1_All, CL342.Contig5_All, CL7945.Contig2_All, CL1573.Contig11_All
98	Regulation of actin cytoskeleton	CL8998.Contig1_All, Unigene23921_All, CL2444.Contig1_All, CL1393.Contig2_All, CL834.Contig6_All, CL1424.Contig1_All, CL4233.Contig1_All, Unigene18768_All, CL5886.Contig1_All, Unigene26445_All, Unigene23599_All, CL2986.Contig2_All, Unigene5927_All, CL1393.Contig4_All, Unigene4349_All, CL5387.Contig2_All, CL7391.Contig3_All, Unigene18266_All, Unigene9846_All, Unigene23556_All, Unigene26416_All, Unigene4416_All, CL1117.Contig2_All, CL8235.Contig2_All, Unigene15532_All, CL834.Contig3_All, CL8974.Contig1_All, Unigene6024_All, CL1444.Contig2_All, CL8953.Contig1_All, CL170.Contig5_All, CL165.Contig1_All, Unigene940_All, CL1117.Contig1_All, Unigene22202_All, CL5387.Contig1_All, CL8307.Contig1_All, CL7945.Contig2_All, CL165.Contig3_All
99	Mineral absorption	CL3904.Contig1_All, CL5108.Contig2_All, CL7299.Contig1_All, CL7299.Contig2_All, CL1573.Contig11_All, CL5253.Contig1_All, CL9932.Contig2_All
100	Hematopoietic cell lineage	CL3787.Contig1_All, CL9692.Contig2_All, CL997.Contig1_All, CL8471.Contig1_All, Unigene23556_All, CL234.Contig2_All, CL959.Contig2_All, Unigene6249_All, CL8471.Contig2_All
101	Proximal tubule bicarbonate reclamation	CL3798.Contig1_All, CL3904.Contig1_All, CL2699.Contig3_All, CL1060.Contig3_All, CL2699.Contig1_All, CL8268.Contig2_All, CL8268.Contig3_All, CL5253.Contig1_All, CL9932.Contig2_All
102	Primary bile acid biosynthesis	CL3932.Contig2_All, CL860.Contig3_All, CL9412.Contig2_All, Unigene3383_All, CL7324.Contig1_All, CL8987.Contig2_All, CL3932.Contig1_All, Unigene326_All
103	Glycosphingolipid biosynthesis—ganglio series	CL2664.Contig1_All
104	Vitamin B6 metabolism	CL6506.Contig1_All
105	Inositol phosphate metabolism	CL5576.Contig2_All, CL8538.Contig1_All, CL3037.Contig5_All, CL3037.Contig1_All, CL8538.Contig2_All
106	Type I diabetes mellitus	CL3471.Contig2_All, CL3471.Contig1_All
107	Cell adhesion molecules (CAMs)	CL2459.Contig21_All, CL5886.Contig1_All, CL5005.Contig5_All, Unigene4416_All, Unigene4061_All, CL170.Contig5_All
108	Viral myocarditis	CL8738.Contig2_All, Unigene23921_All, Unigene5913_All, CL1393.Contig2_All, CL834.Contig6_All, CL4233.Contig1_All, Unigene18768_All, Unigene23599_All, CL1393.Contig4_All, Unigene18266_All, Unigene26416_All, Unigene4416_All, CL1117.Contig2_All, CL4294.Contig3_All, Unigene26870_All, CL834.Contig3_All, Unigene6024_All, Unigene940_All, CL1117.Contig1_All, Unigene22202_All, CL342.Contig5_All, CL7945.Contig2_All
109	NOD-like receptor signaling pathway	CL1922.Contig2_All, Unigene21286_All, CL1922.Contig1_All
110	Systemic lupus erythematosus	CL1969.Contig11_All, Unigene8638_All, CL9820.Contig2_All, CL9820.Contig1_All
111	Retrograde endocannabinoid signaling	CL4623.Contig1_All, CL4746.Contig3_All, Unigene5441_All, CL7683.Contig1_All, CL8736.Contig1_All, Unigene24991_All, CL1742.Contig1_All, Unigene909_All, CL5903.Contig1_All,

		Unigene22507_All
112	Vibrio cholerae infection	CL9723.Contig2_All, CL2717.Contig2_All, CL846.Contig2_All, CL554.Contig1_All, CL846.Contig4_All, CL2170.Contig4_All, CL1117.Contig2_All, CL4380.Contig1_All, CL2170.Contig3_All, CL6826.Contig1_All, CL1117.Contig1_All, CL3382.Contig2_All, CL6826.Contig2_All, Unigene5916_All, CL7945.Contig2_All, CL554.Contig2_All
113	Sulfur metabolism	CL6328.Contig3_All, CL6328.Contig1_All
114	Pantothenate and CoA biosynthesis	CL9413.Contig1_All, CL1660.Contig4_All, CL4016.Contig2_All
115	Ether lipid metabolism	CL6227.Contig2_All, CL6227.Contig1_All, CL5345.Contig1_All
116	Focal adhesion	CL8738.Contig2_All, CL8998.Contig1_All, Unigene26102_All, CL2444.Contig1_All, CL1167.Contig1_All, Unigene6097_All, Unigene28711_All, CL5886.Contig1_All, CL9927.Contig1_All, Unigene26445_All, CL2986.Contig2_All, Unigene4349_All, CL5497.Contig1_All, Unigene23556_All, Unigene4416_All, CL1117.Contig2_All, CL4294.Contig3_All, Unigene26870_All, CL785.Contig2_All, CL8235.Contig2_All, Unigene15532_All, CL8974.Contig1_All, CL2070.Contig1_All, Unigene23665_All, CL8953.Contig1_All, CL170.Contig5_All, Unigene636_All, CL3920.Contig3_All, CL1117.Contig1_All, CL342.Contig5_All, CL7945.Contig2_All, CL572.Contig1_All, CL8274.Contig3_All
117	Purine metabolism	CL2570.Contig3_All, CL9064.Contig1_All, CL3108.Contig1_All, Unigene7583_All, CL200.Contig3_All, CL9025.Contig2_All, CL3033.Contig1_All, CL6973.Contig1_All, CL3108.Contig2_All, CL3070.Contig4_All, CL2764.Contig2_All, CL4316.Contig2_All, CL200.Contig1_All, CL4316.Contig1_All, CL6129.Contig1_All, CL3070.Contig5_All, CL3342.Contig3_All, CL4442.Contig1_All, CL9064.Contig2_All, CL3342.Contig1_All, CL6382.Contig1_All, Unigene4852_All
118	Glycosaminoglycan degradation	CL2664.Contig1_All
119	Autoimmune thyroid disease	Unigene4061_All
120	Caffeine metabolism	Unigene7473_All
121	Ubiquinone and other terpenoid-quinone biosynthesis	CL573.Contig1_All, CL573.Contig2_All, CL6584.Contig1_All, Unigene28629_All, CL222.Contig1_All, CL8754.Contig2_All, CL6584.Contig3_All
122	Shigellosis	CL2444.Contig1_All, Unigene5927_All, CL6184.Contig1_All, Unigene9846_All, Unigene23556_All, Unigene4416_All, CL1117.Contig2_All, CL170.Contig5_All, CL165.Contig1_All, CL1117.Contig1_All, CL7945.Contig2_All, CL165.Contig3_All
123	RNA degradation	CL7321.Contig2_All, CL2464.Contig3_All, CL4990.Contig2_All, Unigene5313_All, Unigene743_All, CL3471.Contig2_All, CL4078.Contig3_All, Unigene8351_All, CL2464.Contig2_All, CL2667.Contig2_All, CL3471.Contig1_All, CL7321.Contig1_All
124	Bile secretion	CL9384.Contig2_All, CL4520.Contig1_All, CL3904.Contig1_All, CL5647.Contig3_All, CL4435.Contig1_All, CL3687.Contig3_All, CL5647.Contig4_All, CL5647.Contig1_All, CL6295.Contig2_All, CL5522.Contig1_All, CL6435.Contig1_All, CL5253.Contig1_All, CL9932.Contig2_All
125	Type II diabetes mellitus	CL6377.Contig3_All, CL3070.Contig4_All, CL3070.Contig5_All
126	Ribosome	CL6235.Contig1_All, Unigene8100_All, Unigene6132_All, Unigene985_All, Unigene6155_All, Unigene1584_All, Unigene72_All, Unigene518_All, CL4434.Contig2_All, CL7042.Contig1_All, CL9310.Contig1_All, Unigene10193_All, CL9705.Contig1_All, Unigene3766_All, Unigene6057_All, Unigene5292_All, CL9005.Contig2_All, Unigene8017_All, Unigene2664_All, CL580.Contig2_All, CL6189.Contig1_All, CL10028.Contig2_All, Unigene3663_All, Unigene3962_All, Unigene9538_All, CL6235.Contig3_All, CL1933.Contig2_All, Unigene6078_All, Unigene3508_All, CL4434.Contig1_All, CL6029.Contig1_All, CL5329.Contig5_All, CL5329.Contig2_All, CL10028.Contig1_All, CL933.Contig2_All, Unigene4454_All, Unigene7882_All, Unigene2648_All, CL602.Contig2_All, CL7042.Contig2_All, CL2875.Contig1_All, CL5922.Contig1_All, CL5922.Contig2_All, Unigene2821_All, Unigene6108_All, CL9705.Contig2_All, CL1778.Contig2_All, Unigene2668_All

127	Leishmaniasis	Unigene4416_All, CL170.Contig5_All
128	p53 signaling pathway	Unigene5913_All, CL9534.Contig1_All
129	Endocytosis	Unigene6284_All, CL4520.Contig1_All, CL2702.Contig3_All, CL2220.Contig1_All, CL305.Contig4_All, CL5815.Contig2_All, CL5815.Contig1_All, CL2220.Contig2_All, CL8137.Contig2_All, CL305.Contig1_All, Unigene1552_All, CL4429.Contig3_All, CL5446.Contig2_All, CL4429.Contig1_All, Unigene4013_All, CL56.Contig2_All, CL1389.Contig3_All, CL56.Contig1_All, CL3782.Contig4_All, Unigene7737_All, CL5446.Contig1_All, CL9533.Contig1_All, CL1389.Contig1_All
130	Pertussis	CL7391.Contig3_All, Unigene23556_All, Unigene4416_All, CL170.Contig5_All
131	Malaria	Unigene4416_All
132	D-Glutamine and D-glutamate metabolism	CL3798.Contig1_All
133	Sulfur relay system	Unigene6153_All
134	Glycosphingolipid biosynthesis - globo series	CL4768.Contig1_All
135	Prion diseases	CL2702.Contig3_All, CL924.Contig2_All, Unigene9167_All, CL6127.Contig1_All, Unigene7737_All, CL6446.Contig2_All
136	Bacterial invasion of epithelial cells	CL2444.Contig1_All, CL1167.Contig1_All, CL2220.Contig1_All, Unigene5927_All, CL6184.Contig1_All, CL2220.Contig2_All, Unigene23556_All, Unigene4416_All, CL1117.Contig2_All, CL8974.Contig1_All, CL170.Contig5_All, CL1117.Contig1_All, CL7945.Contig2_All
137	Proteasome	CL1188.Contig2_All, CL4290.Contig2_All, CL4290.Contig1_All, CL4251.Contig1_All, CL8007.Contig3_All, CL9490.Contig2_All, CL7377.Contig3_All, CL3142.Contig1_All, CL7988.Contig3_All, CL3484.Contig1_All, CL1188.Contig1_All, CL2950.Contig3_All, CL7988.Contig1_All
138	Carbohydrate digestion and absorption	CL3904.Contig1_All, Unigene29100_All, Unigene4471_All, CL5253.Contig1_All, CL9932.Contig2_All
139	Base excision repair	CL3399.Contig3_All
140	Mucin type O-Glycan biosynthesis	CL7008.Contig2_All
141	Amyotrophic lateral sclerosis (ALS)	Unigene5244_All, Unigene5913_All, Unigene2081_All, Unigene21797_All, Unigene9167_All, Unigene27273_All, CL8520.Contig1_All, Unigene28505_All, CL824.Contig2_All
142	Salmonella infection	Unigene23921_All, CL1393.Contig2_All, CL834.Contig6_All, CL4233.Contig1_All, Unigene6097_All, Unigene28711_All, Unigene18768_All, Unigene23599_All, CL4807.Contig4_All, Unigene5927_All, CL4807.Contig1_All, CL1393.Contig4_All, Unigene18266_All, Unigene9846_All, Unigene26416_All, CL1117.Contig2_All, CL834.Contig3_All, Unigene6024_All, Unigene23665_All, CL165.Contig1_All, Unigene940_All, CL1117.Contig1_All, Unigene22202_All, CL7945.Contig2_All, CL165.Contig3_All
143	Insulin signaling pathway	CL8998.Contig1_All, CL213.Contig4_All, CL1962.Contig4_All, CL3136.Contig3_All, CL1962.Contig2_All, CL213.Contig3_All, CL1060.Contig3_All, CL986.Contig2_All, CL6637.Contig1_All, CL5329.Contig5_All, CL8235.Contig2_All, CL5329.Contig2_All, CL3809.Contig2_All, CL3136.Contig1_All, Unigene22681_All
144	Aldosterone-regulated sodium reabsorption	CL3904.Contig1_All, Unigene21272_All, CL5253.Contig1_All, CL9932.Contig2_All
145	mRNA surveillance pathway	CL8998.Contig1_All, CL3630.Contig2_All, CL4990.Contig2_All, Unigene584_All, CL2538.Contig1_All, CL710.Contig1_All, CL2538.Contig3_All, CL3630.Contig1_All, CL9523.Contig1_All, CL8235.Contig2_All, Unigene2366_All, CL2667.Contig2_All
146	Vitamin digestion and absorption	CL1619.Contig2_All
147	Bladder cancer	CL9534.Contig1_All
148	Protein export	Unigene22058_All, CL924.Contig2_All, CL3028.Contig1_All, CL4477.Contig1_All, Unigene5916_All
149	RNA transport	CL3630.Contig2_All, CL4990.Contig2_All, CL918.Contig3_All, Unigene584_All, CL8571.Contig2_All, CL7369.Contig2_All, CL7369.Contig1_All, Unigene29198_All, CL879.Contig2_All, CL3741.Contig3_All, CL1554.Contig3_All, CL4513.Contig2_All, CL8155.Contig3_All, CL550.Contig1_All, CL5015.Contig1_All, CL457.Contig1_All, CL457.Contig2_All, CL3630.Contig1_All,

		CL9523.Contig1_All, Unigene2366_All, CL1554.Contig2_All, Unigene4231_All, CL3741.Contig1_All, CL2667.Contig2_All, Unigene5006_All, CL550.Contig2_All, CL9532.Contig2_All, CL4513.Contig1_All
150	Calcium signaling pathway	CL5576.Contig2_All, Unigene26445_All, CL9626.Contig1_All, CL2986.Contig2_All, CL9189.Contig2_All, CL477.Contig1_All, Unigene686_All, CL5446.Contig2_All, CL2891.Contig3_All, CL9189.Contig1_All, Unigene15532_All, CL4242.Contig1_All, CL5446.Contig1_All, CL8953.Contig1_All, CL4242.Contig2_All, CL2891.Contig1_All, CL1573.Contig11_All
151	Ribosome biogenesis in eukaryotes	CL4524.Contig3_All, CL5800.Contig2_All, CL9760.Contig1_All, CL4429.Contig3_All, CL5800.Contig1_All, CL4429.Contig1_All, CL9523.Contig1_All, Unigene703_All, CL9760.Contig2_All
152	Transcriptional misregulation in cancer	Unigene3189_All, Unigene8425_All, CL1969.Contig11_All
153	Apoptosis	Unigene5913_All, CL1962.Contig4_All, CL1962.Contig2_All
154	Legionellosis	Unigene5913_All, CL2702.Contig3_All, Unigene4416_All, CL3471.Contig2_All, Unigene7737_All, CL8483.Contig2_All, CL3471.Contig1_All
155	RIG-I-like receptor signaling pathway	CL1181.Contig3_All
156	Taste transduction	Unigene5441_All
157	Glycerophospholipid metabolism	Unigene1879_All, CL7291.Contig1_All, CL4126.Contig1_All, CL2415.Contig1_All, CL1951.Contig2_All, CL1951.Contig1_All, Unigene601_All
158	Fc gamma R-mediated phagocytosis	CL6377.Contig3_All, CL9626.Contig1_All, Unigene5927_All, CL5387.Contig2_All, CL7391.Contig3_All, Unigene1552_All, CL1444.Contig2_All, CL5387.Contig1_All, CL8307.Contig1_All
159	Herpes simplex infection	CL2239.Contig1_All, CL8998.Contig1_All, Unigene5913_All, Unigene29319_All, CL1474.Contig1_All, CL214.Contig3_All, CL8235.Contig2_All, CL4562.Contig4_All
160	mTOR signaling pathway	CL5329.Contig5_All, CL5329.Contig2_All
161	Measles	Unigene8642_All, CL6377.Contig3_All, CL2702.Contig3_All, Unigene7737_All, CL9534.Contig1_All
162	Vascular smooth muscle contraction	CL8998.Contig1_All, CL6377.Contig3_All, Unigene23921_All, CL1393.Contig2_All, CL834.Contig6_All, CL4233.Contig1_All, Unigene18768_All, Unigene26445_All, Unigene23599_All, CL2986.Contig2_All, CL1393.Contig4_All, Unigene18266_All, Unigene26416_All, CL8235.Contig2_All, Unigene15532_All, CL834.Contig3_All, Unigene6024_All, CL8953.Contig1_All, Unigene940_All, Unigene22202_All, Unigene2623_All
163	Melanoma	CL9534.Contig1_All
164	HTLV-I infection	Unigene2628_All, CL5150.Contig2_All, CL8369.Contig2_All, CL9927.Contig1_All, Unigene686_All, CL3263.Contig2_All, CL5150.Contig1_All, Unigene3239_All, Unigene4416_All, CL4242.Contig1_All, CL4242.Contig2_All, CL3399.Contig3_All, CL9534.Contig1_All
165	Endocrine and other factor-regulated calcium reabsorption	CL3904.Contig1_All, CL2220.Contig1_All, CL2220.Contig2_All, CL56.Contig2_All, CL56.Contig1_All, CL1573.Contig11_All, CL5253.Contig1_All, CL9932.Contig2_All
166	Gastric acid secretion	CL1424.Contig1_All, CL3904.Contig1_All, Unigene26445_All, CL2986.Contig2_All, CL1117.Contig2_All, Unigene15532_All, CL8953.Contig1_All, CL1117.Contig1_All, CL7945.Contig2_All, CL5253.Contig1_All, CL9932.Contig2_All
167	Vasopressin-regulated water reabsorption	CL4807.Contig4_All, CL4807.Contig1_All, Unigene4171_All
168	Leukocyte transendothelial migration	CL2444.Contig1_All, CL1424.Contig1_All, Unigene4349_All, Unigene4416_All, CL1117.Contig2_All, CL8974.Contig1_All, CL170.Contig5_All, CL1117.Contig1_All, CL7945.Contig2_All
169	Renal cell carcinoma	CL5374.Contig3_All, CL8485.Contig1_All
170	Pancreatic cancer	CL1925.Contig4_All, CL9534.Contig1_All
171	Thyroid cancer	CL817.Contig7_All
172	ABC transporters	CL8183.Contig3_All, CL4247.Contig1_All, CL701.Contig1_All
173	Serotonergic synapse	CL5661.Contig1_All, Unigene7838_All, Unigene5441_All, CL3821.Contig1_All, Unigene6696_All, CL4169.Contig1_All
174	Toll-like receptor signaling pathway	CL8520.Contig1_All
175	GABAergic synapse	CL6614.Contig2_All, Unigene5441_All, CL7038.Contig1_All, CL6614.Contig1_All

176	Cell cycle	CL5150.Contig2_All, CL5150.Contig1_All, CL3399.Contig3_All, CL9534.Contig1_All
177	NF-kappa B signaling pathway (no map in kegg database)	CL6377.Contig3_All
178	Jak-STAT signaling pathway	CL3782.Contig4_All
179	VEGF signaling pathway	CL9626.Contig1_All, Unigene9848_All, CL8974.Contig1_All, CL6092.Contig4_All
180	Phototransduction—fly	Unigene5441_All, CL1117.Contig2_All, CL1117.Contig1_All, CL7945.Contig2_All
181	Influenza A	Unigene5913_All, CL2702.Contig3_All, Unigene686_All, CL710.Contig1_All, CL1117.Contig2_All, CL8520.Contig1_All, CL9523.Contig1_All, Unigene7737_All, CL7083.Contig2_All, CL4242.Contig2_All, CL1117.Contig1_All, CL8075.Contig1_All, CL7945.Contig2_All
182	Alcoholism	CL8998.Contig1_All, Unigene5441_All, CL1969.Contig11_All, CL8235.Contig2_All, Unigene8638_All
183	Progesterone-mediated oocyte maturation	CL5150.Contig2_All, CL5150.Contig1_All
184	Chronic myeloid leukemia	CL9534.Contig1_All
185	Spliceosome	CL2239.Contig1_All, Unigene4216_All, CL3630.Contig2_All, CL3606.Contig2_All, CL2702.Contig3_All, Unigene29319_All, CL7930.Contig3_All, Unigene584_All, Unigene3189_All, Unigene8425_All, Unigene25931_All, CL166.Contig1_All, CL1474.Contig1_All, CL214.Contig3_All, CL3630.Contig1_All, Unigene2366_All, CL4562.Contig4_All, Unigene7737_All, Unigene5979_All, CL171.Contig2_All, Unigene1550_All, CL3606.Contig8_All
186	Adherens junction	CL2444.Contig1_All, CL6709.Contig2_All, CL1117.Contig2_All, CL1117.Contig1_All, CL7945.Contig2_All
187	Tight junction	CL6377.Contig3_All, Unigene23921_All, CL1393.Contig2_All, CL834.Contig6_All, CL4233.Contig1_All, Unigene18768_All, CL2148.Contig1_All, Unigene23599_All, CL2414.Contig2_All, CL6184.Contig1_All, CL1393.Contig4_All, Unigene4349_All, CL7859.Contig1_All, Unigene18266_All, Unigene26416_All, CL1117.Contig2_All, CL2148.Contig2_All, CL834.Contig3_All, Unigene6024_All, Unigene940_All, CL1117.Contig1_All, Unigene22202_All, CL7945.Contig2_All, CL9534.Contig1_All
188	MAPK signaling pathway	Unigene6097_All, CL2702.Contig3_All, Unigene28711_All, CL8549.Contig2_All, CL6847.Contig1_All, CL8520.Contig1_All, Unigene4951_All, Unigene9848_All, Unigene7737_All, Unigene23665_All, CL6092.Contig4_All, Unigene2489_All
189	Endometrial cancer	CL1167.Contig1_All
190	Colorectal cancer	Unigene5913_All
191	Pathogenic Escherichia coli infection	CL1424.Contig1_All, Unigene5927_All, CL6184.Contig1_All, CL7.Contig1_All, Unigene18599_All, Unigene4416_All, CL1117.Contig2_All, CL170.Contig5_All, CL7.Contig8_All, CL1117.Contig1_All, CL7945.Contig2_All
192	Amphetamine addiction	CL8998.Contig1_All, CL8235.Contig2_All
193	Oocyte meiosis	CL8998.Contig1_All, CL5150.Contig2_All, CL5150.Contig1_All, CL8235.Contig2_All
194	Epstein-Barr virus infection	CL1188.Contig2_All, CL2702.Contig3_All, CL7377.Contig3_All, CL3142.Contig1_All, CL7988.Contig3_All, CL8520.Contig1_All, Unigene9848_All, Unigene7737_All, CL1188.Contig1_All, CL2950.Contig3_All, CL6092.Contig4_All, CL7988.Contig1_All
195	Non-small cell lung cancer	CL9534.Contig1_All
196	Pancreatic secretion	CL3904.Contig1_All, CL2891.Contig3_All, CL3242.Contig2_All, CL2891.Contig1_All, CL5253.Contig1_All, CL9932.Contig2_All
197	Morphine addiction	Unigene5441_All
198	Glioma	CL9534.Contig1_All
199	Axon guidance	CL7391.Contig3_All, Unigene4416_All, CL170.Contig5_All
200	Fc epsilon RI signaling pathway	CL6377.Contig3_All, CL8520.Contig1_All
201	Hepatitis C	CL4520.Contig1_All
202	Salivary secretion	CL3904.Contig1_All, Unigene27913_All, CL5253.Contig1_All, CL9932.Contig2_All
203	Ubiquitin mediated proteolysis	CL5150.Contig2_All, CL7930.Contig3_All, CL5150.Contig1_All, Unigene6239_All, Unigene29387_All, CL8485.Contig1_All

204	ErbB signaling pathway	CL5446.Contig2_All, CL5446.Contig1_All
205	Long-term potentiation	CL8998.Contig1_All, CL8235.Contig2_All
206	Chagas disease (American trypanosomiasis)	CL3263.Contig2_All
207	Natural killer cell mediated cytotoxicity	Unigene4416_All
208	Phosphatidylinositol signaling system	CL5576.Contig2_All
209	Wnt signaling pathway	Unigene8436_All, CL2595.Contig1_All, CL2595.Contig3_All
210	Neurotrophin signaling pathway	CL6377.Contig3_All, Unigene4171_All
211	T cell receptor signaling pathway	CL6377.Contig3_All, CL9534.Contig1_All
212	GnRH signaling pathway	CL6377.Contig3_All, CL8520.Contig1_All
213	Gap junction	CL7.Contig1_All, Unigene18599_All, CL7.Contig8_All
214	Chemokine signaling pathway	CL6377.Contig3_All, Unigene5441_All, CL8974.Contig1_All
215	Dopaminergic synapse	CL8998.Contig1_All, Unigene5441_All, CL8235.Contig2_All
216	Glutamatergic synapse	Unigene5441_All, CL3469.Contig1_All
217	Cholinergic synapse	Unigene5441_All

Table S7. Primer sequences for qRT-PCR.

Gene	Forward Primer (5′–3′)	Reverse Primer (5′–3′)
CL8625.Contig1	ATACATCGGCTTTCACAGGC	ACAAAACGGCCTACCACTTG
CL8429.Contig1	TGGCACCTCTAGCCTCAAAT	GGGGACTCATGGAGAACAGA
CL5894.Contig1	AAAGAGGAAAATAGCGACGAGTG	GCGACTAGAACCTGTTGGGAGTAT
CL5488.Contig2	TTCCCTACCCAGTCAGCGTG	ACGGGACAGGTTTGTTCACAG
CL5080.Contig2	CTTGCATCGTGGGAATTCTT	TGTGCCATTTTCCACTTTCA
CL4567.Contig1	CACGGGCTTGTTTATTGACC	CACTCAAACTGCAGCCACAT
CL3263.Contig1	AATGGACAAAGTCGTGAGGC	AACTCGAGGAAAGCAGACCA
CL2270.Contig1	ATGGGTGCAATGTCACAAGA	CTTCCTAGCGTAAACAGCGG
CL2264.Contig1	GCCTATGCATTAAGGGGTCA	TCTTCCATTGTTTTCCCTCG
Unigene2394	GCTGAGGACTCCAGCATCAT	TTTGCCAGCGTTGTTTACTG
Uni2628gene	TGACAACTGGTGGTATGGAAGG	TGACTGTTACTTTCGTCGTGGC
Unigene7511	AACAAGCTCAACGGCAAAGT	AAACTGGTTGCATGGAAAGC
β-tub	TGACAACGAAGCCCTTTACGAC	CAGGCATGAAGAAGTGGAGACG

Table S8. Primer sequences for RNAi. The T7 promoter sequences were underlined.

Primer Names	Primer Sequences (5′-3′)
*FAR*T7 forward	TAATACGACTCACTATAGGGAAAGACTCGGCTCGGA
FAR reverse	TCTGGTGTAAGATTACATTAA
FAR forward	AAAGACTCGGCTCGGA
*FAR*T7 reverse	TAATACGACTCACTATAGGGGTCTGGTGTAAGATTACATTAA
*ENG*T7 forward	TAATACGACTCACTATAGGGTCTTTCATTCGGATTTGC
ENG reverse	CGTCCATTGTGGCCTTCT
ENG forward	TCTTTCATTCGGATTTGC
*ENG*T7 reverse	TAATACGACTCACTATAGGGCGTCCATTGTGGCCTTCT
*GFP*T7 forward	TAATACGACTCACTATAGGGAAAGGAGAAGAACTTTTCAC
GFP reverse	CTGTTACAAACTCAAGAAGG
GFP forward	AAAGGAGAAGAACTTTTCAC
*GFP*T7 reverse	TAATACGACTCACTATAGGGCTGTTACAAACTCAAGAAGG
β-tub forward	TGACAACGAAGCCCTTTACGAC
β-tub reverse	CAGGCATGAAGAAGTGGAGACG
